# Applications of simple and accessible methods for meta-analysis involving rare events: A simulation study

**DOI:** 10.1177/09622802211022385

**Published:** 2021-06-17

**Authors:** Alexander Hodkinson, Evangelos Kontopantelis

**Affiliations:** 1 National Institute for Health Research (NIHR) School for Primary Care Research, Manchester Academic Health Science Centre, University of Manchester, Manchester, UK; 2 Division of Informatics, Imaging & Data Sciences, Faculty of Biology, Medicine & Health, University of Manchester, Manchester, UK

**Keywords:** Rare events, adverse events, heterogeneity, meta-analysis, statistical power, safety, few studies, random effects

## Abstract

Meta-analysis of clinical trials targeting rare events face particular challenges when the data lack adequate number of events and are susceptible to high levels of heterogeneity. The standard meta-analysis methods (DerSimonian Laird (DL) and Mantel–Haenszel (MH)) often lead to serious distortions because of such data sparsity. Applications of the methods suited to specific incidence and heterogeneity characteristics are lacking, thus we compared nine available methods in a simulation study. We generated 360 meta-analysis scenarios where each considered different incidences, sample sizes, between-study variance (heterogeneity) and treatment allocation. We include globally recommended methods such as inverse-variance fixed/random-effect (IV-FE/RE), classical-MH, MH-FE, MH-DL, Peto, Peto-DL and the two extensions for MH bootstrapped-DL (bDL) and Peto-bDL. Performance was assessed on mean bias, mean error, coverage and power. In the absence of heterogeneity, the coverage and power when combined revealed small differences in meta-analysis involving rare and very rare events. The Peto-bDL method performed best, but only in smaller sample sizes involving rare events. For medium-to-larger sample sizes, MH-bDL was preferred. For meta-analysis involving very rare events, Peto-bDL was the best performing method which was sustained across all sample sizes. However, in meta-analysis with 20% or more heterogeneity, the coverage and power were insufficient. Performance based on mean bias and mean error was almost identical across methods. To conclude, in meta-analysis of rare binary outcomes, our results suggest that Peto-bDL is better in both rare and very rare event settings in meta-analysis with limited sample sizes. However, when heterogeneity is large, the coverage and power to detect rare events are insufficient. Whilst this study shows that some of the less studied methods appear to have good properties under sparse data scenarios, further work is needed to assess them against the more complex distributional-based methods to understand their overall performances.

## 1 Introduction

Meta-analysis (MAs) of binary data encounter problems when proportions of events are few.^1^ This is a particular issue in MAs of adverse events that are associated with biomedical interventions.^2^ Difficulties often arise when analysis is done either at patient level using individual patient data or at the study level using just aggregate data from each trial. We concentrate here on MAs of study-level summaries, which is far more common in the assessment of adverse events, though patient-level analysis is to be preferred when data are available.^3^

The methods used when performing MAs of binary data are frequently done using the standard inverse-variance fixed-effects model which is based on large-sample normal approximation, or fixed-effects methods based on exact distributional theory such as the Mantel–Haenszel (MH)^4^ or Peto model,^5^ or the standard random-effects DerSimonian–Laird (DL) model.^6^ Because these methods lack power to investigate the incidence of rare events and are mostly based on large sample normal approximation particularly inverse-variance,^7,8^ their statistical properties for estimating treatment effects are often judged as suboptimal either through results being biased, confidence intervals being inappropriately wide or statistical power being too low to detect any true differences. One leading cause of this bias is the estimation of the between-study variance (τ^2^),^9^ which often displays uncertainty in MAs when there are few studies involved.^10^

Several simulation studies have evaluated the performances of these mainstream methods for MAs^11–13^ and shown that the estimate of τ^2^ is particularly inaccurate when the number of included studies is small. However, evidence of heterogeneity estimation across different sample size settings with varying low levels of incidence (i.e. rare events) and imbalanced treatment allocations is currently lacking.

The Cochrane guidelines (Version 6.1, 2020) recommend the use of methods which are mostly accessible in Review Manager (RevMan); software developed by the Nordic Cochrane Centre and is free-to-access.^8^ Specifically, the guideline suggests that when the event rate is below 1%,^14^ the ‘Peto odds ratio’ method is considered the least biased and most powerful method and provides the best confidence interval coverage.^5^ The method is also thought to perform well when treatment and control group sizes within studies are balanced and treatment effects are small. In other circumstances, when event risks are above 1% and for MAs involving many studies with imbalanced treatment groups; the MH odds ratio (OR) without continuity correction, logistic regression and exact methods are considered to be better performing.^15^ However, there are two shortcomings when using these methods: (i) not all of them are available in RevMan, in particular, the MH without continuity correction, logistic regression and exact methods, and (ii) when heterogeneity are present, meta-analysts often have to revert from inverse-variance weighting to a random-effects DL, to reduce bias in estimation. But, there are still some obvious shortcomings of random-effects methods, as they are based on large-sample variance approximation.^1^

Most recently, there have been several new methods proposed for improved estimation of τ^2^. These include maximum likelihood, profile likelihood and restricted maximum likelihood or non-parametric ‘permutations’ method.^16^ More specifically, a non-parametric bootstrap of the DL estimator was shown to be a better performer in small MAs that were falsely assumed to be homogenous under the standard DL model.^12,17^ This non-parametric bootstrap of the DL has now been extended for both the MH and Peto models, but very little is known about the performances of these methods in MAs involving rare events whilst compounded with the issue of heterogeneity. As these methods are easily accessible and applicable, it is important to assess whether they could support or improve the current recommendations on MAs of rare events.

The focus of this study is to evaluate the use of mainstream fixed- and random-effects MAs methods including two non-parametric bootstrap extensions for analysing rare or very rare outcomes, in a simulation study covering typical scenarios for rare adverse events or rare diseases. The paper is organised as follows. In section 2, we descriptively assess other similar simulation studies to highlight research gaps and limitations, which we are attempting to address in this work. In section 3, we discuss the various meta-analytic methods used for estimating relevant model parameters. In section 4, we report on the simulation study and introduce the tools used to measure the performance of the methods across the simulated scenarios. In section 5, findings are illustrated in tables or graphically, and in section 6, we conclude and provide recommendations for practical work in the future.

## 2 Literature review of simulation studies on rare events

Several simulation studies have looked to assess the performance of MAs methods in clinical trials targeting rare events (see Table S1, online Appendix 1). However, these studies had mostly included methods based on exact distributional assumptions, were limited to certain values of incidence and did not explicitly assess the performances of measurement error based on varying values of heterogeneity. For example, in one study,^18^ only methods that include double-zero studies (i.e. studies which report no event in treatment and control arm) and avoid continuity correction were included; and so the standard methods as outlined in the Cochrane handbook were not of primary concern.^8^ The study only used small values for τ^2^ (0–0.806) across the simulated scenarios, limiting the knowledge for performances of the methods based on different heterogeneity values. A second study^10^ evaluated heterogeneity across three newly derived methods including a simple (unweighted) average treatment effect estimator, a new heterogeneity estimator and a parametric bootstrapping test. Only two values of τ^2^ (0 and 1.2) were explored in this study which again limit the performance evaluation for higher levels of heterogeneity; and results reveal that the new derived methods showed poor performance in their ability to detect heterogeneity anyway, yielding biased overall treatment estimates. Another study^19^ using the same simple average method as in the aforementioned study^10^ showed similar results with τ^2^ fixed at 0.5. Other simulation studies^19–22^ assumed no heterogeneity in the treatment effects, and three studies^15,23,24^ had used a real data set where the true effect and heterogeneity levels were unknown, and hence the studies were limited in the context of comparing methods.

## 3 Statistical methods for MAs of rare data

The following methods described were used in our simulation study because they met our criteria: (i) simple to implement (i.e. a lay trained person with basic MAs training could apply them), (ii) are mentioned in the Cochrane handbook with the exception of the Peto/MH bootstrap methods and (iii) because of their accessibility in free and/or mainstream statistical software such as RevMan, Stata or R.^25–27^

In each subsection heading, we provide the name of the method and, in the parentheses, its abbreviation in the results figures/tables and the statistical software packages (with commands) can be used for parameter estimation. The summations in all of the equations are over *i*, from 1 to the number of patients *N*, and *k* represents the total number of studies, unless otherwise specified.

When analysing rare events and binary data in particular, the most commonly encountered effect measure used in clinical trials is the OR. But, it is important to note that this effect measure is generally found to be approximately the same as the relative risk when the outcome of interest is rare.^28^ However, because the Peto method is only designed upon the OR, this prompted the use of OR for effect estimation throughout even though it is often misinterpreted as being equivalent to the relative risk.^29^ But, it is worth noting that many of the other methods can be analysed using relative risk.

In all MAs of *k* studies involving binary data, the results of each study can be presented in a 2 × 2 table (see Table 1).

**Table 1.** Binary data from one trial.

Study *k*	Event	No event	Total patients
Experimental	$a_{i}$	$b_{i}$	$n_{1i}$
Control	$c_{i}$	$d_{i}$	$n_{2i}$
			N

Note: *i* denotes the patient, *k* denotes the study and N denotes the total number of patients in that specific study.

Then, the OR from each study using Table 1 is given by

(1) $${\mathrm{OR}}_{i}=\frac{a_{i}d_{i}}{b_{i}c_{i}}$$

The standard error of the log OR being

$$se\left\{ln\left({OR}_{i}\right)\right\}=\;\sqrt{\frac{1}{a_{i}}+\frac{1}{b_{i}}+\frac{1}{c_{i}}+\frac{1}{d_{i}}}$$

### 3.1 Inverse-variance (IV) fixed effect (FE) and random effect (RE) [RevMan, R (meta, metafor), Stata (metan)]

The inverse-variance method is the simplest approach to MAs, where the weights given to each study are the inverse of the variance of the effect estimate (i.e. one over the square of its standard error). Thus, larger studies which have smaller standard errors are given more weight than smaller studies, which have larger standard errors. This choice of weight minimizes the imprecision (uncertainty) of the pooled effect estimate.

In the fixed-effects model, the weight (*w_i_*) is given as

(2) $$w_{i}=\frac{1}{{\left(se\left\{\hat{{OR}_{i}}\right\}\right)}^{2}}\;$$

This is then combined to give a summary estimate

(3) $$\hat{{OR}_{IV-FE}}=\frac{\sum w_{i}{\hat{OR}}_{i}}{\sum w_{i}}$$

with

(4) $$se\left\{\hat{{OR}_{IV-FE}}\right\}=\frac{1}{\sqrt{\sum w_{i}}}$$

The heterogeneity statistic is given by the following formula

$$Q_{IV-FE}=\sum w_{i}{\left(\hat{{OR}_{i}}-\;\hat{{OR}_{IV-FE}}\right)}^{2}$$

Under the null hypothesis that there are no differences in intervention effects among studies, this follows a chi-squared distribution with k−1 degrees of freedom (where *k* is the number of studies contributing to the MAs).${\;I}^{2}$ is calculated as

$${\;I}^{2}=\max\left\{100\%\times \frac{Q_{IV-FE}-\left(k-1\right)}{Q_{IV-FE}},\;0\right\}$$

In the random-effects analysis, each study is also weight by the inverse of it variance too, but the different is that the variance now includes the original (within-studies) variance plus the between-studies variance, tau-squared.

Concretely, under the random-effects model, the weight assigned to each study is

$$w_{i}=\frac{1}{V_{i}}$$

where *V_i_* is the within-study variance for study (*i*) plus the between-studies variance, tau-squared ($\tau^{2})$. That is

$$V_{i}=V_{i}+\;\tau^{2}$$

The weighted mean (${OR}_{IV-RE})$ is then computed as

(5) $$\hat{{OR}_{IV-RE}}=\frac{\sum w_{i}T_{i}}{\sum w_{i}}\;$$

where $T_{i}$ is the observed effect calculated by

$$T_{i}={OR}_{i}+\varepsilon_{i}=\mu +{ℶ}_{i}+\varepsilon_{i}$$

The ${OR}_{i}$ is the true effect, and $\varepsilon_{i}$ is the within study error. In turn, ${OR}_{i}$ is determined by the mean of all true effects, μ and the between-study error ${ℶ}_{i}$.

The standard error of the combined effect is then

(6) $$se\left\{\hat{{OR}_{IV-RE}}\right\}=\frac{1}{\sqrt{\sum w_{i}}}\;$$

The heterogeneity statistic is given by the following formula

$$Q_{IV-RE}=\;\sum w_{i}{\left(T_{i}-\;\hat{{OR}_{IV-RE}}\right)}^{2}$$

As is clearly outlined in the Cochrane handbook, because the IV method is based on large-sample variance approximation, they are not intended for use with rare events.^8^ But for consistency, we included both the IV fixed and random effects (IV-FE/IV-RE) in this simulation study as the baseline option. Whilst they have been shown as poor performers globally, they have at times been shown to be useful when comparing the performances against other methods.

### 3.2 Mantel–Haenszel

Unlike with IV methods, the MH estimation methods are considered the default fixed effect methods of MAs in RevMan, and they use a different weighting scheme dependent upon which effect measure is used (e.g. ORs, risk ratios and risk differences) to avoid the issue of normal approximation. MH is also preferred to inverse-variance methods, as they have been shown to have better statistical properties when there are few events, which is common among Cochrane and other reviews generally.

#### 3.2.1 Classical Mantel–Haenszel (MH) [RevMan, R (meta, metafor), Stata (metan, metaan)]

The classical Mantel–Haenszel^4^ method is used specifically for log OR and OR. Here, the MH log OR is given by

$$\ln\left({OR}_{MH}\right)=\ln\left(\frac{\sum w_{MH,i}{OR}_{i}}{\sum w_{MH,i}}\right)$$

and the MH OR is given by

(7) $${OR}_{MH}=\frac{\sum w_{MH,i}{OR}_{i}}{\sum w_{MH,i}}\;$$

where each study’s OR is given weight $w_{MH,i}$ = $\frac{b_{i}c_{i}}{N_{i}}$, *b_i_* is the number of non-events in the intervention group, *c_i_* is the number of events in the control group and *N*_i_ is the total number of patients as detailed in Table 1.

The log OR has standard error given by

(8) $$se\left\{\ln\left({OR}_{MH}\right)\right\}=\sqrt{\frac{1}{2}\left(\frac{E}{R^{2}}+\frac{F+G}{RS}+\frac{H}{S^{2}}\right)}\;$$

where

$$R=\sum \frac{a_{i}d_{i}}{N_{i}};\;S=\sum \frac{b_{i}c_{i}}{N_{i}}$$

$$E=\sum \frac{\left(a_{i}+\;d_{i}\right)a_{i}d_{i}}{N_{i}^{2}};F=\sum \frac{\left(a_{i}+\;d_{i}\right)b_{i}c_{i}}{N_{i}^{2}};$$

$$G=\sum \frac{\left(b_{i}+\;c_{i}\right)a_{i}d_{i}}{N_{i}^{2}};H=\sum \frac{\left(b_{i}+\;c_{i}\right)b_{i}c_{i}}{N_{i}^{2}}$$

The heterogeneity test statistics is given by

$$Q_{MH}=\;\sum w_{i}{\left({\hat{OR}}_{i}-\;\hat{{OR}_{MH}}\right)}^{2}$$

where ${\hat{OR}}_{i}$ represents the log OR and $w_{i}$ are the weights $w_{MH,i}=\;\frac{b_{i}c_{i}}{N_{i}}$. Under the null hypothesis that there are no differences in intervention effect among studies, this follows a chi-squared distributed with *k*−1 degrees of freedom.

The statistic *I*^2^ is calculated as

$${\;I}^{2}=\max\left\{100\%\times \frac{Q_{MH}-\left(k-1\right)}{Q_{MH}},\;0\right\}$$

#### 3.2.2 Mantel–Haenszel with fixed-effect weighting (MH-FE) [RevMan, R(meta, metafor), Stata (metaan)]

The MH-FE method differs to that of the classical MH method, with the use of a different weighting scheme. Namely, the weight from equation (2) is used rather than the weight detailed in section 3.2.1. Then, the heterogeneity test statistics are the same but using this new weight.

#### 3.2.3 Mantel–Haenszel with DL random-effects weighting (MH-DL) [RevMan, R (metafor), Stata (metaan)]

When data are sparse, either in terms of event rates being low or study size being small, the estimates of the standard errors of the effect estimates that are used in the inverse-variance weighting can be poor. A variation on the inverse-variance method is to incorporate an assumption that the different studies are estimating different, yet related, intervention effects. This produces a random-effects MAs, and the simplest version is known as the DerSimonian and Laird (DL) method.

The DL method is the oldest and most widely used random-effects MAs and has proven to be remarkably robust in various scenarios.^6^

Effect sizes are assumed to have a distribution of ${OR}_{i}\;∼\;N\left(OR,\;\tau^{2}\right)$, and the estimate of $\tau^{2}$ is given by

(9) $${\hat{\tau }}_{DL}^{2}=\max\left\{\;\frac{Q-(k-1)}{\sum_{i=1}^{k}\hat{w_{i}}-\sum_{i=1}^{k}\hat{w_{i}^{2}}/\sum_{i=1}^{k}\hat{w_{i}}\;},\;0\right\}$$

where the *w_i_* are the inverse-variance weights, calculated as $w_{i}^{\prime }=\frac{1}{{\mathrm{se}\left\{\hat{{OR}_{i}}\right\}}^{2}}$, *k* is the number of studies contributing to the MAs and Q is the heterogeneity statistic. For binary data, either $Q_{IV-FE/RE}$ or $Q_{MH}$ may be taken. Both are implemented in RevMan, and this is the only difference between random-effects methods under MH and IV options.

Each study’s effect size is given by the weight

(10) $$w_{i}=\frac{1}{{se\left\{\hat{{OR}_{i}}\right\}}^{2}+\hat{\tau_{DL}^{2}}}\;$$

The summary effect size is given by

(11) $$\hat{{OR}_{DL}}=\frac{\sum w_{i}{\hat{OR}}_{i}}{\sum w_{i}}\;$$

and

(12) $$se\left\{\hat{{OR}_{DL}}\right\}=\frac{1}{\sqrt{\sum w_{i}}}\;$$

where the heterogeneity statistic *Q* is less than or equal to its degrees of freedom (*k*−1), the estimate of the between study variation, $\hat{\tau_{DL}^{2}}$, is zero, and the weights coincide with those given by the IV method.

#### 3.2.4 Mantel–Haenszel with bootstrapped DL random-effects weighting (MH-bDL) [R (metafor), Stata (metaan)]

Kontopantelis et al.^12^ recently suggested a non-parametric bootstrap version of the DL method (bDL) by randomly sampling B sets of studies with replacement and then selecting the mean of the truncated estimates. In each set, the MH effect size is estimated as explained in section 3.2.3, and τ^2^ is estimated using the DL method (${\hat{\tau }}_{DL}^{2})$ from equation (9) and then is truncated if negative. ${\hat{\tau }}_{\mathrm{bDL}}^{2}$ is estimated as the mean of these B estimates.^17^

Whilst the MH-bDL method is not recommended by Cochrane, it has been shown to be a good performer in both detecting heterogeneity and returning more accurate overall effect estimates. However, its performance has not yet been extensively assessed in rare event settings, and so it was important to include in our simulation study.

### 3.3 Peto OR

#### 3.3.1 Peto OR with fixed-effect weighting (Peto) [R (metafor), Stata (metan, metaan)]

Peto’s method^5^ can only be used to pool ORs. It uses an inverse-variance approach but utilizes an approximate method of estimating the log OR and uses different weights.

The individual ORs are given by

(13) $${OR}_{\mathrm{Peto},i}=\exp\left\{\frac{X_{i}}{V_{i}}\right\}$$

where *X*_i_ is the ‘O – E’ observed minus expected statistic

$$X_{i}=a_{i}-E\left[a_{i}\right]$$

with the expected number of events in the experimental intervention group

$$E\left[a_{i}\right]=\frac{n_{1i}\left(a_{i}+c_{i}\right)}{N_{i}}$$

and the hypergeometric variance of *a_i_*

(14) $$V_{i}=\frac{n_{1i}n_{2i}\left(a_{i}+c_{i}\right)\left(b_{i}+d_{i}\right)}{N_{i}^{2}\left(N_{i}-1\right)}$$

The logarithm of the OR has standard error

(15) $$se\left\{\ln\left({OR}_{\mathrm{Peto},i}\right)\right\}=\;\sqrt{\frac{1}{V_{i}}}\;$$

Peto for combining summary log OR across studies is given by

$$\ln\left({OR}_{\mathrm{Peto}}\right)=\frac{\sum V_{i}ln\left({OR}_{\mathrm{Peto},i}\right)}{\sum V_{i}}$$

and the summary OR by

(16) $${OR}_{\mathrm{Peto}}=\exp\left\{\frac{\sum V_{i}ln\left({OR}_{\mathrm{Peto},i}\right)}{\sum V_{i}}\right\}\;$$

where the odds ratio ${OR}_{\mathrm{Peto},i}$ is calculated using the approximated method described in equation (13), and $V_{i}$ are the hypergeometric variances described in equation (14).

The heterogeneity statistic is given by

$$Q_{\mathrm{Peto}}=\;\sum V_{i}\left\{{\left(\ln{OR}_{\mathrm{Peto},i}\right)}^{2}-{\left(\ln{OR}_{\mathrm{Peto}}\right)}^{2}\right\}$$

#### 3.3.2 Peto OR with DL random-effects weighting (Peto-DL) [R (metafor or lme4), Stata (metaan)]

The summary Peto OR from section 3.3.1 is used for effect estimation, and ${\hat{\tau }}_{DL}^{2}$ is estimated using equation (9).

#### 3.3.3 Peto OR with bootstrapped DL random-effects weighting (Peto-bDL) [R (metafor), Stata (metaan)]

Again, equivalent to section 3.2.4, but this time using the τ^2^ which is estimated using the DL method (${\hat{\tau }}_{DL}^{2})$ from equation (9), then ${\hat{\tau }}_{\mathrm{bDL}}^{2}$ is estimated as the mean of B estimates. ${\hat{\tau }}_{\mathrm{bDL}}^{2}$ is truncated if negative.

### 3.4 Excluded methods

The following methods were excluded because they either could not be accessed in RevMan or were not included in the Cochrane guidelines: Binomial-normal hierarchical model,^30^ Poisson-normal hierarchical model,^31^ Poisson-Gamma Hierarchical Model^32^ or Beta-binomial model,^18^ Bayesian MAs including weak informative priors,^21^ Exact method based on combining CIs,^33^ Logistic regression^34,35^ and Arcsine difference.^36^

## 4 Simulation setup

The data sets are generated under the *ipdpower* command in Stata^37^ which calculates the power for mixed-effects aggregate (two-level) data from clinical trials. All definitions and calculations might be checked using the original code (online Appendix 2). To mirror real data, true values for the design factors were gathered where possible, from empirical data on performed MAs. Thus, the largest study to date includes 14,886 Cochrane reviews.^38^ Other meta-analyses^39–41^ of rare events were also used to help inform on the design.

An important point to appreciate when designing and analysing of simulation studies is that they are empirical experiments, meaning performance measures are themselves estimated, and estimates of performances are thus subject to error. This feature of simulation studies is often not widely appreciated.^42^ The implications can be two-fold. It is therefore important to present estimates of the simulation uncertainty in relation to *bias* and *error* estimation of the methods and consider the number of repetitions needed.

Monte Carlo standard errors are key to quantifying simulation uncertainty by providing a standard error of the estimate according to the number of simulations. We used this in our study to assess for simulation uncertainty.^43^ The design factors for the simulation design are explained in section 4.1, and the measures used to assess the performance of the methods and simulation uncertainty are explained in section 4.3.

### 4.1 Design factors

The following design factors were varied in the simulation study as follows:

- **Number of patients in a single study:** In a pivotal study that assessed the influence of trial sample size on binary treatment effect estimates within 93 MAs (involving 735 individual trials),^44^ the observed trial sample sizes varied among the MAs (median 34–2371 patients) and within MAs (e.g. trial sample size ranged from 106 to 48,835 patients in one MAs). With this in mind, we include sample size settings of 1500 to 50,000 patients among MAs. We choose to fix the number of patients in the simulations to allow for more consistency when reporting the results and when comparing across heterogeneity and the event incidence level.
- **Number of studies per MAs:** Given that the distribution of the number of studies in Cochrane^38^ and non-Cochrane^39^ studies vary from on average 5 and 23, respectively. We selected a maximum of 20 studies in all scenarios to avoid excessive simulation time. We also chose 3, 5, 7 and 10 studies for scenarios that would reflect that similar of Cochrane reviews involving few studies in MAs.
- **Degree of heterogeneity:** Between-study variance (τ^2^) on its own is perhaps not an efficient way to quantify heterogeneity, since the within variance estimate component (σ^2^) cannot be ignored. In logistic regression within Stata, the within-variance component is fixed to φ^2/3^ or 3.289668, which is central to the data generation mechanism with ‘*ipdpower*’.

Given τ^2^ = (*I*^2^ × φ^2/3^)/(100 − *I*^2^), then if

- *I*^2^=0% (no heterogeneity), then τ^2^=0.822467
- *I*^2^=20% (small heterogeneity), then τ^2^=0.822467
- *I*^2^=50% (medium heterogeneity), then τ^2^=3.289868
- *I*^2^=90% (high heterogeneity), then τ^2^=29.60881

Because other reviews^10,18,22^ have focused only on small values of heterogeneity, we therefore included scenarios of a higher degree of heterogeneity for complete coverage in meta-analyses, especially since heterogeneity tends to be underestimated.^12^

- **Probability of membership for the intervention and control (denoted as *r*):** For the treatment and control arm randomisation, we considered both 1:1 allocation (*r* = 0.5) and unbalanced allocations favouring intervention by *r* = 0.1 (10%–90%). Although a review paper has shown that 78% of clinical trials were conducted with equal patient allocation strategies,^45^ we include imbalanced allocation due to the unpredictable performances associated with Peto OR.^1^
- **Incidence of event:** We considered three different frequencies of rare events, Rare (≥0.01% to < 0.1%), Very rare (<0.01%) and Common (≥1% to < 10%) as defined by the World Health Organisations Council for International Organizations of Medical Sciences,^46^ European Medicines Agency^47^ and the Food and Drug Administration.^48^
- **Treatment effect-size:** In all simulation settings, we imagined the situation of MAs with the outcome being a rare adverse (or sparse) event where the treatment is aiming for a further lowering of events as compared with the control. As such, we consider the null hypothesis with an OR of 1 as the true treatment effect in the ‘no effect’ situation. In the medium effect situation, we use an OR of ln (0.5) = −0.69, which corresponds to the median OR from Turner et al.^38^

### 4.2 Simulation scenarios

Details of the simulation scenarios are shown in Table 2. In total, there were 360 MAs scenarios, each involving 1000 iterations to reduce simulation error. Due to the high number of iterations, it was necessary to use sophisticated in-house high-computational clustering to enable a wider range of scenarios.

**Table 2.** Parameter setup in different simulation scenarios.

Simulation scenarios	Number of patients	Number of studies	Between-study variance (τ^2^)^a^	Incidence rate of rare event (rare, very rare, non-rare)^a^	Probability of membership for intervention^a^
1	1500	3	0/0.822467/3.289868/29.60881	1/1000; 1/10000; 1/10	0.5/0.1
2	2500	5	0/0.822467/3.289868/29.60881	1/1000; 1/10000; 1/10	0.5/0.1
3	3000	3	0/0.822467/3.289868/29.60881	1/1000; 1/10000; 1/10	0.5/0.1
4	3500	7	0/0.822467/3.289868/29.60881	1/1000; 1/10000; 1/10	0.5/0.1
5	5000	5	0/0.822467/3.289868/29.60881	1/1000; 1/10000; 1/10	0.5/0.1
6	5000	10	0/0.822467/3.289868/29.60881	1/1000; 1/10000; 1/10	0.5/0.1
7	7000	7	0/0.822467/3.289868/29.60881	1/1000; 1/10000; 1/10	0.5/0.1
8	7500	3	0/0.822467/3.289868/29.60881	1/1000; 1/10000; 1/10	0.5/0.1
9	10000	10	0/0.822467/3.289868/29.60881	1/1000; 1/10000; 1/10	0.5/0.1
10	10000	20	0/0.822467/3.289868/29.60881	1/1000; 1/10000; 1/10	0.5/0.1
11	12500	5	0/0.822467/3.289868/29.60881	1/1000; 1/10000; 1/10	0.5/0.1
12	17500	7	0/0.822467/3.289868/29.60881	1/1000; 1/10000; 1/10	0.5/0.1
13	20000	20	0/0.822467/3.289868/29.60881	1/1000; 1/10000; 1/10	0.5/0.1
14	25000	10	0/0.822467/3.289868/29.60881	1/1000; 1/10000; 1/10	0.5/0.1
15	50000	20	0/0.822467/3.289868/29.60881	1/1000; 1/10000; 1/10	0.5/0.1

^a^Each of the parameters for heterogeneity, incidence and membership probability were simulated across all 15 scenarios.

### 4.3 Evaluating simulation performance

The following five measures were used to assess the performance of the nine methods on the simulated scenarios:

- *Mean error* is calculated as the aggregate of the ‘absolute difference’ in the estimate of treatment effect to the true parameter (z) expressed as
  $$\frac{1}{1000}\sum_{i=1}^{1000}\left|z-\hat{z_{i}}\right|$$
- *Mean bias* is the aggregate of the difference in the estimate to the true parameter (*z*) and is expressed as
  $$\frac{1}{1000}\sum_{i=1}^{1000}\left(z-\hat{z_{i}}\right)$$
- *Coverage* measures the percentage of the true treatment effects included in the available 95% confidence intervals over all generated data. This should theoretically be close to 95%.
- *Power* indicates the percentage of iterations in which a model coefficient was found to be statistically significant and in the hypothesized direction. Information is then aggregated across all simulated datasets to approximate the overall power.
- *Coverage and power* is a combined average across both measures. Because they are interlinked, it is fundamental and important to assess them simultaneously in this study.

## 5 Results

We present the results on the five performance measures separately, and in the final part, we provide a summary for application of the methods for practitioners.

### 5.1 Mean bias

**Figure 1.**
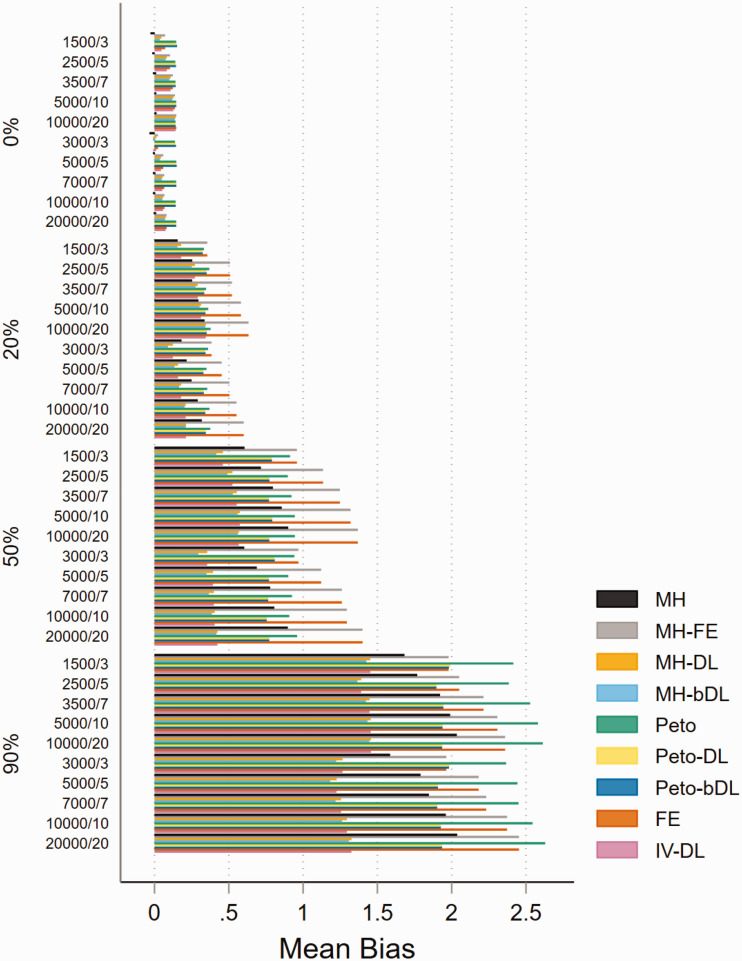
Mean bias of rare event scenarios with imbalanced treatment allocation (*r* = 0.1). The percentage values on the *y*-axis represent the heterogeneity group, i.e. 0%, 20%, 50% and 90%. The value within these groups on the *y*-axis represents the number of patients/studies in each meta-analysis scenario. All other scenarios are provided in the online Appendix. IV: inverse variance; FE: fixed effect;RE: random effect; DL: DerSimonian and Laird; MH: Mantel–Haenszel; bDL: bootstrapped DL.

For MAs involving ‘rare events’ with imbalanced patient randomisation to each treatment group (*r* = 0.1), the results show that when there is no heterogeneity, the pattern of mean bias is consistently low across all of the methods (Figure 1). However, with heterogeneity increasing, the mean bias performance worsened. The MH-DL and MH-bDL methods maintained the lowest levels of bias among greater levels of heterogeneity; this was true across all sample size settings. In MAs with a balanced treatment allocation ratio (*r* = 0.5) (online Figure S1), the mean bias was more modest across all values of heterogeneity, but the pattern was similar to MAs with imbalanced treatment ratio, with the exception of when heterogeneity was around 90%, where Peto-DL and Peto-bDL resulted in the lowest bias estimate. In MAs involving ‘very rare’ events unsurprisingly, the mean bias was greater across all methods and all values of heterogeneity (online Figures S2 and S3). However, in MAs displaying zero heterogeneity, Peto, Peto-DL, Peto-bDL and MH methods maintained the lowest mean bias; and in MAs with high levels of heterogeneity, only Peto-DL/bDL were able to maintain low desirable levels of bias. For non-rare events, the mean bias was similar but was higher across the different values of heterogeneity (online Figures S4 and S5).

### 5.2 Mean error

**Figure 2.**
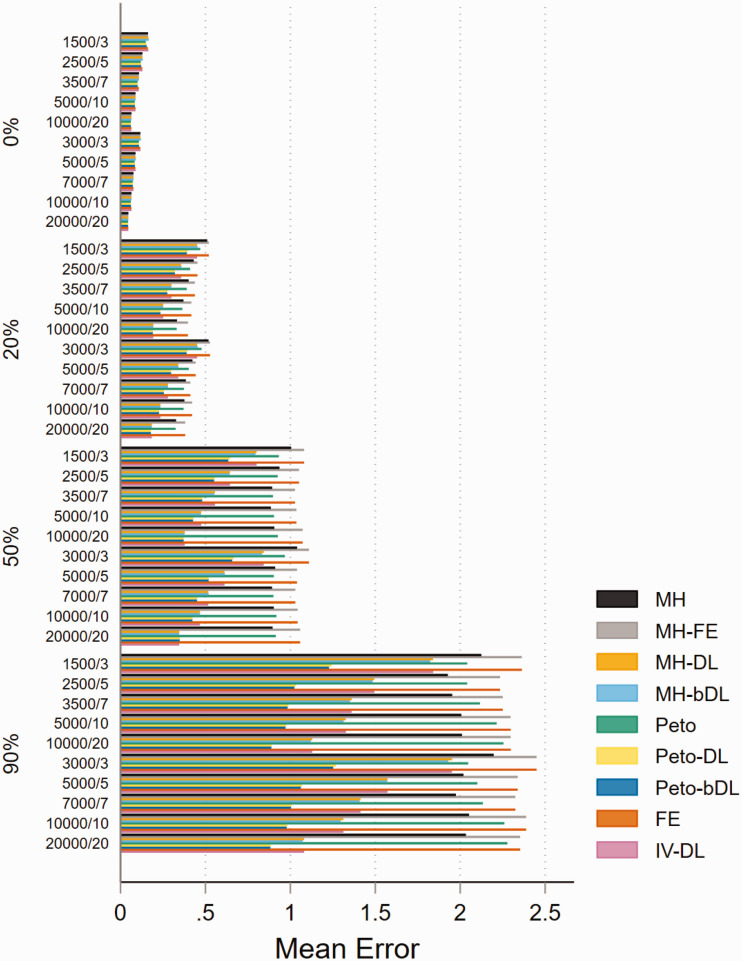
Mean error for rare event scenarios with balanced treatment allocation (*r* = 0.5). IV: inverse variance; FE: fixed effect; RE: random effect; DL: DerSimonian and Laird; MH: Mantel–Haenszel; bDL: bootstrapped DL.

The performance based on mean error was almost identical across both treatment allocation settings, and the mean error and heterogeneity estimates were positively associated as they increased. For MAs with balanced treatment allocations and involving rare events (Figure 2), the Peto-DL and Peto-bDL methods maintained the lowest mean error. This was more prevalent amongst MAs presenting with higher levels of heterogeneity. In contrast, MAs involving imbalanced treatment allocations (online Figure S6) revealed that MH-DL and MH-bDL were preferred. For very rare events, the mean error performance was similar in either treatment allocation setting; but the error rate was greater across all sample size settings than observed within MAs of rare events (online Figures S7 and S8). For none-rare events, the pattern remained consistent and the level of error was smaller than that observed for rare and very rare event settings (online Figures S9 and S10).

### 5.3 Coverage

**Figure 3.**
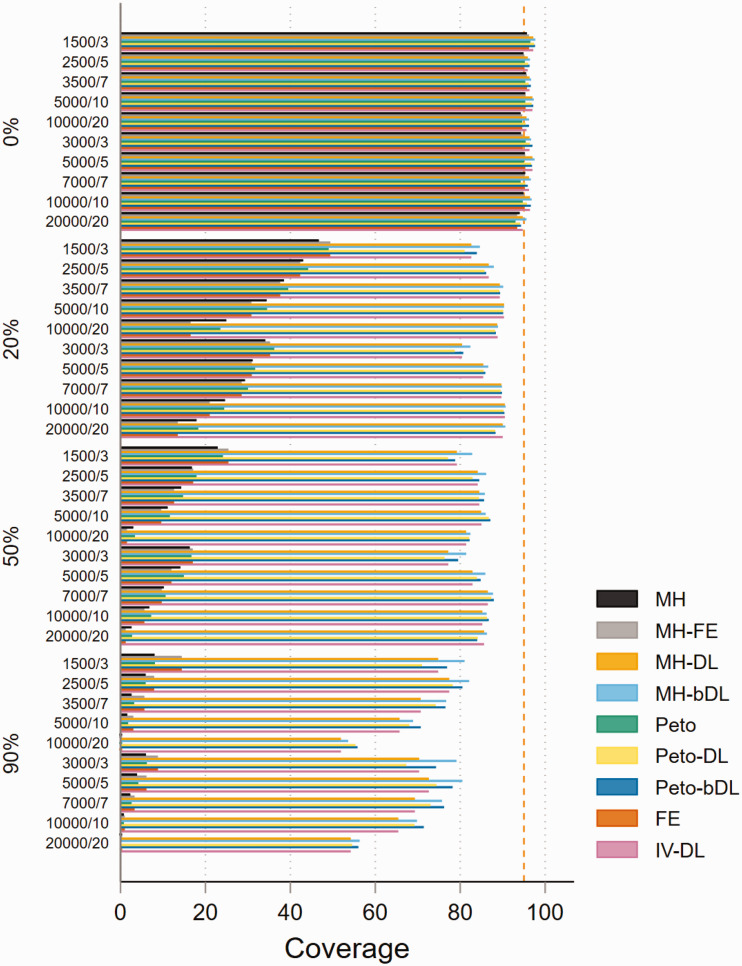
Coverage of rare event scenarios in meta-analysis with balanced treatment allocation (*r* = 0.5). IV: inverse variance; FE: fixed effect;RE: random effect; DL: DerSimonian and Laird; MH: Mantel–Haenszel; bDL: bootstrapped DL.

In the absence of heterogeneity, the performance of the methods based on coverage in MAs with rare events was consistently higher than the 95% level in all four methods (MH-DL, MH-bDL, Peto-DL and Peto-bDL). Performance was considerably better in MAs with balanced treatment allocation (Figure 3). Unsurprisingly, for high levels of heterogeneity, only the DL random-effects methods were able to maintain a coverage above 50%, this was true in both treatment allocation settings (see online Figure S11 for imbalanced setting). For MAs involving very rare events with balanced treatment allocation, the results were similar to that of MAs with rare events (online Figure S12). However, in MAs involving an imbalanced treatment allocation, the coverage varied somewhat across the different sample size settings and for different values of heterogeneity (online Figure S13). The best coverage across all sample size settings and heterogeneity scenarios were maintained by the Peto-DL and Peto-bDL. Coverage performance in non-rare events is shown in online Figures S14 and S15.

### 5.4 Power

**Figure 4.**
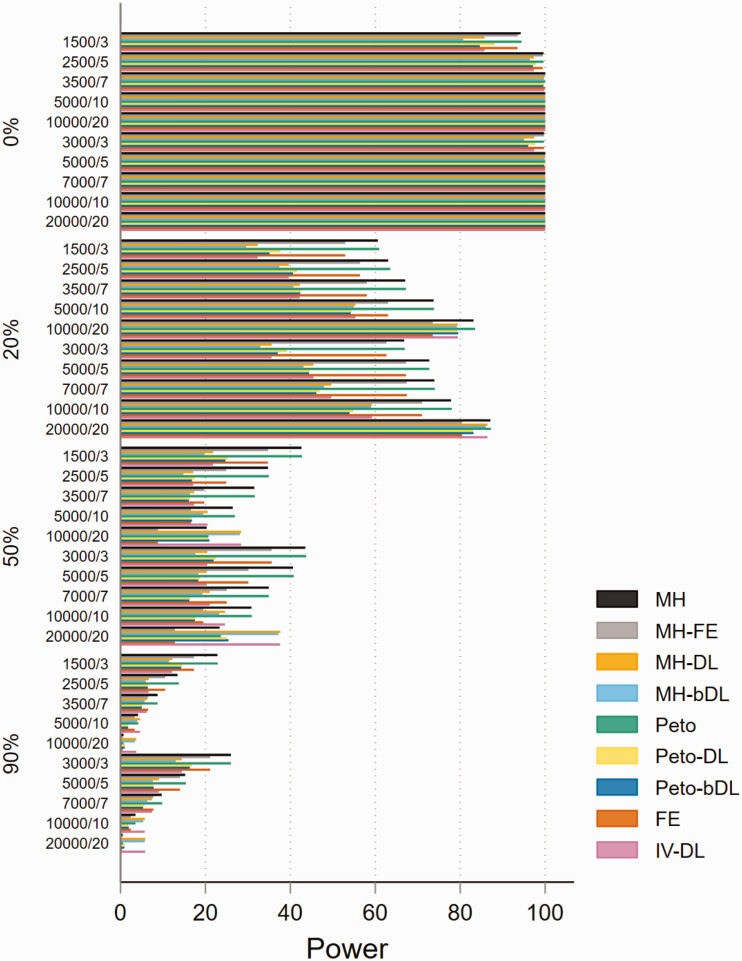
Power of rare event scenarios in meta-analysis with balanced treatment allocation (*r* = 0.5). IV: inverse variance; FE: fixed effect;RE: random effect; DL: DerSimonian and Laird; MH: Mantel–Haenszel; bDL: bootstrapped DL.

**Figure 5.**
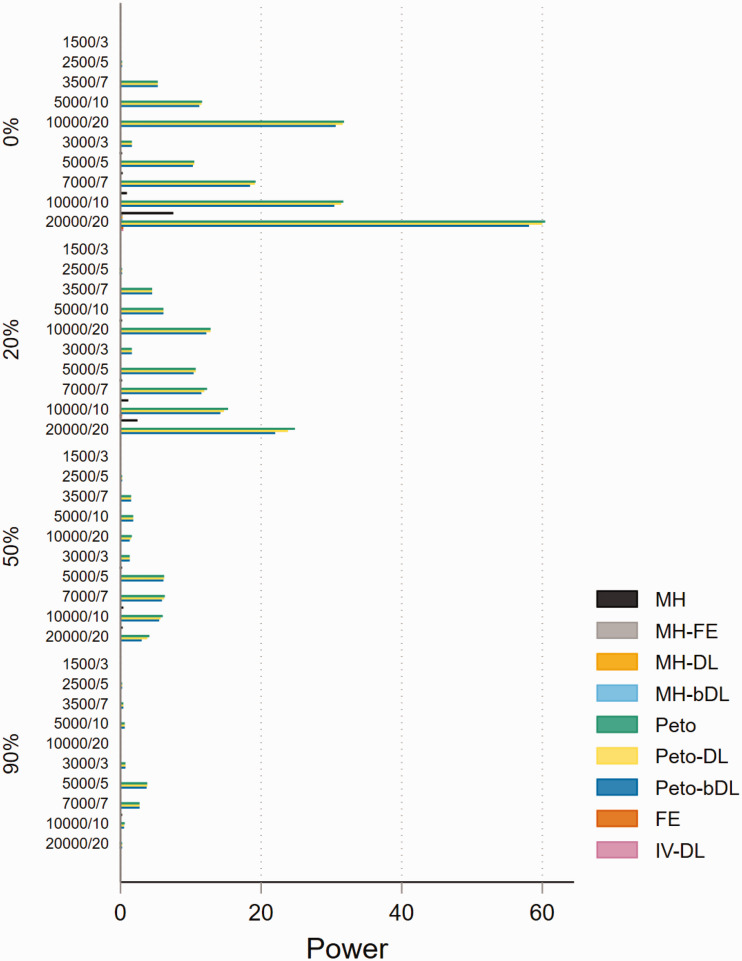
Power of very rare event scenarios with imbalanced treatment allocation (*r* = 0.1). IV: inverse variance; FE: fixed effect; RE: random effect; DL: DerSimonian and Laird; MH: Mantel–Haenszel; bDL: bootstrapped DL.

The performances based on power in MAs involving rare events show that all methods are able to maintain 80% power or above when minimal heterogeneity is present. This was particularly true in MAs with balanced treatment allocations (Figure 4). However, the performance was less consistent in MAs with lower sample sizes and imbalanced treatment allocation (online Figure S16). For example, when heterogeneity was above 20%, this resulted in a power below 30% across all methods. In the smaller sample size settings, the standard MH and Peto methods performed well. In MAs involving very rare events, the power was far less robust and was seen as insufficient. For example, in MAs involving imbalanced treatment allocations, the power to detect a true event failed to exceed 20% in most settings (Figure 5). In contrast, the performance in MAs involving balanced treatment allocations was moderately better when heterogeneity was below 20% (online Figure S17). The results for non-rare events are shown in online Figures S18 and S19.

### 5.5 Convergence

All 360 simulated MAs scenarios successfully converged across all methods (online Figures S20 to S25), and therefore, non-convergence was not an issue for this simulation study. Results for the 360 scenarios are provided in the online Appendix.

### 5.6 Making informed decisions about which methods to use in certain scenarios

In this section, we evaluate the methods used in this simulation study and discuss which are best suited for specific MAs settings. The preferred choice of the methods should always be based on the performances due to coverage and power combined. As the primary concern in MAs of safety should be to discern whether there is any signal of harm in the data, coverage and power are therefore of most importance. The results of coverage and power combined for rare, very rare and non-rare event settings are shown in online Figures S26 to S31.

The most optimal performing methods based on incidence and heterogeneity in MAs involving balance treatment allocations are presented in Table 3. For rare events, the Peto-bDL or Peto-DL methods performed best in MAs with lower sample size (≤3500 patients) settings and when small-to-moderate heterogeneity (0%–50%) were present. When higher values of heterogeneity were present, the MH-bDL method was preferred over Peto. This was especially true when the sample size was above 3500 patients. In MAs involving very rare events, the pattern was similar to that of rare events. However, Peto-bDL was the preferred method in higher sample size settings. For non-rare events, there was no obvious preferred method in the absence of heterogeneity. Otherwise, when heterogeneity was present, MH-DL or MH-bDL was preferred.

**Table 3.** Lookup table for optimal method(s) based on coverage and power for MAs involving balanced allocation ratio (r = 0.5).

		τ^2^			
		0%	20%	50%	90%
Sample size setting (patients/studies)	1500/3	VR=Peto	VR=Peto-bDL	VR=Peto-bDL	VR=Peto-bDL
		R=Peto	R=Peto-bDL	R=Peto-bDL	R=MH-bDL
		NR=Not obvious	NR=Peto-DL	NR=Peto-bDL	NR=MH-bDL
	2500/5	VR=Peto	VR=Peto-bDL	VR=Peto-bDL	VR=MH-bDL
		R=Peto	R=Peto-DL	R=Peto-bDL	R=MH-bDL
		NR=Not obvious	NR=MH-DL	NR=MH-DL	NR=MH-bDL
	3000/3	VR=Peto	VR=Peto-bDL	VR=Peto-bDL	VR=MH-bDL
		R=Peto	R=Peto-DL	R=Peto-bDL	R=MH-bDL
		NR=Not obvious	NR=Peto-DL	NR=Peto-bDL	NR=MH-bDL
	3500/7	VR=MH	VR=Peto-bDL	VR=Peto-bDL	VR=MH-bDL
		R=MH-bDL	R=Peto-bDL	R=Peto-bDL	R=MH-bDL
		NR=Not obvious	NR=MH-DL	NR=MH-DL	NR=MH-bDL
	5000/5	VR=Peto/MH	VR=Peto-DL	VR=Peto-bDL	VR=MH-bDL
		R=MH-bDL	R=MH-DL	R=MH-bDL	R=MH-bDL
		NR=Not obvious	NR=MH-DL	NR=MH-DL	NR=Peto-bDL
	5000/10	VR=MH	VR=Peto-bDL	VR=Peto-bDL	VR=MH-bDL
		R=MH-bDL	R=MH-DL	R=MH-bDL	R=MH-bDL
		NR=Not obvious	NR=MH-DL	NR=MH-DL	NR=Peto-bDL
	7000/7	VR=MH	VR=Peto-bDL	VR=Peto-bDL	VR=MH-bDL
		R=MH-bDL	R=MH-DL	R=MH-DL	R=MH-bDL
		NR=Not obvious	NR=MH-DL	NR=MH-DL	NR=Peto-bDL
	10000/10	VR=MH	VR=Peto-DL	VR=Peto-bDL	VR=MH-bDL
		R=MH-bDL	R=MH-DL	R=MH-DL	R=MH-bDL
		NR=Not obvious	NR=MH-DL	NR=MH-DL	NR=MH-bDL
	10000/20	VR=MH	VR=Peto-bDL	VR=Peto-bDL	VR=Peto-bDL
		R=Peto-bDL	R=MH-DL	R=MH-bDL	R=MH-bDL
		NR=Not obvious	NR=MH-DL	NR=MH-bDL	NR=MH-bDL
	20000/20	VR=MH	VR=Peto-bDL	VR=Peto-bDL	VR=MH-bDL
		R=MH-bDL	R=MH-bDL	R=MH-bDL	R=MH-bDL
		NR=Not obvious	NR=MH-DL	NR=MH-DL	NR=MH-bDL

VR: very rare; R: rare; NR: non-rare.

In MAs involving imbalanced treatment allocations (online Table S2), the trend of the performance was remarkably similar. However, the Peto methods performed well in rare event MAs with sample size settings of up to 5000 patients. In larger sample size settings above 5000 patients with higher levels of heterogeneity, MH-bDL was preferred. For very rare event MAs, Peto-bDL was clearly the most optimal method across all of the design features; and for non-rare event MAs, the Peto-bDL was preferred among smaller sample size settings (<3500 patients) and MH-bDL for larger samples (≥3500 patients). One notable and important observation was that none of the methods were able to achieve above 50% coverage and power whilst heterogeneity levels exceeded 50% in rare event MAs, and 20% in very rare event MAs.

## 6 Discussion

Our results show that some methods used for MAs of rare events can perform better than others under certain samples size settings, incidence and levels of heterogeneity. In MAs involving rare events with no heterogeneity, coverage and power revealed very small performance-based differences between the methods. In very rare event MAs displaying no heterogeneity, the Peto-bDL method performed best across all sample sizes. However, when heterogeneity was above 20%, convergence and power failed to exceed 50% performance – which only worsened as heterogeneity increased. There was a similar trend in MAs involving rare events, where the Peto-bDL was the preferred method, but this was only true in MAs with smaller sample sizes. In MAs involving medium-to-large sample sizes, MH-bDL generally outperformed the other methods.

The error associated with the methods measured by mean bias and mean error was almost identical across all methods in MAs of rare incidence. However, as the mean bias and mean error increased, this trend was closely associated with increased levels of heterogeneity. In general, the MH-bDL method was able to achieve the lowest bias and error in MAs of rare events. However, in MAs involving very rare events, the Peto-DL and Peto-bDL methods maintained better performances. A cautious approach is needed when MAs differ between balanced and imbalanced treatment allocations, where we have shown that using MH-DL and MH-bDL rather than Peto method is preferred in MAs with imbalanced treatment allocations.

### 6.1 Strengths and limitations

We have performed the largest simulation study on rare event meta-analyses to date including 360 realistic data sets with varied incidence rates, sample size settings, allocation of patients by treatment group and heterogeneity. We also include four newly proposed methods (MH-DL, MH-bDL, Peto-DL and Peto-bDL) which have not been used before in rare event MAs and are not specific to the Cochrane guidelines.

Despite these strengths, there remain several limitations. First, whilst our simulation study was restricted to the use of mostly the mainstream Cochrane recommended methods that are easily accessible and regularly used amongst the systematic review community. We are aware that improved performances have been shown in some of the more advanced statistical methods based on exact distributional assumptions.^18,21,30–32^ Such methods are designed on the principles of the inclusion of single zero or double zero events.^15,20^ But, there are several drawbacks to using these methods such as (i) they are not available in RevMan and therefore are not being used widespread among the Cochrane community, (ii) they are not included in any of the main guidelines for performing MAs,^8,49^ (iii) they rely upon authors reporting zero case events in their primary report, potentially precluding their inclusion in MAs in the first place and (iv) they require an understanding of statistical modelling based on distributions or Bayesian inference,^18^ which is another reason for their poor uptake, as the practitioner may not be statistically astute to such methods without adequate training.^7^ We also did not include some of the more recent methods that have only just be added to the Stata package ‘metan’,^50^ which include likelihood-based methods such as profile likelihood and the Bartlett and Skivgaard corrections to the likelihood. Both have been used in an earlier study,^51^ but showed little improvement in MAs involving common events.

Second, we only include methods which include the OR and did not consider other measures like relative risk or risk differences. Whilst OR is considered to have the best statistical properties in the case of the Peto OR, it is often misinterpreted as a relative risk, and authors might opt for the use of other effect measures that are easier to interpret.^18^

Finally, measurement errors can often complicate interpretation of the results by potentially concealing important differences between groups or by indicating differences, which, in reality, do not exist. The total measurement error is generally partitioned into two separate classes of error: systematic and random.^52^ Systematic errors (also known as ‘bias’) are reproducible inaccuracies that lead to a measured value that is consistently larger or smaller than the true value. Random errors lead to variable differences from the true value and give rise, unpredictably, to measurements that are greater or smaller than the true value. Random errors can be reduced by averaging over a number of observations and observing the Monte Carlo standard error. However, if the number of simulations is not large enough, it is likely that differences in point estimates (such as coverage and power) are due to random/simulation error.^37^ Nevertheless, we are confidence whilst averaging the performance measures over 1000 iterations that this is large enough to avoid the potential caveat of random error.

### 6.2 Implications for future practice

Not surprisingly, the random-effects model DL was the preferred method from our simulation analysis, as they are more general models as compared with their fixed-effect counterparts. As such, our work is a convenient and important extension of some of the most recent simulation studies for MAs with rare events.^1,10,14^ These earlier efforts mainly concentrated on the standard fixed-effect methods and were unable to include the more recent MH and Peto DL weighted schemes; and in particular, the non-parametric bootstrap extensions of DL which are not recommended in the guidelines. The bootstrapped DL had been seen to perform well overall despite its larger heterogeneity bias for small MAs^12,17^; however, its performance based on rare events remained relatively unknown until know. Here, we show that the bootstrap DL extensions for both Peto and MH generally outperform the other methods. This was particularly true based on the performances for coverage and power. Therefore, we stress the importance for further research to assess the wider use of these methods for when synthesising rare event data and recommend that any future updates of the guidelines should reflect these findings to encourage their uptake.

Over the last decade, there has been overwhelming support of methods which aim to include double zero studies without continuity correction by applying exact distributional assumptions instead of approximate likelihood. It has been shown that these methods can lead to reduced bias when such data are reported in the primary report. The most recent update of the Cochrane guidelines in 2019^53^ now give some credence to the existence of these methods. Whilst the methods do clearly hold some promise, they are still not being used widespread in the research community; as one study had recently shown.^7^ There are several reasons for this, firstly, they are still in their infancy stage of development, and therefore are not readily available in mainstream statistical software used for performing MAs. Secondly, is of course the fact that researchers are likely to take a rather dogmatic approach when zero events are present and simply apply a risk difference in a sensitivity analysis or apply some sort of continuity correction, or beyond they may just delete double zero studies from their data precluding their inclusion in a MAs. One thing that remains unclear is that when working with published results, whether the failure to mention a particular adverse event means there were no such events, or simply that such events were not included as a measured endpoint.^54^ Meta-analysts need not only clear and more precise guidance, but there should also be a policy requirement for reporting studies with no events by considering ‘joint reporting’ of clinical endpoints and safety events in clinical trials.^55,56^

A major fragility when performing MAs of rare events is that most of the included trials are not adequately powered to detect an effect on the event of interest such is the case for adverse events.^37,57^ This issue mostly arises because adverse events are often defined as secondary outcomes of interest in the study. Applied analysts need to think more critically about whether random-effects meta-analyses, when applied to highly heterogeneous datasets with very few studies or events, are likely to provide more power than individual studies. Power calculations are an important component of research grant proposals, but are rarely used in practice.^58–60^ There are several software options available for performing simple and quick power calculations. For example, in Stata, there is the ‘power’ command which enables robust calculations including power estimation for cluster randomised controlled trials.^61^ There is also a similar command in R (‘clusterPower’) which allows for exactly the same calculation.^62^ Recent supporting evidence for power calculations suggests that at least five or more studies are needed to reasonably consistently achieve powers from random-effects MAs.^57^ But, because this was based on MAs of common events, the statistical inferences in our study which are drawn from MAs with very few studies and/or events means that MAs are likely to be considered even less worthwhile. Further research is desperately needed into power assumptions when the data are sparse.

### 6.3 Conclusions

To conclude in MAs of rare binary outcomes, we have shown that the Peto-bDL or Peto-DL was most effective in both rare and very rare event settings, with the exception of MAs involving medium-to-large sample sizes where MH-bDL is preferred. In cases where heterogeneity is large, performance estimation based on coverage and power was mostly insufficient. Here, we advise analysts to think more critically about their MAs approach, when applied to highly heterogeneous datasets with very few events, and we strongly encourage the use of power calculations before considering a MAs. Whilst this simulation study has clearly shown that some of the methods that are used less often in MAs do appear to have good properties under sparse data scenarios, we urge the need for further work to assess the methods alongside more complex distributional-based methods in future simulation or empirical studies.

## Supplemental Material

sj-pdf-1-smm-10.1177_09622802211022385 - Supplemental material for Applications of simple and accessible methods for meta-analysis involving rare events: A simulation study Click here for additional data file. Supplemental material, sj-pdf-1-smm-10.1177_09622802211022385 for Applications of simple and accessible methods for meta-analysis involving rare events: A simulation study by Alexander Hodkinson and Evangelos Kontopantelis in Statistical Methods in Medical Research

sj-pdf-2-smm-10.1177_09622802211022385 - Supplemental material for Applications of simple and accessible methods for meta-analysis involving rare events: A simulation study Click here for additional data file. Supplemental material, sj-pdf-2-smm-10.1177_09622802211022385 for Applications of simple and accessible methods for meta-analysis involving rare events: A simulation study by Alexander Hodkinson and Evangelos Kontopantelis in Statistical Methods in Medical Research

sj-pdf-3-smm-10.1177_09622802211022385 - Supplemental material for Applications of simple and accessible methods for meta-analysis involving rare events: A simulation study Click here for additional data file. Supplemental material, sj-pdf-3-smm-10.1177_09622802211022385 for Applications of simple and accessible methods for meta-analysis involving rare events: A simulation study by Alexander Hodkinson and Evangelos Kontopantelis in Statistical Methods in Medical Research

sj-pdf-4-smm-10.1177_09622802211022385 - Supplemental material for Applications of simple and accessible methods for meta-analysis involving rare events: A simulation study Click here for additional data file. Supplemental material, sj-pdf-4-smm-10.1177_09622802211022385 for Applications of simple and accessible methods for meta-analysis involving rare events: A simulation study by Alexander Hodkinson and Evangelos Kontopantelis in Statistical Methods in Medical Research
