# Supplementary material for: Applications of simple and accessible methods for meta-analysis involving rare events: A simulation study
Source: Stat Methods Med Res. 2021 Jun 17;30(7):1589–608. doi: 10.1177/09622802211022385 (PMC8411477; doi:10.1177/09622802211022385)
Supplement: sj-pdf-2-smm-10.1177_09622802211022385 - Supplemental material for Applications of simple and accessible methods for meta-analysis involving rare events: A simulation study [file sj-pdf-2-smm-10.1177_09622802211022385.pdf]

Rare events scenarios: 10% vs 5.2632%, OR=0.5

Convergence (successful MA %)

	ssl	ssh	tsq1	cb	MH	MHfe	MHdl	MHbdl	P	Pdl	Pbdl	Pbdl	Pbdl
	1500	3	0	.5	100	100	100	100	100	100	100	100	100
	2500	5	0	.5	100	100	100	100	100	100	100	100	100
	3500	7	0	.5	100	100	100	100	100	100	100	100	100
	5000	10	0	.5	100	100	100	100	100	100	100	100	100
	10000	20	0	.5	100	100	100	100	100	100	100	100	100
	3000	3	0	.5	100	100	100	100	100	100	100	100	100
	5000	5	0	.5	100	100	100	100	100	100	100	100	100
	7000	7	0	.5	100	100	100	100	100	100	100	100	100
	10000	10	0	.5	100	100	100	100	100	100	100	100	100
	20000	20	0	.5	100	100	100	100	100	100	100	100	100
	7500	3	0	.5	100	100	100	100	100	100	100	100	100
	12500	5	0	.5	100	100	100	100	100	100	100	100	100
	17500	7	0	.5	100	100	100	100	100	100	100	100	100
	25000	10	0	.5	100	100	100	100	100	100	100	100	100
	50000	20	0	.5	100	100	100	100	100	100	100	100	100
	1500	3	0	.1	100	100	100	100	100	100	100	100	100
	2500	5	0	.1	100	100	100	100	100	100	100	100	100
	3500	7	0	.1	100	100	100	100	100	100	100	100	100
	5000	10	0	.1	100	100	100	100	100	100	100	100	100
	10000	20	0	.1	100	100	100	100	100	100	100	100	100
	3000	3	0	.1	100	100	100	100	100	100	100	100	100
	5000	5	0	.1	100	100	100	100	100	100	100	100	100
	7000	7	0	.1	100	100	100	100	100	100	100	100	100
	10000	10	0	.1	100	100	100	100	100	100	100	100	100
	20000	20	0	.1	100	100	100	100	100	100	100	100	100
	7500	3	0	.1	100	100	100	100	100	100	100	100	100
	12500	5	0	.1	100	100	100	100	100	100	100	100	100
	17500	7	0	.1	100	100	100	100	100	100	100	100	100
	25000	10	0	.1	100	100	100	100	100	100	100	100	100
	50000	20	0	.1	100	100	100	100	100	100	100	100	100
	1500	3	.822467	.5	100	100	100	100	100	100	100	100	100
	2500	5	.822467	.5	100	100	100	100	100	100	100	100	100
	3500	7	.822467	.5	100	100	100	100	100	100	100	100	100
	5000	10	.822467	.5	100	100	100	100	100	100	100	100	100
	10000	20	.822467	.5	100	100	100	100	100	100	100	100	100
	3000	3	.822467	.5	100	100	100	100	100	100	100	100	100
	5000	5	.822467	.5	100	100	100	100	100	100	100	100	100
	7000	7	.822467	.5	100	100	100	100	100	100	100	100	100
	10000	10	.822467	.5	100	100	100	100	100	100	100	100	100

Create PDF in your applications with the Pdfcrowd HTML to PDF API PDFCROWD

20000	20	.822467	.5	100	100	100	100	100	100	100	100	100
7500	3	.822467	.5	100	100	100	100	100	100	100	100	100
12500	5	.822467	.5	100	100	100	100	100	100	100	100	100
17500	7	.822467	.5	100	100	100	100	100	100	100	100	100
25000	10	.822467	.5	100	100	100	100	100	100	100	100	100
50000	20	.822467	.5	100	100	100	100	100	100	100	100	100
1500	3	.822467	.1	100	100	100	100	100	100	100	100	100
2500	5	.822467	.1	100	100	100	100	100	100	100	100	100
3500	7	.822467	.1	100	100	100	100	100	100	100	100	100
5000	10	.822467	.1	100	100	100	100	100	100	100	100	100
10000	20	.822467	.1	100	100	100	100	100	100	100	100	100
3000	3	.822467	.1	100	100	100	100	100	100	100	100	100
5000	5	.822467	.1	100	100	100	100	100	100	100	100	100
7000	7	.822467	.1	100	100	100	100	100	100	100	100	100
10000	10	.822467	.1	100	100	100	100	100	100	100	100	100
20000	20	.822467	.1	100	100	100	100	100	100	100	100	100
7500	3	.822467	.1	100	100	100	100	100	100	100	100	100
12500	5	.822467	.1	100	100	100	100	100	100	100	100	100
17500	7	.822467	.1	100	100	100	100	100	100	100	100	100
25000	10	.822467	.1	100	100	100	100	100	100	100	100	100
50000	20	.822467	.1	100	100	100	100	100	100	100	100	100
1500	3	3.289868	.5	100	100	100	100	100	100	100	100	100
2500	5	3.289868	.5	100	100	100	100	100	100	100	100	100
3500	7	3.289868	.5	100	100	100	100	100	100	100	100	100
5000	10	3.289868	.5	100	100	100	100	100	100	100	100	100
10000	20	3.289868	.5	100	100	100	100	100	100	100	100	100
3000	3	3.289868	.5	100	100	100	100	100	100	100	100	100
5000	5	3.289868	.5	100	100	100	100	100	100	100	100	100
7000	7	3.289868	.5	100	100	100	100	100	100	100	100	100
10000	10	3.289868	.5	100	100	100	100	100	100	100	100	100
20000	20	3.289868	.5	100	100	100	100	100	100	100	100	100
7500	3	3.289868	.5	100	100	100	100	100	100	100	100	100
12500	5	3.289868	.5	100	100	100	100	100	100	100	100	100
17500	7	3.289868	.5	100	100	100	100	100	100	100	100	100
25000	10	3.289868	.5	100	100	100	100	100	100	100	100	100
50000	20	3.289868	.5	100	100	100	100	100	100	100	100	100
1500	3	3.289868	.1	100	100	100	100	100	100	100	100	100
2500	5	3.289868	.1	100	100	100	100	100	100	100	100	100
3500	7	3.289868	.1	100	100	100	100	100	100	100	100	100
5000	10	3.289868	.1	100	100	100	100	100	100	100	100	100
10000	20	3.289868	.1	100	100	100	100	100	100	100	100	100
3000	3	3.289868	.1	100	100	100	100	100	100	100	100	100
5000	5	3.289868	.1	100	100	100	100	100	100	100	100	100
7000	7	3.289868	.1	100	100	100	100	100	100	100	100	100
10000	10	3.289868	.1	100	100	100	100	100	100	100	100	100
20000	20	3.289868	.1	100	100	100	100	100	100	100	100	100
7500	3	3.289868	.1	100	100	100	100	100	100	100	100	100
12500	5	3.289868	.1	100	100	100	100	100	100	100	100	100
17500	7	3.289868	.1	100	100	100	100	100	100	100	100	100
25000	10	3.289868	.1	100	100	100	100	100	100	100	100	100
50000	20	3.289868	.1	100	100	100	100	100	100	100	100	100
1500	3	29.60881	.5	100	100	100	100	100	100	100	100	100
2500	5	29.60881	.5	100	100	100	100	100	100	100	100	100

Create PDF in your applications with the Pdfcrowd HTML to PDF API PDFCROWD

3500	7	29.60881	.5	100	100	100	100	100	100	100	100	100
5000	10	29.60881	.5	100	100	100	100	100	100	100	100	100
10000	20	29.60881	.5	100	100	100	100	100	100	100	100	100
3000	3	29.60881	.5	100	100	100	100	100	100	100	100	100
5000	5	29.60881	.5	100	100	100	100	100	100	100	100	100
7000	7	29.60881	.5	100	100	100	100	100	100	100	100	100
10000	10	29.60881	.5	100	100	100	100	100	100	100	100	100
20000	20	29.60881	.5	100	100	100	100	100	100	100	100	100
7500	3	29.60881	.5	100	100	100	100	100	100	100	100	100
12500	5	29.60881	.5	100	100	100	100	100	100	100	100	100
17500	7	29.60881	.5	100	100	100	100	100	100	100	100	100
25000	10	29.60881	.5	100	100	100	100	100	100	100	100	100
50000	20	29.60881	.5	100	100	100	100	100	100	100	100	100
1500	3	29.60881	.1	100	100	100	100	100	100	100	100	100
2500	5	29.60881	.1	100	100	100	100	100	100	100	100	100
3500	7	29.60881	.1	100	100	100	100	100	100	100	100	100
5000	10	29.60881	.1	100	100	100	100	100	100	100	100	100
10000	20	29.60881	.1	100	100	100	100	100	100	100	100	100
3000	3	29.60881	.1	100	100	100	100	100	100	100	100	100
5000	5	29.60881	.1	100	100	100	100	100	100	100	100	100
7000	7	29.60881	.1	100	100	100	100	100	100	100	100	100
10000	10	29.60881	.1	100	100	100	100	100	100	100	100	100
20000	20	29.60881	.1	100	100	100	100	100	100	100	100	100
7500	3	29.60881	.1	100	100	100	100	100	100	100	100	100
12500	5	29.60881	.1	100	100	100	100	100	100	100	100	100
17500	7	29.60881	.1	100	100	100	100	100	100	100	100	100
25000	10	29.60881	.1	100	100	100	100	100	100	100	100	100
50000	20	29.60881	.1	100	100	100	100	100	100	100	100	100

SE of convergence

ssl	ssh	tsq1	cb	MH	MHfe	MHdl	MHbdl	P	Pdl	Pbdl	Pbdl	Pbdl
1500	3	0	.5	0	0	0	0	0	0	0	0	0
2500	5	0	.5	0	0	0	0	0	0	0	0	0
3500	7	0	.5	0	0	0	0	0	0	0	0	0
5000	10	0	.5	0	0	0	0	0	0	0	0	0
10000	20	0	.5	0	0	0	0	0	0	0	0	0
3000	3	0	.5	0	0	0	0	0	0	0	0	0
5000	5	0	.5	0	0	0	0	0	0	0	0	0
7000	7	0	.5	0	0	0	0	0	0	0	0	0
10000	10	0	.5	0	0	0	0	0	0	0	0	0
20000	20	0	.5	0	0	0	0	0	0	0	0	0
7500	3	0	.5	0	0	0	0	0	0	0	0	0
12500	5	0	.5	0	0	0	0	0	0	0	0	0
17500	7	0	.5	0	0	0	0	0	0	0	0	0
25000	10	0	.5	0	0	0	0	0	0	0	0	0
50000	20	0	.5	0	0	0	0	0	0	0	0	0

Create PDF in your applications with the Pdfcrowd HTML to PDF API PDFCROWD

1500	3	0	.1	0	0	0	0	0	0	0	0	0
2500	5	0	.1	0	0	0	0	0	0	0	0	0
3500	7	0	.1	0	0	0	0	0	0	0	0	0
5000	10	0	.1	0	0	0	0	0	0	0	0	0
10000	20	0	.1	0	0	0	0	0	0	0	0	0
3000	3	0	.1	0	0	0	0	0	0	0	0	0
5000	5	0	.1	0	0	0	0	0	0	0	0	0
7000	7	0	.1	0	0	0	0	0	0	0	0	0
10000	10	0	.1	0	0	0	0	0	0	0	0	0
20000	20	0	.1	0	0	0	0	0	0	0	0	0
7500	3	0	.1	0	0	0	0	0	0	0	0	0
12500	5	0	.1	0	0	0	0	0	0	0	0	0
17500	7	0	.1	0	0	0	0	0	0	0	0	0
25000	10	0	.1	0	0	0	0	0	0	0	0	0
50000	20	0	.1	0	0	0	0	0	0	0	0	0
1500	3	.822467	.5	0	0	0	0	0	0	0	0	0
2500	5	.822467	.5	0	0	0	0	0	0	0	0	0
3500	7	.822467	.5	0	0	0	0	0	0	0	0	0
5000	10	.822467	.5	0	0	0	0	0	0	0	0	0
10000	20	.822467	.5	0	0	0	0	0	0	0	0	0
3000	3	.822467	.5	0	0	0	0	0	0	0	0	0
5000	5	.822467	.5	0	0	0	0	0	0	0	0	0
7000	7	.822467	.5	0	0	0	0	0	0	0	0	0
10000	10	.822467	.5	0	0	0	0	0	0	0	0	0
20000	20	.822467	.5	0	0	0	0	0	0	0	0	0
7500	3	.822467	.5	0	0	0	0	0	0	0	0	0
12500	5	.822467	.5	0	0	0	0	0	0	0	0	0
17500	7	.822467	.5	0	0	0	0	0	0	0	0	0
25000	10	.822467	.5	0	0	0	0	0	0	0	0	0
50000	20	.822467	.5	0	0	0	0	0	0	0	0	0
1500	3	.822467	.1	0	0	0	0	0	0	0	0	0
2500	5	.822467	.1	0	0	0	0	0	0	0	0	0
3500	7	.822467	.1	0	0	0	0	0	0	0	0	0
5000	10	.822467	.1	0	0	0	0	0	0	0	0	0
10000	20	.822467	.1	0	0	0	0	0	0	0	0	0
3000	3	.822467	.1	0	0	0	0	0	0	0	0	0
5000	5	.822467	.1	0	0	0	0	0	0	0	0	0
7000	7	.822467	.1	0	0	0	0	0	0	0	0	0
10000	10	.822467	.1	0	0	0	0	0	0	0	0	0
20000	20	.822467	.1	0	0	0	0	0	0	0	0	0
7500	3	.822467	.1	0	0	0	0	0	0	0	0	0
12500	5	.822467	.1	0	0	0	0	0	0	0	0	0
17500	7	.822467	.1	0	0	0	0	0	0	0	0	0
25000	10	.822467	.1	0	0	0	0	0	0	0	0	0
50000	20	.822467	.1	0	0	0	0	0	0	0	0	0
1500	3	3.289868	.5	0	0	0	0	0	0	0	0	0
2500	5	3.289868	.5	0	0	0	0	0	0	0	0	0
3500	7	3.289868	.5	0	0	0	0	0	0	0	0	0
5000	10	3.289868	.5	0	0	0	0	0	0	0	0	0
10000	20	3.289868	.5	0	0	0	0	0	0	0	0	0
3000	3	3.289868	.5	0	0	0	0	0	0	0	0	0
5000	5	3.289868	.5	0	0	0	0	0	0	0	0	0
7000	7	3.289868	.5	0	0	0	0	0	0	0	0	0

Create PDF in your applications with the Pdfcrowd HTML to PDF API PDFCROWD

10000	10	3.289868	.5	0	0	0	0	0	0	0	0	0
20000	20	3.289868	.5	0	0	0	0	0	0	0	0	0
7500	3	3.289868	.5	0	0	0	0	0	0	0	0	0
12500	5	3.289868	.5	0	0	0	0	0	0	0	0	0
17500	7	3.289868	.5	0	0	0	0	0	0	0	0	0
25000	10	3.289868	.5	0	0	0	0	0	0	0	0	0
50000	20	3.289868	.5	0	0	0	0	0	0	0	0	0
1500	3	3.289868	.1	0	0	0	0	0	0	0	0	0
2500	5	3.289868	.1	0	0	0	0	0	0	0	0	0
3500	7	3.289868	.1	0	0	0	0	0	0	0	0	0
5000	10	3.289868	.1	0	0	0	0	0	0	0	0	0
10000	20	3.289868	.1	0	0	0	0	0	0	0	0	0
3000	3	3.289868	.1	0	0	0	0	0	0	0	0	0
5000	5	3.289868	.1	0	0	0	0	0	0	0	0	0
7000	7	3.289868	.1	0	0	0	0	0	0	0	0	0
10000	10	3.289868	.1	0	0	0	0	0	0	0	0	0
20000	20	3.289868	.1	0	0	0	0	0	0	0	0	0
7500	3	3.289868	.1	0	0	0	0	0	0	0	0	0
12500	5	3.289868	.1	0	0	0	0	0	0	0	0	0
17500	7	3.289868	.1	0	0	0	0	0	0	0	0	0
25000	10	3.289868	.1	0	0	0	0	0	0	0	0	0
50000	20	3.289868	.1	0	0	0	0	0	0	0	0	0
1500	3	29.60881	.5	0	0	0	0	0	0	0	0	0
2500	5	29.60881	.5	0	0	0	0	0	0	0	0	0
3500	7	29.60881	.5	0	0	0	0	0	0	0	0	0
5000	10	29.60881	.5	0	0	0	0	0	0	0	0	0
10000	20	29.60881	.5	0	0	0	0	0	0	0	0	0
3000	3	29.60881	.5	0	0	0	0	0	0	0	0	0
5000	5	29.60881	.5	0	0	0	0	0	0	0	0	0
7000	7	29.60881	.5	0	0	0	0	0	0	0	0	0
10000	10	29.60881	.5	0	0	0	0	0	0	0	0	0
20000	20	29.60881	.5	0	0	0	0	0	0	0	0	0
7500	3	29.60881	.5	0	0	0	0	0	0	0	0	0
12500	5	29.60881	.5	0	0	0	0	0	0	0	0	0
17500	7	29.60881	.5	0	0	0	0	0	0	0	0	0
25000	10	29.60881	.5	0	0	0	0	0	0	0	0	0
50000	20	29.60881	.5	0	0	0	0	0	0	0	0	0
1500	3	29.60881	.1	0	0	0	0	0	0	0	0	0
2500	5	29.60881	.1	0	0	0	0	0	0	0	0	0
3500	7	29.60881	.1	0	0	0	0	0	0	0	0	0
5000	10	29.60881	.1	0	0	0	0	0	0	0	0	0
10000	20	29.60881	.1	0	0	0	0	0	0	0	0	0
3000	3	29.60881	.1	0	0	0	0	0	0	0	0	0
5000	5	29.60881	.1	0	0	0	0	0	0	0	0	0
7000	7	29.60881	.1	0	0	0	0	0	0	0	0	0
10000	10	29.60881	.1	0	0	0	0	0	0	0	0	0
20000	20	29.60881	.1	0	0	0	0	0	0	0	0	0
7500	3	29.60881	.1	0	0	0	0	0	0	0	0	0
12500	5	29.60881	.1	0	0	0	0	0	0	0	0	0
17500	7	29.60881	.1	0	0	0	0	0	0	0	0	0
25000	10	29.60881	.1	0	0	0	0	0	0	0	0	0
50000	20	29.60881	.1	0	0	0	0	0	0	0	0	0

Create PDF in your applications with the Pdfcrowd HTML to PDF API PDFCROWD

Coverage

ssl	ssh	tsq1	cb	MH	MHfe	MHdl	MHbdl	P	Pdl	Pbdl	Pbdl	Pbdl
1500	3	0	.5	98.4	99.1	99.2	99.2	96.3	96.8	96.9	98.9	99
2500	5	0	.5	97.7	97.6	97.8	98	95.9	96.3	96.6	97.8	98.1
3500	7	0	.5	97	97.4	97.5	97.8	96	96.5	97.1	97.9	98.2
5000	10	0	.5	97.3	97.4	97.5	97.6	97.1	97.2	97.5	97.6	97.6
10000	20	0	.5	96.8	93.8	93.8	94	95.6	95.8	96	96	96
3000	3	0	.5	96.4	97.6	98.5	98.7	96.3	97.2	97.4	97.5	98.4
5000	5	0	.5	95.8	96.8	97.4	97.6	96	97.3	97.4	97	97.6
7000	7	0	.5	95.5	96.2	96.7	97.4	95.5	96.3	97.3	96.6	97.2
10000	10	0	.5	95.7	96.6	96.9	97.4	95.8	96.7	97	96.7	97.1
20000	20	0	.5	95.6	94	94.1	94.6	95.4	96.1	96.4	95.3	95.3
7500	3	0	.5	95.2	96	97	97.7	95.8	96.9	97.5	95.9	97
12500	5	0	.5	95.2	95.4	96.6	97.1	95.8	96.7	97.3	95.4	96.6
17500	7	0	.5	95	95.7	96.3	96.9	96.5	97.1	97.3	95.6	96.2
25000	10	0	.5	94.1	95.7	96.1	96.5	95.3	96.1	96.5	95.6	96.1
50000	20	0	.5	94.5	94.6	95.3	95.7	95.1	95.9	96.2	94.5	95.3
1500	3	0	.1	92.3	89.6	90.3	90.9	97.5	98.7	99	89.8	90.9
2500	5	0	.1	85.1	77.3	77.7	78	96.2	98.3	98.9	77.9	78.5
3500	7	0	.1	81	63.6	63.6	64	96.4	98.5	98.7	65.6	65.6
5000	10	0	.1	71.6	39.2	39.2	39.3	98.3	99	99.2	44.5	44.6
10000	20	0	.1	40.8	1.4	1.4	1.4	97.1	97.4	97.7	4	4
3000	3	0	.1	94.2	92	93.3	93.9	97.1	98.2	98.3	92	93.5
5000	5	0	.1	93.2	83.9	85.6	86.4	98.1	99	99.1	84.2	86
7000	7	0	.1	90.7	79.5	79.8	80.6	96.6	97.6	97.6	80	80.3
10000	10	0	.1	86.7	69.2	69.5	70.7	96.8	97.6	98.2	70.4	70.7
20000	20	0	.1	72	33.1	33.1	33.3	94	94.5	95.1	37.4	37.4
7500	3	0	.1	97	94.9	96.4	97	98	98.5	98.5	94.9	96.4
12500	5	0	.1	96.3	90.4	92.9	94.4	97.2	97.9	98.2	90.6	93.1
17500	7	0	.1	95.3	86.5	89.2	90.5	95.1	95.9	96.7	86.7	89.5
25000	10	0	.1	94.8	82.1	83.4	84.8	93.8	94.3	94.8	82.6	83.8
50000	20	0	.1	91.9	63.7	64	66	86.5	86.6	87.4	64.7	65.1
1500	3	.822467	.5	85	86.1	90.5	92.2	80.6	88.3	90.1	86.3	91.3
2500	5	.822467	.5	81	80.9	88.1	89.6	78.5	88.3	90.1	82.2	89.3
3500	7	.822467	.5	75.6	73.9	83.3	85.3	72.4	87.6	89.2	76.3	86.5
5000	10	.822467	.5	71.7	67.5	76.1	78.7	68.5	85.7	86.9	70.6	81.1
10000	20	.822467	.5	59.9	45.2	53.8	56.8	55.4	79.7	80.4	51.2	65
3000	3	.822467	.5	75	75.9	88.7	90.3	73.6	87.5	89.5	76	89.1
5000	5	.822467	.5	70.5	70	87.4	89.2	70.2	87.1	88.8	70.4	88.1
7000	7	.822467	.5	63	60.9	83.1	85.7	62.3	86.1	87.6	61.6	84.6
10000	10	.822467	.5	55.4	50.8	80	81.9	54.8	85.9	86.5	51.7	81.1
20000	20	.822467	.5	44.2	36	69.2	70.6	42.9	83.8	83.8	37.4	74
7500	3	.822467	.5	56	56.1	81.5	84.8	57	80	82.6	56	81.5
12500	5	.822467	.5	50.5	50.4	84.8	87.2	51.4	84.6	86.6	50.5	85
17500	7	.822467	.5	49.3	46.8	87.1	88.2	50	88.1	89.3	47	87.1
25000	10	.822467	.5	41.2	34.4	85.9	87.4	40	87	87.5	34.5	86.1

Create PDF in your applications with the Pdfcrowd HTML to PDF API PDFCROWD

50000	20	.822467	.5	26.2	17	83.6	83.9	25.2	86.9	86.8	17	84.1
1500	3	.822467	.1	85	79.9	82	83.1	92.5	97.1	97.3	79.9	82.7
2500	5	.822467	.1	70.9	59.2	60.4	61.6	87.5	96	96.6	59.9	61.6
3500	7	.822467	.1	65	45.2	45.3	46.1	87.8	95.4	96	47.4	47.5
5000	10	.822467	.1	49.6	22.3	22.3	22.7	84	93.3	93.8	26.1	26.1
10000	20	.822467	.1	18.7	.5	.5	.5	74.4	82.2	83.6	1.7	1.7
3000	3	.822467	.1	78.7	72.1	79	82	85.7	95	96	72.1	79.5
5000	5	.822467	.1	76.4	64.5	68.5	70.6	83.6	94.1	94.7	64.7	69.6
7000	7	.822467	.1	67.2	51.5	55.5	56.7	80.5	92.2	93.5	52	56.5
10000	10	.822467	.1	56.7	35.3	36.7	37.4	75.7	91	92.4	36.4	38.5
20000	20	.822467	.1	28.4	6.8	7.4	7.4	60.5	73.8	75.8	7.8	8.6
7500	3	.822467	.1	73.4	66.8	82.8	85.8	76.3	91.8	92.5	66.8	83
12500	5	.822467	.1	69.1	55.5	76.7	81	70	90.8	91.5	55.7	77.1
17500	7	.822467	.1	60.2	42.9	67	70.3	61.8	85.5	86.6	42.9	67.9
25000	10	.822467	.1	56	34.7	57.6	59.4	57	80.6	82.7	34.8	58.8
50000	20	.822467	.1	36.4	9.9	28.1	28.7	33.8	56.3	56.6	10	29.2
1500	3	3.289868	.5	61.1	64.2	85.5	88	58.3	83.1	86.7	64.4	86.5
2500	5	3.289868	.5	44.3	47.4	77.5	80.5	41.2	78.1	81.8	48.3	79.9
3500	7	3.289868	.5	33.8	38.4	72.6	75.3	32.5	77.5	79.7	39.8	76.3
5000	10	3.289868	.5	25.2	26.1	66.5	68.5	24	74.6	76.9	28.3	73.1
10000	20	3.289868	.5	7.9	6.6	42.9	43.5	6.8	63.1	63.4	8.9	52.4
3000	3	3.289868	.5	46.7	49.9	82.6	84.8	45	78.3	82.1	49.7	82.5
5000	5	3.289868	.5	36.4	38.7	82.7	85.5	35.8	81.3	84.6	38.5	82.6
7000	7	3.289868	.5	25.9	27.2	80	82.3	25.3	82.2	84	27.6	81.4
10000	10	3.289868	.5	17.4	15.8	75.3	77.2	17.2	79.8	81.5	16.6	76.9
20000	20	3.289868	.5	6.4	4	59.8	59.7	6	69.3	70.3	4.4	62.3
7500	3	3.289868	.5	30.2	33.8	78.6	82.3	31.9	75	78.6	33.5	78.5
12500	5	3.289868	.5	21.4	24	80.7	83.9	22.2	79.4	81.7	23.9	80.6
17500	7	3.289868	.5	17.7	16.7	84.2	86.2	18	82.4	85	16.8	84.4
25000	10	3.289868	.5	10.6	8.7	82.8	83.8	10.7	83.3	85	8.7	83
50000	20	3.289868	.5	2.7	1.3	76	76.5	2.7	77.1	78	1.4	76.4
1500	3	3.289868	.1	59.8	54.8	64.4	65.7	68	92.2	94	54.8	66.4
2500	5	3.289868	.1	46.6	38.4	45	45.7	59.9	89.5	90.5	39.1	46.7
3500	7	3.289868	.1	35	23.7	27.4	27.9	53.1	89.4	90.3	24.8	29.6
5000	10	3.289868	.1	19.6	7.3	9.7	9	39.6	83.5	83.5	8.1	11.3
10000	20	3.289868	.1	1.9	.1	.1	.1	16.7	64.9	65.3	.1	.1
3000	3	3.289868	.1	54.8	50.7	71.3	75.7	59.2	90.4	91.5	50.7	72.1
5000	5	3.289868	.1	43	33.6	54.2	57	51.8	88.9	89.8	34	55.9
7000	7	3.289868	.1	30.5	22	42.6	43.7	39.8	87	87.7	22.2	44.2
10000	10	3.289868	.1	19.1	11.3	25.8	26.5	29.6	81.6	81.8	11.8	27.7
20000	20	3.289868	.1	4.4	.7	4.6	4.1	10.1	59.2	58.5	.8	5.6
7500	3	3.289868	.1	52.7	45.4	81.3	85.8	54.6	92	93.2	45.4	81.4
12500	5	3.289868	.1	35.7	26.5	68.7	75.1	38.2	88.2	89.1	26.5	69
17500	7	3.289868	.1	27.3	16.8	61.6	67.9	28.8	83.5	83.5	17	62.1
25000	10	3.289868	.1	19.6	9.3	54.3	58.3	21.3	78.3	78.4	9.4	55.2
50000	20	3.289868	.1	3.7	.8	35.8	36.6	3.8	57.5	56.8	.8	36.2
1500	3	29.60881	.5	26.7	30.4	79.9	83.4	24	69.3	80	30.1	80.1
2500	5	29.60881	.5	11.8	16.6	73.5	76.7	10.3	60.4	74	16.6	75.3
3500	7	29.60881	.5	6.3	9.9	62.7	66.4	5.6	50.1	62.8	10.2	65.9
5000	10	29.60881	.5	1.8	3.1	50.5	52.7	1.6	40.4	50	3.4	53.7
10000	20	29.60881	.5	.1	.2	21.8	22	.2	19.2	24.4	.2	24.8
3000	3	29.60881	.5	18.4	23	74.6	81	15.3	62.2	72.7	22.3	74
5000	5	29.60881	.5	7.9	11.6	72.2	77.6	7.8	60.6	73.3	11.1	72

Create PDF in your applications with the Pdfcrowd HTML to PDF API PDFCROWD

7000	7	29.60881	.5	3.8	5.5	68.2	71.3	3.5	54.6	65.7	5.5	68.1
10000	10	29.60881	.5	.9	2	57.4	60.7	.8	45	54.1	2	58.1
20000	20	29.60881	.5	.1	.1	32.7	33.8	.1	24.1	29.9	.1	33.5
7500	3	29.60881	.5	9.6	13.5	72	78.9	10	63.6	73.8	13	71.5
12500	5	29.60881	.5	4.9	5.4	72.4	78.1	4.9	62.6	73.7	5.5	72.2
17500	7	29.60881	.5	1.9	3.4	72.2	76.6	2.1	59.4	70.7	3.4	72.2
25000	10	29.60881	.5	.4	1.3	64.4	68.5	.4	53.1	61.9	1.3	64.5
50000	20	29.60881	.5	0	0	44.9	47.1	0	32.5	37	0	45.1
1500	3	29.60881	.1	26.9	25.7	62.6	66	29.4	77.2	81	25.7	63.5
2500	5	29.60881	.1	12.9	11.7	38.6	40.6	16.7	71.4	77.1	11.7	40.1
3500	7	29.60881	.1	5.1	4	22.7	23.7	7.8	60.3	65.4	4.1	24.4
5000	10	29.60881	.1	1.9	1.2	7.6	6.6	3.3	48.6	51.3	1.3	9.2
10000	20	29.60881	.1	0	0	.1	0	.1	18.4	19.3	0	.2
3000	3	29.60881	.1	24.9	24.1	67.1	72.6	26.1	79	83.9	24.1	67.4
5000	5	29.60881	.1	11.5	10.7	55.5	61.9	13.4	73.6	79.5	10.8	56.3
7000	7	29.60881	.1	6	4.9	41.9	45.3	7.9	63.3	66.9	4.9	43.2
10000	10	29.60881	.1	1.5	1.1	25.2	27.3	2.4	51.2	54.9	1.2	27.1
20000	20	29.60881	.1	.1	0	2.6	2.6	.1	19.8	20.6	0	2.9
7500	3	29.60881	.1	26.6	24.4	75.1	83.1	27.5	80.1	82.5	24.4	75.2
12500	5	29.60881	.1	12.4	10	62.3	72.6	13	71.7	77.1	10	62.3
17500	7	29.60881	.1	4.9	2.9	51.8	61.3	5.4	66.1	69.9	2.9	51.8
25000	10	29.60881	.1	1.2	.8	37.4	43.1	1.5	51.7	55.1	.8	37.4
50000	20	29.60881	.1	.1	0	10.9	13.1	.1	19.8	20.2	0	11

SE of coverage

ssl	ssh	tsq1	cb	MH	MHfe	MHdl	MHbdl	P	Pdl	Pbdl	Pbdl	Pbdl
1500	3	0	.5	.3967871	.2986469	.2817091	.2817091	.5969171	.5565609	.5480785	.3298333	.3146427
2500	5	0	.5	.4740359	.4839835	.4638534	.4427189	.6270486	.5969171	.5730969	.4638534	.4317291
3500	7	0	.5	.5394442	.5032296	.4937104	.4638534	.6196773	.5811626	.5306505	.4534203	.4204284
5000	10	0	.5	.5125524	.5032296	.4937104	.4839835	.5306505	.5216896	.4937104	.4839835	.4839835
10000	20	0	.5	.5565609	.7626008	.7626008	.7509993	.6485677	.6343185	.6196773	.6196773	.6196773
3000	3	0	.5	.589101	.4839835	.3843826	.3582039	.5969171	.5216896	.5032296	.4937104	.3967871
5000	5	0	.5	.6343185	.5565609	.5032296	.4839835	.6196773	.5125524	.5032296	.5394442	.4839835
7000	7	0	.5	.6555532	.6046156	.5648982	.5032296	.6555532	.5969171	.5125524	.5730969	.5216896
10000	10	0	.5	.6414905	.5730969	.5480785	.5032296	.6343185	.5648982	.5394442	.5648982	.5306505
20000	20	0	.5	.6485677	.7509993	.7451107	.7147307	.66245	.6122009	.589101	.6692608	.6692608
7500	3	0	.5	.6759882	.6196773	.5394442	.4740359	.6343185	.5480785	.4937104	.6270486	.5394442
12500	5	0	.5	.6759882	.66245	.5730969	.5306505	.6343185	.5648982	.5125524	.66245	.5730969
17500	7	0	.5	.6892024	.6414905	.5969171	.5480785	.5811626	.5306505	.5125524	.6485677	.6046156
25000	10	0	.5	.7451107	.6414905	.6122009	.5811626	.6692608	.6122009	.5811626	.6485677	.6122009
50000	20	0	.5	.7209369	.7147307	.6692608	.6414905	.6826346	.6270486	.6046156	.7209369	.6692608
1500	3	0	.1	.8430362	.9653186	.9359006	.9094999	.4937104	.3582039	.3146427	.957058	.9094999
2500	5	0	.1	1.126051	1.324655	1.316324	1.309962	.6046156	.4087909	.3298333	1.312094	1.299134
3500	7	0	.1	1.240564	1.521526	1.521526	1.517893	.589101	.3843826	.3582039	1.502212	1.502212
5000	10	0	.1	1.425987	1.543813	1.543813	1.54451	.4087909	.3146427	.2817091	1.571544	1.571891
10000	20	0	.1	1.554143	.3715373	.3715373	.3715373	.5306505	.5032296	.4740359	.6196773	.6196773

Create PDF in your applications with the Pdfcrowd HTML to PDF API PDFCROWD

3000	3	0	.1	.7391617	.8579044	.790639	.7568289	.5306505	.4204284	.4087909	.8579044	.7795832
5000	5	0	.1	.7960904	1.162235	1.110243	1.083993	.4317291	.3146427	.2986469	1.153412	1.097269
7000	7	0	.1	.918428	1.276617	1.26963	1.250456	.5730969	.4839835	.4839835	1.264911	1.25774
10000	10	0	.1	1.07383	1.459918	1.455936	1.439274	.5565609	.4839835	.4204284	1.443551	1.439274
20000	20	0	.1	1.419859	1.488083	1.488083	1.490339	.7509993	.7209369	.6826346	1.530111	1.530111
7500	3	0	.1	.5394442	.6956939	.589101	.5394442	.4427189	.3843826	.3843826	.6956939	.589101
12500	5	0	.1	.5969171	.9315793	.8121515	.7270763	.5216896	.4534203	.4204284	.9228434	.8014924
17500	7	0	.1	.6692608	1.080625	.981509	.927227	.6826346	.6270486	.5648982	1.07383	.969407
25000	10	0	.1	.7021111	1.212266	1.176622	1.135324	.7626008	.7331507	.7021111	1.198849	1.165144
50000	20	0	.1	.8627804	1.520628	1.517893	1.497999	1.080625	1.077237	1.0494	1.511261	1.507312
1500	3	.822467	.5	1.129159	1.093979	.927227	.848033	1.250456	1.01642	.9444522	1.087341	.8912407
2500	5	.822467	.5	1.240564	1.243057	1.023909	.9653186	1.299134	1.01642	.9444522	1.209612	.9775019
3500	7	.822467	.5	1.358175	1.388809	1.179453	1.119781	1.413591	1.042228	.981509	1.344734	1.080625
5000	10	.822467	.5	1.424468	1.481131	1.348625	1.294724	1.468928	1.107028	1.066954	1.440708	1.238059
10000	20	.822467	.5	1.549835	1.573836	1.576566	1.566448	1.571891	1.271971	1.255325	1.580683	1.50831
3000	3	.822467	.5	1.369306	1.352476	1.001154	.9359006	1.39393	1.045825	.969407	1.350555	.9854897
5000	5	.822467	.5	1.442134	1.449138	1.0494	.981509	1.446361	1.059995	.9972763	1.443551	1.023909
7000	7	.822467	.5	1.526761	1.54311	1.18507	1.107028	1.53255	1.093979	1.042228	1.537999	1.14142
10000	10	.822467	.5	1.571891	1.580936	1.264911	1.217534	1.573836	1.100541	1.080625	1.580225	1.238059
20000	20	.822467	.5	1.570465	1.517893	1.459918	1.440708	1.565117	1.165144	1.165144	1.530111	1.387083
7500	3	.822467	.5	1.569713	1.569328	1.227905	1.135324	1.565567	1.264911	1.198849	1.569713	1.227905
12500	5	.822467	.5	1.58106	1.581088	1.135324	1.056485	1.580519	1.14142	1.077237	1.58106	1.129159
17500	7	.822467	.5	1.580984	1.577897	1.059995	1.020176	1.581139	1.023909	.9775019	1.57829	1.059995
25000	10	.822467	.5	1.556458	1.502212	1.100541	1.0494	1.549193	1.063485	1.045825	1.503246	1.093979
50000	20	.822467	.5	1.390525	1.187855	1.170914	1.162235	1.372938	1.066954	1.070402	1.187855	1.156369
1500	3	.822467	.1	1.129159	1.267277	1.214907	1.18507	.8329166	.5306505	.5125524	1.267277	1.196123
2500	5	.822467	.1	1.436381	1.554143	1.546557	1.537999	1.045825	.6196773	.5730969	1.549835	1.537999
3500	7	.822467	.1	1.50831	1.573836	1.574138	1.576322	1.034969	.66245	.6196773	1.579	1.579161
5000	10	.822467	.1	1.581088	1.316324	1.316324	1.324655	1.15931	.790639	.7626008	1.388809	1.388809
10000	20	.822467	.1	1.233009	.2230471	.2230471	.2230471	1.380087	1.209612	1.170914	.4087909	.4087909
3000	3	.822467	.1	1.294724	1.418305	1.288022	1.214907	1.107028	.6892024	.6196773	1.418305	1.276617
5000	5	.822467	.1	1.342773	1.513192	1.468928	1.440708	1.170914	.7451107	.7084561	1.511261	1.454593
7000	7	.822467	.1	1.484641	1.580427	1.571544	1.566879	1.252897	.848033	.7795832	1.579873	1.567721
10000	10	.822467	.1	1.566879	1.511261	1.524175	1.530111	1.356285	.9049862	.8379976	1.521526	1.538749
20000	20	.822467	.1	1.425987	.7960904	.8277922	.8277922	1.545882	1.390525	1.354385	.848033	.886589
7500	3	.822467	.1	1.397297	1.489215	1.193382	1.103793	1.344734	.8676174	.8329166	1.489215	1.187855
12500	5	.822467	.1	1.461229	1.571544	1.336828	1.240564	1.449138	.9139803	.8819014	1.570831	1.328755
17500	7	.822467	.1	1.547889	1.565117	1.486943	1.44496	1.536476	1.113441	1.077237	1.565117	1.476343
25000	10	.822467	.1	1.569713	1.505294	1.562767	1.552946	1.565567	1.250456	1.196123	1.506307	1.556458
50000	20	.822467	.1	1.521526	.9444522	1.421404	1.430493	1.495848	1.568538	1.567303	.9486833	1.437832
1500	3	3.289868	.5	1.541684	1.516034	1.113441	1.027619	1.559202	1.18507	1.07383	1.514147	1.080625
2500	5	3.289868	.5	1.570831	1.579	1.320511	1.252897	1.556458	1.307819	1.220148	1.580225	1.267277
3500	7	3.289868	.5	1.495848	1.537999	1.410404	1.363785	1.481131	1.320511	1.271971	1.547889	1.344734
5000	10	3.289868	.5	1.372938	1.388809	1.492565	1.468928	1.350555	1.376532	1.332813	1.424468	1.40228
10000	20	3.289868	.5	.8529889	.7851369	1.565117	1.567721	.7960904	1.525906	1.523299	.9004388	1.579316
3000	3	3.289868	.5	1.577691	1.581136	1.198849	1.135324	1.573213	1.303499	1.212266	1.58111	1.201561
5000	5	3.289868	.5	1.521526	1.540231	1.196123	1.113441	1.516034	1.233009	1.14142	1.538749	1.198849
7000	7	3.289868	.5	1.385348	1.407182	1.264911	1.206942	1.37474	1.209612	1.15931	1.413591	1.230463
10000	10	3.289868	.5	1.198849	1.153412	1.363785	1.32671	1.193382	1.26963	1.227905	1.176622	1.332813
20000	20	3.289868	.5	.7739767	.6196773	1.550471	1.5511	.7509993	1.458599	1.44496	.6485677	1.53255
7500	3	3.289868	.5	1.451882	1.495848	1.296935	1.206942	1.473903	1.369306	1.296935	1.492565	1.299134
12500	5	3.289868	.5	1.296935	1.350555	1.248002	1.162235	1.314215	1.278921	1.222747	1.348625	1.250456
17500	7	3.289868	.5	1.206942	1.179453	1.153412	1.09067	1.214907	1.204259	1.129159	1.182269	1.147449

Create PDF in your applications with the Pdfcrowd HTML to PDF API PDFCROWD

25000	10	3.289868	.5	.973468	.8912407	1.193382	1.165144	.9775019	1.179453	1.129159	.8912407	1.187855
50000	20	3.289868	.5	.5125524	.3582039	1.350555	1.340802	.5125524	1.328755	1.309962	.3715373	1.342773
1500	3	3.289868	.1	1.550471	1.573836	1.514147	1.50117	1.475127	.848033	.7509993	1.573836	1.493667
2500	5	3.289868	.1	1.577479	1.537999	1.573213	1.575281	1.549835	.969407	.927227	1.54311	1.577691
3500	7	3.289868	.1	1.50831	1.344734	1.410404	1.418305	1.578097	.973468	.9359006	1.365635	1.443551
5000	10	3.289868	.1	1.255325	.8226239	.9359006	.9049862	1.546557	1.173776	1.173776	.8627804	1.001154
10000	20	3.289868	.1	.4317291	.09995	.09995	.09995	1.179453	1.509301	1.505294	.09995	.09995
3000	3	3.289868	.1	1.573836	1.580984	1.430493	1.356285	1.554143	.9315793	.8819014	1.580984	1.418305
5000	5	3.289868	.1	1.565567	1.493667	1.575551	1.565567	1.580114	.993373	.957058	1.497999	1.570092
7000	7	3.289868	.1	1.455936	1.309962	1.563726	1.568538	1.547889	1.063485	1.03861	1.314215	1.570465
10000	10	3.289868	.1	1.243057	1.001154	1.383604	1.395618	1.443551	1.225333	1.220148	1.020176	1.415171
20000	20	3.289868	.1	.6485677	.2636475	.66245	.6270486	.9528851	1.554143	1.558124	.2817091	.7270763
7500	3	3.289868	.1	1.578832	1.574433	1.233009	1.103793	1.574433	.8579044	.7960904	1.574433	1.230463
12500	5	3.289868	.1	1.515094	1.395618	1.466394	1.367476	1.536476	1.020176	.9854897	1.395618	1.462532
17500	7	3.289868	.1	1.408797	1.182269	1.537999	1.476343	1.431978	1.173776	1.173776	1.187855	1.534141
25000	10	3.289868	.1	1.255325	.918428	1.575281	1.559202	1.294724	1.303499	1.301322	.9228434	1.572565
50000	20	3.289868	.1	.5969171	.2817091	1.516034	1.523299	.6046156	1.56325	1.566448	.2817091	1.519724
1500	3	29.60881	.5	1.398967	1.454593	1.267277	1.176622	1.350555	1.458599	1.264911	1.450514	1.262533
2500	5	29.60881	.5	1.020176	1.176622	1.395618	1.336828	.9612024	1.546557	1.387083	1.176622	1.363785
3500	7	29.60881	.5	.7683163	.9444522	1.529284	1.493667	.7270763	1.581136	1.52845	.957058	1.499063
5000	10	29.60881	.5	.4204284	.5480785	1.58106	1.578832	.3967871	1.551722	1.581139	.5730969	1.576804
10000	20	29.60881	.5	.09995	.1412799	1.305665	1.309962	.1412799	1.245536	1.358175	.1412799	1.365635
3000	3	29.60881	.5	1.225333	1.330789	1.376532	1.240564	1.13838	1.533349	1.408797	1.316324	1.387083
5000	5	29.60881	.5	.8529889	1.01264	1.416743	1.318423	.848033	1.545199	1.398967	.993373	1.419859
7000	7	29.60881	.5	.6046156	.7209369	1.472671	1.430493	.5811626	1.574433	1.50117	.7209369	1.473903
10000	10	29.60881	.5	.2986469	.4427189	1.563726	1.54451	.2817091	1.573213	1.575814	.4427189	1.560253
20000	20	29.60881	.5	.09995	.09995	1.483479	1.495848	.09995	1.352476	1.447753	.09995	1.492565
7500	3	29.60881	.5	.9315793	1.080625	1.419859	1.290267	.9486833	1.521526	1.390525	1.063485	1.427498
12500	5	29.60881	.5	.6826346	.7147307	1.413591	1.307819	.6826346	1.530111	1.392232	.7209369	1.416743
17500	7	29.60881	.5	.4317291	.5730969	1.416743	1.33882	.4534203	1.552946	1.439274	.5730969	1.416743
25000	10	29.60881	.5	.1995996	.3582039	1.514147	1.468928	.1995996	1.578097	1.535705	.3582039	1.513192
50000	20	29.60881	.5	0	0	1.572892	1.578477	0	1.481131	1.526761	0	1.573528
1500	3	29.60881	.1	1.40228	1.38185	1.530111	1.497999	1.440708	1.32671	1.240564	1.38185	1.522416
2500	5	29.60881	.1	1.059995	1.01642	1.539493	1.552946	1.179453	1.429	1.328755	1.01642	1.549835
3500	7	29.60881	.1	.6956939	.6196773	1.324655	1.344734	.848033	1.547227	1.504274	.6270486	1.358175
5000	10	29.60881	.1	.4317291	.3443254	.8379976	.7851369	.5648982	1.580519	1.580604	.3582039	.9139803
10000	20	29.60881	.1	0	0	.09995	0	.09995	1.225333	1.248002	0	.1412799
3000	3	29.60881	.1	1.367476	1.352476	1.485796	1.410404	1.388809	1.288022	1.162235	1.352476	1.482309
5000	5	29.60881	.1	1.008836	.9775019	1.571544	1.535705	1.077237	1.39393	1.276617	.981509	1.568538
7000	7	29.60881	.1	.7509993	.6826346	1.560253	1.574138	.8529889	1.524175	1.488083	.6826346	1.566448
10000	10	29.60881	.1	.3843826	.3298333	1.372938	1.408797	.4839835	1.580683	1.573528	.3443254	1.405557
20000	20	29.60881	.1	.09995	0	.5032296	.5032296	.09995	1.260143	1.278921	0	.5306505
7500	3	29.60881	.1	1.397297	1.358175	1.367476	1.18507	1.412002	1.262533	1.201561	1.358175	1.365635
12500	5	29.60881	.1	1.042228	.9486833	1.53255	1.410404	1.063485	1.424468	1.328755	.9486833	1.53255
17500	7	29.60881	.1	.6826346	.5306505	1.580114	1.540231	.7147307	1.496927	1.450514	.5306505	1.580114
25000	10	29.60881	.1	.3443254	.2817091	1.530111	1.566011	.3843826	1.580225	1.572892	.2817091	1.530111
50000	20	29.60881	.1	.09995	0	.9854897	1.066954	.09995	1.260143	1.26963	0	.9894443

Mean error

Create PDF in your applications with the Pdfcrowd HTML to PDF API PDFCROWD

ssl	ssh	tsq1	cb	MH	MHfe	MHdl	MHbdl	P	Pdl	Pbdl	Pbdl	Pbdl
1500	3	0	.5	.4712191	.4060436	.4044334	.403688	.4567474	.4578675	.4583136	.4431126	.4417789
2500	5	0	.5	.3498405	.3309344	.3300788	.3288052	.3502484	.3495052	.3486408	.3445097	.3422817
3500	7	0	.5	.3150368	.291307	.2903057	.2890463	.3055736	.304611	.3046622	.2990821	.2981522
5000	10	0	.5	.2416527	.2518752	.2514273	.2508843	.240802	.2424697	.2425761	.2419893	.2414877
10000	20	0	.5	.1746276	.2169084	.216871	.2165275	.1746024	.1751149	.1751411	.1915466	.1913475
3000	3	0	.5	.3590669	.3286231	.3319523	.3329946	.3168364	.3259727	.329891	.3395821	.3431684
5000	5	0	.5	.2843731	.2652211	.2637649	.2635784	.2572818	.2595866	.2604712	.2694202	.2685156
7000	7	0	.5	.22977	.2223489	.220684	.2202144	.2111365	.214286	.2149104	.2241575	.222624
10000	10	0	.5	.1862951	.1900118	.1876688	.1867661	.1736174	.1739007	.1742429	.1889893	.1862182
20000	20	0	.5	.1311681	.1442996	.1438725	.1430376	.1225805	.1230379	.1228308	.1385511	.13795
7500	3	0	.5	.2300484	.2227337	.229966	.2317334	.2050903	.2099322	.2124457	.2235807	.2310291
12500	5	0	.5	.1824351	.1754383	.1756522	.1757678	.1615625	.1625111	.1629762	.1763358	.1767486
17500	7	0	.5	.1471517	.1421187	.1418193	.1419231	.1302914	.1311613	.13166	.14239	.1419275
25000	10	0	.5	.1260137	.1235313	.1236805	.1234055	.1137667	.1148308	.1148696	.1238031	.1239278
50000	20	0	.5	.0870424	.0872516	.0867088	.0863748	.0793494	.0795196	.0795197	.0871218	.0865445
1500	3	0	.1	.7301175	.8966481	.8977152	.8975578	.5617582	.6085333	.617741	.8992203	.8992703
2500	5	0	.1	.701699	.9203757	.9192826	.9187987	.4311123	.4605748	.4711737	.9313791	.9296491
3500	7	0	.1	.6767299	.9115657	.9115174	.9112428	.3644467	.3771524	.3831712	.9281025	.9279457
5000	10	0	.1	.6559666	.9065241	.9065241	.9062943	.2943003	.3025943	.3063134	.9252464	.9252555
10000	20	0	.1	.6647638	.9165134	.9165134	.9164279	.2325951	.2352474	.2373035	.9425039	.9425039
3000	3	0	.1	.4491766	.5529691	.5498678	.5483526	.3757303	.4010016	.4130245	.5521732	.5486637
5000	5	0	.1	.413855	.5703429	.5680455	.5663409	.3067639	.3227929	.3296714	.566474	.5631706
7000	7	0	.1	.4066217	.5856659	.5836534	.5820037	.2727151	.2805443	.2839834	.5787301	.5763734
10000	10	0	.1	.389361	.5929371	.5918611	.5901386	.2351484	.2397583	.2417792	.5856605	.5843889
20000	20	0	.1	.3917223	.6170677	.6170677	.6164418	.2017032	.2024684	.2035694	.6117024	.6117024
7500	3	0	.1	.3485029	.376399	.3644863	.3583791	.2574199	.2671586	.2746329	.3796214	.3676574
12500	5	0	.1	.2819548	.3382695	.3273792	.3222374	.2211945	.2261091	.229009	.3386892	.3277224
17500	7	0	.1	.2375534	.3281492	.3173134	.3130026	.2046549	.2087431	.2109924	.3261849	.3146416
25000	10	0	.1	.2059975	.3112722	.3073035	.3021941	.1860272	.1878911	.1892378	.3096884	.305399
50000	20	0	.1	.1655561	.3136669	.3124942	.3087861	.1752979	.1756011	.1759444	.3104792	.3091006
1500	3	.822467	.5	.6504084	.5828034	.5518801	.5464974	.6487998	.597363	.5921234	.6413873	.6004621
2500	5	.822467	.5	.5241989	.5237569	.4922968	.4859968	.5443523	.4961919	.4901548	.5437769	.5034366
3500	7	.822467	.5	.482176	.5217374	.4839073	.4762564	.5134877	.4402253	.4356899	.5253598	.4757014
5000	10	.822467	.5	.432225	.4992427	.461439	.4537846	.4697063	.394251	.3913833	.499199	.4467016
10000	20	.822467	.5	.3790184	.4885019	.4592669	.4505016	.4212129	.3364696	.334557	.4711384	.4262646
3000	3	.822467	.5	.6217467	.6050705	.542425	.5333701	.5773115	.5117553	.5054185	.6227303	.5570252
5000	5	.822467	.5	.5057573	.5154486	.432347	.4238615	.4869051	.4028485	.3984897	.5240379	.4400468
7000	7	.822467	.5	.4607722	.4904583	.3978781	.3920054	.4554302	.3644249	.361971	.4934495	.3971041
10000	10	.822467	.5	.4473163	.5005833	.387096	.3810764	.4491395	.3304839	.3285644	.4999305	.3801272
20000	20	.822467	.5	.3857768	.4611564	.3390017	.3360996	.3930976	.2646792	.2647696	.4567341	.3262516
7500	3	.822467	.5	.5705413	.5740565	.5010319	.4958406	.5174196	.4449096	.4370474	.5755054	.5047538
12500	5	.822467	.5	.4795814	.5000885	.3710367	.3677165	.4538387	.3400084	.3367616	.5013496	.372849
17500	7	.822467	.5	.4267488	.464531	.317394	.3146755	.4132562	.2895701	.2882456	.4645191	.3172087
25000	10	.822467	.5	.4155792	.4688962	.2850421	.2832382	.4100469	.2598962	.2594877	.4689256	.2839854
50000	20	.822467	.5	.392681	.4636526	.2405521	.239369	.3947658	.2187567	.2189009	.4629225	.2375584
1500	3	.822467	.1	.8091129	.9797029	.9738754	.9726547	.690821	.7238253	.7308548	.9844975	.9774943
2500	5	.822467	.1	.8371254	1.067171	1.059916	1.057547	.6299177	.647934	.6606287	1.091225	1.080569
3500	7	.822467	.1	.8193672	1.052871	1.047877	1.045767	.5398063	.5447872	.5498174	1.080275	1.073779
5000	10	.822467	.1	.8302003	1.080622	1.07586	1.071469	.5180397	.5111807	.5136052	1.114682	1.107599

Create PDF in your applications with the Pdfcrowd HTML to PDF API PDFCROWD

10000	20	.822467	.1	.82942	1.086935	1.085862	1.082119	.4710796	.4643634	.4633918	1.130767	1.128432
3000	3	.822467	.1	.640738	.7769258	.7407557	.7334958	.6196457	.6302986	.6419444	.7782699	.7401284
5000	5	.822467	.1	.5840189	.7723485	.7455303	.7390442	.5062532	.5026434	.5083516	.7709722	.7418819
7000	7	.822467	.1	.5995053	.8310801	.797748	.7903064	.4781332	.4644691	.4669331	.8319937	.7958782
10000	10	.822467	.1	.6174476	.8771433	.8413088	.8315522	.4602293	.4421066	.4430456	.8814	.8410754
20000	20	.822467	.1	.6384415	.9189028	.8904232	.8782215	.4448678	.4247852	.423723	.9251449	.8926908
7500	3	.822467	.1	.5910489	.6857895	.5737572	.5547067	.5268141	.5020685	.5071942	.6919728	.5775018
12500	5	.822467	.1	.5040613	.6846875	.5428013	.5259199	.4624255	.4443338	.4483805	.6874905	.5428293
17500	7	.822467	.1	.507571	.7350918	.5713424	.556992	.4769391	.4480204	.4497024	.7362947	.5683837
25000	10	.822467	.1	.4597682	.7152236	.5510291	.5388508	.4294633	.4024634	.4036433	.7151181	.5477581
50000	20	.822467	.1	.4580503	.7746564	.5778863	.5694255	.4312941	.3981916	.3980973	.774945	.5734601
1500	3	3.289868	.5	1.098219	.9986685	.8374709	.8251347	1.061689	.8072544	.7864257	1.074642	.8974623
2500	5	3.289868	.5	1.116771	1.045474	.8168658	.806679	1.108022	.7366502	.7152613	1.0847	.8318149
3500	7	3.289868	.5	1.086397	1.01395	.7762288	.768492	1.106691	.6794412	.664145	1.049698	.7814178
5000	10	3.289868	.5	1.124374	1.029652	.7573893	.7508299	1.162283	.6262538	.615689	1.062777	.7495521
10000	20	3.289868	.5	1.196958	1.075556	.7555706	.7537452	1.265409	.589344	.5867534	1.107642	.7387296
3000	3	3.289868	.5	1.161412	1.119805	.832199	.8166598	1.044226	.7384592	.7169173	1.145289	.8521694
5000	5	3.289868	.5	1.124909	1.10124	.712189	.6965156	1.056797	.6196719	.6021498	1.115633	.7240881
7000	7	3.289868	.5	1.137283	1.103106	.6648018	.6541889	1.096248	.5666057	.5558059	1.113858	.6684293
10000	10	3.289868	.5	1.182519	1.125728	.6291287	.6225853	1.159808	.5210696	.5146244	1.136625	.6286204
20000	20	3.289868	.5	1.234773	1.160287	.5999522	.5977016	1.236415	.4754481	.4728311	1.169022	.5929738
7500	3	3.289868	.5	1.159872	1.165467	.8262238	.8123179	1.004366	.6650541	.6518061	1.17049	.8334939
12500	5	3.289868	.5	1.152923	1.145553	.6784902	.6655036	1.064215	.5704721	.5617676	1.149824	.6858815
17500	7	3.289868	.5	1.131905	1.14465	.5804975	.5710205	1.073024	.4842397	.4773077	1.147607	.5856794
25000	10	3.289868	.5	1.182032	1.184611	.518878	.5124532	1.142215	.4386819	.4346501	1.186636	.5210448
50000	20	3.289868	.5	1.306798	1.252128	.4522029	.4491624	1.284486	.3906581	.3887666	1.253765	.4507235
1500	3	3.289868	.1	1.242232	1.435	1.33774	1.329917	1.565121	1.368453	1.368873	1.45917	1.357009
2500	5	3.289868	.1	1.240869	1.490715	1.365165	1.359763	1.4378	1.180371	1.185859	1.531298	1.395571
3500	7	3.289868	.1	1.278069	1.543079	1.395292	1.388675	1.44798	1.10403	1.109649	1.595117	1.43513
5000	10	3.289868	.1	1.346495	1.624541	1.451421	1.440096	1.529214	1.12702	1.13017	1.691005	1.495741
10000	20	3.289868	.1	1.388982	1.657648	1.467563	1.451873	1.531753	1.067621	1.068789	1.73557	1.517527
3000	3	3.289868	.1	1.128723	1.324863	1.096557	1.079249	1.499463	1.249632	1.25701	1.332902	1.103376
5000	5	3.289868	.1	1.15746	1.451821	1.14317	1.128171	1.448225	1.111049	1.115961	1.458978	1.144774
7000	7	3.289868	.1	1.225824	1.564949	1.171333	1.158436	1.483733	1.073011	1.076451	1.57516	1.174029
10000	10	3.289868	.1	1.245367	1.604376	1.17772	1.167767	1.465974	1.026459	1.028219	1.623705	1.182804
20000	20	3.289868	.1	1.364325	1.746893	1.2162	1.21108	1.56983	1.039042	1.040416	1.770836	1.222514
7500	3	3.289868	.1	1.073305	1.315803	.861911	.827837	1.338732	1.064677	1.066834	1.318701	.8610226
12500	5	3.289868	.1	1.092689	1.448392	.8572829	.821618	1.361973	1.018672	1.022798	1.450923	.8569964
17500	7	3.289868	.1	1.139973	1.557013	.8512647	.822317	1.421097	.9984914	1.00009	1.561071	.8510684
25000	10	3.289868	.1	1.182149	1.644421	.8523998	.8320673	1.46687	.9954796	.996644	1.648337	.8508612
50000	20	3.289868	.1	1.288471	1.813105	.8112845	.8056368	1.544905	.971311	.9721493	1.817937	.8104208
1500	3	29.60881	.5	2.835484	2.698255	2.075823	2.062895	2.4519	1.442292	1.367203	2.85755	2.212709
2500	5	29.60881	.5	3.045852	2.72629	1.960416	1.95239	2.799184	1.385904	1.264666	2.891287	2.082396
3500	7	29.60881	.5	3.169418	2.790623	1.950595	1.943359	2.978256	1.403367	1.289805	2.93766	2.057623
5000	10	29.60881	.5	3.384575	2.957208	1.999042	1.995334	3.216201	1.449887	1.353578	3.125252	2.109016
10000	20	29.60881	.5	3.479219	2.95688	1.942335	1.940102	3.36916	1.446683	1.394518	3.13276	2.052545
3000	3	29.60881	.5	2.938535	2.929235	2.062602	2.041107	2.430007	1.35387	1.287169	2.985195	2.125933
5000	5	29.60881	.5	3.16102	3.06785	1.89842	1.87712	2.794249	1.269373	1.174822	3.125126	1.970666
7000	7	29.60881	.5	3.301528	3.182657	1.844373	1.829401	2.99005	1.241552	1.159753	3.232728	1.894521
10000	10	29.60881	.5	3.514875	3.329159	1.875935	1.863085	3.233874	1.266301	1.19305	3.378189	1.924178
20000	20	29.60881	.5	3.595915	3.373207	1.754213	1.748384	3.37091	1.20643	1.166523	3.424314	1.793794
7500	3	29.60881	.5	3.055303	3.277585	2.066307	2.034138	2.456186	1.262835	1.216245	3.295233	2.092041
12500	5	29.60881	.5	3.219996	3.389116	1.814926	1.780289	2.793935	1.150826	1.087863	3.402568	1.836061

Create PDF in your applications with the Pdfcrowd HTML to PDF API PDFCROWD

17500	7	29.60881	.5	3.261693	3.387246	1.619203	1.596124	2.931681	1.048422	1.001921	3.397683	1.639534
25000	10	29.60881	.5	3.46294	3.500086	1.567098	1.547475	3.163824	1.027196	.9861449	3.508479	1.579384
50000	20	29.60881	.5	3.64155	3.616289	1.505646	1.496093	3.379988	.9997249	.9769413	3.626008	1.518727
1500	3	29.60881	.1	2.944488	3.16363	2.679165	2.666186	5.296314	3.464438	3.406734	3.236644	2.743814
2500	5	29.60881	.1	3.068225	3.222859	2.608369	2.598814	5.706535	3.272338	3.23299	3.311877	2.684541
3500	7	29.60881	.1	3.273469	3.415056	2.649639	2.641997	6.264079	3.34092	3.309199	3.527461	2.738332
5000	10	29.60881	.1	3.358738	3.462969	2.642491	2.636757	6.564611	3.361871	3.338391	3.592664	2.746612
10000	20	29.60881	.1	3.5115	3.600779	2.636497	2.633167	7.033995	3.342876	3.331312	3.743448	2.740364
3000	3	29.60881	.1	2.811246	3.104478	2.381363	2.355416	5.01967	3.290481	3.254857	3.130354	2.409518
5000	5	29.60881	.1	3.128018	3.452878	2.357971	2.331758	5.776176	3.121596	3.095619	3.483162	2.389617
7000	7	29.60881	.1	3.26273	3.571585	2.379383	2.359409	6.107623	3.131152	3.110402	3.608325	2.416168
10000	10	29.60881	.1	3.437932	3.783284	2.402266	2.385097	6.52211	3.158779	3.144835	3.821847	2.439619
20000	20	29.60881	.1	3.594078	3.881563	2.419702	2.410451	6.983307	3.175425	3.168315	3.922399	2.459648
7500	3	29.60881	.1	2.831182	3.26546	2.159495	2.113691	4.845424	3.074869	3.043418	3.27259	2.168574
12500	5	29.60881	.1	3.124128	3.640943	2.092838	2.039736	5.678512	3.047292	3.032867	3.647908	2.104628
17500	7	29.60881	.1	3.32996	3.855959	2.083818	2.031063	6.192743	3.046592	3.035933	3.864363	2.093432
25000	10	29.60881	.1	3.528115	4.101597	2.139905	2.101124	6.667933	3.116561	3.108756	4.107221	2.149623
50000	20	29.60881	.1	3.607685	4.129018	2.101036	2.080617	6.939243	3.063957	3.060606	4.136329	2.112932

SE of mean error

ssl	ssh	tsq1	cb	MH	MHfe	MHdl	MHbdl	P	Pdl	Pbdl	Pbdl	Pbdl
1500	3	0	.5	.0003775	.0003214	.0003188	.0003184	.0003684	.0003666	.0003643	.0003621	.0003599
2500	5	0	.5	.0002827	.0002506	.0002506	.0002502	.0002745	.0002779	.0002771	.0002735	.0002738
3500	7	0	.5	.000247	.0002162	.0002164	.0002158	.0002353	.0002333	.0002335	.0002334	.0002332
5000	10	0	.5	.000188	.0001824	.0001823	.0001821	.0001861	.000187	.0001874	.0001851	.0001845
10000	20	0	.5	.0001386	.0001415	.0001415	.0001415	.000138	.0001384	.000138	.0001378	.0001378
3000	3	0	.5	.0002904	.0002522	.0002508	.0002518	.0002429	.0002458	.0002468	.000271	.0002689
5000	5	0	.5	.000216	.0001999	.0001994	.0001992	.0001916	.0001944	.0001969	.0002019	.0002006
7000	7	0	.5	.000179	.000174	.0001726	.0001721	.000168	.0001684	.000169	.0001754	.0001738
10000	10	0	.5	.0001494	.0001403	.0001398	.0001389	.0001338	.0001348	.0001351	.0001417	.000141
20000	20	0	.5	.0001002	.0001057	.0001055	.0001051	.0000949	.0000946	.0000947	.0001027	.0001024
7500	3	0	.5	.0001823	.0001754	.0001799	.000181	.0001589	.0001618	.0001623	.000176	.0001811
12500	5	0	.5	.0001377	.0001324	.0001346	.0001351	.000122	.0001234	.0001239	.0001328	.0001351
17500	7	0	.5	.0001173	.0001122	.0001115	.0001117	.0001028	.0001032	.0001033	.0001123	.0001118
25000	10	0	.5	.0000983	.0000944	.0000951	.000095	.0000886	.00009	.0000902	.0000947	.0000954
50000	20	0	.5	.0000688	.0000681	.0000674	.0000672	.0000629	.000063	.000063	.0000682	.0000675
1500	3	0	.1	.0003995	.0003617	.000363	.0003628	.0003971	.0004937	.0005138	.0003956	.000395
2500	5	0	.1	.00037	.0003169	.0003143	.0003127	.0003811	.0004325	.0004418	.0003467	.0003433
3500	7	0	.1	.0003233	.0002654	.0002653	.0002644	.0003155	.000331	.0003359	.0002911	.0002908
5000	10	0	.1	.000282	.0002217	.0002217	.0002212	.0002363	.0002495	.0002519	.000244	.000244
10000	20	0	.1	.0001985	.0001617	.0001617	.0001616	.0001891	.0001937	.0001949	.000176	.000176
3000	3	0	.1	.0003387	.0003809	.0003751	.000373	.0003438	.0003979	.0004263	.000386	.0003795
5000	5	0	.1	.0002857	.0003251	.0003201	.0003173	.0002535	.0002789	.0002925	.0003323	.0003263
7000	7	0	.1	.0002594	.0002859	.0002817	.0002791	.0002211	.0002347	.0002378	.0002973	.0002922
10000	10	0	.1	.0002281	.0002429	.0002416	.0002394	.0001932	.0001983	.0001996	.0002538	.0002522
20000	20	0	.1	.000174	.0001777	.0001777	.0001771	.0001502	.0001512	.0001521	.0001878	.0001878

Create PDF in your applications with the Pdfcrowd HTML to PDF API PDFCROWD

7500	3	0	.1	.0002474	.0002527	.0002396	.0002372	.0001981	.0002101	.0002275	.0002551	.0002423
12500	5	0	.1	.0001993	.0002308	.0002185	.0002145	.0001693	.0001762	.0001805	.0002325	.0002198
17500	7	0	.1	.0001673	.0002145	.0002047	.0002011	.0001538	.0001572	.0001587	.0002151	.000205
25000	10	0	.1	.0001513	.0001961	.0001928	.0001895	.000137	.0001393	.0001404	.0001964	.0001928
50000	20	0	.1	.0001179	.0001593	.0001577	.000155	.0001081	.0001087	.0001089	.0001603	.0001584
1500	3	.822467	.5	.0004807	.0004525	.0004336	.0004289	.0004794	.0004488	.0004395	.0004737	.0004564
2500	5	.822467	.5	.0003982	.0003787	.0003585	.0003554	.0004096	.0003748	.0003678	.0004033	.0003806
3500	7	.822467	.5	.000366	.000344	.0003167	.0003145	.0003867	.0003329	.0003286	.0003592	.0003304
5000	10	.822467	.5	.0003253	.0003213	.0002962	.0002931	.0003506	.000299	.0002965	.0003354	.0003052
10000	20	.822467	.5	.0002625	.0002457	.0002278	.0002262	.0002876	.0002325	.0002313	.0002633	.0002405
3000	3	.822467	.5	.0004658	.0004484	.0004018	.0003988	.0004273	.0003815	.0003773	.000461	.0004164
5000	5	.822467	.5	.0003845	.0003728	.0003236	.0003216	.0003719	.0003152	.0003129	.0003761	.0003285
7000	7	.822467	.5	.0003307	.0003325	.0002824	.0002786	.0003336	.0002698	.0002684	.0003346	.0002835
10000	10	.822467	.5	.000322	.0003195	.0002567	.0002551	.0003258	.000243	.0002417	.0003236	.0002587
20000	20	.822467	.5	.0002638	.0002526	.0002074	.000207	.0002708	.0001931	.000193	.0002568	.0002087
7500	3	.822467	.5	.0004283	.000421	.0003799	.000378	.0003857	.0003309	.000326	.0004218	.0003812
12500	5	.822467	.5	.0003588	.0003533	.000274	.0002724	.0003462	.0002584	.0002565	.0003539	.0002763
17500	7	.822467	.5	.0003304	.0003354	.000243	.0002406	.000328	.0002287	.0002273	.0003359	.0002437
25000	10	.822467	.5	.0003006	.0002955	.0002173	.0002164	.0003	.0002044	.0002039	.0002957	.000218
50000	20	.822467	.5	.0002432	.0002338	.0001594	.0001591	.0002439	.000149	.000149	.0002345	.0001593
1500	3	.822467	.1	.0004782	.0004624	.0004505	.0004483	.0006008	.0006594	.0006714	.0004934	.0004804
2500	5	.822467	.1	.000442	.0004049	.0003923	.0003884	.0005865	.000577	.0005835	.0004424	.0004258
3500	7	.822467	.1	.0003823	.0003494	.0003404	.0003371	.0004884	.0004833	.0004852	.0003728	.0003621
5000	10	.822467	.1	.0003396	.0003116	.0003031	.0002974	.0004437	.0004237	.0004244	.0003335	.0003221
10000	20	.822467	.1	.0002444	.0002259	.0002238	.0002191	.0003493	.0003365	.0003357	.0002444	.0002406
3000	3	.822467	.1	.0004956	.0005667	.0005089	.0004998	.0006012	.0005904	.0006108	.0005717	.0005117
5000	5	.822467	.1	.0004196	.0004743	.0004337	.0004266	.0004689	.0004407	.0004479	.0004842	.0004419
7000	7	.822467	.1	.0003776	.0004241	.0003778	.0003697	.0004218	.0003861	.0003865	.0004374	.0003888
10000	10	.822467	.1	.0003326	.0003746	.0003213	.0003133	.0003749	.0003295	.0003296	.000387	.0003295
20000	20	.822467	.1	.0002588	.0002788	.0002465	.000238	.0002953	.0002662	.0002661	.0002884	.0002525
7500	3	.822467	.1	.0004407	.0005185	.0004027	.0003907	.0005415	.0004798	.0004866	.0005191	.0004047
12500	5	.822467	.1	.0003721	.0004673	.0003433	.0003314	.0004124	.0003647	.000366	.0004683	.0003438
17500	7	.822467	.1	.0003606	.0004584	.0003189	.0003112	.0004052	.0003352	.000336	.0004599	.0003196
25000	10	.822467	.1	.0003231	.0004127	.0002848	.0002796	.0003403	.0002868	.0002869	.0004143	.0002863
50000	20	.822467	.1	.0002568	.000305	.0002138	.000212	.0002462	.0002072	.0002074	.0003067	.0002153
1500	3	3.289868	.5	.0008429	.0007809	.0006428	.0006402	.0007359	.0005935	.0005825	.0008067	.0006808
2500	5	3.289868	.5	.0008212	.0007219	.0005681	.000566	.0007335	.0005408	.0005254	.0007498	.0005941
3500	7	3.289868	.5	.0007452	.0006356	.0004994	.0004968	.0006982	.0004906	.0004773	.0006737	.0005327
5000	10	3.289868	.5	.0007243	.0005606	.0004227	.0004225	.0006933	.0004329	.0004236	.0005997	.0004552
10000	20	3.289868	.5	.0005579	.0004122	.0003226	.0003232	.0005538	.0003495	.0003464	.0004488	.0003586
3000	3	3.289868	.5	.0009069	.0008504	.0006634	.0006555	.0007559	.0005603	.0005417	.0008562	.0006771
5000	5	3.289868	.5	.0008405	.0007683	.0005335	.000527	.000743	.000468	.0004539	.0007755	.0005449
7000	7	3.289868	.5	.000794	.0006989	.0004753	.0004716	.0007207	.0004213	.0004126	.0007078	.0004859
10000	10	3.289868	.5	.0007197	.0005885	.0004299	.0004272	.0006734	.000382	.0003768	.0005962	.0004402
20000	20	3.289868	.5	.0005786	.0004497	.0003421	.0003415	.0005557	.00031	.0003077	.0004585	.0003506
7500	3	3.289868	.5	.0008794	.000878	.00061	.0005978	.0007259	.000492	.0004802	.0008807	.0006147
12500	5	3.289868	.5	.0008081	.0007623	.0005023	.0004967	.0007171	.0004205	.0004129	.0007635	.0005093
17500	7	3.289868	.5	.0007728	.0006976	.000419	.0004135	.0007062	.0003614	.0003549	.0006992	.0004235
25000	10	3.289868	.5	.0007246	.0006166	.0003735	.0003712	.0006728	.0003229	.0003194	.0006183	.0003763
50000	20	3.289868	.5	.0005974	.0004638	.0003061	.0003052	.0005652	.0002637	.0002623	.000465	.0003074
1500	3	3.289868	.1	.0008576	.0008881	.0007219	.0007121	.0017169	.0012803	.0012762	.0009266	.0007601
2500	5	3.289868	.1	.0007535	.0007582	.0005773	.0005702	.0014985	.0010162	.0010138	.0007878	.0005977
3500	7	3.289868	.1	.0006836	.0006794	.0004941	.0004881	.0013888	.000852	.0008506	.0007043	.0005129

Create PDF in your applications with the Pdfcrowd HTML to PDF API PDFCROWD

5000	10	3.289868	.1	.0006058	.0006012	.0004148	.0004096	.0012579	.0007734	.0007726	.0006277	.0004249
10000	20	3.289868	.1	.000454	.0004487	.0002804	.0002781	.0009862	.0005298	.0005309	.0004696	.0002872
3000	3	3.289868	.1	.0009401	.001054	.0007662	.0007505	.0016954	.0012399	.0012416	.0010621	.0007759
5000	5	3.289868	.1	.0008464	.000939	.000623	.000608	.0015438	.0009873	.000981	.0009506	.0006336
7000	7	3.289868	.1	.0007619	.0008483	.0005048	.000495	.0013801	.0008075	.0008068	.000859	.0005152
10000	10	3.289868	.1	.0006769	.0007411	.0004083	.0004033	.0012497	.0006643	.0006642	.0007548	.0004173
20000	20	3.289868	.1	.0005169	.0005449	.0002861	.0002856	.0009655	.0005057	.0005057	.0005538	.0002933
7500	3	3.289868	.1	.0008927	.0010558	.0006294	.0006093	.0016396	.0010548	.0010497	.0010548	.0006302
12500	5	3.289868	.1	.0008102	.0009549	.0005628	.0005413	.0014471	.0008695	.0008683	.000956	.0005662
17500	7	3.289868	.1	.000765	.0008971	.000509	.0004967	.0013108	.0007479	.0007471	.000898	.0005127
25000	10	3.289868	.1	.000723	.0008254	.0004383	.0004301	.0012088	.0006529	.0006529	.0008276	.0004426
50000	20	3.289868	.1	.0005596	.0005981	.0003165	.0003139	.0009514	.0004425	.0004425	.0005995	.00032
1500	3	29.60881	.5	.0017497	.0019211	.0016626	.0016642	.0013337	.0010493	.0009856	.0019992	.0017659
2500	5	29.60881	.5	.0014599	.0015834	.0013623	.0013624	.0011962	.0009881	.0008991	.001657	.0014675
3500	7	29.60881	.5	.001249	.0013728	.0011461	.001146	.0010572	.0008677	.0008184	.0014312	.0012373
5000	10	29.60881	.5	.0010125	.0012111	.0010626	.0010618	.0008165	.0008377	.0008111	.0012695	.0011486
10000	20	29.60881	.5	.0006373	.0008431	.0007356	.0007349	.0004905	.0006286	.0006266	.000879	.0008062
3000	3	29.60881	.5	.0017252	.0019723	.0016581	.0016592	.001324	.0009963	.0009398	.0019813	.0016883
5000	5	29.60881	.5	.0014873	.0017169	.0013803	.0013786	.0012204	.0009126	.0008441	.0017411	.0014454
7000	7	29.60881	.5	.0012795	.0014875	.0012542	.0012531	.001054	.0008311	.000792	.0015109	.001301
10000	10	29.60881	.5	.0010108	.0012532	.0011084	.0011079	.0008048	.0007558	.0007332	.0012723	.001148
20000	20	29.60881	.5	.0006336	.0008739	.0007821	.0007818	.0004808	.0005686	.0005645	.0008844	.0008089
7500	3	29.60881	.5	.0017666	.002147	.0016903	.0016809	.0013776	.0009618	.0009171	.0021503	.0017122
12500	5	29.60881	.5	.0014829	.0017722	.0014006	.0013994	.0012096	.0008443	.0007968	.0017758	.0014158
17500	7	29.60881	.5	.0012923	.0015438	.0012274	.0012229	.0010694	.0007504	.0007283	.0015508	.0012395
25000	10	29.60881	.5	.0010254	.0013148	.0010674	.0010657	.000838	.0006647	.0006497	.0013184	.0010786
50000	20	29.60881	.5	.0006575	.0009046	.0008313	.0008313	.0005014	.0005178	.0005146	.0009074	.0008423
1500	3	29.60881	.1	.0017922	.0018631	.0015753	.0015718	.0037717	.0025798	.0024988	.0019195	.0016325
2500	5	29.60881	.1	.0014412	.0014777	.0012118	.0012082	.0032559	.0021066	.0020579	.0015233	.0012611
3500	7	29.60881	.1	.0011546	.0012129	.0009823	.0009782	.0027556	.001767	.0017342	.0012417	.0010188
5000	10	29.60881	.1	.0009699	.0010326	.000843	.000839	.0023754	.0015555	.0015335	.0010457	.0008865
10000	20	29.60881	.1	.0005953	.0006984	.0005937	.0005917	.0015099	.0010949	.0010868	.0007092	.0006188
3000	3	29.60881	.1	.001875	.0020558	.0016466	.0016391	.0036967	.0024489	.0023917	.002072	.0016688
5000	5	29.60881	.1	.0014914	.0016559	.0012737	.0012658	.003196	.0019574	.0019245	.0016608	.0012998
7000	7	29.60881	.1	.0012959	.0014409	.0011144	.0011073	.0028794	.0017394	.001712	.0014504	.0011446
10000	10	29.60881	.1	.001014	.0011557	.0009467	.0009423	.0023771	.0015051	.001491	.0011631	.0009662
20000	20	29.60881	.1	.0006486	.0007587	.0006878	.0006854	.0015811	.0010886	.0010833	.0007637	.0007021
7500	3	29.60881	.1	.0018732	.0022036	.0017352	.0017266	.0037802	.0024611	.0024019	.0022038	.0017391
12500	5	29.60881	.1	.0015287	.0017986	.0013754	.0013625	.0032917	.0019465	.0019273	.0017978	.0013848
17500	7	29.60881	.1	.0012489	.0014376	.0011524	.0011496	.0027721	.0016	.0015885	.0014442	.0011605
25000	10	29.60881	.1	.0009784	.0011502	.0010384	.0010372	.0022475	.0014192	.0014122	.0011515	.0010446
50000	20	29.60881	.1	.0006258	.0007382	.0007249	.000725	.0015057	.0009711	.0009687	.0007396	.0007301

Mean bias

ssl	ssh	tsq1	cb	MH	MHfe	MHdl	MHbdl	P	Pdl	Pbdl	Pbdl	Pbdl
1500	3	0	.5	-.088919	.1001698	.0980312	.0972258	-.025265	-.0093157	-.0011856	.0350748	.0329799

Create PDF in your applications with the Pdfcrowd HTML to PDF API PDFCROWD

2500	5	0	.5	-.0241783	.1844787	.1830249	.1807797	.0410172	.0488666	.0525981	.1177741	.1150758
3500	7	0	.5	-.0324174	.1813832	.179905	.1775928	.0248229	.0242247	.0267319	.1103764	.1070246
5000	10	0	.5	-.0341131	.1821607	.1817143	.1805598	.017682	.0226436	.0242286	.1145145	.113039
10000	20	0	.5	-.025863	.1851749	.1851375	.1847221	.0183452	.0190984	.0198756	.1198059	.1196039
3000	3	0	.5	-.0446067	.0445863	.0397105	.0382867	.0152855	.0294834	.035376	.0228495	.016508
5000	5	0	.5	-.0158955	.0776283	.0728962	.0708242	.0310543	.0382423	.0414491	.0588588	.0520685
7000	7	0	.5	.0034652	.1020474	.0975211	.0950999	.0434435	.0477301	.0494269	.0832027	.0768749
10000	10	0	.5	-.0079959	.0929825	.0896339	.0870154	.0282661	.0305718	.0312087	.0745913	.0700645
20000	20	0	.5	-.0044721	.1004319	.0992205	.0971915	.0278904	.0291834	.0295541	.0824718	.0805345
7500	3	0	.5	.0021732	.0333286	.0221139	.0187148	.0402214	.0420045	.0439308	.0311907	.0196299
12500	5	0	.5	-.0117246	.0246592	.015416	.0127361	.0226404	.0238025	.0248276	.0222322	.0125102
17500	7	0	.5	-.0205987	.0175551	.0111725	.0088974	.0124158	.0127929	.0134115	.0152096	.0083881
25000	10	0	.5	-.0052408	.0322286	.0279951	.0260879	.0249041	.026014	.0263821	.0297086	.0251016
50000	20	0	.5	-.0061265	.0348609	.0322584	.0306132	.0222231	.0228475	.0230434	.0321649	.0292812
1500	3	0	.1	.6854142	.8966481	.8977152	.8975578	.1474457	.1942208	.2034371	.8977157	.8977656
2500	5	0	.1	.6875869	.9203757	.9192826	.9187987	.1810159	.2106052	.221216	.9313421	.9296121
3500	7	0	.1	.6707976	.9115657	.9115174	.9112428	.1609659	.1744765	.18196	.9281025	.9279457
5000	10	0	.1	.6533036	.9065241	.9065241	.9062943	.1408579	.1499673	.1556529	.9252464	.9252555
10000	20	0	.1	.6647638	.9165134	.9165134	.9164279	.1548106	.1575411	.1602405	.9425039	.9425039
3000	3	0	.1	.3695802	.5370179	.5339166	.5324364	.1696766	.1966078	.2093753	.5235524	.520043
5000	5	0	.1	.3655719	.5656383	.5633409	.5616363	.1611349	.1781558	.1859036	.5532593	.5499559
7000	7	0	.1	.3784308	.5840602	.5820477	.580398	.1658407	.1741722	.1786304	.5743355	.5719788
10000	10	0	.1	.376702	.5925664	.5914904	.5897679	.1566571	.1615485	.1643097	.5848585	.5835868
20000	20	0	.1	.3891864	.6170677	.6170677	.6164418	.1705862	.1713867	.1727635	.6116067	.6116067
7500	3	0	.1	.0286631	.1676149	.1555589	.1500242	.1566672	.1676563	.1756394	.1608483	.1486595
12500	5	0	.1	.0670171	.235823	.2248702	.2194627	.1634269	.1686219	.1718681	.2299924	.2184592
17500	7	0	.1	.0942784	.2763809	.2652494	.2604178	.1720319	.1762406	.1787046	.2721691	.2603301
25000	10	0	.1	.0885541	.2828036	.2787516	.273392	.1625621	.1644524	.1661467	.2786499	.2742772
50000	20	0	.1	.1086338	.3076649	.3064922	.3026988	.1679265	.1682932	.168715	.3037979	.3024179
1500	3	.822467	.5	.1478621	.3401932	.3047404	.296566	.2163691	.1761174	.1738818	.2778061	.2318339
2500	5	.822467	.5	.2226745	.4004216	.367133	.3603104	.282217	.2254473	.2219335	.3559077	.3106709
3500	7	.822467	.5	.3109793	.465279	.426347	.4176093	.3634789	.2822918	.2777093	.4335392	.3791752
5000	10	.822467	.5	.3083277	.4674799	.4278152	.419369	.3588465	.2745228	.2712937	.4392553	.3833093
10000	20	.822467	.5	.3303691	.4830281	.4533302	.4442472	.3797382	.2871456	.2843416	.4578603	.4120469
3000	3	.822467	.5	.2023497	.3185821	.2278359	.2118634	.259098	.1943267	.1912152	.3012736	.2028218
5000	5	.822467	.5	.243664	.3578995	.2461492	.2340765	.2788096	.1784321	.1757005	.3448782	.2240359
7000	7	.822467	.5	.2989727	.4116837	.2924723	.2824452	.3246152	.2170518	.2147377	.4033054	.2744138
10000	10	.822467	.5	.3551041	.4613751	.3305847	.3225378	.3750673	.2421067	.2402659	.4547101	.3128508
20000	20	.822467	.5	.3432413	.4515818	.3196152	.3155353	.3606504	.2151643	.2151355	.444276	.298236
7500	3	.822467	.5	.2031568	.2897868	.1262488	.108165	.2573367	.1774029	.1738596	.2883716	.1213823
12500	5	.822467	.5	.2679689	.3582907	.1556902	.1419145	.2987559	.1678417	.1652101	.3561699	.1500808
17500	7	.822467	.5	.2931527	.3877536	.1710348	.161818	.3149724	.1696594	.1683036	.3862947	.1656756
25000	10	.822467	.5	.3375922	.4298484	.1807963	.1756624	.3526916	.176805	.176515	.4285995	.1752872
50000	20	.822467	.5	.3705488	.4584264	.194857	.193467	.3795857	.1807804	.1810723	.4574589	.1884877
1500	3	.822467	.1	.7666386	.9796424	.9738149	.9725942	.3281831	.3612433	.3683001	.9836237	.9766205
2500	5	.822467	.1	.8253827	1.067171	1.059916	1.057547	.4564114	.4746704	.4877566	1.091225	1.080569
3500	7	.822467	.1	.816775	1.052871	1.047877	1.045767	.4127892	.4185593	.4258014	1.080275	1.073779
5000	10	.822467	.1	.828593	1.080622	1.07586	1.071469	.4394876	.4330602	.4362677	1.114682	1.107599
10000	20	.822467	.1	.829242	1.086935	1.085862	1.082119	.4420325	.4353164	.4346136	1.130767	1.128432
3000	3	.822467	.1	.5632324	.756152	.7199819	.7127446	.4338694	.4456134	.4577462	.7462332	.7080918
5000	5	.822467	.1	.5425654	.767928	.7411098	.7346237	.3724228	.3697141	.3774926	.7618017	.7327114
7000	7	.822467	.1	.5819244	.8302183	.7968862	.7894445	.4031735	.3907171	.3944942	.8296033	.7934878
10000	10	.822467	.1	.6095112	.8768575	.8410231	.8312664	.4199151	.4021154	.4033565	.8801431	.8398186

Create PDF in your applications with the Pdfcrowd HTML to PDF API PDFCROWD

20000	20	.822467	.1	.637924	.9189028	.8904232	.8782215	.436412	.4163294	.4154028	.9251449	.8926908
7500	3	.822467	.1	.2909051	.4953376	.3828715	.363571	.4214369	.3982649	.4045731	.4886579	.37338
12500	5	.822467	.1	.3483956	.609799	.4660316	.4491686	.4109353	.3935664	.3984322	.6068768	.4602938
17500	7	.822467	.1	.4101415	.7014446	.536509	.5218184	.4468324	.4183994	.4206843	.7004845	.5310745
25000	10	.822467	.1	.3953445	.7004725	.5362568	.5234023	.4121715	.3853211	.386785	.6994162	.5314455
50000	20	.822467	.1	.4429903	.7718189	.5748796	.5664268	.4278621	.3947905	.3947442	.7717794	.5701252
1500	3	3.289868	.5	.6508071	.7716495	.6066365	.5938709	.6567295	.4093017	.3942198	.7390115	.5543507
2500	5	3.289868	.5	.9314959	.957059	.7239623	.7136669	.9349366	.5441479	.5248398	.9568951	.6939851
3500	7	3.289868	.5	.9785493	.9731075	.7297779	.7212532	1.004702	.5473426	.5325042	.9830516	.7044174
5000	10	3.289868	.5	1.075799	1.015383	.7395768	.7326028	1.117408	.5536988	.543013	1.037821	.7159377
10000	20	3.289868	.5	1.190643	1.074926	.7545855	.7526537	1.259851	.5694955	.5668903	1.106498	.7344044
3000	3	3.289868	.5	.7496321	.8390393	.5028231	.4793167	.7263989	.4018745	.3826478	.8246875	.4772237
5000	5	3.289868	.5	.920093	.98109	.5409695	.5204859	.8934844	.4200514	.4038036	.9771582	.5220301
7000	7	3.289868	.5	1.033306	1.04738	.5676217	.5542886	1.007687	.440747	.4301409	1.052077	.5565821
10000	10	3.289868	.5	1.131538	1.102097	.575119	.5667058	1.116921	.4414834	.435129	1.108326	.5619654
20000	20	3.289868	.5	1.228425	1.158933	.5861945	.5839883	1.230787	.4482939	.4459442	1.16761	.5735632
7500	3	3.289868	.5	.6473259	.7785891	.2476549	.207797	.6621604	.2942986	.2805578	.7741661	.2392786
12500	5	3.289868	.5	.9177795	.9987598	.3748149	.3433348	.9010692	.3609343	.3525813	.9964337	.3675723
17500	7	3.289868	.5	1.00872	1.08286	.3602205	.3381366	.9832197	.3349301	.3279659	1.082593	.3546105
25000	10	3.289868	.5	1.137371	1.165532	.37693	.3641988	1.108699	.3480852	.3441067	1.166703	.3715294
50000	20	3.289868	.5	1.304622	1.251741	.3975717	.3937575	1.282994	.359964	.3582111	1.253341	.392094
1500	3	3.289868	.1	1.203973	1.434969	1.337708	1.329885	1.2958	1.099132	1.099557	1.457388	1.355227
2500	5	3.289868	.1	1.237528	1.490715	1.365165	1.359763	1.320165	1.06303	1.069063	1.531298	1.395571
3500	7	3.289868	.1	1.275402	1.543079	1.395292	1.388675	1.384964	1.041744	1.047872	1.595117	1.43513
5000	10	3.289868	.1	1.346243	1.624541	1.451421	1.440096	1.504073	1.102362	1.106024	1.691005	1.495741
10000	20	3.289868	.1	1.388982	1.657648	1.467563	1.451873	1.528005	1.064008	1.065249	1.73557	1.517527
3000	3	3.289868	.1	1.065614	1.309589	1.081283	1.063976	1.345505	1.096373	1.104045	1.305245	1.075719
5000	5	3.289868	.1	1.131044	1.44836	1.139709	1.12471	1.367735	1.032477	1.038857	1.45253	1.138326
7000	7	3.289868	.1	1.217467	1.564793	1.171177	1.158279	1.44517	1.034719	1.038732	1.574639	1.173508
10000	10	3.289868	.1	1.241028	1.604376	1.17772	1.167767	1.44488	1.005757	1.007668	1.623384	1.182483
20000	20	3.289868	.1	1.364233	1.746893	1.2162	1.21108	1.568334	1.037563	1.038991	1.770836	1.222514
7500	3	3.289868	.1	.790164	1.123506	.6684344	.6344032	1.22828	.9560025	.9602826	1.121694	.6627051
12500	5	3.289868	.1	.969107	1.387986	.7947047	.7587473	1.315673	.9741116	.9793355	1.388634	.7907923
17500	7	3.289868	.1	1.087664	1.5404	.8321233	.8031975	1.402516	.9810481	.9834091	1.542423	.829892
25000	10	3.289868	.1	1.151642	1.635494	.8424892	.8221003	1.456145	.9849808	.9863181	1.638542	.8399326
50000	20	3.289868	.1	1.286403	1.8122	.8101201	.8044922	1.54423	.9707007	.9715764	1.816848	.8090723
1500	3	29.60881	.5	2.48706	2.502923	1.877014	1.862154	2.114735	1.10389	1.031121	2.575122	1.924843
2500	5	29.60881	.5	2.932444	2.67952	1.909622	1.90142	2.691592	1.245531	1.121938	2.807763	1.991809
3500	7	29.60881	.5	3.114257	2.767768	1.922736	1.915342	2.921954	1.314138	1.200002	2.896182	2.006598
5000	10	29.60881	.5	3.377337	2.953893	1.993315	1.989548	3.208883	1.418324	1.32111	3.120059	2.097769
10000	20	29.60881	.5	3.479103	2.95688	1.942335	1.940102	3.36916	1.443935	1.391617	3.13276	2.052519
3000	3	29.60881	.5	2.462734	2.583075	1.680497	1.652313	2.074491	.9884175	.9225928	2.58083	1.679253
5000	5	29.60881	.5	2.994207	2.949772	1.736797	1.713216	2.66497	1.085721	.9906652	2.988599	1.780634
7000	7	29.60881	.5	3.23854	3.136304	1.76207	1.746254	2.939816	1.135853	1.053173	3.180494	1.796629
10000	10	29.60881	.5	3.506807	3.325713	1.856574	1.843237	3.226812	1.230906	1.156695	3.373592	1.898087
20000	20	29.60881	.5	3.595915	3.373207	1.751301	1.74545	3.37091	1.20062	1.160057	3.424314	1.789808
7500	3	29.60881	.5	2.422304	2.771259	1.390973	1.339853	2.089075	.8739993	.8292075	2.767594	1.393694
12500	5	29.60881	.5	3.037595	3.262328	1.494991	1.44722	2.678137	.9639963	.9005059	3.267464	1.500941
17500	7	29.60881	.5	3.200841	3.349225	1.420861	1.38406	2.890358	.9351231	.8874768	3.357718	1.426829
25000	10	29.60881	.5	3.447343	3.489537	1.460749	1.435076	3.15228	.9682839	.926137	3.497307	1.469397
50000	20	29.60881	.5	3.639019	3.614684	1.488581	1.478279	3.378233	.9912144	.9679043	3.624297	1.499848
1500	3	29.60881	.1	2.927743	3.16363	2.679165	2.666186	5.140833	3.308958	3.251256	3.236115	2.743285
2500	5	29.60881	.1	3.062716	3.222859	2.608369	2.598814	5.653003	3.218907	3.179723	3.311876	2.68454

Create PDF in your applications with the Pdfcrowd HTML to PDF API PDFCROWD

3500	7	29.60881	.1	3.273012	3.415056	2.649639	2.641997	6.245386	3.322227	3.290658	3.527461	2.738332
5000	10	29.60881	.1	3.358738	3.462969	2.642491	2.636757	6.558652	3.356002	3.332517	3.592664	2.746612
10000	20	29.60881	.1	3.5115	3.600779	2.636497	2.633167	7.033995	3.342876	3.331312	3.743448	2.740364
3000	3	29.60881	.1	2.768264	3.096567	2.373452	2.347538	4.900085	3.171339	3.136003	3.11366	2.392824
5000	5	29.60881	.1	3.113023	3.451026	2.356119	2.329908	5.738992	3.084731	3.058967	3.478611	2.385066
7000	7	29.60881	.1	3.257238	3.571153	2.378951	2.358977	6.087828	3.111357	3.0907	3.607362	2.415206
10000	10	29.60881	.1	3.436505	3.7831	2.402082	2.384913	6.518861	3.155646	3.141741	3.821663	2.439435
20000	20	29.60881	.1	3.594078	3.881563	2.419702	2.410451	6.98326	3.175378	3.168269	3.922399	2.459648
7500	3	29.60881	.1	2.558102	3.071069	1.964569	1.9186	4.727398	2.959006	2.928289	3.070481	1.965929
12500	5	29.60881	.1	3.027232	3.582505	2.032291	1.979193	5.63701	3.0075	2.993619	3.584471	2.038829
17500	7	29.60881	.1	3.3062	3.845054	2.071139	2.018329	6.181612	3.035812	3.025319	3.853458	2.080752
25000	10	29.60881	.1	3.520689	4.097488	2.135537	2.096752	6.664782	3.113466	3.105679	4.103111	2.145255
50000	20	29.60881	.1	3.607685	4.129018	2.101036	2.080617	6.939243	3.063957	3.060606	4.136329	2.112932

SE of mean bias

ssl	ssh	tsq1	cb	MH	MHfe	MHdl	MHbdl	P	Pdl	Pbdl	Pbdl	Pbdl
1500	3	0	.5	.0005974	.0005082	.0005057	.000505	.0005865	.0005866	.0005857	.0005713	.000569
2500	5	0	.5	.0004493	.0003719	.0003719	.0003716	.0004432	.000444	.0004424	.0004239	.0004231
3500	7	0	.5	.0003991	.0003142	.0003143	.000314	.000385	.000383	.000383	.000363	.0003632
5000	10	0	.5	.0003044	.0002521	.0002519	.000252	.0003039	.0003054	.0003056	.0002824	.0002822
10000	20	0	.5	.0002215	.0001811	.0001811	.0001811	.0002218	.0002224	.0002222	.0002034	.0002033
3000	3	0	.5	.0004598	.000412	.0004143	.0004159	.000399	.0004073	.0004106	.000434	.0004358
5000	5	0	.5	.0003569	.000323	.0003226	.0003228	.0003194	.0003222	.000324	.0003316	.0003312
7000	7	0	.5	.0002913	.0002633	.0002627	.0002629	.0002664	.0002684	.0002689	.0002723	.0002718
10000	10	0	.5	.0002388	.0002172	.0002162	.000216	.0002174	.0002179	.0002183	.0002242	.0002229
20000	20	0	.5	.000165	.0001481	.0001483	.0001486	.0001525	.0001525	.0001523	.0001515	.0001518
7500	3	0	.5	.0002936	.0002816	.0002912	.0002935	.0002564	.0002618	.0002638	.0002829	.000293
12500	5	0	.5	.0002283	.0002185	.0002208	.0002214	.0002013	.0002027	.0002033	.0002197	.0002222
17500	7	0	.5	.0001871	.0001803	.0001801	.0001804	.0001655	.0001665	.0001668	.0001808	.0001805
25000	10	0	.5	.0001598	.0001522	.0001535	.0001536	.0001421	.0001436	.0001437	.000153	.0001544
50000	20	0	.5	.0001108	.0001051	.000105	.0001051	.0000988	.0000989	.0000989	.0001059	.0001058
1500	3	0	.1	.0004722	.0003617	.000363	.0003628	.0006721	.0007594	.0007775	.000399	.0003984
2500	5	0	.1	.0003957	.0003169	.0003143	.0003127	.0005463	.0005958	.000607	.0003468	.0003434
3500	7	0	.1	.0003354	.0002654	.0002653	.0002644	.0004545	.0004706	.000476	.0002911	.0002908
5000	10	0	.1	.0002881	.0002217	.0002217	.0002212	.0003502	.0003625	.0003649	.000244	.000244
10000	20	0	.1	.0001985	.0001617	.0001617	.0001616	.0002567	.0002609	.000262	.000176	.000176
3000	3	0	.1	.0004242	.0004031	.0003975	.0003954	.0004803	.0005297	.0005555	.000424	.0004179
5000	5	0	.1	.0003454	.0003332	.0003283	.0003256	.0003639	.0003877	.0003997	.0003539	.0003482
7000	7	0	.1	.0002991	.0002892	.000285	.0002824	.0003096	.0003217	.0003245	.0003057	.0003007
10000	10	0	.1	.0002485	.0002438	.0002425	.0002403	.000261	.000266	.000267	.0002556	.000254
20000	20	0	.1	.0001796	.0001777	.0001777	.0001771	.0001848	.0001857	.0001864	.0001881	.0001881
7500	3	0	.1	.0004266	.0004213	.0004076	.0004029	.0002846	.0002957	.0003104	.0004283	.0004146
12500	5	0	.1	.0003389	.0003349	.0003231	.000319	.0002256	.0002318	.0002356	.0003405	.0003287
17500	7	0	.1	.0002749	.0002781	.0002689	.0002657	.0001897	.0001929	.0001943	.0002804	.0002707
25000	10	0	.1	.0002398	.0002353	.0002322	.0002292	.0001642	.0001663	.0001671	.0002385	.000235
50000	20	0	.1	.0001718	.0001706	.0001691	.0001666	.0001193	.0001197	.0001198	.0001726	.0001708

Create PDF in your applications with the Pdfcrowd HTML to PDF API PDFCROWD

1500	3	.822467	.5	.0007954	.0006549	.0006324	.0006284	.0007774	.0007263	.0007168	.0007476	.0007179
2500	5	.822467	.5	.0006197	.0005075	.000486	.0004824	.0006202	.0005797	.0005714	.0005761	.0005495
3500	7	.822467	.5	.0005195	.0004173	.0003908	.0003891	.0005303	.0004744	.0004699	.000466	.0004379
5000	10	.822467	.5	.0004446	.0003661	.000343	.0003406	.0004635	.0004117	.0004093	.0004109	.0003819
10000	20	.822467	.5	.0003217	.0002563	.0002394	.0002382	.0003405	.0002913	.0002909	.0002858	.0002641
3000	3	.822467	.5	.0007503	.0006826	.0006356	.0006316	.0006701	.0006082	.0006012	.000714	.0006654
5000	5	.822467	.5	.0005869	.000526	.0004808	.0004779	.0005457	.0004795	.0004753	.0005452	.0005015
7000	7	.822467	.5	.0004821	.0004263	.0003906	.0003893	.000462	.0003982	.0003963	.0004387	.0004035
10000	10	.822467	.5	.0004216	.000374	.0003263	.000326	.0004089	.0003312	.0003297	.0003846	.000337
20000	20	.822467	.5	.0003172	.0002693	.0002362	.0002372	.0003128	.0002471	.0002472	.0002778	.0002471
7500	3	.822467	.5	.0006841	.0006504	.0006162	.0006142	.000592	.0005255	.0005169	.0006528	.000621
12500	5	.822467	.5	.0005358	.0004966	.0004343	.0004352	.0004865	.0003928	.0003899	.0004998	.0004392
17500	7	.822467	.5	.0004532	.0004219	.0003614	.0003616	.0004233	.0003277	.0003263	.0004236	.0003642
25000	10	.822467	.5	.0003862	.0003499	.0003096	.0003102	.0003657	.0002795	.0002789	.0003517	.0003122
50000	20	.822467	.5	.0002758	.0002439	.0002129	.0002126	.000267	.0001933	.0001932	.000245	.0002152
1500	3	.822467	.1	.0005437	.0004626	.0004506	.0004484	.0008549	.0009103	.0009218	.0004951	.0004822
2500	5	.822467	.1	.0004636	.0004049	.0003923	.0003884	.0007298	.0007264	.0007343	.0004424	.0004258
3500	7	.822467	.1	.0003878	.0003494	.0003404	.0003371	.0005997	.0005961	.0005971	.0003728	.0003621
5000	10	.822467	.1	.0003435	.0003116	.0003031	.0002974	.0005217	.0005034	.0005037	.0003335	.0003221
10000	20	.822467	.1	.000245	.0002259	.0002238	.0002191	.0003854	.0003734	.0003722	.0002444	.0002406
3000	3	.822467	.1	.0005822	.0005941	.0005379	.000529	.0007466	.0007399	.0007588	.000613	.0005553
5000	5	.822467	.1	.000472	.0004815	.0004412	.0004341	.000581	.0005571	.0005627	.0004986	.0004569
7000	7	.822467	.1	.0004042	.0004258	.0003796	.0003716	.000494	.0004607	.0004602	.0004419	.0003936
10000	10	.822467	.1	.0003469	.0003753	.000322	.000314	.0004196	.0003774	.0003772	.0003899	.0003327
20000	20	.822467	.1	.0002601	.0002788	.0002465	.000238	.0003076	.0002793	.0002789	.0002884	.0002525
7500	3	.822467	.1	.0006777	.0007029	.0005874	.000573	.0006271	.000569	.0005749	.000714	.0005984
12500	5	.822467	.1	.0005208	.0005616	.000442	.0004298	.0004638	.000419	.0004199	.000569	.0004484
17500	7	.822467	.1	.0004686	.0005084	.0003746	.0003672	.0004382	.0003715	.0003717	.0005128	.0003784
25000	10	.822467	.1	.0003994	.0004373	.0003118	.0003076	.0003611	.0003095	.0003093	.0004403	.0003155
50000	20	.822467	.1	.000282	.0003121	.0002218	.0002199	.0002522	.0002136	.0002138	.0003146	.000224
1500	3	3.289868	.5	.0012222	.001006	.0008642	.0008593	.0011127	.0009148	.000896	.0011225	.0009809
2500	5	3.289868	.5	.0010268	.0008357	.0006826	.0006796	.0009444	.0007343	.0007159	.0009074	.0007506
3500	7	3.289868	.5	.0008822	.0006965	.0005652	.0005633	.0008385	.0006347	.0006209	.0007678	.0006311
5000	10	3.289868	.5	.0007948	.000586	.0004532	.0004534	.0007636	.0005226	.0005135	.0006419	.0005065
10000	20	3.289868	.5	.0005713	.0004139	.0003249	.0003258	.0005663	.000381	.0003781	.0004516	.0003674
3000	3	3.289868	.5	.0012689	.0011286	.0009382	.0009313	.0010652	.0008355	.0008133	.0011685	.0009784
5000	5	3.289868	.5	.001061	.0009169	.0007067	.0007015	.0009332	.0006533	.000637	.0009442	.0007409
7000	7	3.289868	.5	.0009254	.00078	.0005881	.0005859	.0008402	.0005517	.0005424	.0007969	.0006109
10000	10	3.289868	.5	.0007975	.0006317	.0004999	.000499	.0007425	.0004718	.0004664	.0006474	.0005227
20000	20	3.289868	.5	.0005919	.0004532	.0003652	.0003645	.0005681	.0003481	.0003456	.0004621	.0003816
7500	3	3.289868	.5	.001304	.0012344	.000997	.0009872	.0010478	.0007734	.0007596	.0012438	.0010079
12500	5	3.289868	.5	.0010679	.0009467	.0007566	.0007564	.0009139	.0006101	.0006016	.0009552	.0007714
17500	7	3.289868	.5	.000928	.0007902	.0006189	.0006188	.0008268	.000503	.0004963	.0007963	.00063
25000	10	3.289868	.5	.0007929	.000652	.0005165	.0005176	.0007267	.0004191	.0004154	.0006552	.0005246
50000	20	3.289868	.5	.0006021	.0004648	.0003744	.000374	.0005685	.0003043	.0003027	.0004662	.0003794
1500	3	3.289868	.1	.0009106	.0008882	.000722	.0007121	.0019285	.001518	.0015146	.0009294	.0007633
2500	5	3.289868	.1	.0007589	.0007582	.0005773	.0005702	.0016032	.0011385	.0011364	.0007878	.0005977
3500	7	3.289868	.1	.0006886	.0006794	.0004941	.0004881	.0014517	.0009272	.0009257	.0007043	.0005129
5000	10	3.289868	.1	.0006064	.0006012	.0004148	.0004096	.0012879	.0008082	.0008068	.0006277	.0004249
10000	20	3.289868	.1	.000454	.0004487	.0002804	.0002781	.000992	.000537	.000538	.0004696	.0002872
3000	3	3.289868	.1	.0010112	.001073	.0007876	.000772	.0018201	.0013774	.0013795	.0010959	.0008139
5000	5	3.289868	.1	.0008815	.0009443	.0006294	.0006144	.0016156	.0010693	.0010624	.0009604	.0006451
7000	7	3.289868	.1	.0007752	.0008486	.0005052	.0004954	.0014204	.0008561	.0008549	.00086	.0005164

Create PDF in your applications with the Pdfcrowd HTML to PDF API PDFCROWD

10000	10	3.289868	.1	.0006848	.0007411	.0004083	.0004033	.0012741	.0006952	.0006951	.0007555	.0004182
20000	20	3.289868	.1	.0005171	.0005449	.0002861	.0002856	.0009679	.0005088	.0005087	.0005538	.0002933
7500	3	3.289868	.1	.0011511	.0012587	.0008322	.0008089	.0017239	.0011543	.0011481	.0012625	.0008364
12500	5	3.289868	.1	.0009547	.0010408	.0006482	.0006265	.0014894	.0009192	.000917	.0010445	.0006556
17500	7	3.289868	.1	.0008378	.0009254	.0005397	.000527	.0013307	.0007706	.0007689	.0009296	.0005463
25000	10	3.289868	.1	.0007707	.000843	.0004571	.0004489	.0012217	.0006687	.0006684	.0008468	.000463
50000	20	3.289868	.1	.0005644	.0006008	.0003195	.0003168	.0009525	.0004438	.0004437	.0006028	.0003234
1500	3	29.60881	.5	.0022176	.0021697	.0018844	.0018863	.0018221	.0014013	.0013335	.0023521	.0020762
2500	5	29.60881	.5	.0016763	.0016614	.0014327	.0014327	.0014221	.0011602	.0010721	.001795	.0015884
3500	7	29.60881	.5	.0013809	.0014183	.0011923	.0011923	.0012043	.0009978	.0009453	.0015134	.0013185
5000	10	29.60881	.5	.0010364	.0012191	.0010733	.0010726	.0008448	.0008902	.000863	.0012822	.001169
10000	20	29.60881	.5	.0006379	.0008431	.0007356	.0007349	.0004905	.0006349	.000633	.000879	.0008062
3000	3	29.60881	.5	.0023556	.0024083	.0020448	.0020471	.0018319	.00136	.0012999	.0024857	.0021335
5000	5	29.60881	.5	.0018	.0019129	.001579	.0015779	.0014818	.0011251	.0010544	.0019664	.0016741
7000	7	29.60881	.5	.0014316	.001583	.0013675	.0013667	.001187	.0009707	.0009291	.001618	.0014333
10000	10	29.60881	.5	.0010385	.0012623	.0011406	.0011407	.0008327	.0008123	.0007894	.0012845	.0011907
20000	20	29.60881	.5	.0006336	.0008739	.0007886	.0007883	.0004808	.0005808	.0005776	.0008844	.0008177
7500	3	29.60881	.5	.0025674	.0027704	.0022791	.0022738	.0018889	.0013254	.0012781	.0027975	.002317
12500	5	29.60881	.5	.001828	.0019962	.0017383	.001742	.0014482	.0010528	.0010039	.0020138	.0017674
17500	7	29.60881	.5	.0014365	.0016247	.0014526	.0014588	.0011767	.0008877	.0008642	.0016356	.0014796
25000	10	29.60881	.5	.0010767	.0013426	.001209	.001213	.0008804	.000748	.0007328	.0013478	.0012244
50000	20	29.60881	.5	.0006714	.000911	.0008615	.0008626	.0005131	.000534	.0005315	.0009142	.0008755
1500	3	29.60881	.1	.0018194	.0018631	.0015753	.0015718	.0039812	.0027766	.0026982	.0019204	.0016334
2500	5	29.60881	.1	.0014529	.0014777	.0012118	.0012082	.0033481	.0021874	.0021393	.0015233	.0012611
3500	7	29.60881	.1	.0011559	.0012129	.0009823	.0009782	.0027977	.0018019	.0017691	.0012417	.0010188
5000	10	29.60881	.1	.0009699	.0010326	.000843	.000839	.0023919	.0015681	.0015463	.0010457	.0008865
10000	20	29.60881	.1	.0005953	.0006984	.0005937	.0005917	.0015099	.0010949	.0010868	.0007092	.0006188
3000	3	29.60881	.1	.001938	.0020677	.001658	.0016504	.0038539	.0026015	.0025457	.002097	.0016927
5000	5	29.60881	.1	.0015224	.0016597	.0012771	.0012693	.0032624	.0020151	.0019823	.0016703	.0013081
7000	7	29.60881	.1	.0013097	.001442	.0011153	.0011082	.002921	.0017746	.0017474	.0014528	.0011466
10000	10	29.60881	.1	.0010188	.0011563	.0009471	.0009428	.002386	.0015117	.0014975	.0011637	.0009666
20000	20	29.60881	.1	.0006486	.0007587	.0006878	.0006854	.0015813	.0010887	.0010835	.0007637	.0007021
7500	3	29.60881	.1	.002232	.0024676	.0019534	.0019413	.0039269	.0025994	.0025411	.0024779	.0019655
12500	5	29.60881	.1	.0017128	.0019124	.0014634	.0014491	.0033624	.0020075	.0019878	.0019213	.00148
17500	7	29.60881	.1	.0013106	.0014665	.0011751	.0011718	.0027968	.0016204	.0016086	.0014731	.0011831
25000	10	29.60881	.1	.0010049	.0011648	.0010473	.001046	.0022568	.001426	.001419	.0011661	.0010535
50000	20	29.60881	.1	.0006258	.0007382	.0007249	.000725	.0015057	.0009711	.0009687	.0007396	.0007301

Power

ssl	ssh	tsq1	cb	MH	MHfe	MHdl	MHbdl	P	Pdl	Pbdl	Pbdl	Pbdl
1500	3	0	.5	14.6	5.1	5.1	5.1	19	17.3	16.5	7	7
2500	5	0	.5	27.8	7.4	7	7	28.5	25.3	21.7	9.5	9.1
3500	7	0	.5	43.1	14.1	14.1	13.7	40.7	37.7	33.6	18.2	18.1
5000	10	0	.5	60.7	23.8	23.7	22.6	55.3	51.1	47.9	29.6	29.3
10000	20	0	.5	88.9	58.2	58.2	57.8	83.7	82.2	80.4	63.8	63.8
3000	3	0	.5	33.1	22.5	21.1	19.9	35.5	31.4	29.7	23.5	22.1

Create PDF in your applications with the Pdfcrowd HTML to PDF API PDFCROWD

5000	5	0	.5	52.8	38	35	32.4	52.6	46.5	42.8	39.5	36.2
7000	7	0	.5	68.2	51.8	49.2	46.9	67.1	60.8	57.9	52.9	49.9
10000	10	0	.5	84.1	68.9	67.2	65.7	83.3	78.7	77.2	70.2	68.2
20000	20	0	.5	98.7	95.3	95.1	94.7	98.3	97.9	97.8	95.5	95.2
7500	3	0	.5	68.9	64.1	55.5	51.3	69.5	59.6	56	64.2	55.3
12500	5	0	.5	89.6	86.8	80.4	77.3	89.6	83.5	80.3	86.8	80.2
17500	7	0	.5	97.1	95.9	94.3	92.9	97	95.3	94.4	95.9	94.3
25000	10	0	.5	99.4	98.8	98.2	97.9	99.3	98.5	98.2	98.8	98.2
50000	20	0	.5	100	100	100	100	100	100	100	100	100
1500	3	0	.1	0	0	0	0	0	0	0	0	0
2500	5	0	.1	0	0	0	0	.2	.2	.2	0	0
3500	7	0	.1	0	0	0	0	5.3	5.3	5.3	0	0
5000	10	0	.1	0	0	0	0	11.6	11.5	11.2	0	0
10000	20	0	.1	0	0	0	0	31.8	31.6	30.6	0	0
3000	3	0	.1	0	0	0	0	1.6	1.6	1.6	0	0
5000	5	0	.1	.1	0	0	0	10.5	10.5	10.3	0	0
7000	7	0	.1	.3	0	0	0	19.2	19.1	18.4	0	0
10000	10	0	.1	.9	0	0	0	31.7	31.4	30.4	0	0
20000	20	0	.1	7.5	.3	.3	.3	60.4	60	58.1	.4	.4
7500	3	0	.1	4.7	1	1	1	21.2	20.9	20.3	1.1	1.1
12500	5	0	.1	24.3	8.1	8.1	7.9	40.3	39.5	37.7	9	9
17500	7	0	.1	38.4	14.2	14.2	14.1	52.9	51.3	49	14.7	14.7
25000	10	0	.1	61.9	22.7	22.7	22.6	75.1	73.5	70.6	23.4	23.4
50000	20	0	.1	92.4	47.4	47.4	47.6	96.6	96	95.3	47.7	47.7
1500	3	.822467	.5	12.6	4.6	4.5	4.4	17	15.1	14.8	6.8	6.7
2500	5	.822467	.5	19.4	7.2	7.1	6.8	20.9	18.4	16.6	9	8.8
3500	7	.822467	.5	22	6.4	6	5.9	21.4	17	15.8	8.6	8.1
5000	10	.822467	.5	30.1	10.1	10.1	9.5	27.2	21.7	20.7	12.6	12.4
10000	20	.822467	.5	43.1	15.9	15.9	15.7	36.7	33.2	32.7	18.7	18.5
3000	3	.822467	.5	27.5	16.3	14.6	13.9	29.6	22.7	21.9	17.5	15.5
5000	5	.822467	.5	33.9	20	17	15.9	34.3	26.6	24.5	20.8	17.8
7000	7	.822467	.5	38.5	22.4	20.5	19	38.6	27.8	27	22.9	20.8
10000	10	.822467	.5	40.3	23.8	22.2	21.9	38.7	29.6	28.9	24.2	22.6
20000	20	.822467	.5	55.6	37.2	38	38	54.2	50.2	50.3	37.9	38.9
7500	3	.822467	.5	46.7	35.9	25.1	23.1	47.2	28.7	27.5	35.9	24.9
12500	5	.822467	.5	52.7	40.9	29.6	27.8	52.4	34.2	32.9	41	29.6
17500	7	.822467	.5	58.2	46.5	35.4	34.1	58.1	39.2	38.9	46.6	35.4
25000	10	.822467	.5	60.5	45.3	40.9	40.3	60.5	45.2	45.3	45.3	40.9
50000	20	.822467	.5	69	54	63.3	63.1	68.9	68.2	68.5	54.1	63.3
1500	3	.822467	.1	0	0	0	0	0	0	0	0	0
2500	5	.822467	.1	0	0	0	0	.1	.1	.1	0	0
3500	7	.822467	.1	0	0	0	0	4.5	4.5	4.5	0	0
5000	10	.822467	.1	0	0	0	0	6.1	6.1	6.1	0	0
10000	20	.822467	.1	.1	0	0	0	12.8	12.8	12.2	0	0
3000	3	.822467	.1	0	0	0	0	1.6	1.6	1.6	0	0
5000	5	.822467	.1	0	0	0	0	10.7	10.7	10.4	0	0
7000	7	.822467	.1	.1	0	0	0	12.3	11.9	11.5	0	0
10000	10	.822467	.1	1.1	0	0	0	15.3	14.7	14.2	.1	.1
20000	20	.822467	.1	2.4	0	0	0	24.8	23.8	22	0	0
7500	3	.822467	.1	5.8	1.3	1.3	1.3	19.8	19	18.7	1.8	1.8
12500	5	.822467	.1	15.1	4.4	4.4	4.4	25.8	23.3	21.6	4.8	4.8
17500	7	.822467	.1	19.9	6	6	6	28.6	25.7	24.7	6.3	6.3
25000	10	.822467	.1	28.5	7.5	7.4	7.2	39.4	33.3	30.9	7.7	7.6

Create PDF in your applications with the Pdfcrowd HTML to PDF API PDFCROWD

50000	20	.822467	.1	39.1	5.2	5.8	5.8	48.7	39.4	37.9	5.3	5.9
1500	3	3.289868	.5	12.3	4	3.9	3.7	15.6	13.3	12.8	5.8	5.7
2500	5	3.289868	.5	12.2	4.6	4.4	4.1	12.5	10.5	9.3	6	5.8
3500	7	3.289868	.5	11.5	3.4	3.4	3.2	11.3	9.3	8.6	4.1	4
5000	10	3.289868	.5	9.7	2.8	2.7	2.7	8.7	7.5	7.1	3.8	3.7
10000	20	3.289868	.5	5.2	1.6	1.9	1.9	4	5.2	5.4	2.2	2.4
3000	3	3.289868	.5	21.4	12.1	11	10.6	23	17.5	16.5	13.1	11.9
5000	5	3.289868	.5	19.9	11.1	9.4	8.3	19.9	13.9	12.9	11.4	9.5
7000	7	3.289868	.5	17.1	9.1	7.6	7.2	17.2	11.4	11.2	9.2	7.7
10000	10	3.289868	.5	14	7.2	6.2	6.3	13.8	10.2	10.2	7.4	6.2
20000	20	3.289868	.5	8.9	3.5	6.2	6.2	8.6	10.9	11.1	3.6	6.7
7500	3	3.289868	.5	35.8	27.6	19.1	17.5	36.4	24	22.6	27.7	19
12500	5	3.289868	.5	28.1	20.1	14.2	13.8	28.1	16.1	15.9	20.1	14.2
17500	7	3.289868	.5	24.6	15.9	14.5	13.6	24.5	16.7	16.6	16	14.5
25000	10	3.289868	.5	19.4	10	12.8	12.6	19.5	13.5	13.5	10.2	12.9
50000	20	3.289868	.5	9.4	4.1	15.9	15.9	9.3	17.9	17.8	4.1	16.1
1500	3	3.289868	.1	0	0	0	0	0	0	0	0	0
2500	5	3.289868	.1	0	0	0	0	.2	.2	.2	0	0
3500	7	3.289868	.1	0	0	0	0	1.5	1.5	1.5	0	0
5000	10	3.289868	.1	0	0	0	0	1.8	1.8	1.8	0	0
10000	20	3.289868	.1	0	0	0	0	1.6	1.5	1.3	0	0
3000	3	3.289868	.1	0	0	0	0	1.3	1.3	1.3	0	0
5000	5	3.289868	.1	.1	0	0	0	6.2	6.2	6.1	0	0
7000	7	3.289868	.1	0	0	0	0	6.3	6.2	5.9	0	0
10000	10	3.289868	.1	.4	0	0	0	6	5.7	5.5	0	0
20000	20	3.289868	.1	.3	0	0	0	4.1	3.8	3	0	0
7500	3	3.289868	.1	7.3	2	2	2	17.9	17.6	16.8	2.1	2.1
12500	5	3.289868	.1	11.8	3.6	3.6	3.6	17	14.1	12.7	3.7	3.7
17500	7	3.289868	.1	10.1	2.1	2	2	14.9	12.3	11.3	2.3	2.2
25000	10	3.289868	.1	9.8	2.6	2.6	2.6	14.2	10.1	9.6	2.6	2.6
50000	20	3.289868	.1	4.1	.2	.4	.4	5.4	2.8	2.9	.2	.4
1500	3	29.60881	.5	9.4	3.8	3.8	3.7	12.7	11.7	11.4	5.1	5.1
2500	5	29.60881	.5	5.7	2.2	2.2	2.2	5.9	4.9	4.5	3	3
3500	7	29.60881	.5	3.7	1.7	1.7	1.7	3.8	3.3	3.3	2.1	2
5000	10	29.60881	.5	.9	.5	.4	.4	.9	.8	.8	.5	.4
10000	20	29.60881	.5	.1	0	0	0	.1	.1	.1	.1	.1
3000	3	29.60881	.5	18.2	11.7	11.3	10.8	18.6	15.2	14.9	13	12.6
5000	5	29.60881	.5	8.6	5.7	5.8	5.4	8.8	6.9	7.1	5.8	5.9
7000	7	29.60881	.5	4.2	2.9	2.8	2.8	4.2	3.5	3.6	3.1	2.8
10000	10	29.60881	.5	.8	.5	.7	.7	.9	.9	.9	.6	.7
20000	20	29.60881	.5	.2	0	.1	.1	.2	.3	.3	0	.1
7500	3	29.60881	.5	22.2	18.1	14.2	13.9	22.4	16.8	16.2	18.1	14.2
12500	5	29.60881	.5	10.1	7.1	5.5	5.6	10.1	7	7	7.2	5.5
17500	7	29.60881	.5	4.6	3.1	3.6	3.4	4.7	3.7	3.6	3.1	3.6
25000	10	29.60881	.5	1.3	1.4	1.7	1.7	1.3	1.5	1.6	1.4	1.7
50000	20	29.60881	.5	.1	.1	.6	.6	.1	.9	.7	.1	.6
1500	3	29.60881	.1	0	0	0	0	0	0	0	0	0
2500	5	29.60881	.1	0	0	0	0	.2	.2	.2	0	0
3500	7	29.60881	.1	0	0	0	0	.4	.4	.4	0	0
5000	10	29.60881	.1	0	0	0	0	.6	.6	.6	0	0
10000	20	29.60881	.1	0	0	0	0	0	0	0	0	0
3000	3	29.60881	.1	0	0	0	0	.7	.7	.7	0	0
5000	5	29.60881	.1	0	0	0	0	3.8	3.8	3.7	0	0

Create PDF in your applications with the Pdfcrowd HTML to PDF API PDFCROWD

7000	7	29.60881	.1	0	0	0	0	2.7	2.7	2.7	0	0
10000	10	29.60881	.1	.2	0	0	0	.6	.5	.5	0	0
20000	20	29.60881	.1	0	0	0	0	.1	.1	.1	0	0
7500	3	29.60881	.1	7.4	1.6	1.6	1.6	16.8	16.3	16	1.7	1.7
12500	5	29.60881	.1	6.9	3.8	3.8	3.8	8.7	7.2	6.9	4.1	4.1
17500	7	29.60881	.1	2.5	1	1	1	3.6	2.4	2.4	1	1
25000	10	29.60881	.1	.7	.5	.5	.5	1	.6	.6	.5	.5
50000	20	29.60881	.1	.1	0	0	0	.1	0	0	0	0

SE of power

ssl	ssh	tsq1	cb	MH	MHfe	MHdl	MHbdl	P	Pdl	Pbdl	Pbdl	Pbdl
1500	3	0	.5	1.11662	.6956939	.6956939	.6956939	1.240564	1.196123	1.173776	.8068457	.8068457
2500	5	0	.5	1.416743	.8277922	.8068457	.8068457	1.427498	1.37474	1.303499	.927227	.9094999
3500	7	0	.5	1.566011	1.100541	1.100541	1.087341	1.553548	1.53255	1.493667	1.220148	1.217534
5000	10	0	.5	1.54451	1.346685	1.344734	1.322588	1.572231	1.580756	1.579744	1.443551	1.439274
10000	20	0	.5	.993373	1.559731	1.559731	1.561781	1.168037	1.209612	1.255325	1.519724	1.519724
3000	3	0	.5	1.488083	1.320511	1.290267	1.262533	1.513192	1.467665	1.44496	1.340802	1.312094
5000	5	0	.5	1.578658	1.534927	1.50831	1.479946	1.579	1.57726	1.56466	1.545882	1.519724
7000	7	0	.5	1.472671	1.580114	1.580936	1.578097	1.485796	1.543813	1.561278	1.578477	1.581136
10000	10	0	.5	1.156369	1.463827	1.484641	1.50117	1.179453	1.294724	1.32671	1.446361	1.472671
20000	20	0	.5	.3582039	.6692608	.6826346	.7084561	.4087909	.4534203	.4638534	.6555532	.6759882
7500	3	0	.5	1.463827	1.516967	1.571544	1.580604	1.455936	1.551722	1.569713	1.516034	1.572231
12500	5	0	.5	.9653186	1.070402	1.255325	1.324655	.9653186	1.173776	1.25774	1.070402	1.260143
17500	7	0	.5	.5306505	.6270486	.7331507	.8121515	.5394442	.6692608	.7270763	.6270486	.7331507
25000	10	0	.5	.244213	.3443254	.4204284	.4534203	.2636475	.3843826	.4204284	.3443254	.4204284
50000	20	0	.5	0	0	0	0	0	0	0	0	0
1500	3	0	.1	0	0	0	0	0	0	0	0	0
2500	5	0	.1	0	0	0	0	.1412799	.1412799	.1412799	0	0
3500	7	0	.1	0	0	0	0	.7084561	.7084561	.7084561	0	0
5000	10	0	.1	0	0	0	0	1.01264	1.008836	.9972763	0	0
10000	20	0	.1	0	0	0	0	1.472671	1.470184	1.457271	0	0
3000	3	0	.1	0	0	0	0	.3967871	.3967871	.3967871	0	0
5000	5	0	.1	.09995	0	0	0	.969407	.969407	.9612024	0	0
7000	7	0	.1	.1729451	0	0	0	1.245536	1.243057	1.225333	0	0
10000	10	0	.1	.2986469	0	0	0	1.471431	1.467665	1.454593	0	0
20000	20	0	.1	.8329166	.1729451	.1729451	.1729451	1.546557	1.549193	1.560253	.1995996	.1995996
7500	3	0	.1	.6692608	.3146427	.3146427	.3146427	1.292501	1.285764	1.271971	.3298333	.3298333
12500	5	0	.1	1.356285	.8627804	.8627804	.8529889	1.5511	1.545882	1.53255	.9049862	.9049862
17500	7	0	.1	1.537999	1.103793	1.103793	1.100541	1.578477	1.580604	1.580823	1.119781	1.119781
25000	10	0	.1	1.535705	1.324655	1.324655	1.322588	1.367476	1.395618	1.440708	1.33882	1.33882
50000	20	0	.1	.8379976	1.579	1.579	1.579316	.5730969	.6196773	.6692608	1.579465	1.579465
1500	3	.822467	.5	1.0494	.66245	.6555532	.6485677	1.187855	1.13225	1.122925	.7960904	.790639
2500	5	.822467	.5	1.250456	.8174105	.8121515	.7960904	1.285764	1.225333	1.176622	.9049862	.8958571
3500	7	.822467	.5	1.309962	.7739767	.7509993	.7451107	1.296935	1.187855	1.153412	.886589	.8627804
5000	10	.822467	.5	1.450514	.9528851	.9528851	.927227	1.407182	1.303499	1.281214	1.0494	1.042228
10000	20	.822467	.5	1.566011	1.156369	1.156369	1.150439	1.524175	1.489215	1.483479	1.233009	1.227905

Create PDF in your applications with the Pdfcrowd HTML to PDF API PDFCROWD

3000	3	.822467	.5	1.412002	1.168037	1.11662	1.093979	1.443551	1.324655	1.307819	1.201561	1.144443
5000	5	.822467	.5	1.496927	1.264911	1.187855	1.156369	1.50117	1.397297	1.360055	1.283495	1.209612
7000	7	.822467	.5	1.538749	1.318423	1.276617	1.240564	1.539493	1.416743	1.403923	1.328755	1.283495
10000	10	.822467	.5	1.5511	1.346685	1.314215	1.307819	1.540231	1.443551	1.433454	1.354385	1.322588
20000	20	.822467	.5	1.571191	1.52845	1.534927	1.534927	1.575551	1.581126	1.58111	1.534141	1.541684
7500	3	.822467	.5	1.577691	1.516967	1.371127	1.332813	1.578658	1.430493	1.412002	1.516967	1.367476
12500	5	.822467	.5	1.578832	1.554731	1.443551	1.416743	1.579316	1.50012	1.485796	1.555313	1.443551
17500	7	.822467	.5	1.559731	1.57726	1.51223	1.499063	1.560253	1.543813	1.541684	1.577479	1.51223
25000	10	.822467	.5	1.545882	1.574138	1.554731	1.5511	1.545882	1.573836	1.574138	1.574138	1.554731
50000	20	.822467	.5	1.462532	1.576071	1.524175	1.525906	1.463827	1.472671	1.468928	1.575814	1.524175
1500	3	.822467	.1	0	0	0	0	0	0	0	0	0
2500	5	.822467	.1	0	0	0	0	.09995	.09995	.09995	0	0
3500	7	.822467	.1	0	0	0	0	.6555532	.6555532	.6555532	0	0
5000	10	.822467	.1	0	0	0	0	.7568289	.7568289	.7568289	0	0
10000	20	.822467	.1	.09995	0	0	0	1.056485	1.056485	1.034969	0	0
3000	3	.822467	.1	0	0	0	0	.3967871	.3967871	.3967871	0	0
5000	5	.822467	.1	0	0	0	0	.9775019	.9775019	.9653186	0	0
7000	7	.822467	.1	.09995	0	0	0	1.03861	1.023909	1.008836	0	0
10000	10	.822467	.1	.3298333	0	0	0	1.13838	1.119781	1.103793	.09995	.09995
20000	20	.822467	.1	.4839835	0	0	0	1.365635	1.346685	1.309962	0	0
7500	3	.822467	.1	.7391617	.3582039	.3582039	.3582039	1.260143	1.240564	1.233009	.4204284	.4204284
12500	5	.822467	.1	1.13225	.6485677	.6485677	.6485677	1.383604	1.336828	1.301322	.6759882	.6759882
17500	7	.822467	.1	1.262533	.7509993	.7509993	.7509993	1.429	1.38185	1.363785	.7683163	.7683163
25000	10	.822467	.1	1.427498	.8329166	.8277922	.8174105	1.545199	1.490339	1.461229	.8430362	.8379976
50000	20	.822467	.1	1.54311	.7021111	.7391617	.7391617	1.580604	1.545199	1.534141	.7084561	.7451107
1500	3	3.289868	.5	1.03861	.6196773	.6122009	.5969171	1.147449	1.07383	1.056485	.7391617	.7331507
2500	5	3.289868	.5	1.034969	.66245	.6485677	.6270486	1.045825	.969407	.918428	.7509993	.7391617
3500	7	3.289868	.5	1.008836	.5730969	.5730969	.5565609	1.001154	.918428	.886589	.6270486	.6196773
5000	10	3.289868	.5	.9359006	.5216896	.5125524	.5125524	.8912407	.8329166	.8121515	.6046156	.5969171
10000	20	3.289868	.5	.7021111	.3967871	.4317291	.4317291	.6196773	.7021111	.7147307	.4638534	.4839835
3000	3	3.289868	.5	1.296935	1.031305	.9894443	.973468	1.330789	1.201561	1.173776	1.066954	1.023909
5000	5	3.289868	.5	1.262533	.993373	.9228434	.8724162	1.262533	1.093979	1.059995	1.005007	.927227
7000	7	3.289868	.5	1.190626	.9094999	.8379976	.8174105	1.193382	1.005007	.9972763	.9139803	.8430362
10000	10	3.289868	.5	1.097269	.8174105	.7626008	.7683163	1.09067	.957058	.957058	.8277922	.7626008
20000	20	3.289868	.5	.9004388	.5811626	.7626008	.7626008	.886589	.9854897	.993373	.589101	.790639
7500	3	3.289868	.5	1.516034	1.413591	1.243057	1.201561	1.521526	1.350555	1.322588	1.415171	1.240564
12500	5	3.289868	.5	1.421404	1.267277	1.103793	1.09067	1.421404	1.162235	1.156369	1.267277	1.103793
17500	7	3.289868	.5	1.361925	1.156369	1.113441	1.083993	1.360055	1.179453	1.176622	1.15931	1.113441
25000	10	3.289868	.5	1.250456	.9486833	1.056485	1.0494	1.252897	1.080625	1.080625	.957058	1.059995
50000	20	3.289868	.5	.9228434	.6270486	1.156369	1.156369	.918428	1.212266	1.209612	.6270486	1.162235
1500	3	3.289868	.1	0	0	0	0	0	0	0	0	0
2500	5	3.289868	.1	0	0	0	0	.1412799	.1412799	.1412799	0	0
3500	7	3.289868	.1	0	0	0	0	.3843826	.3843826	.3843826	0	0
5000	10	3.289868	.1	0	0	0	0	.4204284	.4204284	.4204284	0	0
10000	20	3.289868	.1	0	0	0	0	.3967871	.3843826	.3582039	0	0
3000	3	3.289868	.1	0	0	0	0	.3582039	.3582039	.3582039	0	0
5000	5	3.289868	.1	.09995	0	0	0	.7626008	.7626008	.7568289	0	0
7000	7	3.289868	.1	0	0	0	0	.7683163	.7626008	.7451107	0	0
10000	10	3.289868	.1	.1995996	0	0	0	.7509993	.7331507	.7209369	0	0
20000	20	3.289868	.1	.1729451	0	0	0	.6270486	.6046156	.5394442	0	0
7500	3	3.289868	.1	.8226239	.4427189	.4427189	.4427189	1.212266	1.204259	1.182269	.4534203	.4534203
12500	5	3.289868	.1	1.020176	.589101	.589101	.589101	1.187855	1.100541	1.052953	.5969171	.5969171
17500	7	3.289868	.1	.9528851	.4534203	.4427189	.4427189	1.126051	1.03861	1.001154	.4740359	.4638534

Create PDF in your applications with the Pdfcrowd HTML to PDF API PDFCROWD

25000	10	3.289868	.1	.9401915	.5032296	.5032296	.5032296	1.103793	.9528851	.9315793	.5032296	.5032296
50000	20	3.289868	.1	.6270486	.1412799	.1995996	.1995996	.7147307	.5216896	.5306505	.1412799	.1995996
1500	3	29.60881	.5	.9228434	.6046156	.6046156	.5969171	1.052953	1.01642	1.005007	.6956939	.6956939
2500	5	29.60881	.5	.7331507	.4638534	.4638534	.4638534	.7451107	.6826346	.6555532	.5394442	.5394442
3500	7	29.60881	.5	.5969171	.4087909	.4087909	.4087909	.6046156	.5648982	.5648982	.4534203	.4427189
5000	10	29.60881	.5	.2986469	.2230471	.1995996	.1995996	.2986469	.2817091	.2817091	.2230471	.1995996
10000	20	29.60881	.5	.09995	0	0	0	.09995	.09995	.09995	.09995	.09995
3000	3	29.60881	.5	1.220148	1.01642	1.001154	.981509	1.230463	1.135324	1.126051	1.063485	1.0494
5000	5	29.60881	.5	.886589	.7331507	.7391617	.7147307	.8958571	.8014924	.8121515	.7391617	.7451107
7000	7	29.60881	.5	.6343185	.5306505	.5216896	.5216896	.6343185	.5811626	.589101	.5480785	.5216896
10000	10	29.60881	.5	.2817091	.2230471	.2636475	.2636475	.2986469	.2986469	.2986469	.244213	.2636475
20000	20	29.60881	.5	.1412799	0	.09995	.09995	.1412799	.1729451	.1729451	0	.09995
7500	3	29.60881	.5	1.314215	1.217534	1.103793	1.093979	1.318423	1.182269	1.165144	1.217534	1.103793
12500	5	29.60881	.5	.9528851	.8121515	.7209369	.7270763	.9528851	.8068457	.8068457	.8174105	.7209369
17500	7	29.60881	.5	.66245	.5480785	.589101	.5730969	.6692608	.5969171	.589101	.5480785	.589101
25000	10	29.60881	.5	.3582039	.3715373	.4087909	.4087909	.3582039	.3843826	.3967871	.3715373	.4087909
50000	20	29.60881	.5	.09995	.09995	.244213	.244213	.09995	.2986469	.2636475	.09995	.244213
1500	3	29.60881	.1	0	0	0	0	0	0	0	0	0
2500	5	29.60881	.1	0	0	0	0	.1412799	.1412799	.1412799	0	0
3500	7	29.60881	.1	0	0	0	0	.1995996	.1995996	.1995996	0	0
5000	10	29.60881	.1	0	0	0	0	.244213	.244213	.244213	0	0
10000	20	29.60881	.1	0	0	0	0	0	0	0	0	0
3000	3	29.60881	.1	0	0	0	0	.2636475	.2636475	.2636475	0	0
5000	5	29.60881	.1	0	0	0	0	.6046156	.6046156	.5969171	0	0
7000	7	29.60881	.1	0	0	0	0	.5125524	.5125524	.5125524	0	0
10000	10	29.60881	.1	.1412799	0	0	0	.244213	.2230471	.2230471	0	0
20000	20	29.60881	.1	0	0	0	0	.09995	.09995	.09995	0	0
7500	3	29.60881	.1	.8277922	.3967871	.3967871	.3967871	1.182269	1.168037	1.15931	.4087909	.4087909
12500	5	29.60881	.1	.8014924	.6046156	.6046156	.6046156	.8912407	.8174105	.8014924	.6270486	.6270486
17500	7	29.60881	.1	.4937104	.3146427	.3146427	.3146427	.589101	.4839835	.4839835	.3146427	.3146427
25000	10	29.60881	.1	.2636475	.2230471	.2230471	.2230471	.3146427	.244213	.244213	.2230471	.2230471
50000	20	29.60881	.1	.09995	0	0	0	.09995	0	0	0	0

MH: Mantel-Haenszel weighting (fixed-effect)
MHfe: Mantel-Haenszel with fixed-effect weighting (different to MH weighting)
MHdl: Mantel-Haenszel with DL random-effects weighting
MHbdl: Mantel-Haenszel with bootstrapped DL random-effects weighting
P: Peto OR with fixed-effect weighting
Pdl: Peto OR with DL random-effects weighting
Pbdl: Peto OR with bootstrapped DL random-effects weighting

ssl: size for lower level unit (patients)
ssh: size for higher level unit (studies)
tsq1: between study variance for the exposure
cb: probability of membership for the intervention (0.5=balanced design)

Create PDF in your applications with the Pdfcrowd HTML to PDF API PDFCROWD

```
effsize: ln(0.5)
I^2:     0% (tau^2=0), 20% (tau^2=0.822467), 50% (tau^2=3.289868), 90% (tau^2=29.60881)
```

Create PDF in your applications with the Pdfcrowd HTML to PDF API PDFCROWD
