# Supplementary material for: Applications of simple and accessible methods for meta-analysis involving rare events: A simulation study
Source: Stat Methods Med Res. 2021 Jun 17;30(7):1589–608. doi: 10.1177/09622802211022385 (PMC8411477; doi:10.1177/09622802211022385)
Supplement: sj-pdf-3-smm-10.1177_09622802211022385 - Supplemental material for Applications of simple and accessible methods for meta-analysis involving rare events: A simulation study [file sj-pdf-3-smm-10.1177_09622802211022385.pdf]

Very rare events scenarios: 1% vs 0.50251%, OR=0.5

Convergence (successful MA %)

ssl	ssh	tsq1	cb	MH	MHfe	MHdl	MHbdl	P	Pdl	Pbdl	Pbdl	Pbdl
1500	3	0	.5	100	100	100	100	100	100	100	100	100
2500	5	0	.5	100	100	100	100	100	100	100	100	100
3500	7	0	.5	100	100	100	100	100	100	100	100	100
5000	10	0	.5	100	100	100	100	100	100	100	100	100
10000	20	0	.5	100	100	100	100	100	100	100	100	100
3000	3	0	.5	100	100	100	100	100	100	100	100	100
5000	5	0	.5	100	100	100	100	100	100	100	100	100
7000	7	0	.5	100	100	100	100	100	100	100	100	100
10000	10	0	.5	100	100	100	100	100	100	100	100	100
20000	20	0	.5	100	100	100	100	100	100	100	100	100
7500	3	0	.5	100	100	100	100	100	100	100	100	100
12500	5	0	.5	100	100	100	100	100	100	100	100	100
17500	7	0	.5	100	100	100	100	100	100	100	100	100
25000	10	0	.5	100	100	100	100	100	100	100	100	100
50000	20	0	.5	100	100	100	100	100	100	100	100	100
1500	3	0	.1	100	100	100	100	100	100	100	100	100
2500	5	0	.1	100	100	100	100	100	100	100	100	100
3500	7	0	.1	100	100	100	100	100	100	100	100	100
5000	10	0	.1	100	100	100	100	100	100	100	100	100
10000	20	0	.1	100	100	100	100	100	100	100	100	100
3000	3	0	.1	100	100	100	100	100	100	100	100	100
5000	5	0	.1	100	100	100	100	100	100	100	100	100
7000	7	0	.1	100	100	100	100	100	100	100	100	100
10000	10	0	.1	100	100	100	100	100	100	100	100	100
20000	20	0	.1	100	100	100	100	100	100	100	100	100
7500	3	0	.1	100	100	100	100	100	100	100	100	100
12500	5	0	.1	100	100	100	100	100	100	100	100	100
17500	7	0	.1	100	100	100	100	100	100	100	100	100
25000	10	0	.1	100	100	100	100	100	100	100	100	100
50000	20	0	.1	100	100	100	100	100	100	100	100	100
1500	3	.822467	.5	100	100	100	100	100	100	100	100	100
2500	5	.822467	.5	100	100	100	100	100	100	100	100	100
3500	7	.822467	.5	100	100	100	100	100	100	100	100	100
5000	10	.822467	.5	100	100	100	100	100	100	100	100	100
10000	20	.822467	.5	100	100	100	100	100	100	100	100	100
3000	3	.822467	.5	100	100	100	100	100	100	100	100	100
5000	5	.822467	.5	100	100	100	100	100	100	100	100	100
7000	7	.822467	.5	100	100	100	100	100	100	100	100	100
10000	10	.822467	.5	100	100	100	100	100	100	100	100	100

Create PDF in your applications with the Pdfcrowd HTML to PDF API PDFCROWD

20000	20	.822467	.5	100	100	100	100	100	100	100	100	100
7500	3	.822467	.5	100	100	100	100	100	100	100	100	100
12500	5	.822467	.5	100	100	100	100	100	100	100	100	100
17500	7	.822467	.5	100	100	100	100	100	100	100	100	100
25000	10	.822467	.5	100	100	100	100	100	100	100	100	100
50000	20	.822467	.5	100	100	100	100	100	100	100	100	100
1500	3	.822467	.1	100	100	100	100	100	100	100	100	100
2500	5	.822467	.1	100	100	100	100	100	100	100	100	100
3500	7	.822467	.1	100	100	100	100	100	100	100	100	100
5000	10	.822467	.1	100	100	100	100	100	100	100	100	100
10000	20	.822467	.1	100	100	100	100	100	100	100	100	100
3000	3	.822467	.1	100	100	100	100	100	100	100	100	100
5000	5	.822467	.1	100	100	100	100	100	100	100	100	100
7000	7	.822467	.1	100	100	100	100	100	100	100	100	100
10000	10	.822467	.1	100	100	100	100	100	100	100	100	100
20000	20	.822467	.1	100	100	100	100	100	100	100	100	100
7500	3	.822467	.1	100	100	100	100	100	100	100	100	100
12500	5	.822467	.1	100	100	100	100	100	100	100	100	100
17500	7	.822467	.1	100	100	100	100	100	100	100	100	100
25000	10	.822467	.1	100	100	100	100	100	100	100	100	100
50000	20	.822467	.1	100	100	100	100	100	100	100	100	100
1500	3	3.289868	.5	100	100	100	100	100	100	100	100	100
2500	5	3.289868	.5	100	100	100	100	100	100	100	100	100
3500	7	3.289868	.5	100	100	100	100	100	100	100	100	100
5000	10	3.289868	.5	100	100	100	100	100	100	100	100	100
10000	20	3.289868	.5	100	100	100	100	100	100	100	100	100
3000	3	3.289868	.5	100	100	100	100	100	100	100	100	100
5000	5	3.289868	.5	100	100	100	100	100	100	100	100	100
7000	7	3.289868	.5	100	100	100	100	100	100	100	100	100
10000	10	3.289868	.5	100	100	100	100	100	100	100	100	100
20000	20	3.289868	.5	100	100	100	100	100	100	100	100	100
7500	3	3.289868	.5	100	100	100	100	100	100	100	100	100
12500	5	3.289868	.5	100	100	100	100	100	100	100	100	100
17500	7	3.289868	.5	100	100	100	100	100	100	100	100	100
25000	10	3.289868	.5	100	100	100	100	100	100	100	100	100
50000	20	3.289868	.5	100	100	100	100	100	100	100	100	100
1500	3	3.289868	.1	100	100	100	100	100	100	100	100	100
2500	5	3.289868	.1	100	100	100	100	100	100	100	100	100
3500	7	3.289868	.1	100	100	100	100	100	100	100	100	100
5000	10	3.289868	.1	100	100	100	100	100	100	100	100	100
10000	20	3.289868	.1	100	100	100	100	100	100	100	100	100
3000	3	3.289868	.1	100	100	100	100	100	100	100	100	100
5000	5	3.289868	.1	100	100	100	100	100	100	100	100	100
7000	7	3.289868	.1	100	100	100	100	100	100	100	100	100
10000	10	3.289868	.1	100	100	100	100	100	100	100	100	100
20000	20	3.289868	.1	100	100	100	100	100	100	100	100	100
7500	3	3.289868	.1	100	100	100	100	100	100	100	100	100
12500	5	3.289868	.1	100	100	100	100	100	100	100	100	100
17500	7	3.289868	.1	100	100	100	100	100	100	100	100	100
25000	10	3.289868	.1	100	100	100	100	100	100	100	100	100
50000	20	3.289868	.1	100	100	100	100	100	100	100	100	100
1500	3	29.60881	.5	100	100	100	100	100	100	100	100	100
2500	5	29.60881	.5	100	100	100	100	100	100	100	100	100

Create PDF in your applications with the Pdfcrowd HTML to PDF API PDFCROWD

3500	7	29.60881	.5	100	100	100	100	100	100	100	100	100
5000	10	29.60881	.5	100	100	100	100	100	100	100	100	100
10000	20	29.60881	.5	100	100	100	100	100	100	100	100	100
3000	3	29.60881	.5	100	100	100	100	100	100	100	100	100
5000	5	29.60881	.5	100	100	100	100	100	100	100	100	100
7000	7	29.60881	.5	100	100	100	100	100	100	100	100	100
10000	10	29.60881	.5	100	100	100	100	100	100	100	100	100
20000	20	29.60881	.5	100	100	100	100	100	100	100	100	100
7500	3	29.60881	.5	100	100	100	100	100	100	100	100	100
12500	5	29.60881	.5	100	100	100	100	100	100	100	100	100
17500	7	29.60881	.5	100	100	100	100	100	100	100	100	100
25000	10	29.60881	.5	100	100	100	100	100	100	100	100	100
50000	20	29.60881	.5	100	100	100	100	100	100	100	100	100
1500	3	29.60881	.1	100	100	100	100	100	100	100	100	100
2500	5	29.60881	.1	100	100	100	100	100	100	100	100	100
3500	7	29.60881	.1	100	100	100	100	100	100	100	100	100
5000	10	29.60881	.1	100	100	100	100	100	100	100	100	100
10000	20	29.60881	.1	100	100	100	100	100	100	100	100	100
3000	3	29.60881	.1	100	100	100	100	100	100	100	100	100
5000	5	29.60881	.1	100	100	100	100	100	100	100	100	100
7000	7	29.60881	.1	100	100	100	100	100	100	100	100	100
10000	10	29.60881	.1	100	100	100	100	100	100	100	100	100
20000	20	29.60881	.1	100	100	100	100	100	100	100	100	100
7500	3	29.60881	.1	100	100	100	100	100	100	100	100	100
12500	5	29.60881	.1	100	100	100	100	100	100	100	100	100
17500	7	29.60881	.1	100	100	100	100	100	100	100	100	100
25000	10	29.60881	.1	100	100	100	100	100	100	100	100	100
50000	20	29.60881	.1	100	100	100	100	100	100	100	100	100

SE of convergence

ssl	ssh	tsq1	cb	MH	MHfe	MHdl	MHbdl	P	Pdl	Pbdl	Pbdl	Pbdl
1500	3	0	.5	0	0	0	0	0	0	0	0	0
2500	5	0	.5	0	0	0	0	0	0	0	0	0
3500	7	0	.5	0	0	0	0	0	0	0	0	0
5000	10	0	.5	0	0	0	0	0	0	0	0	0
10000	20	0	.5	0	0	0	0	0	0	0	0	0
3000	3	0	.5	0	0	0	0	0	0	0	0	0
5000	5	0	.5	0	0	0	0	0	0	0	0	0
7000	7	0	.5	0	0	0	0	0	0	0	0	0
10000	10	0	.5	0	0	0	0	0	0	0	0	0
20000	20	0	.5	0	0	0	0	0	0	0	0	0
7500	3	0	.5	0	0	0	0	0	0	0	0	0
12500	5	0	.5	0	0	0	0	0	0	0	0	0
17500	7	0	.5	0	0	0	0	0	0	0	0	0
25000	10	0	.5	0	0	0	0	0	0	0	0	0
50000	20	0	.5	0	0	0	0	0	0	0	0	0

Create PDF in your applications with the Pdfcrowd HTML to PDF API PDFCROWD

1500	3	0	.1	0	0	0	0	0	0	0	0	0
2500	5	0	.1	0	0	0	0	0	0	0	0	0
3500	7	0	.1	0	0	0	0	0	0	0	0	0
5000	10	0	.1	0	0	0	0	0	0	0	0	0
10000	20	0	.1	0	0	0	0	0	0	0	0	0
3000	3	0	.1	0	0	0	0	0	0	0	0	0
5000	5	0	.1	0	0	0	0	0	0	0	0	0
7000	7	0	.1	0	0	0	0	0	0	0	0	0
10000	10	0	.1	0	0	0	0	0	0	0	0	0
20000	20	0	.1	0	0	0	0	0	0	0	0	0
7500	3	0	.1	0	0	0	0	0	0	0	0	0
12500	5	0	.1	0	0	0	0	0	0	0	0	0
17500	7	0	.1	0	0	0	0	0	0	0	0	0
25000	10	0	.1	0	0	0	0	0	0	0	0	0
50000	20	0	.1	0	0	0	0	0	0	0	0	0
1500	3	.822467	.5	0	0	0	0	0	0	0	0	0
2500	5	.822467	.5	0	0	0	0	0	0	0	0	0
3500	7	.822467	.5	0	0	0	0	0	0	0	0	0
5000	10	.822467	.5	0	0	0	0	0	0	0	0	0
10000	20	.822467	.5	0	0	0	0	0	0	0	0	0
3000	3	.822467	.5	0	0	0	0	0	0	0	0	0
5000	5	.822467	.5	0	0	0	0	0	0	0	0	0
7000	7	.822467	.5	0	0	0	0	0	0	0	0	0
10000	10	.822467	.5	0	0	0	0	0	0	0	0	0
20000	20	.822467	.5	0	0	0	0	0	0	0	0	0
7500	3	.822467	.5	0	0	0	0	0	0	0	0	0
12500	5	.822467	.5	0	0	0	0	0	0	0	0	0
17500	7	.822467	.5	0	0	0	0	0	0	0	0	0
25000	10	.822467	.5	0	0	0	0	0	0	0	0	0
50000	20	.822467	.5	0	0	0	0	0	0	0	0	0
1500	3	.822467	.1	0	0	0	0	0	0	0	0	0
2500	5	.822467	.1	0	0	0	0	0	0	0	0	0
3500	7	.822467	.1	0	0	0	0	0	0	0	0	0
5000	10	.822467	.1	0	0	0	0	0	0	0	0	0
10000	20	.822467	.1	0	0	0	0	0	0	0	0	0
3000	3	.822467	.1	0	0	0	0	0	0	0	0	0
5000	5	.822467	.1	0	0	0	0	0	0	0	0	0
7000	7	.822467	.1	0	0	0	0	0	0	0	0	0
10000	10	.822467	.1	0	0	0	0	0	0	0	0	0
20000	20	.822467	.1	0	0	0	0	0	0	0	0	0
7500	3	.822467	.1	0	0	0	0	0	0	0	0	0
12500	5	.822467	.1	0	0	0	0	0	0	0	0	0
17500	7	.822467	.1	0	0	0	0	0	0	0	0	0
25000	10	.822467	.1	0	0	0	0	0	0	0	0	0
50000	20	.822467	.1	0	0	0	0	0	0	0	0	0
1500	3	3.289868	.5	0	0	0	0	0	0	0	0	0
2500	5	3.289868	.5	0	0	0	0	0	0	0	0	0
3500	7	3.289868	.5	0	0	0	0	0	0	0	0	0
5000	10	3.289868	.5	0	0	0	0	0	0	0	0	0
10000	20	3.289868	.5	0	0	0	0	0	0	0	0	0
3000	3	3.289868	.5	0	0	0	0	0	0	0	0	0
5000	5	3.289868	.5	0	0	0	0	0	0	0	0	0
7000	7	3.289868	.5	0	0	0	0	0	0	0	0	0

Create PDF in your applications with the Pdfcrowd HTML to PDF API PDFCROWD

10000	10	3.289868	.5	0	0	0	0	0	0	0	0	0
20000	20	3.289868	.5	0	0	0	0	0	0	0	0	0
7500	3	3.289868	.5	0	0	0	0	0	0	0	0	0
12500	5	3.289868	.5	0	0	0	0	0	0	0	0	0
17500	7	3.289868	.5	0	0	0	0	0	0	0	0	0
25000	10	3.289868	.5	0	0	0	0	0	0	0	0	0
50000	20	3.289868	.5	0	0	0	0	0	0	0	0	0
1500	3	3.289868	.1	0	0	0	0	0	0	0	0	0
2500	5	3.289868	.1	0	0	0	0	0	0	0	0	0
3500	7	3.289868	.1	0	0	0	0	0	0	0	0	0
5000	10	3.289868	.1	0	0	0	0	0	0	0	0	0
10000	20	3.289868	.1	0	0	0	0	0	0	0	0	0
3000	3	3.289868	.1	0	0	0	0	0	0	0	0	0
5000	5	3.289868	.1	0	0	0	0	0	0	0	0	0
7000	7	3.289868	.1	0	0	0	0	0	0	0	0	0
10000	10	3.289868	.1	0	0	0	0	0	0	0	0	0
20000	20	3.289868	.1	0	0	0	0	0	0	0	0	0
7500	3	3.289868	.1	0	0	0	0	0	0	0	0	0
12500	5	3.289868	.1	0	0	0	0	0	0	0	0	0
17500	7	3.289868	.1	0	0	0	0	0	0	0	0	0
25000	10	3.289868	.1	0	0	0	0	0	0	0	0	0
50000	20	3.289868	.1	0	0	0	0	0	0	0	0	0
1500	3	29.60881	.5	0	0	0	0	0	0	0	0	0
2500	5	29.60881	.5	0	0	0	0	0	0	0	0	0
3500	7	29.60881	.5	0	0	0	0	0	0	0	0	0
5000	10	29.60881	.5	0	0	0	0	0	0	0	0	0
10000	20	29.60881	.5	0	0	0	0	0	0	0	0	0
3000	3	29.60881	.5	0	0	0	0	0	0	0	0	0
5000	5	29.60881	.5	0	0	0	0	0	0	0	0	0
7000	7	29.60881	.5	0	0	0	0	0	0	0	0	0
10000	10	29.60881	.5	0	0	0	0	0	0	0	0	0
20000	20	29.60881	.5	0	0	0	0	0	0	0	0	0
7500	3	29.60881	.5	0	0	0	0	0	0	0	0	0
12500	5	29.60881	.5	0	0	0	0	0	0	0	0	0
17500	7	29.60881	.5	0	0	0	0	0	0	0	0	0
25000	10	29.60881	.5	0	0	0	0	0	0	0	0	0
50000	20	29.60881	.5	0	0	0	0	0	0	0	0	0
1500	3	29.60881	.1	0	0	0	0	0	0	0	0	0
2500	5	29.60881	.1	0	0	0	0	0	0	0	0	0
3500	7	29.60881	.1	0	0	0	0	0	0	0	0	0
5000	10	29.60881	.1	0	0	0	0	0	0	0	0	0
10000	20	29.60881	.1	0	0	0	0	0	0	0	0	0
3000	3	29.60881	.1	0	0	0	0	0	0	0	0	0
5000	5	29.60881	.1	0	0	0	0	0	0	0	0	0
7000	7	29.60881	.1	0	0	0	0	0	0	0	0	0
10000	10	29.60881	.1	0	0	0	0	0	0	0	0	0
20000	20	29.60881	.1	0	0	0	0	0	0	0	0	0
7500	3	29.60881	.1	0	0	0	0	0	0	0	0	0
12500	5	29.60881	.1	0	0	0	0	0	0	0	0	0
17500	7	29.60881	.1	0	0	0	0	0	0	0	0	0
25000	10	29.60881	.1	0	0	0	0	0	0	0	0	0
50000	20	29.60881	.1	0	0	0	0	0	0	0	0	0

Create PDF in your applications with the Pdfcrowd HTML to PDF API PDFCROWD

Coverage

ssl	ssh	tsq1	cb	MH	MHfe	MHdl	MHbdl	P	Pdl	Pbdl	Pbdl	Pbdl
1500	3	0	.5	95.7	96.2	97.2	97.7	96.5	97.4	97.6	96.2	97.2
2500	5	0	.5	94.9	95.1	95.9	96.4	95.2	96	96.3	95.1	95.9
3500	7	0	.5	95.5	95.7	96.3	96.7	95.3	96.1	96.6	95.7	96.3
5000	10	0	.5	95.3	95.4	97	97.3	95.3	96.8	97.2	95.4	97
10000	20	0	.5	94.2	94.6	95.6	96.2	94.6	95.4	96.2	94.6	95.6
3000	3	0	.5	94.3	94.7	96.3	96.7	95.4	96.4	97	94.7	96.3
5000	5	0	.5	95.2	95.3	97	97.5	95	96.7	96.9	95.3	97
7000	7	0	.5	95.3	95.2	96.2	96.6	94.2	95.3	95.9	95.2	96.2
10000	10	0	.5	94.9	95.1	96.4	96.8	94.7	95.7	96.6	95.1	96.4
20000	20	0	.5	94	93.4	94.7	95.6	93	94.1	94.3	93.4	94.7
7500	3	0	.5	94.8	94.8	96	97	94.5	96	96.9	94.8	96
12500	5	0	.5	96.8	96.5	97.7	98.5	96.2	97.2	97.4	96.5	97.7
17500	7	0	.5	95.1	95	95.6	96.6	95	95.9	96.5	95	95.6
25000	10	0	.5	95.9	96	96.8	97.1	94.7	95.6	96.1	96	96.8
50000	20	0	.5	96.1	96	96.7	96.9	91.5	92.7	93.8	96	96.7
1500	3	0	.1	96.6	95.3	97.3	97.6	96.3	97.1	97.6	95.3	97.3
2500	5	0	.1	95.5	93.2	95.5	96.3	95.7	96.2	96.2	93.2	95.5
3500	7	0	.1	96.4	92.1	94.8	96	94.4	95.7	96.3	92.1	94.8
5000	10	0	.1	94.9	89.5	92	93.1	91.6	92.2	92.4	89.5	92
10000	20	0	.1	94.5	82.8	84.9	87.2	82.5	82.9	83.2	82.8	84.9
3000	3	0	.1	95.8	94.8	96.2	96.8	95.3	96.2	96.7	94.8	96.2
5000	5	0	.1	94.4	92.8	95.4	95.7	89.2	90.9	91.8	92.8	95.4
7000	7	0	.1	95.5	93	95.3	96	86.9	88.4	89.2	93	95.3
10000	10	0	.1	94.3	91.8	94	94.8	81.4	82.6	84.1	91.8	94
20000	20	0	.1	96	88.8	91.4	92.9	58.9	59.6	60.2	88.8	91.4
7500	3	0	.1	95.5	94.8	96.3	96.9	85.9	88.4	89.7	94.8	96.3
12500	5	0	.1	95	94.9	96.6	97	76.1	77.8	79.4	94.9	96.6
17500	7	0	.1	95.1	94.7	96.6	96.7	63.2	65.9	68.3	94.7	96.6
25000	10	0	.1	95.7	94	96	96.6	46.9	47.8	50.1	94	96
50000	20	0	.1	95.7	92.9	94.6	95.5	12	12.3	12.6	92.9	94.6
1500	3	.822467	.5	46.7	49.4	82.6	84.6	49	81	83.9	49.4	82.6
2500	5	.822467	.5	43	42.3	86.7	87.9	44.2	85.7	86.1	42.3	86.7
3500	7	.822467	.5	38.5	37.6	89.3	90.1	39.5	89.2	89.4	37.6	89.3
5000	10	.822467	.5	34.4	30.8	90.3	90.3	34.5	90.1	90.1	30.8	90.3
10000	20	.822467	.5	24.9	16.5	88.7	88.9	23.5	88.2	88.4	16.5	88.8
3000	3	.822467	.5	34.1	35.2	80.4	82.4	36.2	78.7	80.7	35.2	80.4
5000	5	.822467	.5	31.1	30.9	85.4	86.6	31.7	85.5	85.9	30.9	85.4
7000	7	.822467	.5	29.3	28.5	89.7	89.8	30	89.5	89.8	28.5	89.7
10000	10	.822467	.5	24.6	21	90.5	90.7	24.4	90.2	90.4	21	90.5
20000	20	.822467	.5	17.9	13.5	90	90.6	18.3	88.2	88.3	13.5	90
7500	3	.822467	.5	22.4	22.9	78.2	81	22.8	77.3	80	22.9	78.2
12500	5	.822467	.5	18.4	19.6	85.9	87.4	19.1	86.1	86.6	19.6	85.9
17500	7	.822467	.5	20.3	18	88.7	89.1	21	89.4	89.5	18	88.7
25000	10	.822467	.5	15.9	13.4	89	89.2	16.1	89.3	89.4	13.4	89

Create PDF in your applications with the Pdfcrowd HTML to PDF API PDFCROWD

50000	20	.822467	.5	10.1	6.6	92.1	92.3	9.8	91.1	90.9	6.6	92.1
1500	3	.822467	.1	73.3	66.4	86.8	90.1	74.4	91	91.8	66.4	86.8
2500	5	.822467	.1	62.3	49	83.3	85.6	62.1	87.3	87.9	49	83.3
3500	7	.822467	.1	61.2	43.4	79.9	83	58	83.6	84.3	43.4	79.9
5000	10	.822467	.1	52.7	29.9	75.8	77.6	47	78.6	79.1	29.9	75.8
10000	20	.822467	.1	36	11.7	61.6	63.2	24.5	54.1	54	11.7	61.6
3000	3	.822467	.1	56.2	51.6	84.9	88.6	60.4	89.1	90.7	51.6	84.9
5000	5	.822467	.1	52.7	42.6	84.2	87.1	52.6	85.8	86.8	42.6	84.2
7000	7	.822467	.1	51.3	35.2	84.7	86.6	44.4	82	82.7	35.2	84.7
10000	10	.822467	.1	39.5	22.9	82.6	84.1	30.5	73.3	73.3	22.9	82.6
20000	20	.822467	.1	29.6	7	79.7	80.2	14.5	50.5	50.3	7	79.7
7500	3	.822467	.1	38.6	38.2	79.8	82.9	46.4	82.9	84.4	38.2	79.8
12500	5	.822467	.1	36.9	29.6	85.4	86.5	36.2	82.9	84.4	29.6	85.4
17500	7	.822467	.1	35	23	87.3	88.2	27.8	80.9	81.3	23	87.3
25000	10	.822467	.1	28.3	19.1	87.6	89.2	20.7	72.5	72.7	19.1	87.6
50000	20	.822467	.1	17.7	5.5	86.6	87.7	6.8	44.8	44.7	5.5	86.6
1500	3	3.289868	.5	22.9	25.4	79.2	82.8	24.1	77.1	78.8	25.4	79.2
2500	5	3.289868	.5	16.8	17.1	84.1	86.1	17.9	82.9	84.5	17.1	84.1
3500	7	3.289868	.5	14.3	12.6	84.5	85.8	14.7	84.4	85.6	12.6	84.5
5000	10	3.289868	.5	11.1	9.6	84.9	86	11.6	86.7	87.1	9.6	85
10000	20	3.289868	.5	3	1.5	81.4	82.4	3.4	82.1	82.2	1.5	81.4
3000	3	3.289868	.5	16.3	17	77.2	81.4	16.7	76.3	79.5	17	77.2
5000	5	3.289868	.5	14.1	12	82.9	85.9	14.9	83.9	84.8	12	82.9
7000	7	3.289868	.5	10.2	9.7	86.5	87.7	10.6	87.3	87.9	9.7	86.5
10000	10	3.289868	.5	6.8	5.6	85.2	86.2	7.2	86.3	86.7	5.6	85.2
20000	20	3.289868	.5	2.6	1.2	85.6	86.3	2.7	84.1	84	1.2	85.6
7500	3	3.289868	.5	11.3	12.7	77.6	82.2	11.5	77	78.8	12.7	77.6
12500	5	3.289868	.5	7.3	8.5	81.9	84.4	7.9	82.7	84.3	8.5	81.9
17500	7	3.289868	.5	6.3	7.1	85.1	86.9	6.1	87.2	88.3	7.1	85.1
25000	10	3.289868	.5	5.3	2.6	87.6	88.2	5.4	88.1	88.8	2.6	87.6
50000	20	3.289868	.5	1.3	.5	88.1	88.4	1.4	85.7	85.6	.5	88.1
1500	3	3.289868	.1	44.5	38.9	83.3	87.4	48.9	89.6	91.5	38.9	83.3
2500	5	3.289868	.1	34.1	24.2	76.8	81.4	35.6	85.7	86.3	24.2	76.8
3500	7	3.289868	.1	26.2	13.4	75.5	79	26.7	82.8	83.4	13.4	75.5
5000	10	3.289868	.1	17.6	7.1	73.6	75.8	15.7	73.4	73.2	7.1	73.6
10000	20	3.289868	.1	4.4	.8	58.6	60.1	3.3	44.9	44.5	.8	58.6
3000	3	3.289868	.1	29.5	26.3	75.4	82	38.4	84.1	85.7	26.3	75.4
5000	5	3.289868	.1	25.3	18.4	80.6	84.8	29.4	84.2	85	18.4	80.6
7000	7	3.289868	.1	20.4	10.9	80.8	84.3	20.7	82.1	82.6	10.9	80.8
10000	10	3.289868	.1	14.1	5.9	79.2	81.6	13	75.5	75.7	5.9	79.2
20000	20	3.289868	.1	5.5	.3	68.9	70.1	3	43.9	43.4	.3	68.9
7500	3	3.289868	.1	21.4	19.9	79.1	84.2	28.8	81.8	83.8	19.9	79.1
12500	5	3.289868	.1	14	10.1	80.7	83.9	16.6	81	82.4	10.1	80.7
17500	7	3.289868	.1	11.5	6.6	81.4	83.9	12	80.1	80.5	6.6	81.4
25000	10	3.289868	.1	11.1	2.8	81.7	83.3	8.8	71.5	71.7	2.8	81.7
50000	20	3.289868	.1	3.4	.1	82.3	82.7	1.2	44.4	44.2	.1	82.3
1500	3	29.60881	.5	8	14.4	74.8	81	8.1	71	76.9	14.4	74.8
2500	5	29.60881	.5	5.9	7.9	77.4	82.1	5.9	78.2	80.5	7.9	77.4
3500	7	29.60881	.5	2.6	5.6	70.7	76.7	3.2	74.2	76.5	5.6	70.7
5000	10	29.60881	.5	1.6	3	65.7	68.9	1.8	68	70.7	3	65.7
10000	20	29.60881	.5	.1	.1	51.9	53.6	.1	55.3	55.8	.1	51.9
3000	3	29.60881	.5	6	8.8	70.3	79.1	6.2	67.3	74.3	8.8	70.3
5000	5	29.60881	.5	3.9	6.1	72.6	80.5	4.2	74.4	78.2	6.1	72.6

Create PDF in your applications with the Pdfcrowd HTML to PDF API PDFCROWD

7000	7	29.60881	.5	2.3	3.3	69.3	75.7	2.6	73	76.2	3.3	69.3
10000	10	29.60881	.5	.8	1	65.4	69.8	.8	69.2	71.4	1	65.4
20000	20	29.60881	.5	.1	.2	54.2	56.3	0	54.5	56	.2	54.2
7500	3	29.60881	.5	3.7	6.3	71.8	81.6	3.9	70.4	75.5	6.3	71.8
12500	5	29.60881	.5	2.1	3.5	71.5	80.6	2.1	74.5	77.7	3.5	71.5
17500	7	29.60881	.5	1.4	2.2	71.8	77.9	1	76.9	78.6	2.2	71.8
25000	10	29.60881	.5	.4	1.6	67.7	72.8	.6	73	74.1	1.6	67.7
50000	20	29.60881	.5	0	0	57.9	59.8	.1	56.1	57.7	0	57.9
1500	3	29.60881	.1	17.5	24.1	78.1	84.8	25.6	82.8	85.2	24.1	78.1
2500	5	29.60881	.1	11.1	13	71.1	77.2	13.4	74.3	74.7	13	71.1
3500	7	29.60881	.1	4.4	7.9	61.9	67.7	5.5	64.1	64.7	7.9	61.9
5000	10	29.60881	.1	2	2.9	51.7	57	2.4	49.9	50.2	2.9	51.7
10000	20	29.60881	.1	0	0	27.8	30	0	16.2	15.8	0	27.8
3000	3	29.60881	.1	10.2	14.8	71.5	80	14.7	73.9	77.3	14.8	71.5
5000	5	29.60881	.1	7.6	9.9	70.8	78.6	9	71.4	72.7	9.9	70.8
7000	7	29.60881	.1	4.8	6	63.2	70.7	5.2	64.1	64.8	6	63.2
10000	10	29.60881	.1	1.8	2.6	55.9	63.4	2.1	50.3	50.5	2.6	55.9
20000	20	29.60881	.1	.5	.1	33.3	36.4	.4	16	15.6	.1	33.3
7500	3	29.60881	.1	7	13.1	71.9	81.3	10.3	70.9	75.1	13.1	71.9
12500	5	29.60881	.1	4.3	7.4	69.3	80	5.7	69.7	71	7.4	69.3
17500	7	29.60881	.1	1.9	2.9	66.3	74.8	2.8	66	66.7	2.9	66.3
25000	10	29.60881	.1	1.1	1.2	56.6	62.7	1	49.3	49.3	1.2	56.6
50000	20	29.60881	.1	.1	0	41	44.1	0	13.7	13.3	0	41

SE of coverage

ssl	ssh	tsq1	cb	MH	MHfe	MHdl	MHbdl	P	Pdl	Pbdl	Pbdl	Pbdl
1500	3	0	.5	.6414905	.6046156	.5216896	.4740359	.5811626	.5032296	.4839835	.6046156	.5216896
2500	5	0	.5	.6956939	.6826346	.6270486	.589101	.6759882	.6196773	.5969171	.6826346	.6270486
3500	7	0	.5	.6555532	.6414905	.5969171	.5648982	.6692608	.6122009	.5730969	.6414905	.5969171
5000	10	0	.5	.6692608	.66245	.5394442	.5125524	.6692608	.5565609	.5216896	.66245	.5394442
10000	20	0	.5	.7391617	.7147307	.6485677	.6046156	.7147307	.66245	.6046156	.7147307	.6485677
3000	3	0	.5	.7331507	.7084561	.5969171	.5648982	.66245	.589101	.5394442	.7084561	.5969171
5000	5	0	.5	.6759882	.6692608	.5394442	.4937104	.6892024	.5648982	.5480785	.6692608	.5394442
7000	7	0	.5	.6692608	.6759882	.6046156	.5730969	.7391617	.6692608	.6270486	.6759882	.6046156
10000	10	0	.5	.6956939	.6826346	.589101	.5565609	.7084561	.6414905	.5730969	.6826346	.589101
20000	20	0	.5	.7509993	.7851369	.7084561	.6485677	.8068457	.7451107	.7331507	.7851369	.7084561
7500	3	0	.5	.7021111	.7021111	.6196773	.5394442	.7209369	.6196773	.5480785	.7021111	.6196773
12500	5	0	.5	.5565609	.5811626	.4740359	.3843826	.6046156	.5216896	.5032296	.5811626	.4740359
17500	7	0	.5	.6826346	.6892024	.6485677	.5730969	.6892024	.6270486	.5811626	.6892024	.6485677
25000	10	0	.5	.6270486	.6196773	.5565609	.5306505	.7084561	.6485677	.6122009	.6196773	.5565609
50000	20	0	.5	.6122009	.6196773	.5648982	.5480785	.8819014	.8226239	.7626008	.6196773	.5648982
1500	3	0	.1	.5730969	.6692608	.5125524	.4839835	.5969171	.5306505	.4839835	.6692608	.5125524
2500	5	0	.1	.6555532	.7960904	.6555532	.5969171	.6414905	.6046156	.6046156	.7960904	.6555532
3500	7	0	.1	.589101	.8529889	.7021111	.6196773	.7270763	.6414905	.5969171	.8529889	.7021111
5000	10	0	.1	.6956939	.969407	.8579044	.8014924	.8771773	.848033	.8379976	.969407	.8579044
10000	20	0	.1	.7209369	1.193382	1.13225	1.056485	1.201561	1.190626	1.182269	1.193382	1.13225

Create PDF in your applications with the Pdfcrowd HTML to PDF API PDFCROWD

3000	3	0	.1	.6343185	.7021111	.6046156	.5565609	.6692608	.6046156	.5648982	.7021111	.6046156
5000	5	0	.1	.7270763	.8174105	.66245	.6414905	.981509	.9094999	.8676174	.8174105	.66245
7000	7	0	.1	.6555532	.8068457	.6692608	.6196773	1.066954	1.01264	.981509	.8068457	.6692608
10000	10	0	.1	.7331507	.8676174	.7509993	.7021111	1.230463	1.198849	1.156369	.8676174	.7509993
20000	20	0	.1	.6196773	.9972763	.886589	.8121515	1.555889	1.551722	1.547889	.9972763	.886589
7500	3	0	.1	.6555532	.7021111	.5969171	.5480785	1.100541	1.01264	.9612024	.7021111	.5969171
12500	5	0	.1	.6892024	.6956939	.5730969	.5394442	1.348625	1.314215	1.278921	.6956939	.5730969
17500	7	0	.1	.6826346	.7084561	.5730969	.5648982	1.525044	1.499063	1.471431	.7084561	.5730969
25000	10	0	.1	.6414905	.7509993	.6196773	.5730969	1.578097	1.579608	1.581136	.7509993	.6196773
50000	20	0	.1	.6414905	.8121515	.7147307	.6555532	1.027619	1.03861	1.0494	.8121515	.7147307
1500	3	.822467	.5	1.577691	1.581025	1.198849	1.14142	1.580823	1.240564	1.162235	1.581025	1.198849
2500	5	.822467	.5	1.565567	1.562277	1.07383	1.031305	1.570465	1.107028	1.093979	1.562277	1.07383
3500	7	.822467	.5	1.538749	1.531744	.9775019	.9444522	1.545882	.981509	.973468	1.531744	.9775019
5000	10	.822467	.5	1.502212	1.459918	.9359006	.9359006	1.503246	.9444522	.9444522	1.459918	.9359006
10000	20	.822467	.5	1.367476	1.173776	1.001154	.993373	1.340802	1.020176	1.01264	1.173776	.9972763
3000	3	.822467	.5	1.499063	1.510285	1.255325	1.204259	1.519724	1.294724	1.248002	1.510285	1.255325
5000	5	.822467	.5	1.463827	1.461229	1.11662	1.077237	1.471431	1.113441	1.100541	1.461229	1.11662
7000	7	.822467	.5	1.439274	1.427498	.9612024	.957058	1.449138	.969407	.957058	1.427498	.9612024
10000	10	.822467	.5	1.361925	1.288022	.927227	.918428	1.358175	.9401915	.9315793	1.288022	.927227
20000	20	.822467	.5	1.212266	1.080625	.9486833	.9228434	1.222747	1.020176	1.01642	1.080625	.9486833
7500	3	.822467	.5	1.318423	1.328755	1.305665	1.240564	1.32671	1.324655	1.264911	1.328755	1.305665
12500	5	.822467	.5	1.225333	1.255325	1.100541	1.0494	1.243057	1.093979	1.077237	1.255325	1.100541
17500	7	.822467	.5	1.271971	1.214907	1.001154	.9854897	1.288022	.973468	.969407	1.214907	1.001154
25000	10	.822467	.5	1.156369	1.077237	.9894443	.981509	1.162235	.9775019	.973468	1.077237	.9894443
50000	20	.822467	.5	.9528851	.7851369	.8529889	.8430362	.9401915	.9004388	.9094999	.7851369	.8529889
1500	3	.822467	.1	1.398967	1.493667	1.070402	.9444522	1.380087	.9049862	.8676174	1.493667	1.070402
2500	5	.822467	.1	1.53255	1.580823	1.179453	1.110243	1.534141	1.052953	1.031305	1.580823	1.179453
3500	7	.822467	.1	1.540961	1.567303	1.267277	1.187855	1.560769	1.170914	1.150439	1.567303	1.267277
5000	10	.822467	.1	1.578832	1.447753	1.354385	1.318423	1.57829	1.296935	1.285764	1.447753	1.354385
10000	20	.822467	.1	1.517893	1.01642	1.537999	1.525044	1.360055	1.575814	1.576071	1.01642	1.537999
3000	3	.822467	.1	1.568936	1.580329	1.13225	1.005007	1.546557	.9854897	.918428	1.580329	1.13225
5000	5	.822467	.1	1.578832	1.563726	1.153412	1.059995	1.579	1.103793	1.070402	1.563726	1.153412
7000	7	.822467	.1	1.580604	1.510285	1.13838	1.077237	1.571191	1.214907	1.196123	1.510285	1.13838
10000	10	.822467	.1	1.545882	1.328755	1.198849	1.156369	1.455936	1.398967	1.398967	1.328755	1.198849
20000	20	.822467	.1	1.443551	.8068457	1.271971	1.260143	1.113441	1.58106	1.58111	.8068457	1.271971
7500	3	.822467	.1	1.539493	1.536476	1.26963	1.190626	1.577035	1.190626	1.147449	1.536476	1.26963
12500	5	.822467	.1	1.525906	1.443551	1.11662	1.080625	1.519724	1.190626	1.147449	1.443551	1.11662
17500	7	.822467	.1	1.50831	1.330789	1.052953	1.020176	1.416743	1.243057	1.233009	1.330789	1.052953
25000	10	.822467	.1	1.424468	1.243057	1.042228	.981509	1.281214	1.412002	1.408797	1.243057	1.042228
50000	20	.822467	.1	1.206942	.7209369	1.077237	1.03861	.7960904	1.572565	1.572231	.7209369	1.077237
1500	3	3.289868	.5	1.328755	1.376532	1.283495	1.193382	1.352476	1.328755	1.292501	1.376532	1.283495
2500	5	3.289868	.5	1.182269	1.190626	1.156369	1.093979	1.212266	1.190626	1.144443	1.190626	1.156369
3500	7	3.289868	.5	1.107028	1.0494	1.144443	1.103793	1.119781	1.147449	1.110243	1.0494	1.144443
5000	10	3.289868	.5	.993373	.9315793	1.13225	1.097269	1.01264	1.07383	1.059995	.9315793	1.129159
10000	20	3.289868	.5	.5394442	.3843826	1.230463	1.204259	.5730969	1.212266	1.209612	.3843826	1.230463
3000	3	3.289868	.5	1.168037	1.187855	1.32671	1.230463	1.179453	1.344734	1.276617	1.187855	1.32671
5000	5	3.289868	.5	1.100541	1.027619	1.190626	1.100541	1.126051	1.162235	1.135324	1.027619	1.190626
7000	7	3.289868	.5	.957058	.9359006	1.080625	1.03861	.973468	1.052953	1.031305	.9359006	1.080625
10000	10	3.289868	.5	.7960904	.7270763	1.122925	1.09067	.8174105	1.087341	1.07383	.7270763	1.122925
20000	20	3.289868	.5	.5032296	.3443254	1.110243	1.087341	.5125524	1.156369	1.15931	.3443254	1.110243
7500	3	3.289868	.5	1.001154	1.052953	1.318423	1.209612	1.008836	1.330789	1.292501	1.052953	1.318423
12500	5	3.289868	.5	.8226239	.8819014	1.217534	1.147449	.8529889	1.196123	1.150439	.8819014	1.217534
17500	7	3.289868	.5	.7683163	.8121515	1.126051	1.066954	.7568289	1.056485	1.01642	.8121515	1.126051

Create PDF in your applications with the Pdfcrowd HTML to PDF API PDFCROWD

25000	10	3.289868	.5	.7084561	.5032296	1.042228	1.020176	.7147307	1.023909	.9972763	.5032296	1.042228
50000	20	3.289868	.5	.3582039	.2230471	1.023909	1.01264	.3715373	1.107028	1.110243	.2230471	1.023909
1500	3	3.289868	.1	1.571544	1.541684	1.179453	1.0494	1.580756	.9653186	.8819014	1.541684	1.179453
2500	5	3.289868	.1	1.499063	1.354385	1.334826	1.230463	1.514147	1.107028	1.087341	1.354385	1.334826
3500	7	3.289868	.1	1.390525	1.077237	1.360055	1.288022	1.398967	1.193382	1.176622	1.077237	1.360055
5000	10	3.289868	.1	1.204259	.8121515	1.39393	1.354385	1.150439	1.397297	1.400628	.8121515	1.39393
10000	20	3.289868	.1	.6485677	.2817091	1.557575	1.548544	.5648982	1.572892	1.571544	.2817091	1.557575
3000	3	3.289868	.1	1.442134	1.392232	1.361925	1.214907	1.537999	1.156369	1.107028	1.392232	1.361925
5000	5	3.289868	.1	1.37474	1.225333	1.250456	1.135324	1.440708	1.153412	1.129159	1.225333	1.250456
7000	7	3.289868	.1	1.2743	.9854897	1.245536	1.150439	1.281214	1.212266	1.198849	.9854897	1.245536
10000	10	3.289868	.1	1.100541	.7451107	1.283495	1.225333	1.063485	1.360055	1.356285	.7451107	1.283495
20000	20	3.289868	.1	.7209369	.1729451	1.463827	1.447753	.5394442	1.569328	1.567303	.1729451	1.463827
7500	3	3.289868	.1	1.296935	1.262533	1.285764	1.153412	1.431978	1.220148	1.165144	1.262533	1.285764
12500	5	3.289868	.1	1.097269	.9528851	1.248002	1.162235	1.176622	1.240564	1.204259	.9528851	1.248002
17500	7	3.289868	.1	1.008836	.7851369	1.230463	1.162235	1.027619	1.262533	1.252897	.7851369	1.230463
25000	10	3.289868	.1	.993373	.5216896	1.222747	1.179453	.8958571	1.427498	1.424468	.5216896	1.222747
50000	20	3.289868	.1	.5730969	.09995	1.206942	1.196123	.3443254	1.571191	1.570465	.09995	1.206942
1500	3	29.60881	.5	.8579044	1.110243	1.372938	1.240564	.8627804	1.434922	1.332813	1.110243	1.372938
2500	5	29.60881	.5	.7451107	.8529889	1.322588	1.212266	.7451107	1.305665	1.252897	.8529889	1.322588
3500	7	29.60881	.5	.5032296	.7270763	1.439274	1.336828	.5565609	1.383604	1.340802	.7270763	1.439274
5000	10	29.60881	.5	.3967871	.5394442	1.50117	1.463827	.4204284	1.475127	1.439274	.5394442	1.50117
10000	20	29.60881	.5	.09995	.09995	1.579997	1.577035	.09995	1.572231	1.570465	.09995	1.579997
3000	3	29.60881	.5	.7509993	.8958571	1.44496	1.285764	.7626008	1.483479	1.38185	.8958571	1.44496
5000	5	29.60881	.5	.6122009	.7568289	1.410404	1.252897	.6343185	1.380087	1.305665	.7568289	1.410404
7000	7	29.60881	.5	.4740359	.5648982	1.458599	1.356285	.5032296	1.403923	1.346685	.5648982	1.458599
10000	10	29.60881	.5	.2817091	.3146427	1.504274	1.451882	.2817091	1.459918	1.429	.3146427	1.504274
20000	20	29.60881	.5	.09995	.1412799	1.575551	1.568538	0	1.574722	1.569713	.1412799	1.575551
7500	3	29.60881	.5	.5969171	.7683163	1.422941	1.225333	.6122009	1.443551	1.360055	.7683163	1.422941
12500	5	29.60881	.5	.4534203	.5811626	1.427498	1.250456	.4534203	1.378314	1.316324	.5811626	1.427498
17500	7	29.60881	.5	.3715373	.4638534	1.422941	1.312094	.3146427	1.332813	1.296935	.4638534	1.422941
25000	10	29.60881	.5	.1995996	.3967871	1.478753	1.407182	.244213	1.403923	1.385348	.3967871	1.478753
50000	20	29.60881	.5	0	0	1.561278	1.550471	.09995	1.569328	1.562277	0	1.561278
1500	3	29.60881	.1	1.201561	1.352476	1.307819	1.135324	1.380087	1.193382	1.122925	1.352476	1.307819
2500	5	29.60881	.1	.993373	1.063485	1.433454	1.32671	1.077237	1.38185	1.37474	1.063485	1.433454
3500	7	29.60881	.1	.6485677	.8529889	1.535705	1.478753	.7209369	1.516967	1.511261	.8529889	1.535705
5000	10	29.60881	.1	.4427189	.5306505	1.580225	1.565567	.4839835	1.581136	1.581126	.5306505	1.580225
10000	20	29.60881	.1	0	0	1.416743	1.449138	0	1.165144	1.153412	0	1.416743
3000	3	29.60881	.1	.957058	1.122925	1.427498	1.264911	1.119781	1.388809	1.324655	1.122925	1.427498
5000	5	29.60881	.1	.8379976	.9444522	1.437832	1.296935	.9049862	1.429	1.408797	.9444522	1.437832
7000	7	29.60881	.1	.6759882	.7509993	1.525044	1.439274	.7021111	1.516967	1.510285	.7509993	1.525044
10000	10	29.60881	.1	.4204284	.5032296	1.570092	1.523299	.4534203	1.58111	1.58106	.5032296	1.570092
20000	20	29.60881	.1	.2230471	.09995	1.490339	1.521526	.1995996	1.15931	1.147449	.09995	1.490339
7500	3	29.60881	.1	.8068457	1.066954	1.421404	1.233009	.9612024	1.436381	1.367476	1.066954	1.421404
12500	5	29.60881	.1	.6414905	.8277922	1.458599	1.264911	.7331507	1.453241	1.434922	.8277922	1.458599
17500	7	29.60881	.1	.4317291	.5306505	1.494761	1.372938	.5216896	1.497999	1.490339	.5306505	1.494761
25000	10	29.60881	.1	.3298333	.3443254	1.567303	1.529284	.3146427	1.580984	1.580984	.3443254	1.567303
50000	20	29.60881	.1	.09995	0	1.555313	1.570092	0	1.087341	1.07383	0	1.555313

Mean error

Create PDF in your applications with the Pdfcrowd HTML to PDF API PDFCROWD

ssl	ssh	tsq1	cb	MH	MHfe	MHdl	MHbdl	P	Pdl	Pbdl	Pbdl	Pbdl
1500	3	0	.5	.1619386	.1604599	.1636815	.1660399	.1479947	.1513936	.1536268	.1604599	.1636815
2500	5	0	.5	.1286893	.1271877	.1291473	.130527	.1184974	.120621	.1215038	.1271713	.1291555
3500	7	0	.5	.1084212	.10689	.1073072	.1076195	.0997345	.100091	.1001821	.1068787	.107296
5000	10	0	.5	.0877606	.0878986	.088338	.0884889	.0833052	.0841916	.084379	.0878934	.0883368
10000	20	0	.5	.0646532	.0635756	.0635872	.0636191	.060349	.0605225	.0606323	.0635655	.0635689
3000	3	0	.5	.1168184	.1160158	.1174759	.1193754	.1070825	.1078939	.1090093	.1160158	.1174759
5000	5	0	.5	.0879064	.0870613	.0889014	.0896467	.0823174	.0838499	.0846939	.0870532	.0888886
7000	7	0	.5	.0757152	.0752079	.0757295	.0758806	.0710892	.0714282	.0716236	.07521	.0757316
10000	10	0	.5	.0641271	.0637606	.0641758	.0643002	.0605507	.0609912	.0610514	.0637573	.0641739
20000	20	0	.5	.0459366	.0456284	.0455033	.0455258	.0445404	.0446106	.0446939	.0456291	.0455027
7500	3	0	.5	.0716102	.0714023	.0725923	.0747247	.0669597	.0675757	.0691126	.0713966	.0725849
12500	5	0	.5	.055554	.0553313	.0565414	.0566529	.0534021	.0544183	.0545755	.0553313	.0565414
17500	7	0	.5	.0487988	.0485819	.0487084	.0489803	.0469091	.0473686	.047624	.0485793	.0487058
25000	10	0	.5	.0390424	.0388538	.0391061	.0391611	.0382403	.0384462	.0384722	.0388538	.0391061
50000	20	0	.5	.0285133	.0283402	.0285286	.0285719	.03046	.0304933	.0305457	.0283402	.0285286
1500	3	0	.1	.2909851	.2944086	.2825267	.2805237	.2097577	.2130564	.2152951	.2944086	.2825267
2500	5	0	.1	.2324897	.2436083	.2329646	.2303558	.181549	.1842895	.1852883	.2436083	.2329646
3500	7	0	.1	.186845	.2093818	.1979053	.193519	.1645683	.1656039	.16611	.2093818	.1979053
5000	10	0	.1	.1603329	.1976189	.1895539	.1857204	.160748	.1614674	.1621058	.1976612	.1895962
10000	20	0	.1	.1150252	.1682376	.1634534	.1590981	.1444544	.1448247	.1450641	.1682253	.1634411
3000	3	0	.1	.2123244	.2117674	.2109891	.2112417	.1682847	.1710905	.1741961	.2117674	.2109891
5000	5	0	.1	.1730547	.175908	.1722518	.1715382	.1605095	.1622506	.1631146	.175908	.1722518
7000	7	0	.1	.1395897	.1471869	.1411098	.1399064	.1527535	.1536654	.1541866	.1471869	.1411098
10000	10	0	.1	.1200305	.1307867	.1260759	.1243192	.1466802	.1471031	.1474368	.1307867	.1260759
20000	20	0	.1	.0778009	.1022703	.0966106	.0941645	.1452039	.1452917	.1453444	.1022703	.0966106
7500	3	0	.1	.1387689	.1379798	.1401806	.1430844	.1502328	.1515968	.1532074	.1379798	.1401806
12500	5	0	.1	.1041228	.1059381	.105635	.1057093	.146934	.1477446	.1480062	.1059381	.105635
17500	7	0	.1	.087604	.0907302	.0890099	.088878	.1458235	.1463921	.1467472	.0907302	.0890099
25000	10	0	.1	.071265	.0751729	.0737454	.0734458	.143666	.1438329	.144036	.0751729	.0737454
50000	20	0	.1	.0511129	.056715	.0547729	.0543116	.1444717	.1444925	.1445555	.0567124	.0547666
1500	3	.822467	.5	.5099574	.5200055	.4504611	.4530362	.4688732	.3933717	.3903199	.5200055	.4504611
2500	5	.822467	.5	.4308142	.452655	.3565669	.3583059	.4096745	.3205279	.3198183	.452636	.3566674
3500	7	.822467	.5	.4007087	.4371122	.2995615	.2988443	.3900529	.2760368	.2756354	.4371289	.2997212
5000	10	.822467	.5	.3701596	.4168507	.2512493	.2508172	.3634208	.2352744	.2351142	.4168498	.2512102
10000	20	.822467	.5	.3319253	.3961945	.1925462	.1922887	.3301409	.1899588	.1899735	.3961873	.1925873
3000	3	.822467	.5	.5174284	.526644	.4511178	.4529625	.4768231	.3916338	.3887158	.526644	.4511178
5000	5	.822467	.5	.4230132	.4423702	.3393435	.3411121	.4014127	.2979115	.2973119	.4424118	.339556
7000	7	.822467	.5	.3836109	.4111602	.279226	.2790032	.372439	.2551758	.2548749	.4111602	.279226
10000	10	.822467	.5	.3755814	.4210103	.2346146	.2342556	.3707095	.2261712	.226045	.421019	.23469
20000	20	.822467	.5	.3264777	.3802133	.183648	.1835833	.3241144	.1789467	.1789128	.3802115	.1836249
7500	3	.822467	.5	.5074258	.5137352	.4463927	.4460104	.4709568	.3878331	.3848404	.5137352	.4463927
12500	5	.822467	.5	.4321897	.4515641	.3292607	.3296749	.4131661	.2921642	.2914663	.4515641	.3292607
17500	7	.822467	.5	.3762186	.4061099	.2719433	.27229	.365593	.2429573	.2428136	.4061099	.2719433
25000	10	.822467	.5	.3623839	.4008911	.2341234	.2342832	.3563385	.2171259	.2170357	.400888	.2341345
50000	20	.822467	.5	.3354581	.3890156	.166685	.1666558	.3344557	.1699234	.1699265	.3890148	.1666883
1500	3	.822467	.1	.5401461	.6182455	.4798243	.4665256	.4326877	.4194081	.4194783	.6182455	.4798243
2500	5	.822467	.1	.4796112	.6265945	.4320543	.4182602	.422141	.4008868	.4019814	.6265945	.4320543
3500	7	.822467	.1	.4175225	.5822715	.3917631	.3809512	.3817574	.3669129	.3675198	.5822715	.3917631
5000	10	.822467	.1	.3887174	.611388	.3672434	.3597115	.3779495	.3582482	.3584964	.611388	.3672434

Create PDF in your applications with the Pdfcrowd HTML to PDF API PDFCROWD

10000	20	.822467	.1	.3670441	.6356828	.3555626	.3528972	.3790983	.3535334	.353641	.6356821	.3555417
3000	3	.822467	.1	.5316827	.6105778	.4572206	.4469741	.4354862	.4100239	.4107006	.6105778	.4572206
5000	5	.822467	.1	.4455353	.5631217	.3738305	.3684011	.3911719	.3662631	.367073	.5631217	.3738305
7000	7	.822467	.1	.3963221	.557888	.3255893	.321836	.3756732	.3502727	.350886	.557888	.3255893
10000	10	.822467	.1	.3756335	.5737325	.2989572	.2952112	.3802611	.3498294	.3501494	.5737345	.2989035
20000	20	.822467	.1	.3428547	.6019155	.248754	.2475629	.3762956	.3450464	.3452575	.6019115	.2487168
7500	3	.822467	.1	.537145	.5947767	.4558231	.45423	.4366472	.4000634	.3996649	.5947767	.4558231
12500	5	.822467	.1	.4346435	.5445686	.3446694	.342529	.3849506	.3602738	.36089	.5445686	.3446694
17500	7	.822467	.1	.4016525	.5566615	.2970532	.2956338	.3954064	.3587483	.359192	.5566615	.2970532
25000	10	.822467	.1	.3609193	.5377316	.2507191	.2501095	.367168	.334806	.3351105	.5377298	.2507042
50000	20	.822467	.1	.3414979	.5746428	.1932416	.1925487	.3757478	.3396179	.3397371	.5746413	.1932154
1500	3	3.289868	.5	1.004374	1.080789	.8009362	.7939041	.9317033	.6428534	.6344271	1.080802	.800949
2500	5	3.289868	.5	.935487	1.050367	.6439159	.6424375	.9247323	.5526606	.5503004	1.050367	.6439159
3500	7	3.289868	.5	.8922012	1.027664	.5563396	.5519713	.8975917	.4818641	.48042	1.027656	.5563483
5000	10	3.289868	.5	.8852785	1.03543	.4750801	.4733642	.9032217	.4275856	.4268798	1.035468	.4751521
10000	20	3.289868	.5	.9054122	1.072316	.3780708	.376627	.9262508	.3710392	.3708769	1.072341	.3779403
3000	3	3.289868	.5	1.039399	1.109353	.8425934	.8324238	.9676279	.6649571	.6577383	1.109353	.8425934
5000	5	3.289868	.5	.9103621	1.038651	.6115365	.61392	.9024415	.5196757	.5180791	1.038663	.6116827
7000	7	3.289868	.5	.8913819	1.029036	.5164808	.5180134	.8997857	.4507336	.4497545	1.02904	.5165023
10000	10	3.289868	.5	.9020573	1.042466	.4674495	.4677748	.9177653	.4235081	.4230378	1.042463	.4674292
20000	20	3.289868	.5	.8946862	1.057243	.3458287	.3454258	.9141279	.3480687	.3479798	1.057248	.3458585
7500	3	3.289868	.5	.9994068	1.073579	.8583696	.8703531	.9298762	.642216	.640027	1.073579	.8583696
12500	5	3.289868	.5	.9327251	1.037846	.6409505	.644971	.9158964	.5341048	.5330931	1.037867	.6410969
17500	7	3.289868	.5	.8921566	1.024032	.5291855	.5305288	.8953261	.4439122	.443303	1.024029	.529348
25000	10	3.289868	.5	.8830054	1.03499	.4367319	.4379425	.8996198	.3940057	.3937772	1.03499	.4367319
50000	20	3.289868	.5	.9208949	1.082315	.3273449	.3272069	.9434822	.3443214	.3442667	1.082315	.3273449
1500	3	3.289868	.1	1.034304	1.260553	.8203591	.7895385	1.035239	.9070872	.9060565	1.260553	.8203591
2500	5	3.289868	.1	.9126548	1.235555	.7008873	.6749344	.9518273	.8239066	.8248853	1.235555	.7008873
3500	7	3.289868	.1	.8875868	1.282631	.6487013	.6264268	.9455267	.7913756	.7920974	1.282631	.6487013
5000	10	3.289868	.1	.894749	1.329704	.6220815	.608448	.9525349	.8004983	.8010823	1.329735	.6221026
10000	20	3.289868	.1	.9044724	1.36821	.5796877	.5735726	.9449889	.7728815	.7731606	1.36821	.5796877
3000	3	3.289868	.1	1.061683	1.286947	.8395062	.8062928	1.0523	.9116914	.9123048	1.286947	.8395062
5000	5	3.289868	.1	.9169634	1.231493	.6688532	.6481062	.9533237	.8163021	.8178624	1.231493	.6688532
7000	7	3.289868	.1	.884431	1.292846	.5714942	.5562763	.9466831	.7838724	.7845244	1.292846	.5714942
10000	10	3.289868	.1	.8610173	1.306197	.5250932	.5142693	.9164225	.7627888	.7631279	1.306217	.5252138
20000	20	3.289868	.1	.9030784	1.399611	.4652459	.4592939	.9594595	.7718818	.7719928	1.399611	.4652459
7500	3	3.289868	.1	.99652	1.235645	.8030576	.7872031	.9441072	.8244818	.8242258	1.235645	.8030576
12500	5	3.289868	.1	.9202331	1.258545	.6353965	.6285297	.9530095	.8145027	.8149929	1.258554	.6354111
17500	7	3.289868	.1	.8899784	1.299137	.5416543	.5379447	.9536469	.7847766	.7852508	1.299137	.5416543
25000	10	3.289868	.1	.8643893	1.317251	.4814646	.4761159	.9350582	.7657473	.7660089	1.317251	.4814646
50000	20	3.289868	.1	.8795202	1.38385	.361347	.3585208	.9358942	.7482066	.7482907	1.38385	.361347
1500	3	29.60881	.5	2.123801	2.362307	1.838953	1.822922	2.040825	1.241615	1.227395	2.362445	1.840447
2500	5	29.60881	.5	1.926704	2.234466	1.492857	1.483693	2.040452	1.030795	1.02393	2.2348	1.494091
3500	7	29.60881	.5	1.952018	2.250165	1.360458	1.350205	2.115501	.9882384	.9836068	2.250237	1.360427
5000	10	29.60881	.5	2.005957	2.296443	1.325411	1.313021	2.214467	.974469	.9715092	2.296879	1.326142
10000	20	29.60881	.5	2.009538	2.296881	1.127789	1.119683	2.25486	.8887461	.8874382	2.296999	1.128102
3000	3	29.60881	.5	2.1964	2.448961	1.951138	1.929839	2.046329	1.266956	1.251935	2.449038	1.950871
5000	5	29.60881	.5	2.019204	2.337743	1.571525	1.570077	2.100963	1.067065	1.061303	2.337754	1.57174
7000	7	29.60881	.5	1.974409	2.324436	1.412139	1.407299	2.133271	1.006655	1.003809	2.324457	1.41228
10000	10	29.60881	.5	2.051803	2.387472	1.31226	1.296292	2.259807	.9804156	.9782578	2.387538	1.312475
20000	20	29.60881	.5	2.032907	2.352684	1.080881	1.072251	2.277222	.8829327	.8820632	2.352711	1.080966
7500	3	29.60881	.5	2.222521	2.525109	2.094857	2.08739	2.036303	1.251446	1.241959	2.525161	2.095111
12500	5	29.60881	.5	2.033844	2.410843	1.636647	1.647972	2.111671	1.072073	1.06891	2.410843	1.636647

Create PDF in your applications with the Pdfcrowd HTML to PDF API PDFCROWD

17500	7	29.60881	.5	1.935582	2.339123	1.382884	1.386582	2.093182	.9620957	.9606361	2.339143	1.383013
25000	10	29.60881	.5	1.986812	2.350405	1.21028	1.204224	2.187629	.9175821	.9166497	2.350413	1.210333
50000	20	29.60881	.5	2.033556	2.395979	1.002009	.994884	2.27812	.8564836	.8560649	2.395986	1.002151
1500	3	29.60881	.1	2.100147	2.318147	1.840742	1.823399	2.542892	2.115264	2.10485	2.318147	1.840742
2500	5	29.60881	.1	1.926041	2.159143	1.56841	1.547394	2.433135	1.944474	1.943812	2.159143	1.56841
3500	7	29.60881	.1	1.980605	2.250263	1.53348	1.512564	2.546476	1.960449	1.960181	2.250323	1.53362
5000	10	29.60881	.1	2.006435	2.315185	1.4933	1.473711	2.586363	1.941799	1.941645	2.315232	1.493465
10000	20	29.60881	.1	2.034232	2.359185	1.464166	1.453477	2.612909	1.935888	1.935893	2.359185	1.464166
3000	3	29.60881	.1	2.122921	2.387025	1.835793	1.810117	2.492351	2.104915	2.100411	2.387025	1.835793
5000	5	29.60881	.1	1.973381	2.302318	1.545573	1.515475	2.487768	1.94568	1.945652	2.302318	1.545573
7000	7	29.60881	.1	1.937043	2.297632	1.445925	1.421133	2.471729	1.923457	1.923364	2.297632	1.445925
10000	10	29.60881	.1	1.975715	2.382707	1.393143	1.369184	2.548441	1.930301	1.930263	2.382808	1.393353
20000	20	29.60881	.1	2.037185	2.452723	1.344303	1.328087	2.629179	1.935166	1.935168	2.452723	1.344303
7500	3	29.60881	.1	2.125485	2.423433	1.872327	1.849914	2.378658	1.981272	1.976209	2.423433	1.872327
12500	5	29.60881	.1	1.981919	2.37963	1.547925	1.52336	2.46608	1.943528	1.94362	2.37963	1.547925
17500	7	29.60881	.1	1.964993	2.404881	1.39555	1.370156	2.517795	1.929436	1.929291	2.404881	1.39555
25000	10	29.60881	.1	2.028235	2.494215	1.361307	1.33421	2.629011	1.969499	1.969423	2.494247	1.361369
50000	20	29.60881	.1	2.024239	2.508772	1.18819	1.168881	2.60577	1.925603	1.925601	2.508772	1.18819

SE of mean error

ssl	ssh	tsq1	cb	MH	MHfe	MHdl	MHbdl	P	Pdl	Pbdl	Pbdl	Pbdl
1500	3	0	.5	.000123	.000121	.0001239	.0001252	.000111	.0001131	.0001144	.000121	.0001239
2500	5	0	.5	.0000979	.0000962	.0000969	.0000975	.0000895	.0000904	.000091	.0000962	.0000969
3500	7	0	.5	.0000805	.0000784	.0000797	.0000805	.0000737	.0000749	.0000752	.0000784	.0000797
5000	10	0	.5	.0000673	.0000658	.0000658	.000066	.000062	.0000622	.0000623	.0000658	.0000659
10000	20	0	.5	.0000499	.0000487	.0000488	.0000489	.0000459	.0000462	.0000463	.0000487	.0000488
3000	3	0	.5	.0000887	.0000878	.0000894	.0000914	.0000815	.0000827	.0000843	.0000878	.0000894
5000	5	0	.5	.0000684	.0000674	.0000694	.0000697	.0000633	.0000644	.0000647	.0000674	.0000694
7000	7	0	.5	.0000564	.0000563	.0000565	.0000567	.0000546	.0000549	.0000551	.0000563	.0000565
10000	10	0	.5	.0000478	.0000475	.0000475	.0000476	.0000457	.0000456	.0000456	.0000475	.0000475
20000	20	0	.5	.0000348	.0000348	.0000349	.0000349	.0000342	.0000343	.0000343	.0000348	.0000349
7500	3	0	.5	.0000557	.0000556	.0000569	.0000587	.0000525	.0000536	.0000549	.0000556	.0000569
12500	5	0	.5	.0000394	.0000392	.0000406	.000041	.0000379	.0000387	.000039	.0000392	.0000406
17500	7	0	.5	.0000365	.0000362	.0000367	.0000369	.0000345	.0000347	.0000348	.0000362	.0000367
25000	10	0	.5	.0000291	.0000289	.0000289	.0000289	.000029	.000029	.000029	.0000289	.0000289
50000	20	0	.5	.0000204	.0000204	.0000205	.0000205	.0000224	.0000226	.0000226	.0000204	.0000205
1500	3	0	.1	.0002361	.0002242	.0002205	.0002199	.0001667	.0001712	.0001759	.0002242	.0002205
2500	5	0	.1	.0001804	.0001782	.0001733	.0001719	.0001387	.0001418	.0001428	.0001782	.0001733
3500	7	0	.1	.0001432	.0001531	.0001458	.0001431	.0001173	.0001189	.0001191	.0001531	.0001458
5000	10	0	.1	.0001292	.0001372	.0001322	.0001308	.0001087	.0001097	.0001099	.0001372	.0001322
10000	20	0	.1	.000086	.0001109	.0001071	.0001052	.0000847	.0000851	.0000853	.0001109	.0001071
3000	3	0	.1	.0001698	.0001603	.0001617	.0001646	.0001242	.0001288	.0001346	.0001603	.0001617
5000	5	0	.1	.0001318	.0001292	.0001284	.0001288	.0001131	.0001146	.0001148	.0001292	.0001284
7000	7	0	.1	.0001098	.0001087	.0001062	.0001057	.0000953	.0000958	.0000959	.0001087	.0001062
10000	10	0	.1	.0000936	.000095	.0000919	.0000912	.0000835	.0000838	.000084	.000095	.0000919
20000	20	0	.1	.0000596	.0000726	.0000695	.0000684	.0000596	.0000596	.0000596	.0000726	.0000695

Create PDF in your applications with the Pdfcrowd HTML to PDF API PDFCROWD

7500	3	0	.1	.0001062	.0001036	.0001087	.0001125	.0000927	.0000947	.0001018	.0001036	.0001087
12500	5	0	.1	.0000818	.0000806	.000081	.0000818	.000077	.0000777	.0000781	.0000806	.000081
17500	7	0	.1	.0000667	.0000663	.0000655	.0000657	.0000653	.0000656	.0000656	.0000663	.0000655
25000	10	0	.1	.0000552	.0000559	.000055	.0000549	.000054	.0000542	.0000543	.0000559	.000055
50000	20	0	.1	.0000385	.0000422	.0000416	.0000411	.0000388	.0000388	.0000388	.0000422	.0000416
1500	3	.822467	.5	.0003907	.0003902	.0003403	.0003389	.0003652	.0002981	.000294	.0003902	.0003403
2500	5	.822467	.5	.0003202	.0003321	.0002697	.0002707	.0003161	.0002454	.0002448	.0003321	.0002696
3500	7	.822467	.5	.0003002	.0003123	.0002262	.0002258	.0003017	.000213	.0002127	.0003122	.000226
5000	10	.822467	.5	.0002744	.0002864	.0001958	.0001952	.0002765	.0001876	.0001874	.0002864	.0001957
10000	20	.822467	.5	.0002158	.0002205	.0001451	.0001449	.0002161	.0001421	.0001421	.0002205	.0001452
3000	3	.822467	.5	.0003963	.0004018	.0003458	.0003457	.0003748	.0003027	.0002997	.0004018	.0003458
5000	5	.822467	.5	.0003149	.000322	.0002547	.0002564	.0003091	.000224	.0002236	.000322	.0002553
7000	7	.822467	.5	.0002828	.0002932	.0002082	.0002083	.0002836	.000193	.0001928	.0002932	.0002082
10000	10	.822467	.5	.0002722	.0002802	.0001824	.0001822	.0002745	.0001761	.000176	.0002802	.0001825
20000	20	.822467	.5	.0002181	.0002241	.0001383	.0001382	.0002187	.0001366	.0001366	.0002241	.0001383
7500	3	.822467	.5	.0003767	.0003762	.0003431	.0003381	.0003571	.000297	.0002902	.0003762	.0003431
12500	5	.822467	.5	.0003119	.0003236	.0002438	.0002439	.0003094	.0002191	.0002188	.0003236	.0002438
17500	7	.822467	.5	.0002895	.0002975	.000203	.0002033	.0002905	.0001884	.0001882	.0002975	.000203
25000	10	.822467	.5	.0002618	.0002699	.0001852	.0001852	.000263	.0001689	.0001688	.0002699	.0001852
50000	20	.822467	.5	.0002103	.0002115	.0001205	.0001205	.00021	.0001226	.0001225	.0002115	.0001205
1500	3	.822467	.1	.0004017	.0004574	.0003623	.0003539	.0004186	.0003841	.0003829	.0004574	.0003623
2500	5	.822467	.1	.0003383	.0004208	.0003021	.0002957	.0003575	.0003151	.0003151	.0004208	.0003021
3500	7	.822467	.1	.0002974	.0003869	.0002772	.0002725	.0003119	.0002847	.0002846	.0003869	.0002772
5000	10	.822467	.1	.0002664	.0003387	.0002384	.0002369	.0002653	.0002419	.000242	.0003387	.0002384
10000	20	.822467	.1	.0002234	.0002735	.0001967	.0001967	.0002097	.0001871	.0001872	.0002735	.0001967
3000	3	.822467	.1	.000397	.0004552	.0003409	.0003331	.0004155	.0003537	.0003522	.0004552	.0003409
5000	5	.822467	.1	.0003277	.0004055	.0002789	.0002729	.000349	.0003049	.0003056	.0004055	.0002789
7000	7	.822467	.1	.0002987	.0003762	.000245	.0002414	.0003054	.0002613	.0002616	.0003762	.000245
10000	10	.822467	.1	.0002606	.0003376	.0002049	.0002044	.0002599	.0002225	.0002226	.0003376	.000205
20000	20	.822467	.1	.0002257	.0002727	.0001703	.0001696	.0002052	.0001743	.0001742	.0002727	.0001704
7500	3	.822467	.1	.0004114	.0004628	.0003554	.0003537	.0004379	.0003729	.0003714	.0004628	.0003554
12500	5	.822467	.1	.0003199	.0003949	.0002615	.0002618	.0003329	.0002865	.0002866	.0003949	.0002615
17500	7	.822467	.1	.0003057	.0003867	.0002191	.0002181	.0003181	.0002603	.0002603	.0003867	.0002191
25000	10	.822467	.1	.0002555	.0003395	.0001852	.0001841	.0002561	.000218	.000218	.0003395	.0001852
50000	20	.822467	.1	.0002122	.0002625	.0001435	.0001433	.0001949	.000162	.000162	.0002625	.0001436
1500	3	3.289868	.5	.0006993	.0007727	.0006031	.0006012	.0006815	.0004933	.0004874	.0007727	.0006032
2500	5	3.289868	.5	.0006257	.0006852	.0004944	.0004922	.0006463	.0004307	.000429	.0006852	.0004944
3500	7	3.289868	.5	.0005522	.0006028	.0004212	.0004226	.000582	.0003715	.0003706	.0006028	.0004212
5000	10	3.289868	.5	.0005046	.0005342	.0003528	.0003524	.0005376	.0003188	.0003182	.0005342	.0003529
10000	20	3.289868	.5	.0003953	.0003911	.0002791	.0002785	.0004208	.0002574	.0002572	.0003912	.0002792
3000	3	3.289868	.5	.0007339	.0007883	.0006267	.0006322	.000709	.0005047	.0004998	.0007883	.0006267
5000	5	3.289868	.5	.0006355	.0007047	.000471	.0004727	.0006649	.0004112	.0004099	.0007047	.0004713
7000	7	3.289868	.5	.0005678	.000623	.0004057	.0004072	.0006012	.0003572	.0003565	.000623	.0004057
10000	10	3.289868	.5	.0005124	.0005387	.0003501	.0003502	.0005431	.000318	.0003178	.0005387	.0003501
20000	20	3.289868	.5	.0004016	.0004036	.0002574	.0002569	.0004261	.0002484	.0002483	.0004036	.0002575
7500	3	3.289868	.5	.0007217	.0008195	.000627	.0006323	.0007085	.0004915	.0004902	.0008195	.000627
12500	5	3.289868	.5	.0006191	.0006755	.0004919	.0004948	.000635	.0003985	.0003974	.0006755	.000492
17500	7	3.289868	.5	.0005611	.000612	.0003822	.0003829	.0005913	.0003387	.0003382	.000612	.0003823
25000	10	3.289868	.5	.0005164	.0005427	.000333	.0003336	.0005477	.0002992	.0002991	.0005427	.000333
50000	20	3.289868	.5	.0004097	.00041	.0002409	.0002407	.0004365	.0002435	.0002435	.00041	.0002409
1500	3	3.289868	.1	.0007248	.0008563	.0006197	.0005996	.0010317	.000805	.0008033	.0008563	.0006197
2500	5	3.289868	.1	.0006155	.0007594	.0005211	.0005122	.0008169	.0006559	.0006556	.0007594	.0005211
3500	7	3.289868	.1	.0005677	.0006624	.0004561	.00045	.0007265	.0005532	.0005531	.0006624	.0004561

Create PDF in your applications with the Pdfcrowd HTML to PDF API PDFCROWD

5000	10	3.289868	.1	.000502	.0005586	.0004072	.0004044	.0006169	.0004825	.0004823	.0005586	.0004072
10000	20	3.289868	.1	.0003831	.0003958	.0003163	.0003152	.0004541	.0003438	.0003437	.0003958	.0003163
3000	3	3.289868	.1	.0007416	.0008819	.0006211	.0006008	.0010314	.0008183	.0008179	.0008819	.0006211
5000	5	3.289868	.1	.0006444	.0007714	.0005215	.0005089	.0008692	.0006722	.0006713	.0007714	.0005215
7000	7	3.289868	.1	.0005745	.0006816	.0004314	.0004249	.0007415	.0005543	.000554	.0006816	.0004314
10000	10	3.289868	.1	.0004943	.0005708	.0003714	.0003675	.000605	.0004643	.0004643	.0005708	.0003714
20000	20	3.289868	.1	.0004028	.0004195	.0003108	.0003091	.0004681	.0003447	.0003446	.0004195	.0003108
7500	3	3.289868	.1	.0007257	.0008809	.0005879	.0005787	.0009901	.0007611	.0007604	.0008809	.0005879
12500	5	3.289868	.1	.0006163	.0007355	.0004857	.0004809	.0008239	.0006283	.0006279	.0007355	.0004857
17500	7	3.289868	.1	.000558	.00067	.0004178	.0004125	.0007121	.0005377	.0005376	.00067	.0004178
25000	10	3.289868	.1	.0005258	.0006012	.0003503	.000348	.0006379	.0004749	.0004748	.0006012	.0003503
50000	20	3.289868	.1	.0003913	.000403	.0002652	.0002644	.0004479	.0003293	.0003293	.000403	.0002652
1500	3	29.60881	.5	.0012097	.0014908	.0014243	.0014391	.001137	.0009444	.000932	.0014909	.0014273
2500	5	29.60881	.5	.0009609	.0012337	.0011391	.0011393	.0010209	.000778	.0007739	.0012334	.0011389
3500	7	29.60881	.5	.0008243	.0010599	.0009993	.0009977	.0008907	.0006891	.0006862	.0010599	.0009993
5000	10	29.60881	.5	.000739	.0009193	.0009481	.0009484	.0007982	.0006453	.0006436	.0009203	.0009491
10000	20	29.60881	.5	.0004986	.000622	.0007252	.0007248	.0005543	.0004858	.0004851	.000622	.0007252
3000	3	29.60881	.5	.0012505	.0015377	.0014213	.0014482	.0011428	.0009182	.0009041	.0015378	.0014215
5000	5	29.60881	.5	.0009971	.0013032	.0011989	.0011979	.0010287	.0007858	.0007815	.0013032	.001199
7000	7	29.60881	.5	.0008564	.0011002	.0010555	.0010526	.0009163	.0007019	.0007	.0011002	.0010553
10000	10	29.60881	.5	.0007341	.0009384	.0009505	.0009501	.0007867	.0006349	.0006338	.0009385	.0009504
20000	20	29.60881	.5	.0004916	.0006292	.0007158	.000715	.0005418	.0004805	.0004801	.0006293	.0007159
7500	3	29.60881	.5	.0013282	.0016714	.0015406	.0015608	.0011656	.0009245	.0009134	.0016715	.0015403
12500	5	29.60881	.5	.0010126	.001299	.0012274	.0012328	.0010302	.0007928	.0007901	.001299	.0012274
17500	7	29.60881	.5	.0008611	.001112	.0010506	.0010505	.0009232	.0007084	.0007075	.001112	.0010506
25000	10	29.60881	.5	.0007215	.0009562	.0009213	.0009211	.0007871	.0006033	.0006027	.0009562	.0009213
50000	20	29.60881	.5	.0005039	.0006454	.0007074	.0007056	.0005613	.0004814	.0004812	.0006454	.0007079
1500	3	29.60881	.1	.0012252	.0013274	.0013479	.0013519	.0018392	.0015237	.0015092	.0013274	.0013479
2500	5	29.60881	.1	.0009686	.0011122	.0011192	.0011291	.0015033	.0012311	.0012289	.0011122	.0011192
3500	7	29.60881	.1	.0008125	.0009465	.0009615	.0009648	.0012824	.00103	.0010286	.0009465	.0009615
5000	10	29.60881	.1	.0007068	.0007756	.000865	.0008679	.0011271	.0008893	.0008887	.0007756	.0008651
10000	20	29.60881	.1	.0004757	.0005077	.0006402	.0006415	.0007882	.0006291	.000629	.0005077	.0006402
3000	3	29.60881	.1	.0011908	.0013673	.0013483	.0013567	.0018037	.0014698	.0014604	.0013673	.0013483
5000	5	29.60881	.1	.0009572	.0011493	.0011238	.0011289	.0014992	.001211	.0012092	.0011493	.0011238
7000	7	29.60881	.1	.0008596	.0009823	.0010009	.0009995	.001339	.001032	.0010309	.0009823	.0010009
10000	10	29.60881	.1	.0007351	.0008365	.0009165	.0009171	.0011488	.0009163	.0009161	.0008365	.0009168
20000	20	29.60881	.1	.0005187	.000549	.0007228	.0007241	.000841	.0006615	.0006615	.000549	.0007228
7500	3	29.60881	.1	.0012659	.001558	.0014334	.0014581	.0018461	.0014935	.0014828	.001558	.0014334
12500	5	29.60881	.1	.0009686	.0012194	.0011718	.0011652	.0015166	.0012045	.0012032	.0012194	.0011718
17500	7	29.60881	.1	.0008374	.0010203	.0009927	.0009935	.001312	.001017	.0010167	.0010203	.0009927
25000	10	29.60881	.1	.0007087	.0008306	.000927	.0009243	.0011174	.0009034	.0009032	.0008306	.000927
50000	20	29.60881	.1	.0004868	.0005315	.0007119	.0007134	.000794	.0006165	.0006165	.0005315	.0007119

Mean bias

ssl	ssh	tsq1	cb	MH	MHfe	MHdl	MHbdl	P	Pdl	Pbdl	Pbdl	Pbdl
1500	3	0	.5	-.0007167	.0103576	.0058404	.0031432	.025972	.0272479	.0276783	.0103576	.0058404

Create PDF in your applications with the Pdfcrowd HTML to PDF API PDFCROWD

2500	5	0	.5	.0024192	.0160041	.0114628	.0098788	.0267302	.0273468	.0275559	.0159878	.011471
3500	7	0	.5	-.0034185	.0109902	.0064466	.005209	.0203812	.0197835	.0199177	.010979	.0064354
5000	10	0	.5	.0035645	.0181301	.0153718	.0144285	.0259014	.0266555	.0268608	.0181028	.0153334
10000	20	0	.5	-.0055692	.0100737	.0074485	.0065791	.0168275	.01691	.0170496	.0100452	.0074118
3000	3	0	.5	-.0034372	.0020499	.0000688	-.0009457	.0208708	.0223375	.0228134	.0020499	.0000688
5000	5	0	.5	.0011016	.0078562	.0047416	.0041355	.0237838	.023719	.023917	.0078481	.0047256
7000	7	0	.5	.0032062	.0100953	.007629	.007056	.0252305	.0251253	.0251596	.0100831	.0076168
10000	10	0	.5	-.0004924	.0066831	.0047365	.0041196	.0215487	.0214681	.0214231	.0066704	.0047205
20000	20	0	.5	-.0015355	.0059654	.0048176	.004458	.0202051	.0203009	.0204247	.0059521	.0048029
7500	3	0	.5	-.0014271	.0007601	-.001211	-.0025849	.0209671	.02059	.0205108	.0007543	-.0012184
12500	5	0	.5	-.000638	.0018939	.0003782	.0001059	.0212142	.0209842	.0210221	.0018939	.0003782
17500	7	0	.5	-.0034881	-.0008631	-.0013981	-.0015747	.0185008	.018891	.0189597	-.0008656	-.0014007
25000	10	0	.5	-.0030927	-.0003044	-.0009943	-.00118	.0186811	.0187861	.0188329	-.0003044	-.0009943
50000	20	0	.5	-.0010278	.0019665	.0013098	.001128	.0204022	.0203721	.020391	.0019665	.0013098
1500	3	0	.1	-.0266963	.0709466	.0462212	.035589	.1448766	.1486383	.151815	.0709466	.0462212
2500	5	0	.1	-.0145198	.1031369	.0821378	.0740156	.1393869	.142411	.1439477	.1031369	.0821378
3500	7	0	.1	.0000473	.1227568	.1074094	.0996572	.1405284	.1418512	.1424214	.1227568	.1074094
5000	10	0	.1	.0114307	.1373977	.1263116	.1194377	.1450502	.1457565	.1464184	.1373554	.1262693
10000	20	0	.1	.0132106	.1471451	.1416176	.135967	.140776	.1411464	.1414137	.1471327	.1416052
3000	3	0	.1	-.0339452	.0226493	.0019042	-.0064337	.1375624	.1406796	.1441115	.0226493	.0019042
5000	5	0	.1	-.0088088	.0588439	.0424614	.0362457	.1458768	.1476435	.1486059	.0588439	.0424614
7000	7	0	.1	-.00473	.0649523	.0520266	.0472999	.1447916	.1458746	.1463953	.0649523	.0520266
10000	10	0	.1	-.0059608	.0668254	.0562463	.0517307	.1419773	.1424694	.1428256	.0668254	.0562463
20000	20	0	.1	.005045	.0810999	.0742519	.0703417	.1450398	.1451276	.1451811	.0810999	.0742519
7500	3	0	.1	-.012254	.010761	-.0007919	-.0064848	.1430012	.1441499	.1457811	.010761	-.0007919
12500	5	0	.1	-.0031312	.0241479	.0163039	.0133614	.1453763	.1461912	.1464523	.0241479	.0163039
17500	7	0	.1	-.0010738	.0280317	.0212874	.0192454	.1453407	.1459093	.1462636	.0280317	.0212874
25000	10	0	.1	-.0023324	.028094	.0221244	.0201249	.1435753	.1437421	.1439453	.028094	.0221244
50000	20	0	.1	.0008254	.0324889	.0279689	.0262721	.1444717	.1444925	.1445555	.0324862	.0279625
1500	3	.822467	.5	.1673688	.2419803	.0500897	.0311464	.2121143	.1237285	.1199169	.2419803	.0500897
2500	5	.822467	.5	.2249021	.3082156	.0722295	.0611181	.2506437	.1258961	.1248791	.3081965	.0720448
3500	7	.822467	.5	.2798163	.3630179	.1019021	.0968512	.2958732	.1488473	.1484009	.3629986	.1017424
5000	10	.822467	.5	.2921649	.3734264	.1028211	.0999945	.3034704	.1430711	.1430551	.3734255	.102782
10000	20	.822467	.5	.3061242	.3877855	.1032093	.1023123	.3109613	.1419289	.1420155	.3877603	.1030172
3000	3	.822467	.5	.1783779	.2423896	.0406004	.0273134	.2226513	.1274866	.1246637	.2423896	.0406004
5000	5	.822467	.5	.2110598	.2837238	.0240382	.0156575	.2381038	.0976633	.0967295	.2836821	.0238257
7000	7	.822467	.5	.2595824	.3301574	.0608409	.0574887	.2760692	.1295916	.1293856	.3301574	.0608409
10000	10	.822467	.5	.3133782	.383207	.0823751	.0804166	.3229303	.1467275	.1466446	.3831932	.0822562
20000	20	.822467	.5	.2944379	.3671115	.0528754	.0523055	.3003333	.1191615	.1192284	.3671097	.0528523
7500	3	.822467	.5	.1985217	.2522489	.0594647	.0474045	.2382982	.147645	.1442161	.2522489	.0594647
12500	5	.822467	.5	.2457105	.3082316	.0407514	.0361647	.2693398	.1270301	.1262937	.3082316	.0407514
17500	7	.822467	.5	.2595697	.3262319	.027789	.0255316	.2754772	.1133263	.113052	.3262319	.027789
25000	10	.822467	.5	.2892415	.3563998	.0346436	.0334314	.2999813	.121124	.1210268	.3563967	.0345596
50000	20	.822467	.5	.3154123	.3809243	.0389402	.0385686	.3197725	.1237801	.1237944	.3809235	.0389171
1500	3	.822467	.1	.154949	.3548606	.1793013	.1538649	.3319557	.3219918	.324122	.3548606	.1793013
2500	5	.822467	.1	.2540196	.507827	.2713715	.2504972	.3680363	.3484163	.3503894	.507827	.2713715
3500	7	.822467	.1	.2521183	.5204177	.2908372	.2751505	.3468959	.3329518	.334231	.5204177	.2908372
5000	10	.822467	.1	.2954351	.5810973	.3120121	.3027008	.3614487	.3422887	.3427536	.5810973	.3120121
10000	20	.822467	.1	.3360247	.6321945	.3443153	.3414129	.3767055	.3513935	.3515601	.6321938	.3442944
3000	3	.822467	.1	.1811018	.384012	.1231028	.089608	.3602553	.3405634	.342917	.384012	.1231028
5000	5	.822467	.1	.2152729	.4511835	.1576301	.1342837	.3491075	.3274463	.3288961	.4511835	.1576301
7000	7	.822467	.1	.249277	.5042481	.177982	.1647256	.3542169	.330094	.3311369	.5042481	.177982
10000	10	.822467	.1	.2906186	.5517333	.2094205	.2020731	.3695424	.3410623	.3415962	.5517352	.2093668

Create PDF in your applications with the Pdfcrowd HTML to PDF API PDFCROWD

20000	20	.822467	.1	.3177546	.5996042	.2111737	.2098486	.3751806	.344311	.3445391	.5996002	.2111365
7500	3	.822467	.1	.1709337	.3356759	.0524057	.022405	.3629606	.3335519	.334228	.3356759	.0524057
12500	5	.822467	.1	.2137488	.4208681	.0817661	.0660387	.3482541	.3274695	.3286109	.4208681	.0817661
17500	7	.822467	.1	.2795771	.5071574	.1140781	.106082	.3805871	.3460553	.3466493	.5071574	.1140781
25000	10	.822467	.1	.270992	.5108351	.0972961	.0922042	.3586574	.328031	.3284488	.5108333	.0972812
50000	20	.822467	.1	.3129891	.5702373	.1097468	.1086108	.3747728	.3388366	.3389607	.5702358	.1097206
1500	3	3.289868	.5	.4914121	.6987117	.1215914	.0704723	.5793705	.2641516	.2565667	.6987245	.1216042
2500	5	3.289868	.5	.7251058	.9164017	.2665134	.2391483	.7700849	.3472958	.3449709	.9164017	.2665134
3500	7	3.289868	.5	.7728226	.9696417	.252328	.2330912	.8058294	.3292136	.3279082	.9696338	.2521745
5000	10	3.289868	.5	.8332792	1.016429	.2454118	.2360053	.8615943	.319635	.3190325	1.016467	.2453386
10000	20	3.289868	.5	.9000745	1.071362	.2608732	.2576837	.9218129	.3282972	.3283098	1.071387	.260702
3000	3	3.289868	.5	.5653823	.7525058	.1498274	.098894	.6495982	.3191794	.3118268	.7525058	.1498274
5000	5	3.289868	.5	.6985547	.9060705	.1693606	.1423985	.7436544	.3100468	.3083836	.9060824	.1695069
7000	7	3.289868	.5	.7758226	.976197	.1620323	.1455099	.80975	.3000724	.2990994	.9762004	.1620538
10000	10	3.289868	.5	.8410876	1.017182	.1836108	.1756665	.8697265	.3121492	.3118189	1.017179	.1835905
20000	20	3.289868	.5	.8849206	1.055882	.1716878	.1690845	.9061455	.2999326	.2998962	1.055887	.1716482
7500	3	3.289868	.5	.5015411	.7067136	.0137698	-.034902	.5897711	.2518641	.2482504	.7067136	.0137698
12500	5	3.289868	.5	.6944297	.8839831	.1113513	.0915129	.7452423	.3034195	.3023251	.8839618	.1112049
17500	7	3.289868	.5	.754835	.9501977	.07997	.0693587	.7914862	.2763155	.275894	.9501944	.0798075
25000	10	3.289868	.5	.8298159	1.01036	.0943926	.0883793	.8576701	.2872371	.287038	1.01036	.0943926
50000	20	3.289868	.5	.9164269	1.081962	.1159507	.1141534	.9399865	.3032492	.3032211	1.081962	.1159507
1500	3	3.289868	.1	.6050068	.95772	.459714	.4160495	.9112215	.7875873	.7896075	.95772	.459714
2500	5	3.289868	.1	.7153143	1.133803	.5241334	.4907219	.8964548	.7710196	.7728725	1.133803	.5241334
3500	7	3.289868	.1	.7958258	1.24733	.5526004	.5260944	.9217731	.7692562	.7704854	1.24733	.5526004
5000	10	3.289868	.1	.8555573	1.319271	.574387	.5583289	.9436843	.791778	.792378	1.319302	.5744082
10000	20	3.289868	.1	.8996695	1.367913	.5664958	.5604233	.9441361	.7719988	.7723051	1.367913	.5664958
3000	3	3.289868	.1	.603549	.9685405	.3537499	.2945779	.9411986	.8065493	.8086402	.9685405	.3537499
5000	5	3.289868	.1	.6876105	1.120868	.3931096	.3491425	.9001201	.7682923	.770428	1.120868	.3931096
7000	7	3.289868	.1	.7777869	1.260161	.3990692	.3649861	.924516	.7640706	.7651139	1.260161	.3990692
10000	10	3.289868	.1	.8055819	1.294564	.4043761	.3850925	.9062865	.7539429	.7543381	1.294584	.4042556
20000	20	3.289868	.1	.8965068	1.399608	.4230771	.4155383	.9591433	.7716844	.7717955	1.399608	.4230771
7500	3	3.289868	.1	.4821465	.8935793	.1164913	.0496903	.8289553	.7187672	.7208152	.8935793	.1164913
12500	5	3.289868	.1	.6964431	1.148047	.2683096	.2235001	.9039242	.7717573	.7728807	1.148056	.2683243
17500	7	3.289868	.1	.7881999	1.266908	.2840281	.2536593	.9342446	.7681183	.7688081	1.266908	.2840281
25000	10	3.289868	.1	.8103564	1.305764	.2719977	.2519914	.926509	.7592169	.7594914	1.305764	.2719977
50000	20	3.289868	.1	.8736755	1.383458	.2554471	.2491865	.9354556	.7479464	.7480292	1.383458	.2554471
1500	3	29.60881	.5	1.530915	1.887795	.9874011	.9405807	1.710238	.8700172	.8554845	1.887933	.9888943
2500	5	29.60881	.5	1.73295	2.066838	.9570304	.9135946	1.91899	.8271262	.8201492	2.066733	.9557958
3500	7	29.60881	.5	1.863266	2.192296	1.011987	.9740833	2.054098	.8636417	.8589448	2.192367	1.011956
5000	10	29.60881	.5	1.993673	2.276494	1.098058	1.073293	2.205778	.9033837	.9004849	2.27693	1.09879
10000	20	29.60881	.5	2.009161	2.296228	1.055987	1.043806	2.254452	.873461	.8721743	2.296346	1.056028
3000	3	29.60881	.5	1.463395	1.860116	.8586849	.7908132	1.671109	.8645123	.8508632	1.860193	.858418
5000	5	29.60881	.5	1.760825	2.121016	.8912813	.8212875	1.949583	.8439285	.8383322	2.121026	.8914964
7000	7	29.60881	.5	1.873757	2.24987	.9252695	.8788138	2.065891	.8669997	.8642558	2.249891	.9251284
10000	10	29.60881	.5	2.037934	2.378509	1.054528	1.019861	2.24852	.9241174	.921974	2.378576	1.054536
20000	20	29.60881	.5	2.032753	2.352684	.9636324	.9495972	2.276984	.8695337	.8686694	2.352711	.9637179
7500	3	29.60881	.5	1.382246	1.866488	.6740916	.5795505	1.637163	.8243895	.8164855	1.86654	.6736135
12500	5	29.60881	.5	1.782087	2.226519	.7807493	.6997668	1.963748	.8634172	.8606635	2.226519	.7807493
17500	7	29.60881	.5	1.848648	2.277052	.7408349	.6746241	2.034462	.8299245	.8284797	2.277071	.7409631
25000	10	29.60881	.5	1.962822	2.334789	.7686782	.7273537	2.169032	.8410489	.8401197	2.334797	.7687312
50000	20	29.60881	.5	2.031468	2.395471	.8019842	.7854941	2.276603	.8429227	.8425001	2.395478	.8021256
1500	3	29.60881	.1	1.681761	1.978975	1.452317	1.425521	2.414788	1.990145	1.981206	1.978975	1.452317
2500	5	29.60881	.1	1.768085	2.050015	1.390412	1.365425	2.383659	1.896977	1.896772	2.050015	1.390412

Create PDF in your applications with the Pdfcrowd HTML to PDF API PDFCROWD

3500	7	29.60881	.1	1.921656	2.214219	1.446433	1.422492	2.527241	1.943563	1.943493	2.214279	1.446573
5000	10	29.60881	.1	1.988796	2.307447	1.454147	1.433015	2.580684	1.937719	1.937582	2.307495	1.454312
10000	20	29.60881	.1	2.034232	2.359185	1.455674	1.444708	2.612909	1.935888	1.935893	2.359185	1.455674
3000	3	29.60881	.1	1.584787	1.964026	1.264758	1.222209	2.365799	1.985202	1.981758	1.964026	1.264758
5000	5	29.60881	.1	1.789645	2.18132	1.22551	1.181014	2.44265	1.906624	1.907189	2.18132	1.22551
7000	7	29.60881	.1	1.8462	2.232912	1.254754	1.218103	2.449319	1.902209	1.902219	2.232912	1.254754
10000	10	29.60881	.1	1.960106	2.372728	1.294105	1.261934	2.54461	1.926923	1.926934	2.372829	1.294315
20000	20	29.60881	.1	2.036606	2.452723	1.324764	1.306959	2.629179	1.9351	1.935105	2.452723	1.324764
7500	3	29.60881	.1	1.428322	1.887873	.9411622	.8807883	2.239725	1.850536	1.846969	1.887873	.9411622
12500	5	29.60881	.1	1.737448	2.210716	1.05461	.9830193	2.413466	1.898179	1.898566	2.210716	1.05461
17500	7	29.60881	.1	1.888102	2.35004	1.083279	1.019113	2.499929	1.913765	1.913754	2.35004	1.083279
25000	10	29.60881	.1	2.007221	2.479895	1.174633	1.123172	2.624395	1.965232	1.965157	2.479927	1.174696
50000	20	29.60881	.1	2.02412	2.508772	1.143696	1.118551	2.60577	1.925603	1.925601	2.508772	1.143696

SE of mean bias

ssl	ssh	tsq1	cb	MH	MHfe	MHdl	MHbdl	P	Pdl	Pbdl	Pbdl	Pbdl
1500	3	0	.5	.0002034	.0002008	.0002053	.000208	.0001832	.0001871	.0001896	.0002008	.0002053
2500	5	0	.5	.0001617	.0001587	.0001611	.0001627	.0001461	.0001483	.0001493	.0001587	.0001611
3500	7	0	.5	.000135	.0001321	.0001336	.0001343	.0001224	.0001235	.0001237	.0001321	.0001336
5000	10	0	.5	.0001106	.0001083	.0001091	.0001095	.0001006	.0001013	.0001014	.0001083	.0001092
10000	20	0	.5	.0000815	.0000795	.0000799	.00008	.000074	.0000742	.0000744	.0000794	.0000798
3000	3	0	.5	.0001467	.0001455	.0001477	.0001504	.000133	.0001341	.0001359	.0001455	.0001477
5000	5	0	.5	.0001114	.0001098	.0001127	.0001135	.0001011	.000103	.0001039	.0001098	.0001127
7000	7	0	.5	.0000944	.0000934	.0000942	.0000945	.000086	.0000865	.0000868	.0000934	.0000942
10000	10	0	.5	.00008	.0000792	.0000798	.0000799	.0000728	.0000731	.0000731	.0000792	.0000798
20000	20	0	.5	.0000576	.0000571	.0000571	.0000572	.0000524	.0000525	.0000525	.0000571	.0000571
7500	3	0	.5	.0000908	.0000905	.0000922	.000095	.0000825	.0000838	.0000859	.0000905	.0000922
12500	5	0	.5	.0000681	.0000678	.0000697	.00007	.000062	.0000634	.0000637	.0000678	.0000697
17500	7	0	.5	.0000609	.0000606	.000061	.0000613	.0000552	.0000556	.0000559	.0000606	.000061
25000	10	0	.5	.0000486	.0000485	.0000486	.0000487	.0000442	.0000443	.0000444	.0000485	.0000486
50000	20	0	.5	.000035	.0000349	.0000351	.0000352	.0000319	.000032	.0000321	.0000349	.0000351
1500	3	0	.1	.0003739	.0003633	.0003555	.0003548	.0002255	.0002294	.000233	.0003633	.0003555
2500	5	0	.1	.000294	.0002837	.0002786	.0002778	.0001811	.0001839	.0001844	.0002837	.0002786
3500	7	0	.1	.0002355	.0002285	.0002211	.0002192	.0001453	.0001465	.0001466	.0002285	.0002211
5000	10	0	.1	.0002056	.0001976	.0001936	.0001932	.0001289	.0001299	.0001301	.0001976	.0001936
10000	20	0	.1	.000143	.0001377	.0001347	.0001338	.0000907	.0000911	.0000912	.0001377	.0001347
3000	3	0	.1	.0002698	.0002647	.0002659	.0002678	.0001576	.0001615	.0001664	.0002647	.0002659
5000	5	0	.1	.0002174	.0002103	.0002107	.0002115	.0001314	.0001329	.0001331	.0002103	.0002107
7000	7	0	.1	.0001776	.0001711	.0001688	.0001689	.000107	.0001073	.0001074	.0001711	.0001688
10000	10	0	.1	.0001522	.0001472	.0001456	.0001453	.0000912	.0000915	.0000916	.0001472	.0001456
20000	20	0	.1	.0000979	.0000957	.000093	.0000928	.00006	.00006	.00006	.0000957	.000093
7500	3	0	.1	.0001744	.0001723	.0001774	.0001819	.0001035	.0001057	.0001122	.0001723	.0001774
12500	5	0	.1	.0001324	.000131	.0001322	.000133	.0000799	.0000806	.0000809	.000131	.0001322
17500	7	0	.1	.0001102	.0001088	.0001085	.0001089	.0000664	.0000667	.0000667	.0001088	.0001085
25000	10	0	.1	.0000901	.0000894	.0000893	.0000895	.0000543	.0000545	.0000546	.0000894	.0000893
50000	20	0	.1	.000064	.0000628	.0000628	.0000628	.0000388	.0000388	.0000388	.0000628	.0000628

Create PDF in your applications with the Pdfcrowd HTML to PDF API PDFCROWD

1500	3	.822467	.5	.0006204	.0006036	.0005625	.0005651	.0005554	.000478	.0004738	.0006036	.0005625
2500	5	.822467	.5	.0004875	.0004694	.0004414	.000445	.0004528	.0003837	.000383	.0004694	.0004414
3500	7	.822467	.5	.0004153	.000396	.0003614	.000362	.0003946	.0003153	.000315	.0003961	.0003615
5000	10	.822467	.5	.0003564	.0003411	.0003016	.0003018	.0003413	.0002648	.0002645	.0003411	.0003015
10000	20	.822467	.5	.0002511	.000235	.000218	.000218	.0002429	.0001902	.0001901	.000235	.0002181
3000	3	.822467	.5	.0006271	.0006166	.0005671	.0005694	.0005643	.0004785	.0004749	.0006166	.0005671
5000	5	.822467	.5	.0004834	.000468	.0004238	.0004266	.0004473	.0003598	.0003593	.000468	.0004243
7000	7	.822467	.5	.0003998	.0003822	.000343	.0003435	.0003781	.0002926	.0002923	.0003822	.000343
10000	10	.822467	.5	.000342	.00033	.0002856	.0002858	.0003294	.0002463	.0002462	.0003301	.0002858
20000	20	.822467	.5	.0002598	.000245	.0002238	.0002238	.0002504	.000191	.0001909	.000245	.0002238
7500	3	.822467	.5	.0006001	.0005848	.00056	.0005578	.000541	.0004658	.0004601	.0005848	.00056
12500	5	.822467	.5	.0004731	.0004623	.0004078	.0004086	.0004404	.0003425	.000342	.0004623	.0004078
17500	7	.822467	.5	.0003975	.0003835	.0003383	.000339	.0003771	.0002859	.0002857	.0003835	.0003383
25000	10	.822467	.5	.000341	.0003264	.0002966	.0002968	.0003259	.000247	.000247	.0003264	.0002966
50000	20	.822467	.5	.0002394	.0002258	.000202	.0002021	.0002318	.0001691	.0001691	.0002258	.000202
1500	3	.822467	.1	.0006553	.0006825	.0005741	.0005652	.0005023	.0004688	.0004664	.0006825	.0005741
2500	5	.822467	.1	.0005293	.0005585	.0004521	.0004469	.000413	.0003724	.0003717	.0005585	.0004521
3500	7	.822467	.1	.0004464	.0004668	.0003818	.0003791	.0003503	.0003238	.0003231	.0004668	.0003818
5000	10	.822467	.1	.0003672	.0003884	.0003072	.0003065	.0002874	.0002641	.0002638	.0003884	.0003072
10000	20	.822467	.1	.0002678	.0002814	.0002158	.0002161	.0002139	.0001911	.000191	.0002814	.0002158
3000	3	.822467	.1	.0006385	.0006579	.0005571	.0005504	.0004822	.0004211	.0004186	.0006579	.0005571
5000	5	.822467	.1	.0005096	.0005273	.0004391	.0004385	.0003912	.0003463	.0003464	.0005273	.0004391
7000	7	.822467	.1	.0004293	.0004456	.0003667	.0003671	.0003301	.0002864	.0002862	.0004456	.0003667
10000	10	.822467	.1	.000353	.0003725	.0002959	.0002969	.000275	.0002357	.0002356	.0003725	.0002959
20000	20	.822467	.1	.0002599	.0002777	.0002152	.0002146	.0002072	.0001757	.0001757	.0002777	.0002152
7500	3	.822467	.1	.0006549	.0006749	.0005758	.0005754	.0005008	.0004335	.0004313	.0006749	.0005758
12500	5	.822467	.1	.0004957	.0005248	.000425	.0004262	.0003712	.0003235	.0003231	.0005248	.000425
17500	7	.822467	.1	.0004204	.0004497	.0003511	.0003518	.0003357	.000277	.0002768	.0004497	.0003511
25000	10	.822467	.1	.0003495	.0003788	.0002962	.0002967	.0002679	.000228	.0002279	.0003788	.0002962
50000	20	.822467	.1	.0002524	.0002719	.0002143	.0002141	.0001968	.0001637	.0001636	.0002719	.0002143
1500	3	3.289868	.5	.0011212	.0011304	.0009956	.0009937	.0009987	.0007663	.000758	.0011304	.0009956
2500	5	3.289868	.5	.000861	.0008563	.0007671	.0007734	.0008247	.0006087	.0006067	.0008563	.0007671
3500	7	3.289868	.5	.0007099	.0006924	.0006508	.0006551	.0007037	.0005118	.0005106	.0006924	.0006509
5000	10	3.289868	.5	.0005866	.0005696	.0005386	.000541	.0006021	.000427	.0004264	.0005696	.0005387
10000	20	3.289868	.5	.0004073	.0003937	.000391	.0003912	.0004305	.0003101	.0003098	.0003938	.0003911
3000	3	3.289868	.5	.0011402	.0011342	.0010397	.0010409	.0010087	.0007716	.0007652	.0011342	.0010397
5000	5	3.289868	.5	.0008631	.0008687	.0007533	.0007618	.0008389	.0005858	.0005844	.0008687	.0007536
7000	7	3.289868	.5	.0007178	.000703	.0006366	.0006428	.000718	.0004907	.00049	.000703	.0006367
10000	10	3.289868	.5	.0006074	.0005851	.0005546	.0005575	.0006172	.000428	.0004275	.0005851	.0005546
20000	20	3.289868	.5	.0004227	.0004072	.0003956	.000396	.0004428	.0003048	.0003047	.0004072	.0003957
7500	3	3.289868	.5	.0011265	.0011512	.0010633	.0010756	.0010096	.0007687	.0007672	.0011512	.0010633
12500	5	3.289868	.5	.0008783	.0008674	.0008005	.000808	.0008289	.0005935	.0005924	.0008674	.0008007
17500	7	3.289868	.5	.0007357	.0007214	.0006481	.0006508	.0007245	.0004853	.0004847	.0007214	.0006483
25000	10	3.289868	.5	.0005982	.0005874	.0005412	.0005436	.0006114	.0004029	.0004027	.0005874	.0005412
50000	20	3.289868	.5	.0004196	.000411	.0003896	.00039	.000444	.0002931	.0002931	.000411	.0003896
1500	3	3.289868	.1	.0011089	.0011856	.0009199	.0009001	.0011428	.0009224	.0009181	.0011856	.0009199
2500	5	3.289868	.1	.0008369	.0009044	.0006988	.0006909	.0008773	.0007174	.0007162	.0009044	.0006988
3500	7	3.289868	.1	.0006906	.0007267	.0005688	.0005641	.0007564	.0005836	.0005828	.0007267	.0005688
5000	10	3.289868	.1	.0005663	.0005828	.0004722	.0004712	.0006304	.0004967	.0004965	.0005828	.0004722
10000	20	3.289868	.1	.0003942	.0003968	.0003394	.0003381	.0004559	.0003458	.0003457	.0003968	.0003394
3000	3	3.289868	.1	.0011461	.0012233	.0009828	.0009617	.0011338	.0009222	.0009206	.0012233	.0009828
5000	5	3.289868	.1	.0008853	.000925	.0007517	.0007466	.0009242	.0007266	.0007253	.000925	.0007517
7000	7	3.289868	.1	.0007124	.0007404	.0005946	.0005975	.0007689	.0005813	.0005806	.0007404	.0005946

Create PDF in your applications with the Pdfcrowd HTML to PDF API PDFCROWD

10000	10	3.289868	.1	.0005804	.0005968	.0005003	.0005013	.0006201	.0004785	.0004785	.0005968	.0005005
20000	20	3.289868	.1	.0004172	.0004196	.0003662	.0003659	.0004687	.0003452	.0003451	.0004196	.0003662
7500	3	3.289868	.1	.0011349	.0012268	.0009888	.0009761	.0010884	.0008618	.0008592	.0012268	.0009888
12500	5	3.289868	.1	.0008614	.0008984	.0007536	.0007594	.0008775	.0006801	.0006791	.0008984	.0007536
17500	7	3.289868	.1	.0006945	.0007292	.0006225	.0006288	.0007374	.0005613	.0005609	.0007292	.0006225
25000	10	3.289868	.1	.0006059	.0006258	.0005298	.0005334	.0006502	.0004853	.0004852	.0006258	.0005298
50000	20	3.289868	.1	.0004042	.0004043	.0003684	.0003693	.0004488	.0003299	.0003299	.0004043	.0003684
1500	3	29.60881	.5	.0019059	.0020594	.0021066	.0021241	.0015919	.0012952	.0012822	.0020596	.0021092
2500	5	29.60881	.5	.001278	.0014979	.001616	.0016328	.0012343	.000992	.0009875	.0014984	.0016178
3500	7	29.60881	.5	.0010092	.001175	.0013513	.0013677	.0010245	.0008401	.0008372	.0011751	.0013513
5000	10	29.60881	.5	.0007716	.0009677	.0012043	.0012133	.0008219	.0007416	.0007398	.0009687	.0012053
10000	20	29.60881	.5	.0005001	.0006244	.0008264	.0008304	.000556	.0005128	.0005121	.0006244	.0008267
3000	3	29.60881	.5	.0020614	.0022146	.0022567	.0022802	.0016438	.0013045	.001289	.0022147	.0022567
5000	5	29.60881	.5	.0014042	.0016327	.0017647	.0017965	.0012931	.0010219	.0010172	.0016327	.0017649
7000	7	29.60881	.5	.0010589	.0012457	.001501	.0015223	.0010596	.0008687	.0008666	.0012457	.0015011
10000	10	29.60881	.5	.0007718	.000961	.0012305	.0012424	.0008184	.0007145	.0007133	.000961	.0012306
20000	20	29.60881	.5	.0004923	.0006292	.0008674	.0008715	.0005428	.0005044	.000504	.0006293	.0008675
7500	3	29.60881	.5	.00219	.0023852	.0025123	.0025419	.0016812	.0013199	.0013081	.0023852	.0025124
12500	5	29.60881	.5	.0014096	.0015947	.0018915	.001936	.0012903	.0010162	.0010132	.0015947	.0018915
17500	7	29.60881	.5	.0010348	.0012342	.0015712	.0016039	.0010464	.0008596	.0008586	.0012343	.0015713
25000	10	29.60881	.5	.0007844	.0009937	.0013129	.0013306	.000837	.0007062	.0007056	.0009937	.0013129
50000	20	29.60881	.5	.0005122	.0006473	.0009282	.0009333	.0005674	.0005048	.0005046	.0006473	.0009286
1500	3	29.60881	.1	.0017564	.0017947	.0017599	.0017668	.0020046	.001684	.0016684	.0017947	.0017599
2500	5	29.60881	.1	.0012338	.0013026	.0013341	.0013437	.0015806	.0013032	.0013004	.0013026	.0013341
3500	7	29.60881	.1	.0009437	.0010281	.0010881	.0010934	.0013199	.0010615	.0010598	.0010281	.0010882
5000	10	29.60881	.1	.000755	.0007983	.0009294	.0009336	.0011401	.0008982	.0008975	.0007984	.0009295
10000	20	29.60881	.1	.0004757	.0005077	.0006593	.000661	.0007882	.0006291	.000629	.0005077	.0006593
3000	3	29.60881	.1	.001848	.0019266	.0018948	.001904	.0019669	.001628	.0016179	.0019266	.0018948
5000	5	29.60881	.1	.0012682	.0013652	.0014665	.0014756	.0015717	.0012716	.0012691	.0013652	.0014665
7000	7	29.60881	.1	.0010407	.0011218	.0012324	.0012391	.0013796	.0010707	.0010695	.0011218	.0012324
10000	10	29.60881	.1	.0007758	.0008644	.0010519	.00106	.0011573	.0009234	.000923	.0008645	.0010521
20000	20	29.60881	.1	.0005209	.000549	.000758	.0007616	.000841	.0006617	.0006616	.000549	.000758
7500	3	29.60881	.1	.0020205	.0021768	.0021627	.0021852	.0020125	.0016529	.0016412	.0021768	.0021627
12500	5	29.60881	.1	.0013596	.0015044	.0016304	.0016472	.0015991	.0012748	.0012732	.0015044	.0016304
17500	7	29.60881	.1	.0009989	.0011411	.0013268	.0013515	.0013457	.0010462	.0010457	.0011411	.0013268
25000	10	29.60881	.1	.0007662	.0008724	.0011546	.001172	.0011282	.0009126	.0009125	.0008725	.0011546
50000	20	29.60881	.1	.0004873	.0005315	.0007814	.0007901	.000794	.0006165	.0006165	.0005315	.0007814

Power

ssl	ssh	tsq1	cb	MH	MHfe	MHdl	MHbdl	P	Pdl	Pbdl	Pbdl	Pbdl
1500	3	0	.5	94.2	93.5	85.7	80.7	94.4	88.1	84.6	93.5	85.7
2500	5	0	.5	99.6	99.4	97.3	96.4	99.6	97.8	97.1	99.4	97.3
3500	7	0	.5	100	100	99.8	99.7	100	99.7	99.6	100	99.8
5000	10	0	.5	100	100	100	100	100	100	100	100	100
10000	20	0	.5	100	100	100	100	100	100	100	100	100
3000	3	0	.5	99.7	99.7	97.4	95	99.7	97.7	96	99.7	97.4

Create PDF in your applications with the Pdfcrowd HTML to PDF API PDFCROWD

5000	5	0	.5	100	100	100	99.8	100	100	99.8	100	100
7000	7	0	.5	100	100	100	100	100	100	100	100	100
10000	10	0	.5	100	100	100	100	100	100	100	100	100
20000	20	0	.5	100	100	100	100	100	100	100	100	100
7500	3	0	.5	100	100	99.9	99.6	100	99.9	99.4	100	99.9
12500	5	0	.5	100	100	100	100	100	100	100	100	100
17500	7	0	.5	100	100	100	100	100	100	100	100	100
25000	10	0	.5	100	100	100	100	100	100	100	100	100
50000	20	0	.5	100	100	100	100	100	100	100	100	100
1500	3	0	.1	42.7	27	26.3	24.9	47	44	42.1	27	26.3
2500	5	0	.1	71.7	48.8	46.5	44.2	73.8	70.4	67.6	48.8	46.5
3500	7	0	.1	86.3	65.1	63.8	62.6	88.4	86.3	85	65.1	63.8
5000	10	0	.1	95.7	80.6	80.5	79.4	96.4	95.5	94.5	80.6	80.5
10000	20	0	.1	100	98	98.1	98	100	100	100	98	98.1
3000	3	0	.1	82.7	74.5	65.3	61.1	83.2	77.8	75.3	74.5	65.3
5000	5	0	.1	95.1	90.4	85.8	82.7	95.5	93	91.3	90.4	85.8
7000	7	0	.1	99	98	95.8	94.9	99.1	98.7	98.3	98	95.8
10000	10	0	.1	100	99.6	99.1	99.1	100	99.8	99.8	99.6	99.1
20000	20	0	.1	100	100	100	100	100	100	100	100	100
7500	3	0	.1	99.7	99.4	94.2	91.8	99.7	97.7	96.5	99.4	94.2
12500	5	0	.1	99.9	99.9	99.5	99.2	99.9	99.9	99.5	99.9	99.5
17500	7	0	.1	100	100	100	100	100	100	100	100	100
25000	10	0	.1	100	100	100	100	100	100	100	100	100
50000	20	0	.1	100	100	100	100	100	100	100	100	100
1500	3	.822467	.5	60.6	52.9	32.3	29.6	60.9	37.6	35.1	52.9	32.3
2500	5	.822467	.5	63	56.4	39.6	37.4	63.5	41.6	40.6	56.4	39.6
3500	7	.822467	.5	67	58	42.2	40.7	67.2	42.5	42.3	58	42.2
5000	10	.822467	.5	73.7	63	55.3	54.9	73.8	54.4	54.2	63	55.3
10000	20	.822467	.5	83.1	73.5	79.4	79.2	83.5	79.3	79.5	73.5	79.4
3000	3	.822467	.5	66.8	62.6	35.6	32.9	66.9	39.1	37	62.6	35.6
5000	5	.822467	.5	72.7	67.2	45.4	43.1	72.7	44.5	44.4	67.2	45.4
7000	7	.822467	.5	73.9	67.4	49.6	47.9	74	46.9	46.1	67.4	49.6
10000	10	.822467	.5	77.8	71	59.2	59	78	54.8	53.9	71	59.2
20000	20	.822467	.5	87.1	80.4	86.4	86	87.2	83	83.1	80.4	86.4
7500	3	.822467	.5	71.1	68	37.2	34	71.2	39	36.7	68	37.2
12500	5	.822467	.5	75.3	69.8	45.8	43.3	75.3	43.7	43	69.8	45.8
17500	7	.822467	.5	80.8	75.5	56.4	55.1	80.8	51.9	51.4	75.5	56.4
25000	10	.822467	.5	83	78	67.9	67	83	62.1	62.8	78	67.9
50000	20	.822467	.5	90.6	86	91.6	91.4	90.6	86.6	86.9	86	91.6
1500	3	.822467	.1	35.2	22	20.7	19.8	38.6	33.4	31.5	22	20.7
2500	5	.822467	.1	40.8	21.2	19.8	19	42.5	32.6	31.5	21.2	19.8
3500	7	.822467	.1	49.7	23.4	22.6	22.1	51.4	38.7	37.3	23.4	22.6
5000	10	.822467	.1	54	20.2	22.2	22	56.2	38.9	38	20.2	22.2
10000	20	.822467	.1	65.7	21.7	35.6	35.7	68.2	50.3	50.2	21.7	35.6
3000	3	.822467	.1	50.2	34.5	25.5	23.5	50.8	35.9	34	34.5	25.5
5000	5	.822467	.1	60.4	38.4	30.9	29.4	61.1	41.3	40.6	38.4	30.9
7000	7	.822467	.1	65.1	38	35.2	33.9	65.3	42.3	41.9	38	35.2
10000	10	.822467	.1	67.6	35.9	37.7	37.4	68.1	43.4	42.7	35.9	37.7
20000	20	.822467	.1	79.1	37	63	62.9	79.4	55.9	56.5	37	63
7500	3	.822467	.1	63.8	54.5	36.4	33.8	63.8	45.3	44.1	54.5	36.4
12500	5	.822467	.1	70.3	53.1	39.9	37.9	70.4	42.6	41.9	53.1	39.9
17500	7	.822467	.1	72.4	51.1	44.2	43	72.5	42.3	41.5	51.1	44.2
25000	10	.822467	.1	79.7	52.6	57.2	56.2	79.9	47.8	47.7	52.6	57.2

Create PDF in your applications with the Pdfcrowd HTML to PDF API PDFCROWD

50000	20	.822467	.1	87	52.1	81.9	81.8	87.2	59.4	59.3	52.1	81.9
1500	3	3.289868	.5	42.6	34.7	21.8	19.8	42.7	25.2	24.7	34.7	21.8
2500	5	3.289868	.5	34.7	24.9	17.1	14.8	34.9	17.4	16.8	24.9	17.1
3500	7	3.289868	.5	31.5	19.7	17.3	16.3	31.6	16.3	16.1	19.7	17.3
5000	10	3.289868	.5	26.4	16.6	20.5	19.5	26.9	16.7	16.8	16.6	20.5
10000	20	3.289868	.5	20.3	8.8	28.4	28.1	20.7	20.6	20.9	8.8	28.4
3000	3	3.289868	.5	43.5	35.6	20.4	17.6	43.7	22.5	21.9	35.6	20.4
5000	5	3.289868	.5	40.6	30.1	20.3	18.3	40.8	18.7	18.3	30.1	20.3
7000	7	3.289868	.5	34.9	25	21	19.3	34.9	16.4	16.2	25	21
10000	10	3.289868	.5	30.8	19.5	24.6	23.2	30.9	17.6	17.5	19.5	24.6
20000	20	3.289868	.5	23.3	12.8	37.6	37.2	23.6	25	25.4	12.8	37.6
7500	3	3.289868	.5	50.4	43.7	23.4	19.4	50.5	25.2	24.4	43.7	23.4
12500	5	3.289868	.5	42.1	33.3	23.4	21.1	42.2	20	19.9	33.3	23.4
17500	7	3.289868	.5	38.7	29.3	28.1	25.2	38.7	19.9	19.3	29.3	28.1
25000	10	3.289868	.5	35.1	23.5	29.9	29	35.3	18.4	18.3	23.5	29.9
50000	20	3.289868	.5	26.3	14.6	43.5	42.8	26.3	24.4	24.3	14.6	43.5
1500	3	3.289868	.1	28.5	16.8	16.3	15.5	30.3	24	23.3	16.8	16.3
2500	5	3.289868	.1	26	11	10.3	10.1	28	19.3	18.6	11	10.3
3500	7	3.289868	.1	21.7	7.3	7.6	7.1	22.8	12.7	12.5	7.3	7.6
5000	10	3.289868	.1	18.2	4.5	6.1	6.1	20	9.8	9.9	4.5	6.1
10000	20	3.289868	.1	12.5	.9	4.5	4.5	13.9	3.2	3.2	.9	4.5
3000	3	3.289868	.1	35.5	24.3	19.1	18.1	35.8	25.4	25	24.3	19.1
5000	5	3.289868	.1	34.3	17.1	13.9	12.9	34.6	19	18.7	17.1	13.9
7000	7	3.289868	.1	30.4	11	12.3	11.9	30.8	13.7	13.3	11	12.3
10000	10	3.289868	.1	25.8	7.3	12.3	11.9	26.7	10.9	10.9	7.3	12.3
20000	20	3.289868	.1	18.9	2.8	14.6	14.6	19.4	4.9	4.9	2.8	14.6
7500	3	3.289868	.1	47.3	33	22.8	20.1	47.4	27.5	27.1	33	22.8
12500	5	3.289868	.1	37.1	19.8	18	15.6	37.1	17.8	17.7	19.8	18
17500	7	3.289868	.1	34.2	14.6	17	15.1	34.3	12.4	12	14.6	17
25000	10	3.289868	.1	32.8	11.9	22.5	21.1	33.1	11.8	11.8	11.9	22.5
50000	20	3.289868	.1	24.2	2.6	29.2	28.8	24.2	5.3	5.5	2.6	29.2
1500	3	29.60881	.5	22.8	17.3	12.1	11.4	22.9	14.5	14.3	17.3	12.1
2500	5	29.60881	.5	13.4	10.5	6.6	5.9	13.7	6.4	6.4	10.5	6.6
3500	7	29.60881	.5	8.7	6.5	6.2	5.6	8.7	5.1	5	6.5	6.2
5000	10	29.60881	.5	4.1	3.3	4.6	3.9	4.2	1.7	1.8	3.3	4.6
10000	20	29.60881	.5	.7	.5	3.7	3.4	.7	1.1	1	.5	3.7
3000	3	29.60881	.5	26	21.1	14.4	13	26	16.8	16.3	21.1	14.4
5000	5	29.60881	.5	15.2	14	9	7.6	15.4	7.7	7.8	14	9
7000	7	29.60881	.5	9.7	7.7	7.4	6.3	9.8	5.5	5.3	7.7	7.4
10000	10	29.60881	.5	3.5	2.4	5.7	5.3	3.5	1.8	1.9	2.4	5.7
20000	20	29.60881	.5	.5	.3	5.8	5.7	.6	.9	.9	.3	5.8
7500	3	29.60881	.5	28.3	24.9	16.4	12.3	28.4	17.9	17.2	24.9	16.4
12500	5	29.60881	.5	16.2	11.9	10.6	8.1	16.2	7.7	7.8	11.9	10.6
17500	7	29.60881	.5	10.8	7.6	11.2	7.9	10.9	4.5	4.3	7.6	11.2
25000	10	29.60881	.5	5.3	4.9	10.7	9.3	5.3	2.3	2.3	4.9	10.7
50000	20	29.60881	.5	.7	.3	11.2	11.1	.7	1	1	.3	11.2
1500	3	29.60881	.1	18.5	13.1	12.2	12.2	20	15.7	15.4	13.1	12.2
2500	5	29.60881	.1	10.2	5.8	5.5	5.5	11.4	7.8	7.8	5.8	5.5
3500	7	29.60881	.1	5.2	3.1	2.6	2.6	6	3.1	3	3.1	2.6
5000	10	29.60881	.1	2.6	1.2	1.1	1.2	2.8	1.4	1.4	1.2	1.1
10000	20	29.60881	.1	.2	0	.4	.3	.5	0	0	0	.4
3000	3	29.60881	.1	21.7	14.5	12.8	12.2	22.2	15.4	15.3	14.5	12.8
5000	5	29.60881	.1	13.2	6.1	5.2	4.8	13.4	6.1	6.1	6.1	5.2

Create PDF in your applications with the Pdfcrowd HTML to PDF API PDFCROWD

7000	7	29.60881	.1	9.1	4.8	3.8	3.5	9.2	3.6	3.6	4.8	3.8
10000	10	29.60881	.1	3.7	2.2	2	1.8	3.9	1.1	1.1	2.2	2
20000	20	29.60881	.1	1	.2	1	.9	1.1	.2	.2	.2	1
7500	3	29.60881	.1	27.7	20.5	14.4	13.2	27.7	17.9	17.4	20.5	14.4
12500	5	29.60881	.1	15.4	11	8.2	7	15.5	7.7	7.5	11	8.2
17500	7	29.60881	.1	8.5	5.5	5	4.3	8.6	3	2.9	5.5	5
25000	10	29.60881	.1	4	2.2	4	2.9	4	1	1	2.2	4
50000	20	29.60881	.1	.7	0	3.5	3.4	.7	0	0	0	3.5

SE of power

ssl	ssh	tsq1	cb	MH	MHfe	MHdl	MHbdl	P	Pdl	Pbdl	Pbdl	Pbdl
1500	3	0	.5	.7391617	.7795832	1.107028	1.248002	.7270763	1.023909	1.14142	.7795832	1.107028
2500	5	0	.5	.1995996	.244213	.5125524	.589101	.1995996	.4638534	.5306505	.244213	.5125524
3500	7	0	.5	0	0	.1412799	.1729451	0	.1729451	.1995996	0	.1412799
5000	10	0	.5	0	0	0	0	0	0	0	0	0
10000	20	0	.5	0	0	0	0	0	0	0	0	0
3000	3	0	.5	.1729451	.1729451	.5032296	.6892024	.1729451	.4740359	.6196773	.1729451	.5032296
5000	5	0	.5	0	0	0	.1412799	0	0	.1412799	0	0
7000	7	0	.5	0	0	0	0	0	0	0	0	0
10000	10	0	.5	0	0	0	0	0	0	0	0	0
20000	20	0	.5	0	0	0	0	0	0	0	0	0
7500	3	0	.5	0	0	.09995	.1995996	0	.09995	.244213	0	.09995
12500	5	0	.5	0	0	0	0	0	0	0	0	0
17500	7	0	.5	0	0	0	0	0	0	0	0	0
25000	10	0	.5	0	0	0	0	0	0	0	0	0
50000	20	0	.5	0	0	0	0	0	0	0	0	0
1500	3	0	.1	1.564196	1.403923	1.392232	1.367476	1.57829	1.569713	1.561278	1.403923	1.392232
2500	5	0	.1	1.424468	1.580683	1.57726	1.570465	1.390525	1.443551	1.479946	1.580683	1.57726
3500	7	0	.1	1.087341	1.507312	1.519724	1.530111	1.01264	1.087341	1.129159	1.507312	1.519724
5000	10	0	.1	.6414905	1.250456	1.252897	1.278921	.589101	.6555532	.7209369	1.250456	1.252897
10000	20	0	.1	0	.4427189	.4317291	.4427189	0	0	0	.4427189	.4317291
3000	3	0	.1	1.196123	1.378314	1.505294	1.541684	1.182269	1.314215	1.363785	1.378314	1.505294
5000	5	0	.1	.6826346	.9315793	1.103793	1.196123	.6555532	.8068457	.8912407	.9315793	1.103793
7000	7	0	.1	.3146427	.4427189	.6343185	.6956939	.2986469	.3582039	.4087909	.4427189	.6343185
10000	10	0	.1	0	.1995996	.2986469	.2986469	0	.1412799	.1412799	.1995996	.2986469
20000	20	0	.1	0	0	0	0	0	0	0	0	0
7500	3	0	.1	.1729451	.244213	.7391617	.8676174	.1729451	.4740359	.5811626	.244213	.7391617
12500	5	0	.1	.09995	.09995	.2230471	.2817091	.09995	.09995	.2230471	.09995	.2230471
17500	7	0	.1	0	0	0	0	0	0	0	0	0
25000	10	0	.1	0	0	0	0	0	0	0	0	0
50000	20	0	.1	0	0	0	0	0	0	0	0	0
1500	3	.822467	.5	1.545199	1.578477	1.478753	1.443551	1.54311	1.531744	1.509301	1.578477	1.478753
2500	5	.822467	.5	1.526761	1.568133	1.546557	1.530111	1.522416	1.558666	1.552946	1.568133	1.546557
3500	7	.822467	.5	1.486943	1.560769	1.561781	1.553548	1.484641	1.56325	1.562277	1.560769	1.561781
5000	10	.822467	.5	1.392232	1.526761	1.572231	1.573528	1.390525	1.575005	1.575551	1.526761	1.572231
10000	20	.822467	.5	1.18507	1.395618	1.278921	1.283495	1.173776	1.281214	1.276617	1.395618	1.278921

Create PDF in your applications with the Pdfcrowd HTML to PDF API PDFCROWD

3000	3	.822467	.5	1.489215	1.530111	1.514147	1.485796	1.488083	1.54311	1.526761	1.530111	1.514147
5000	5	.822467	.5	1.408797	1.484641	1.574433	1.566011	1.408797	1.571544	1.571191	1.484641	1.574433
7000	7	.822467	.5	1.388809	1.482309	1.581088	1.579744	1.387083	1.578097	1.576322	1.482309	1.581088
10000	10	.822467	.5	1.314215	1.434922	1.554143	1.555313	1.309962	1.573836	1.576322	1.434922	1.554143
20000	20	.822467	.5	1.059995	1.255325	1.083993	1.097269	1.056485	1.187855	1.18507	1.255325	1.083993
7500	3	.822467	.5	1.433454	1.475127	1.52845	1.497999	1.431978	1.542401	1.524175	1.475127	1.52845
12500	5	.822467	.5	1.363785	1.451882	1.575551	1.566879	1.363785	1.568538	1.565567	1.451882	1.575551
17500	7	.822467	.5	1.245536	1.360055	1.568133	1.572892	1.245536	1.579997	1.580519	1.360055	1.568133
25000	10	.822467	.5	1.187855	1.309962	1.476343	1.486943	1.187855	1.534141	1.52845	1.309962	1.476343
50000	20	.822467	.5	.9228434	1.097269	.8771773	.886589	.9228434	1.077237	1.066954	1.097269	.8771773
1500	3	.822467	.1	1.510285	1.309962	1.281214	1.260143	1.539493	1.491456	1.468928	1.309962	1.281214
2500	5	.822467	.1	1.554143	1.292501	1.260143	1.240564	1.56325	1.482309	1.468928	1.292501	1.260143
3500	7	.822467	.1	1.58111	1.33882	1.322588	1.312094	1.580519	1.540231	1.529284	1.33882	1.322588
5000	10	.822467	.1	1.576071	1.26963	1.314215	1.309962	1.568936	1.541684	1.534927	1.26963	1.314215
10000	20	.822467	.1	1.50117	1.303499	1.514147	1.515094	1.472671	1.58111	1.581126	1.303499	1.514147
3000	3	.822467	.1	1.581126	1.503246	1.378314	1.340802	1.580936	1.516967	1.497999	1.503246	1.378314
5000	5	.822467	.1	1.546557	1.537999	1.461229	1.440708	1.541684	1.55702	1.552946	1.537999	1.461229
7000	7	.822467	.1	1.507312	1.534927	1.510285	1.496927	1.505294	1.562277	1.560253	1.534927	1.510285
10000	10	.822467	.1	1.479946	1.516967	1.53255	1.530111	1.473903	1.567303	1.564196	1.516967	1.53255
20000	20	.822467	.1	1.285764	1.526761	1.526761	1.527609	1.278921	1.570092	1.567721	1.526761	1.526761
7500	3	.822467	.1	1.519724	1.574722	1.521526	1.495848	1.519724	1.574138	1.570092	1.574722	1.521526
12500	5	.822467	.1	1.44496	1.578097	1.548544	1.534141	1.443551	1.563726	1.560253	1.578097	1.548544
17500	7	.822467	.1	1.413591	1.580756	1.570465	1.565567	1.412002	1.562277	1.558124	1.580756	1.570465
25000	10	.822467	.1	1.271971	1.579	1.56466	1.568936	1.267277	1.579608	1.579465	1.579	1.56466
50000	20	.822467	.1	1.063485	1.579744	1.217534	1.220148	1.056485	1.552946	1.553548	1.579744	1.217534
1500	3	3.289868	.5	1.563726	1.505294	1.305665	1.260143	1.564196	1.372938	1.363785	1.505294	1.305665
2500	5	3.289868	.5	1.505294	1.367476	1.190626	1.122925	1.507312	1.198849	1.182269	1.367476	1.190626
3500	7	3.289868	.5	1.468928	1.25774	1.196123	1.168037	1.470184	1.168037	1.162235	1.25774	1.196123
5000	10	3.289868	.5	1.39393	1.176622	1.276617	1.252897	1.40228	1.179453	1.182269	1.176622	1.276617
10000	20	3.289868	.5	1.271971	.8958571	1.425987	1.421404	1.281214	1.278921	1.285764	.8958571	1.425987
3000	3	3.289868	.5	1.567721	1.514147	1.2743	1.204259	1.568538	1.320511	1.307819	1.514147	1.2743
5000	5	3.289868	.5	1.552946	1.450514	1.271971	1.222747	1.554143	1.233009	1.222747	1.450514	1.271971
7000	7	3.289868	.5	1.507312	1.369306	1.288022	1.248002	1.507312	1.170914	1.165144	1.369306	1.288022
10000	10	3.289868	.5	1.459918	1.252897	1.361925	1.334826	1.461229	1.204259	1.201561	1.252897	1.361925
20000	20	3.289868	.5	1.336828	1.056485	1.531744	1.52845	1.342773	1.369306	1.376532	1.056485	1.531744
7500	3	3.289868	.5	1.581088	1.568538	1.33882	1.250456	1.58106	1.372938	1.358175	1.568538	1.33882
12500	5	3.289868	.5	1.561278	1.490339	1.33882	1.290267	1.561781	1.264911	1.262533	1.490339	1.33882
17500	7	3.289868	.5	1.540231	1.439274	1.421404	1.372938	1.540231	1.262533	1.248002	1.439274	1.421404
25000	10	3.289868	.5	1.509301	1.340802	1.447753	1.434922	1.511261	1.225333	1.222747	1.340802	1.447753
50000	20	3.289868	.5	1.392232	1.11662	1.567721	1.56466	1.392232	1.358175	1.356285	1.11662	1.567721
1500	3	3.289868	.1	1.427498	1.182269	1.168037	1.144443	1.453241	1.350555	1.336828	1.182269	1.168037
2500	5	3.289868	.1	1.387083	.9894443	.9612024	.9528851	1.419859	1.248002	1.230463	.9894443	.9612024
3500	7	3.289868	.1	1.303499	.8226239	.8379976	.8121515	1.32671	1.052953	1.045825	.8226239	.8379976
5000	10	3.289868	.1	1.220148	.6555532	.7568289	.7568289	1.264911	.9401915	.9444522	.6555532	.7568289
10000	20	3.289868	.1	1.045825	.2986469	.6555532	.6555532	1.093979	.5565609	.5565609	.2986469	.6555532
3000	3	3.289868	.1	1.513192	1.356285	1.243057	1.217534	1.516034	1.376532	1.369306	1.356285	1.243057
5000	5	3.289868	.1	1.50117	1.190626	1.093979	1.059995	1.504274	1.240564	1.233009	1.190626	1.093979
7000	7	3.289868	.1	1.454593	.9894443	1.03861	1.023909	1.459918	1.087341	1.07383	.9894443	1.03861
10000	10	3.289868	.1	1.383604	.8226239	1.03861	1.023909	1.398967	.9854897	.9854897	.8226239	1.03861
20000	20	3.289868	.1	1.238059	.5216896	1.11662	1.11662	1.250456	.6826346	.6826346	.5216896	1.11662
7500	3	3.289868	.1	1.578832	1.486943	1.32671	1.267277	1.579	1.412002	1.405557	1.486943	1.32671
12500	5	3.289868	.1	1.527609	1.260143	1.214907	1.147449	1.527609	1.209612	1.206942	1.260143	1.214907
17500	7	3.289868	.1	1.50012	1.11662	1.187855	1.13225	1.50117	1.042228	1.027619	1.11662	1.187855

Create PDF in your applications with the Pdfcrowd HTML to PDF API PDFCROWD

25000	10	3.289868	.1	1.484641	1.023909	1.320511	1.290267	1.488083	1.020176	1.020176	1.023909	1.320511
50000	20	3.289868	.1	1.354385	.5032296	1.437832	1.431978	1.354385	.7084561	.7209369	.5032296	1.437832
1500	3	29.60881	.5	1.32671	1.196123	1.031305	1.005007	1.328755	1.113441	1.107028	1.196123	1.031305
2500	5	29.60881	.5	1.077237	.969407	.7851369	.7451107	1.087341	.7739767	.7739767	.969407	.7851369
3500	7	29.60881	.5	.8912407	.7795832	.7626008	.7270763	.8912407	.6956939	.6892024	.7795832	.7626008
5000	10	29.60881	.5	.6270486	.5648982	.66245	.6122009	.6343185	.4087909	.4204284	.5648982	.66245
10000	20	29.60881	.5	.2636475	.2230471	.5969171	.5730969	.2636475	.3298333	.3146427	.2230471	.5969171
3000	3	29.60881	.5	1.387083	1.290267	1.110243	1.063485	1.387083	1.182269	1.168037	1.290267	1.110243
5000	5	29.60881	.5	1.135324	1.097269	.9049862	.8379976	1.14142	.8430362	.848033	1.097269	.9049862
7000	7	29.60881	.5	.9359006	.8430362	.8277922	.7683163	.9401915	.7209369	.7084561	.8430362	.8277922
10000	10	29.60881	.5	.5811626	.4839835	.7331507	.7084561	.5811626	.4204284	.4317291	.4839835	.7331507
20000	20	29.60881	.5	.2230471	.1729451	.7391617	.7331507	.244213	.2986469	.2986469	.1729451	.7391617
7500	3	29.60881	.5	1.424468	1.367476	1.170914	1.03861	1.425987	1.212266	1.193382	1.367476	1.170914
12500	5	29.60881	.5	1.165144	1.023909	.973468	.8627804	1.165144	.8430362	.848033	1.023909	.973468
17500	7	29.60881	.5	.981509	.8379976	.9972763	.8529889	.9854897	.6555532	.6414905	.8379976	.9972763
25000	10	29.60881	.5	.7084561	.6826346	.9775019	.918428	.7084561	.4740359	.4740359	.6826346	.9775019
50000	20	29.60881	.5	.2636475	.1729451	.9972763	.993373	.2636475	.3146427	.3146427	.1729451	.9972763
1500	3	29.60881	.1	1.227905	1.066954	1.034969	1.034969	1.264911	1.150439	1.14142	1.066954	1.034969
2500	5	29.60881	.1	.957058	.7391617	.7209369	.7209369	1.005007	.848033	.848033	.7391617	.7209369
3500	7	29.60881	.1	.7021111	.5480785	.5032296	.5032296	.7509993	.5480785	.5394442	.5480785	.5032296
5000	10	29.60881	.1	.5032296	.3443254	.3298333	.3443254	.5216896	.3715373	.3715373	.3443254	.3298333
10000	20	29.60881	.1	.1412799	0	.1995996	.1729451	.2230471	0	0	0	.1995996
3000	3	29.60881	.1	1.303499	1.113441	1.056485	1.034969	1.314215	1.14142	1.13838	1.113441	1.056485
5000	5	29.60881	.1	1.070402	.7568289	.7021111	.6759882	1.077237	.7568289	.7568289	.7568289	.7021111
7000	7	29.60881	.1	.9094999	.6759882	.6046156	.5811626	.9139803	.589101	.589101	.6759882	.6046156
10000	10	29.60881	.1	.5969171	.4638534	.4427189	.4204284	.6122009	.3298333	.3298333	.4638534	.4427189
20000	20	29.60881	.1	.3146427	.1412799	.3146427	.2986469	.3298333	.1412799	.1412799	.1412799	.3146427
7500	3	29.60881	.1	1.415171	1.276617	1.110243	1.070402	1.415171	1.212266	1.198849	1.276617	1.110243
12500	5	29.60881	.1	1.14142	.9894443	.8676174	.8068457	1.144443	.8430362	.8329166	.9894443	.8676174
17500	7	29.60881	.1	.8819014	.7209369	.6892024	.6414905	.886589	.5394442	.5306505	.7209369	.6892024
25000	10	29.60881	.1	.6196773	.4638534	.6196773	.5306505	.6196773	.3146427	.3146427	.4638534	.6196773
50000	20	29.60881	.1	.2636475	0	.5811626	.5730969	.2636475	0	0	0	.5811626

MH: Mantel-Haenszel weighting (fixed-effect)
MHfe: Mantel-Haenszel with fixed-effect weighting (different to MH weighting)
MHdl: Mantel-Haenszel with DL random-effects weighting
MHbdl: Mantel-Haenszel with bootstrapped DL random-effects weighting
P: Peto OR with fixed-effect weighting
Pdl: Peto OR with DL random-effects weighting
Pbdl: Peto OR with bootstrapped DL random-effects weighting

ss1: size for lower level unit (patients)
ssh: size for higher level unit (studies)
tsq1: between study variance for the exposure
cb: probability of membership for the intervention (0.5=balanced design)

Create PDF in your applications with the Pdfcrowd HTML to PDF API PDFCROWD

```
effsize: ln(0.5)
I^2:      0% (tau^2=0), 20% (tau^2=0.822467), 50% (tau^2=3.289868), 90% (tau^2=29.60881)
```

Create PDF in your applications with the Pdfcrowd HTML to PDF API PDFCROWD
