# Supplementary material for: Applications of simple and accessible methods for meta-analysis involving rare events: A simulation study
Source: Stat Methods Med Res. 2021 Jun 17;30(7):1589–608. doi: 10.1177/09622802211022385 (PMC8411477; doi:10.1177/09622802211022385)
Supplement: sj-pdf-4-smm-10.1177_09622802211022385 - Supplemental material for Applications of simple and accessible methods for meta-analysis involving rare events: A simulation study [file sj-pdf-4-smm-10.1177_09622802211022385.pdf]

Supplementary file for “Applications of simple and accessible methods for meta-analysis involving rare events: A simulation study”

This supplementary file includes results for all additional simulation settings, which were not fully presented in the main paper and were only discussed in comparison to results presented in the manuscript.

Contents

Appendix 1: Literature review 2
Appendix 2: Simulation code 10
Appendix 3: Mean bias 16
Appendix 4: Mean error 21
Appendix 5: Coverage 26
Appendix 6: Power 31
Appendix 7: Convergence 35
Appendix 8: Application of methods in practice 41

Appendix 1: Literature review

Table S1: Descriptive narrative literature review of similar simulations studies of rare events

Study (date)	Journal	Objectives	Data used to inform simulation design	Design considerations	Heterogeneity (or between study heterogeneity) assessment	Methods used to assess	Summary of findings
Kuss 2014	Statistics in medicine	A simulation study to compares statistical methods for meta-analysis that explicitly include information from **double-zero studies** without using continuity corrections	Yes<br><br>Design informed by Cochrane review (Turner et al)	• Proportion of double-zero studies (25%, 50%, and 75%)<br>• Size of treatment effect (OR = 0.684)<br>• Number of studies per meta-analysis (log normal ~ (0.65, 1.2))<br>• Sample size of single study (log-normal ~ (4.615, 1.1))<br>• Random effects variance (FEM, REM)	τ^2 was sampled from the distribution from Turner et al., which is log-normal with mean= **-1.47, SD=1.65, and skewness= -0.55** equivalence to distribution with 25% percentile/median/75% percentile of τ^2 of **0.079/0.274/0.806**	• Treatment as one trial<br>• GLMM<br>• Marginal GEE<br>• Beta-binomial regression<br>• Conditional logistic regression<br>• Logistic regression with fixed study effect and Firth likelihood<br>• Stijnen's bivariate Binomial-Normal model<br>• Cai's Poisson-Gamma models<br>• Stijnen's bivariate Poisson-normal model<br>• Mantel-Haenszel method<br>• Arcsine difference	In meta-analyses with binary outcomes and double-zero or single-zero studies, we recommend to use beta-binomial regression methods to arrive at summary estimates for the odds ratio, the relative risk or the risk difference. Methods that ignore information from double-zero studies or use continuity corrections, should no longer be used.

Cheng 2016	BMJ Open	Evaluate the impact of **including** or **excluding both-armed zero-event** studies in meta-analysis of RCTs with rare outcome events through a simulation study.	Yes<br><br>Unknown	• OR (0.2, 0.5, 0.8, 1, 1.25, 2, 5)<br>• Control group prob (p): 0.001, 0.005, 0.01<br>• 5 studies in each MAs<br>• 50, 100, 250 patients<br>• Between study SD: 0.1, 0.5, 1<br>• Ratio of group size 1:1	Between-study SD was set **as 0.1 (small), 0.5 (moderate), 1 (large)**	• IV random effects<br>• IV fixed effects<br>• M-H random effects<br>• M-H fixed effects<br>• Peto	Recommend excluding BA0E studies in conjunction of Peto method in the following two scenarios: (1) when treatment effects are likely to present and (2) when evaluating harmful outcomes such as mortality, mobility or adverse events.
Sharma 2017	Journal of Clinical Epidemiology	Identify the validity of effect estimates for serious rare adverse events in clinical study reports of antidepressants trials, across different meta-analysis methods	No<br><br>Clinical study reports	70 CSRs, all-cause mortality, suicidality, aggressive behaviour and akathisia	NA	• Peto's OR<br>• GLMM<br>• Conditional logistic regression<br>• Bayesian approach<br>• Beta-binomial	To ensure reduction of bias and erroneous inferences, sensitivity analyses should be performed using different methods instead of the Yusuf-Peto approach, in particular the beta-binomial method, which was shown to be superior through a simulation study.

Lane 2011	Statistical methods in medical research	Review of methods for the meta-analysis of incidence of rare events using summary-level data	No<br><br>Dataset used in a published analysis of cardiovascular safety in rosiglitazone trials	48 trials included	NA	• Risk difference<br>• Relative risk or odds<br>• Bayesian	The common method of adding a correction factor to handle zeroes may introduce bias where the incidence of events is small, as in this case. Alternative analyses on the log-odds scale are shown to give similar results, but the choice between them is less important than the potential sources of bias in any meta-analysis arising from limitations in the underlying dataset
Gunhan 2018	Research Synthesis Methods	Assessment of more complex methods for the meta-analysis of incidence of rare events including **double zero events**	Yes<br><br>Cochrane Database of Systematic Reviews (CDSR)	Simulation scenarios are similar to those considered by Friede 2017<br><br>The datasets are generated under the BNHM, more specifically (1).<br>Numbers of studies (*k* including {2, 3, 5}) and true treatment effects (*theta* = {−5,−4,−3,−2,−1,−0.5, 0, 0.5, 1, 2, 3, 4, 5}) are varied, resulting in a	The degree of heterogeneity (τ) is taken as τ **= 0.28** (moderate heterogeneity), which is the median value of the predictive distribution	• Beta-binomial<br>• MH<br>• MLE<br>• Bayesian vague prior<br>• Bayesian weak informative prior	BNHM with a WIP outperformed a Bayesian method without a WIP and a maximum likelihood estimator in terms of smaller bias and shorter

				total of 39 simulation scenarios. To reflect the rare-event cases, true baseline risks on the probability scale are taken uniformly between 0.005 and 0.05. Following Kuss 2014 a lognormal distribution is fitted to the sample sizes obtained from the CDSR data, resulting in a log-normal distribution with parameters μ = 5 and *sigma* = 1. Hence, sample sizes are generated from LN(5, 12), the minimum sample size is restricted to 2 patients (values below 2 are rounded up to 2), and at least 1 patient in each treatment arm is assumed. The degree of heterogeneity (*tau*) is taken as *tau* = 0.28 (moderate heterogeneity), which is the median value of the predictive distribution for between-study heterogeneity in a meta-analysis as estimated by Turner et al. According to a binomial probability of 0.5, patients were allocated to the treatment groups, thus mimicking randomization. The simulations were carried out with 10,000 replications per scenario.	for between-study heterogeneity in a meta-analysis in a general setting as estimated by Turner et al.		interval estimates with similar coverage.
Efthimiou 2018	BMJ - Evidence based mental health	Present an overview of some approaches suitable for meta-analysing rare events and we provide	No<br><br>Previously published data regarding mortality risk in antipsychotics	Comprised two independent meta-analyses. The first one included 18 trials that compared long-acting injectable antipsychotics (LAI-AP) to placebo, for all-cause mortality. The event was very rare; the	NA	• Peto’s method<br>• M-H with continuity correction (CC)<br>• I-V with CC<br>• Bayesian meta-analysis (random/fixed)<br>• Beta-binomial	Researchers should define the primary analysis a priori, in order to avoid selective reporting. A sensitivity analysis using a range of

		Practical recommendations for their use.		risk of death across both arms in all studies was around 0.2%. Only seven events were reported in the drug arms (total 3774 patients) and six events in the placebo arm (2145 patients).		• Arcsine difference	methods should be used to assess the robustness of results. Suboptimal methods such as using a continuity correction should be avoided.
Cai 2010	Statistics in Medicine	Propose alternative approaches based on Poisson random effects models to make inference about the relative risk between two treatment groups when the event rate is very rare and including **zero event** studies	Yes<br><br>Simulation design informed by real example using data from rosiglitazone data	Sample sizes are generated by sampling the paired sample sizes from the rosiglitazone Data (Nissen et al. N Engl J Med 2007)<br><br>Total number of events for two groups derived from Poisson gamma.<br><br>β chosen to be either 380 or 38 resulting in a very rare baseline event rate of 0.38 per cent or a moderately rare event rate of 3.8 per cent, respectively. _=1.44 and β=380 were chosen to mimic the rosiglitazone study.	We consider = **358 (log(φ)=5.88) and =3.58 (log(φ)=1.28),** representing a small and large between-study variation, respectively.	• Likelihood ratio beta-binomial<br>• LR gamma-beta<br>• M-H FEs (no CC)<br>• M-H FEs (with CC)<br>• DerSimonian-Laird REs (with CC)<br>• Peto's FE<br>• Peto's RE<br>• Bayesian approach<br>• Fixed RR with gamma baseline	We propose alternative approaches based on Poisson random effects models to make inference about the relative risk between two treatment groups. Simulation studies show that the proposed methods perform well when the underlying event rates are low
Bhaumik 2012	Journal of American Statistical Association	The focus of this article is on random-effects meta-analysis for sparse data. We	Yes<br><br>Unclear	• Performance of moment-based estimate of theta	**τ^2 = 0, 0.2, 0.4, 0.6, 1.2**<br><br>The values of mu are varied from -5.5 (0.4%) to 5.5 (99%). For this	• DerSimonian and Laird<br>• Mantel and Henszel<br>• Empirical logit	Recommend the SA with the 0.5 continuity correction for sparse data with

		first look for a **continuity correction** to make our moment-based estimate of the treatment effect asymptotically unbiased for a single study. Next, we extend this concept of bias correction for multiple studies and propose an asymptotically unbiased estimate		• Type I error rates and power functions for testing theta<br>• Performance estimates of tau squared	comparison, we set theta at 0 and the number of studies were set to 20. Sample sizes in each treatment arm were drawn from U(50, 1000)	• A simple (unweighted) average (SA) treatment effect estimate<br>• Parametric bootstrapping test for heterogeneity	heterogeneity. To estimate the heterogeneity parameter, our recommendation is to use the IPM. We recommend the PB for testing the heterogeneity parameter
Li & Wang 2017	Statistical methods in medical research	Recently, Bhaumik et al. developed a simple average estimator for the overall treatment effect based on a random effects model. We consider the mean squared error to assess estimation efficiency and show that SA_0.5 (**continuity correction**) fails to minimize the mean squared error	Yes<br><br>Rosiglitazone meta-analysis (Nissen et al. N Engl J Med 2007)	See Bhaumik 2012 for simulation design	**τ^2 was set to 0.5**	• DerSimonian and Laird<br>• Generalized linear mixed model<br>• Integrated median unbiased estimator (iMUE)<br>• Mantel-Haenszel<br>• Simple average	We find that for large samples, SA_0.5 performs very well: it is the least biased in nearly all the settings examined; it is the most efficient in some settings and always among the most efficient group otherwise; and it has good performance on the type I error and

							coverage rate as well. We also find iMUE and SA_0.25 perform quite well for large samples in terms of all the four measures. In addition, SA_1 can achieve the minimum MSE in some of the settings, which confirms our theoretical results in ‘‘MSE’’ section.
Spittal 2015	BMC medical Research methodology	Conduct a simulation study to compare the inverse-variance method of conducting a meta-analysis (**with** and **without** the continuity correction) with alternative methods	Yes<br><br>Motivating example included a pervious meta-analysis of structural interventions at suicide hotspots (Pirkis 2013)	Simulated datasets were drawn from a Poisson distribution.<br>Our simulations fixed β_{int} at log*(*0.2*)* = −1.609, meaning that the number of events in the intervention group were 80<br>Percent less per unit of time than in the control group.<br><br>**Fixed parameters:**<br>Incidence rate ratio, exp(βint) 0.2<br>Time, intervention group, t1 t1 ~ Uniform(2, 5)	The amount of baseline variability and between-study heterogeneity was varied: considered scenarios where baseline variability was either σ = 0.1 or 1.0 or where the baseline variability was proportionate to the baseline event rate (σ = 0.1 × exp*(β*1*)*).<br><br>Similarly, we examined two values of between-study heterogeneity in the intervention effect, **τ = 0.5 or 2.5.**	• IV method<br>• IV (+0.5)<br>• Poisson regression with fixed effects<br>• Poisson regression with random effects<br>• Bayesian Poisson regression with random effects with inverse-gamma prior for variance parameters<br>• Bayesian Poisson regression with random intervention effects with a half-	Inverse-variance methods perform poorly when the data contains zeros in either the control or intervention arms. Methods based on Poisson regression with random effect terms for the variance components are very flexible offer substantial improvement

				Time, control group, t_0 $t_0 \sim$ Uniform(2, 10)<br>Number of simulated datasets per scenario, Bs<br>500<br>Varied parameters<br>Percent of zero counts in the intervention group, Poisson($\beta_1 * 0.2$)<br>0.09%, 5%, 14%, 37%, 55%, 82%<br>Heterogeneity: control and intervention groups, σ, τ<br>Scenario A (0.1, 0.5)<br>Scenario B (0.1, 2.5)<br>Scenario C (1.0, 0.5)<br>Scenario D (1.0, 2.5)<br>Scenario E ($0.1 \times \beta_1$, 2.5)<br>Number of studies, k 5, 10, 20	In all, five combinations of σ and τ were examined.	Cauchy prior for the variance parameters	

Appendix 2: Simulation code

```
capture program drop mainsim

//core code. simulate one dataset per specifications and run ipdforest and ipdmetan and return the results (coverage, power, etc)

program define mainsim, rclass

	version 14.1

	set maxiter 50

	//inputs

   /*command syntax - identical to ipdpower for now plus publication bias*/

   syntax, sn(integer) ssl(integer) ssh(integer) b0(real) b1(real) b2(real) b3(real) [minsh(integer 50) hpoisson icluster outc(string) cb(real 0.5) cexp cexpd(string) errsd(real 1) /*

	*/ sderrsd(real 0) derr(string) bcov bcb(real 0.5) ccovd(string) slcov model(integer 1) tsq0(real 0) tsq1(real 0) tsq2(real 0) tsq3(real 0) dtp0(string) /*

	*/ dtp1(string) dtp2(string) dtp3(string) covmat(name) missp(real -127.77) mar(real -127.77) mnar(real -127.77) minum(integer -127) mipmm(integer -127) /*

	*/ clvl(real 95) seed(integer -127) nskip dnorm xnodts NODIsplay moreon pubbias(integer 0)]


	/*debug only

	local ssl=5000

	local ssh=20

	local tsq0=0.5

	local tsq1=0.0

	local outc="cont"

	local b0=1.0

	local b1=0.5

	local b2=0.3*/


	//generate a single dataset

	ipdpower, sn(1) ssl(`ssl') ssh(`ssh') b0(`b0') b1(`b1') b2(0) b3(0) outc(binr) model(1) tsq0(0) tsq1(`tsq1') cb(`cb') nodisplay

	//collapse

	qui gen outc2=outcome if outcome==1
```

```
qui gen outc3=outcome if outcome==0
qui collapse (count) den=outc3 num=outc2, by(studyid grp)
qui reshape wide den num, i(studyid) j(grp)

/* not used for this
//if publication bias is modelled then drop some small studies that indicate null effects
if `pubbias'!=0 {
	//percentage of missing studies
	if `pubbias'<1 | `pubbias'>20 {
		di as error "% of publication bias must be between 1 and 20"
		error 197
	}
	tempfile tempf
	qui save `tempf', replace
	//get effects for each study
	local strcont="regress"
	local strbinr="logistic"
	local strcount="poisson"
	forvalues i=1(1)`ssh' {
		qui `str`outc'' outcome i.grp xcovar if studyid==`i'
		local res`i'=_b[1.grp]
	}
	qui collapse (count) patnum=id, by(studyid)
	qui gen eff=.
	forvalues i=1(1)`ssh' {
		qui replace eff=`res`i'' if studyid==`i'
	}
	//sort on size and effect size (smaller)
	sort patnum
	qui egen temp1=seq()
	sort eff
```

```
		qui egen temp2=seq()
		qui replace temp1=temp1+temp2
		sort temp1
		qui egen tempid=seq()
		//keep what is dictated by the input (rounded up so at least one study will be
missing)
		local drnum=ceil(`ssh'*`pubbias'/100)
		qui keep if tempid<=`drnum'
		qui keep studyid
		qui merge 1:m studyid using `tempf'
		qui drop if _merge==3
		qui drop _merge
	}
	//debug
	*di `pubbias'
	*tab studyid
	*/

	//restart scalars
	foreach x in ub1_grp se1_grp ub2_grp se2_grp ub3_grp se3_grp ub4_grp se4_grp /*
	*/ ub5_grp se5_grp ub6_grp se6_grp ub7_grp se7_grp ub8_grp se8_grp ub9_grp se9_grp {
		scalar `x' = .
	}

	//Model 1: MH
	capture metaan num1 den1 num0 den0, mhor
	if _rc==0 {
		//main effect results
		scalar ub1_grp=r(eff)
		scalar se1_grp=sqrt(r(effvar))
	}
```

```
//Model 2: MH with FE weighting
capture metaan num1 den1 num0 den0, mhor fe
if _rc==0 {
	//main effect results
	scalar ub2_grp=r(eff)
	scalar se2_grp=sqrt(r(effvar))
}
//Model 3: MH with DL (IV) weighting
capture metaan num1 den1 num0 den0, mhor dl
if _rc==0 {
	//main effect results
	scalar ub3_grp=r(eff)
	scalar se3_grp=sqrt(r(effvar))
}
//Model 4: MH with BDL (IV) weighting
capture metaan num1 den1 num0 den0, mhor bdl reps(100)
if _rc==0 {
	//main effect results
	scalar ub4_grp=r(eff)
	scalar se4_grp=sqrt(r(effvar))
}
//Model 5: Peto
capture metaan num1 den1 num0 den0, por
if _rc==0 {
	//main effect results
	scalar ub5_grp=r(eff)
	scalar se5_grp=sqrt(r(effvar))
}
//Model X: Peto with FE weighting - identical with the above not used
/*
capture metaan num1 den1 num0 den0, por fe
```

```
if _rc==0 {
	//main effect results
	scalar ub6_grp=r(eff)
	scalar se6_grp=sqrt(r(effvar))
}
*/
//Model 6: Peto with DL (IV) weighting
capture metaan num1 den1 num0 den0, por dl
if _rc==0 {
	//main effect results
	scalar ub6_grp=r(eff)
	scalar se6_grp=sqrt(r(effvar))
}
//Model 7: Peto with BDL (IV) weighting
capture metaan num1 den1 num0 den0, por bdl reps(100)
if _rc==0 {
	//main effect results
	scalar ub7_grp=r(eff)
	scalar se7_grp=sqrt(r(effvar))
}
//Model 8: FE
capture metan num1 den1 num0 den0, or fixedi nograph
if _rc==0 {
	//main effect results
	scalar ub8_grp=ln(r(ES))
	scalar se8_grp=r(selogES)
}
//Model 9: IV RE
capture metan num1 den1 num0 den0, or randomi nograph
if _rc==0 {
	//main effect results
```

```
		scalar ub9_grp=ln(r(ES))
		scalar se9_grp=r(selogES)
	}

	//return all results to caller
	foreach x in ub1_grp se1_grp ub2_grp se2_grp ub3_grp se3_grp ub4_grp se4_grp /*
	*/ ub5_grp se5_grp ub6_grp se6_grp ub7_grp se7_grp ub8_grp se8_grp ub9_grp se9_grp {
		return scalar `x'=`x'
	}
end
```

Appendix 3: Mean bias

Figure S1: Mean bias for rare events with balanced allocation (r=0.5)

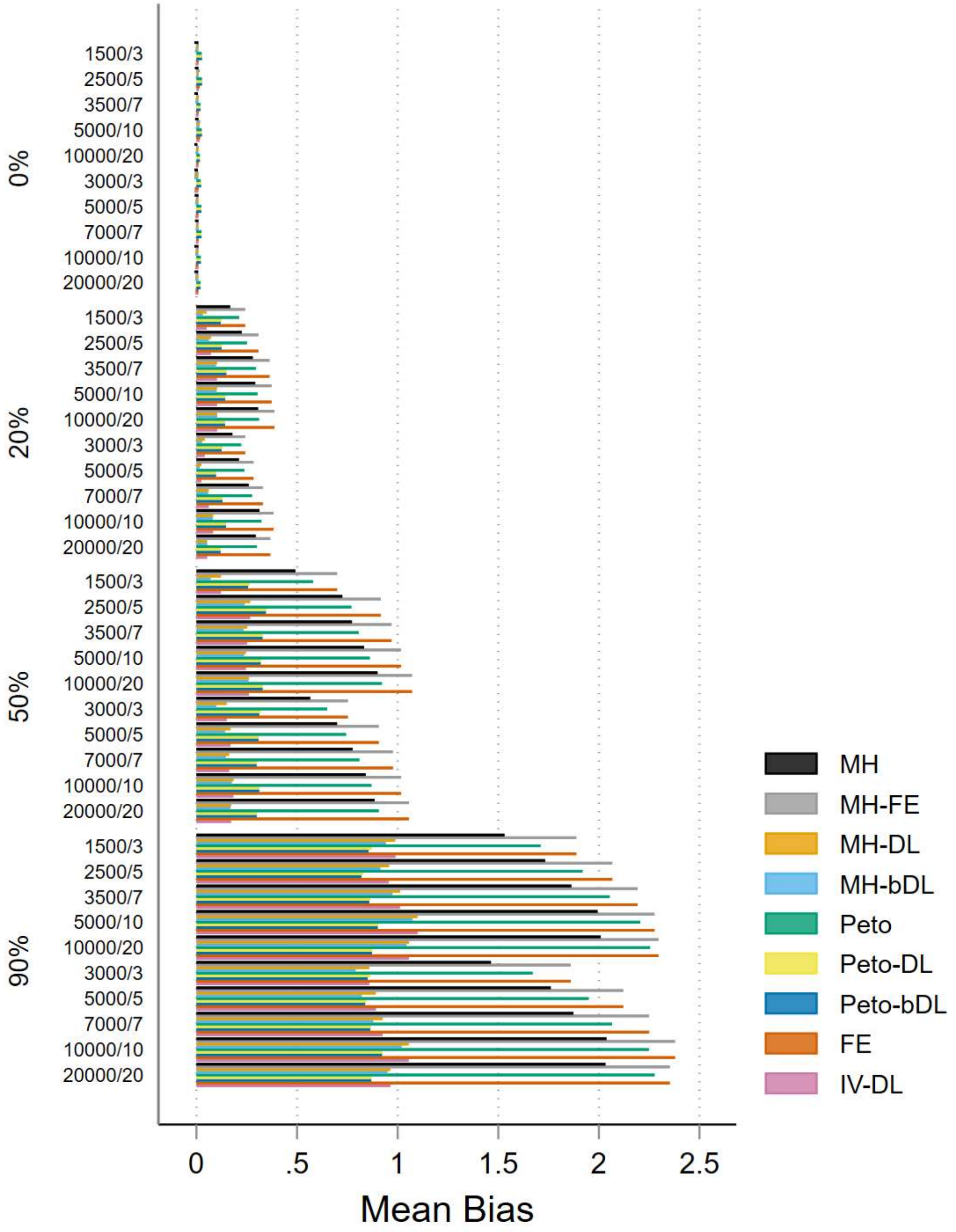

Figure S2: Mean bias in very rare events for imbalanced allocation (r=0.1)

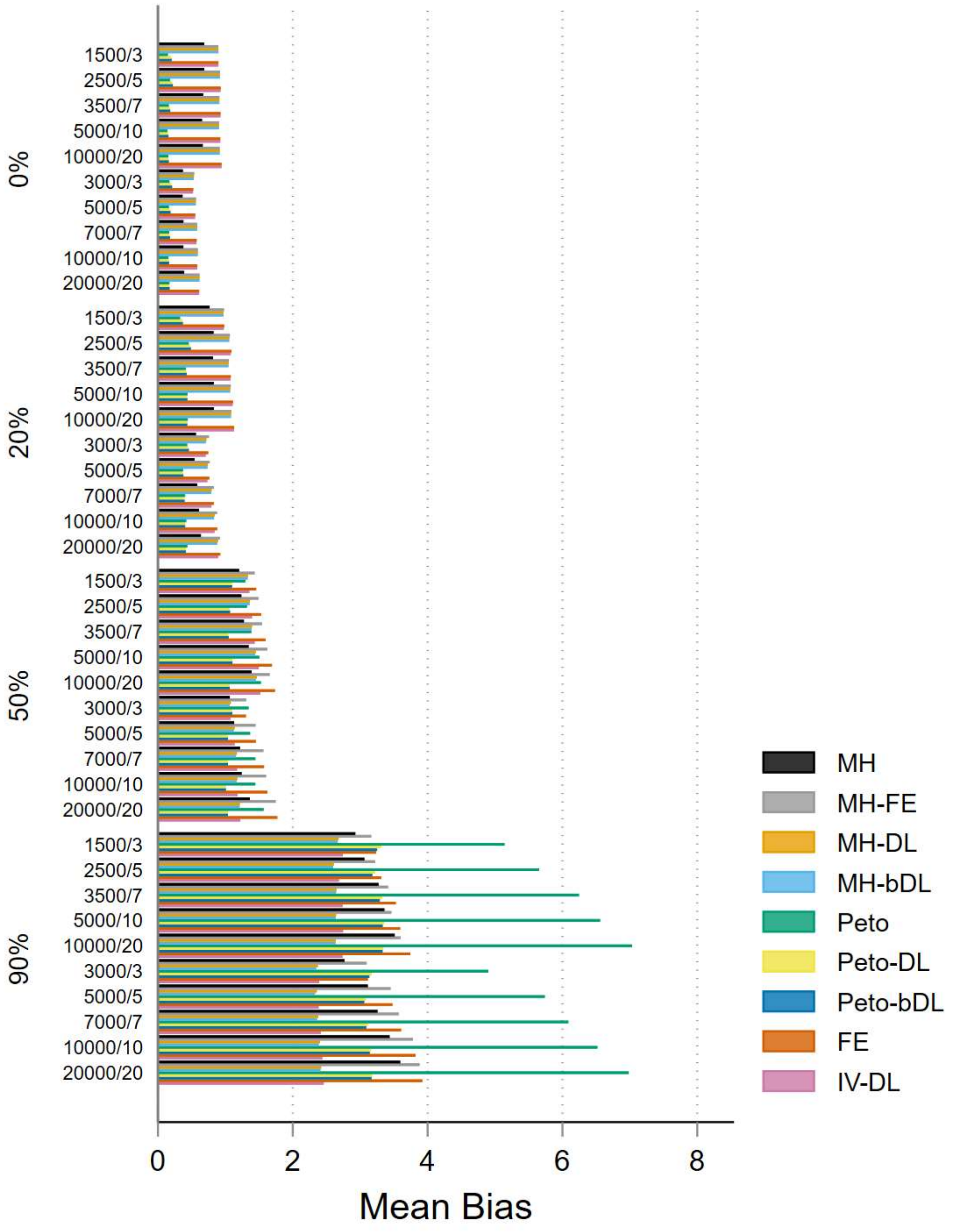

Figure S3: Mean bias for very rare events with balanced allocation (r=0.5)

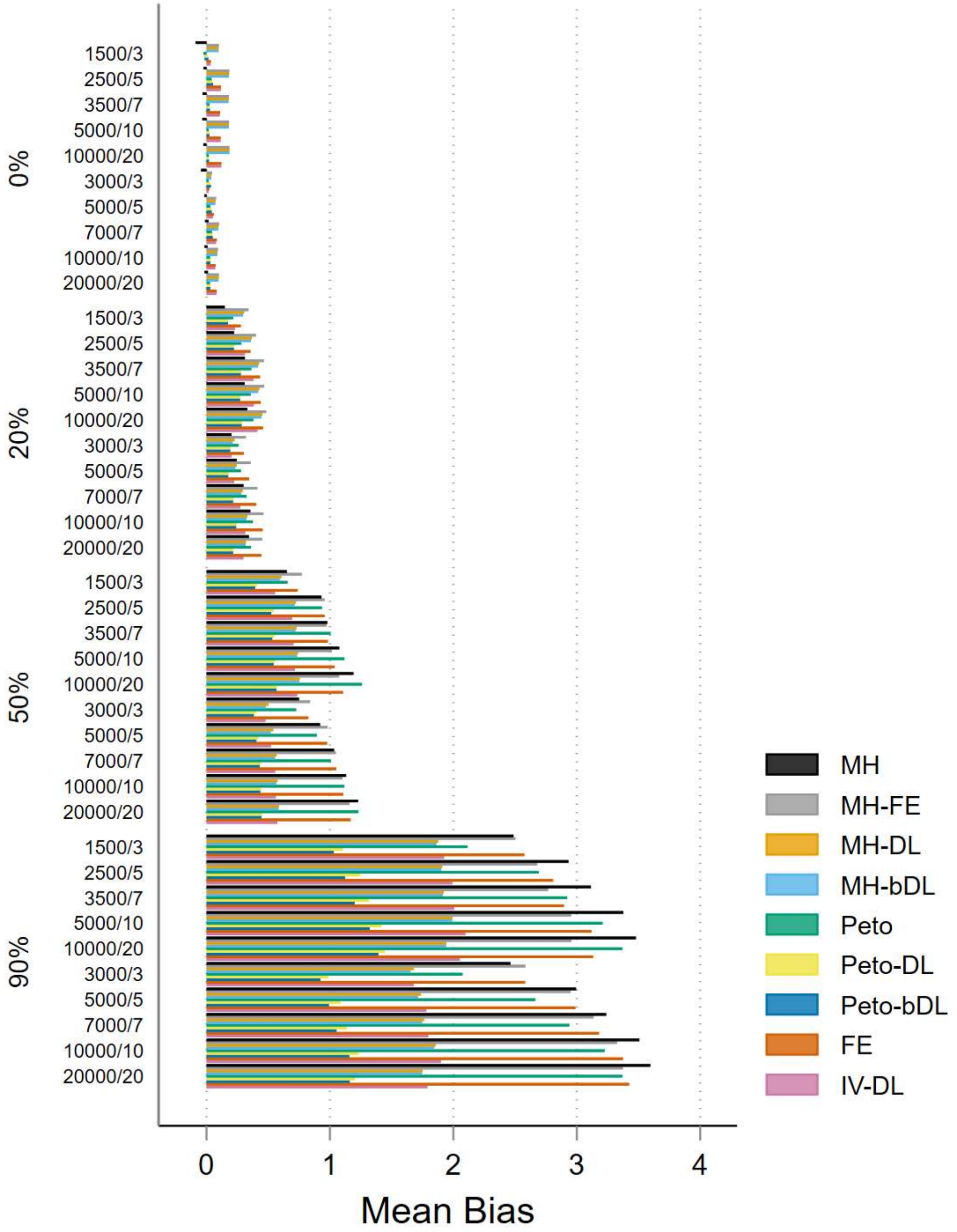

Figure S4: Mean bias of non-rare events for imbalanced allocation (r=0.1)

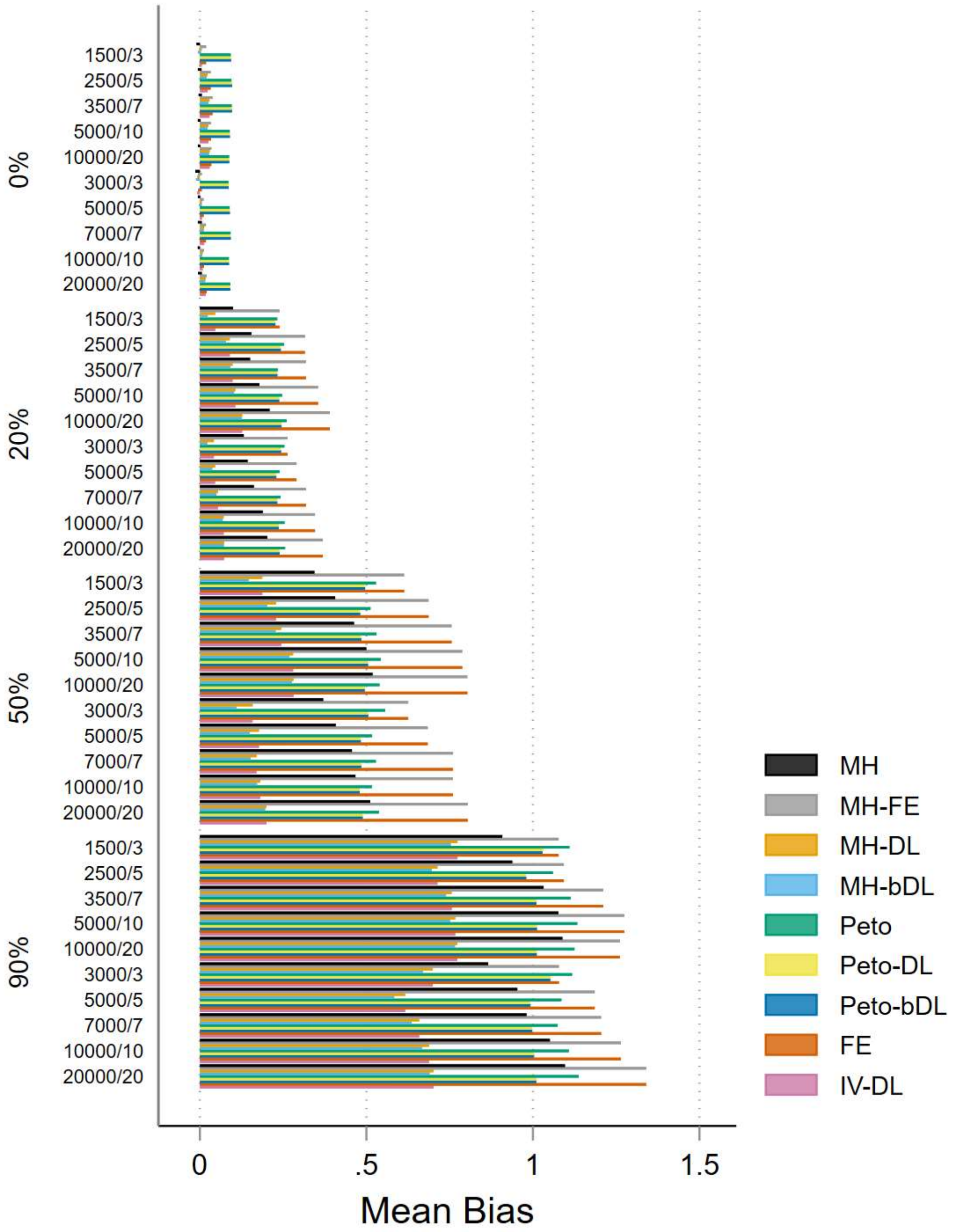

Figure S5: Mean bias of non-rare events with balanced allocation (r=0.5)

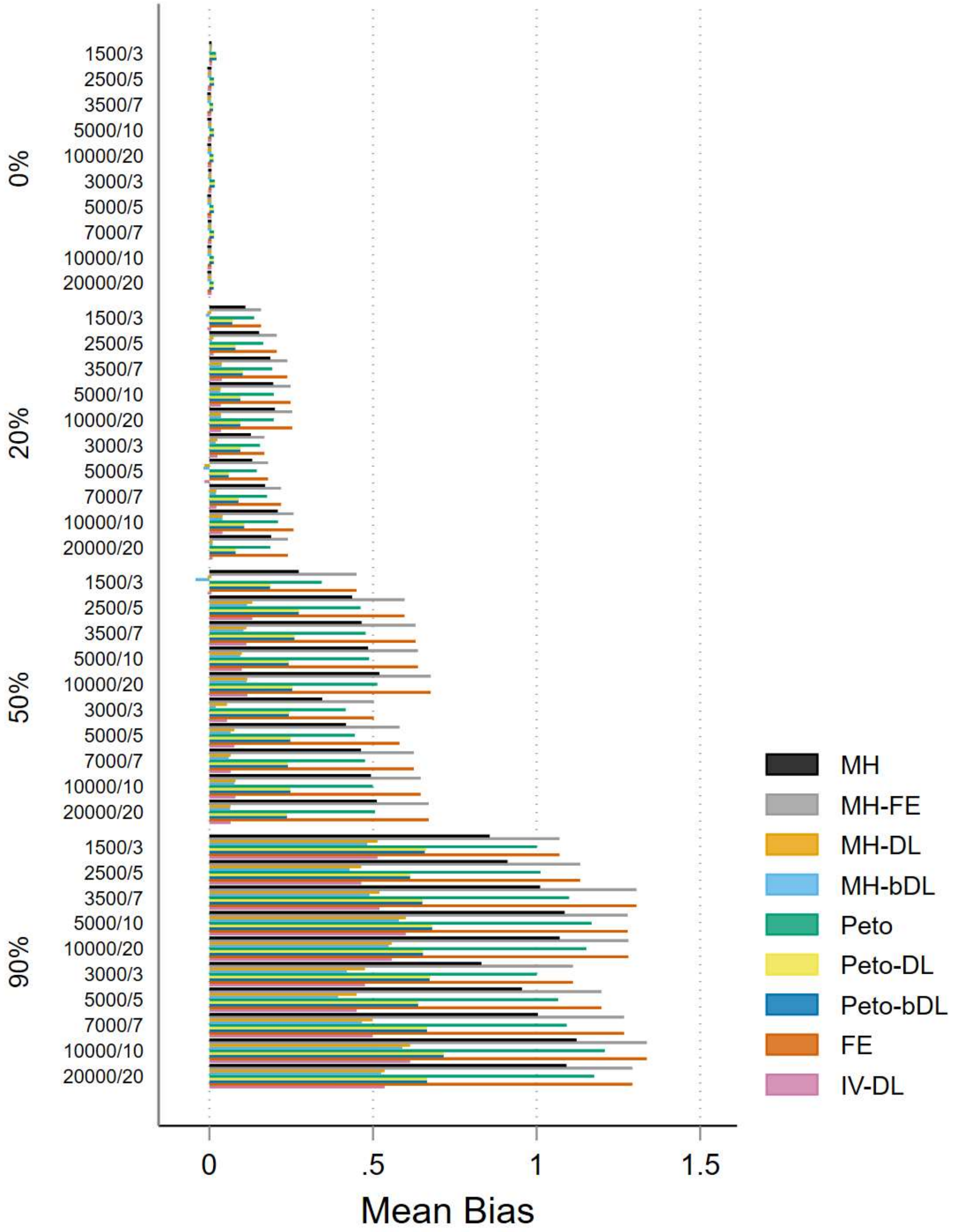

Appendix 4: Mean error

Figure S6: Mean error for rare events with imbalanced allocation (r=0.1)

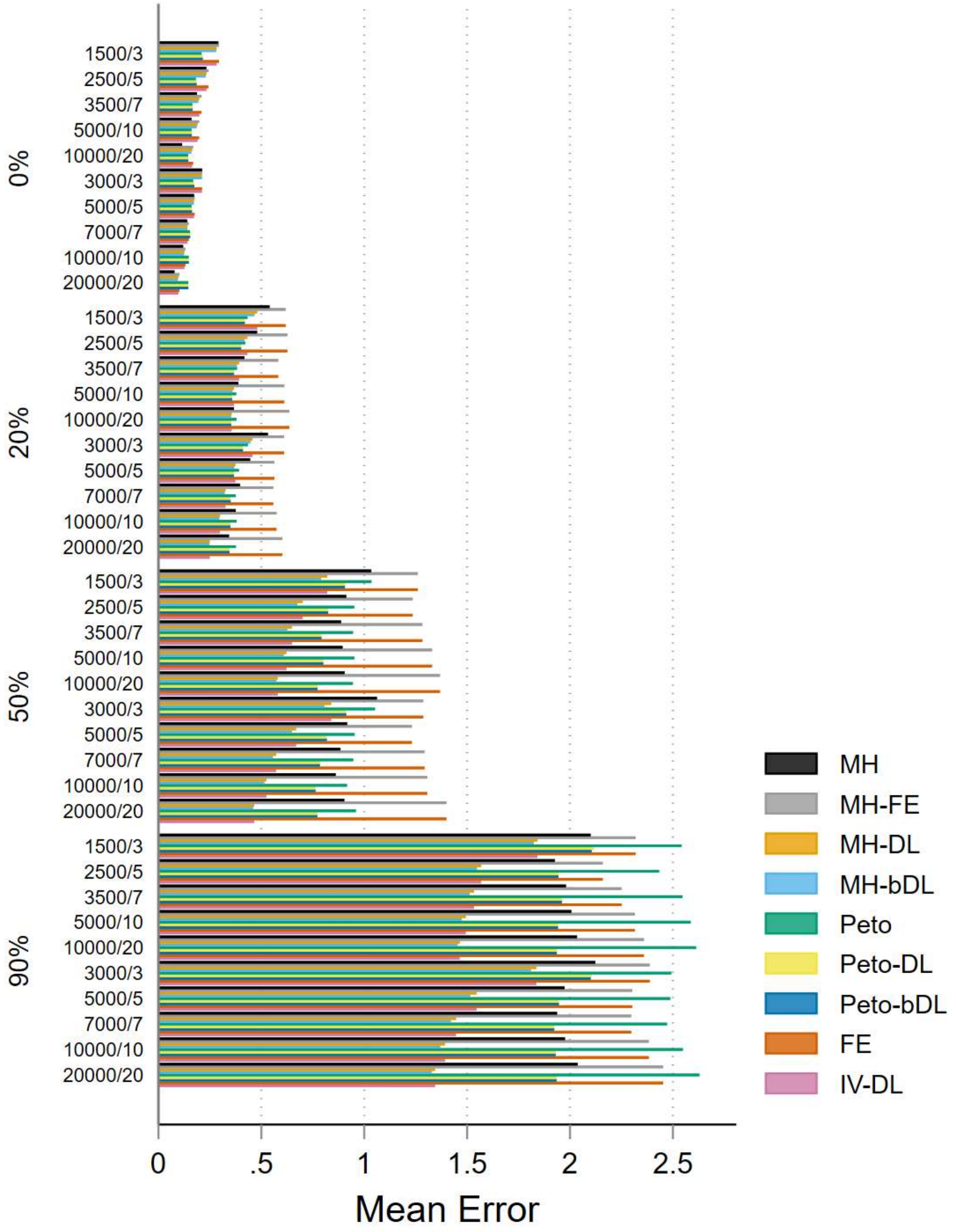

Figure S7: Mean error for very rare events with imbalanced allocation (r=0.1)

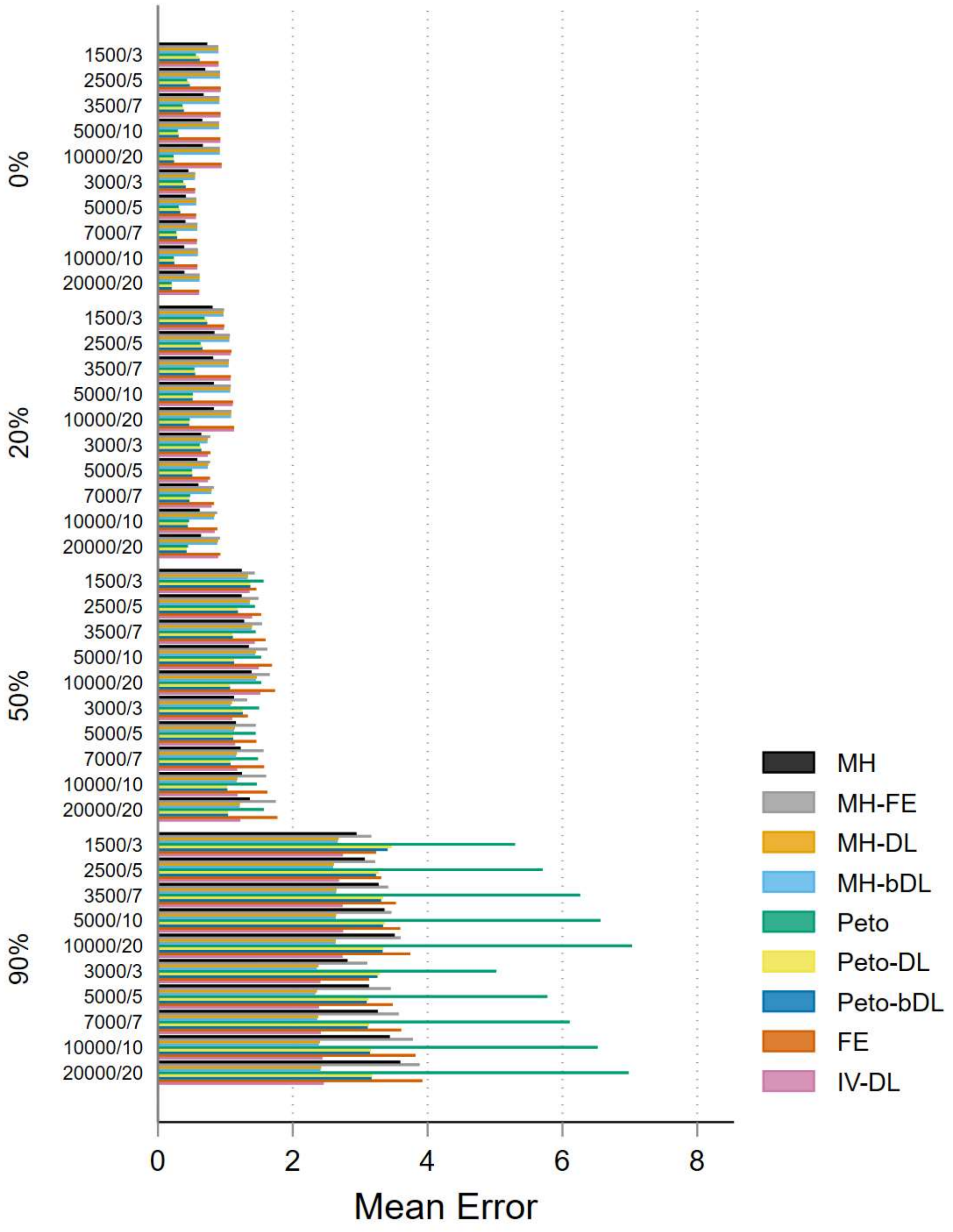

Figure S8: Mean error for very rare events with balanced allocation (r=0.5)

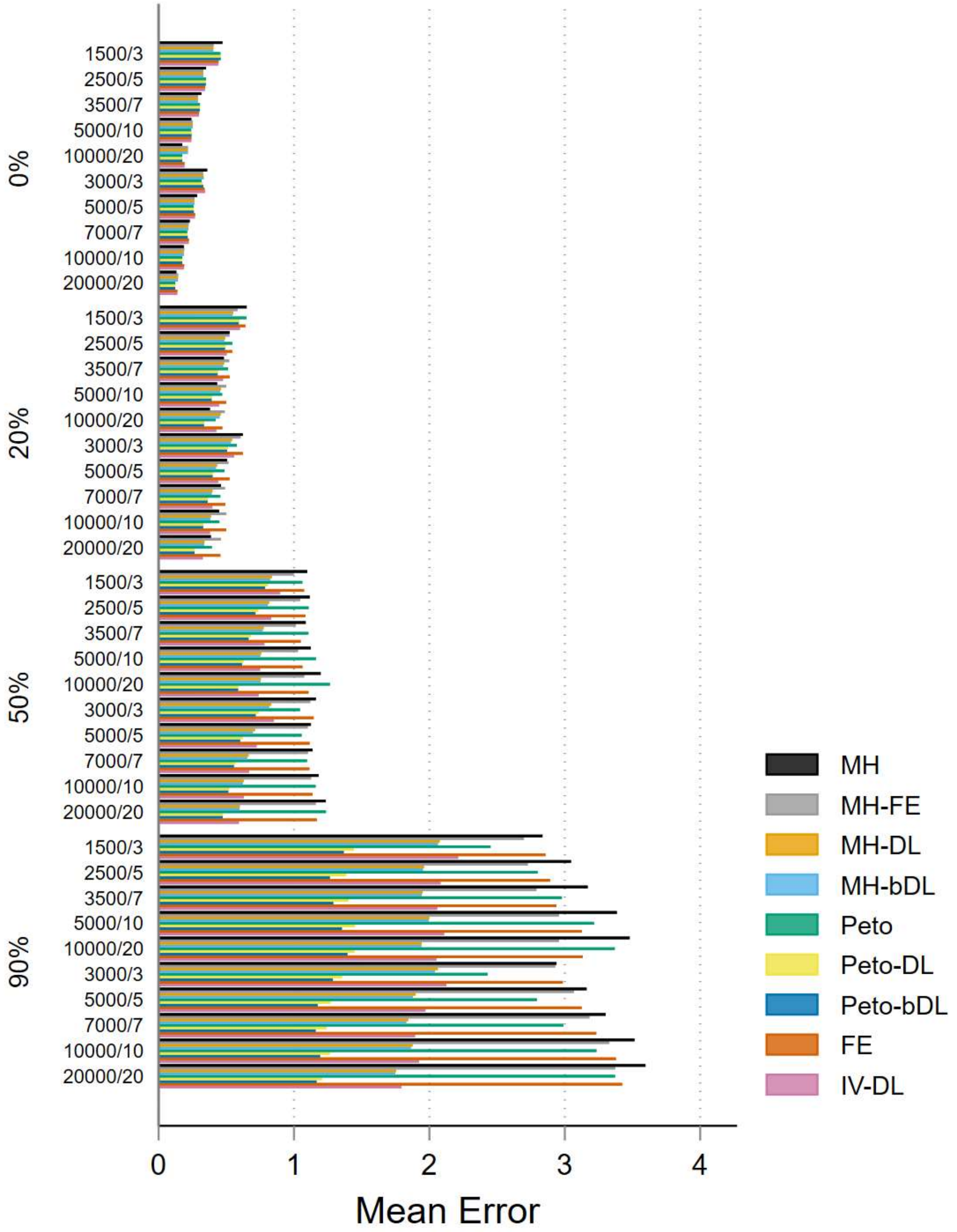

Figure S9: Mean error for non-rare events with imbalance allocation (r=0.1)

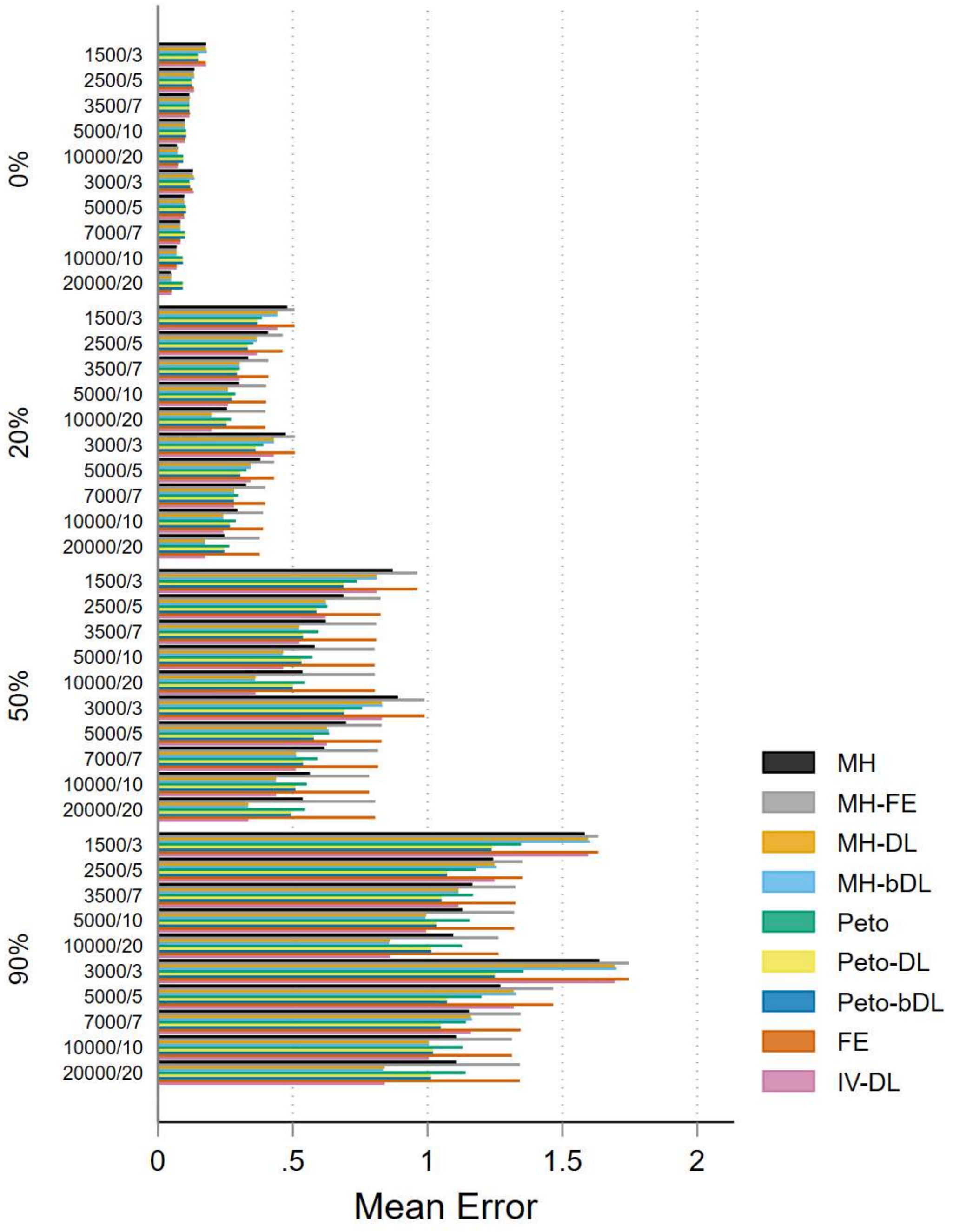

Figure S10: Mean error for non-rare events in balanced allocation (r=0.5)

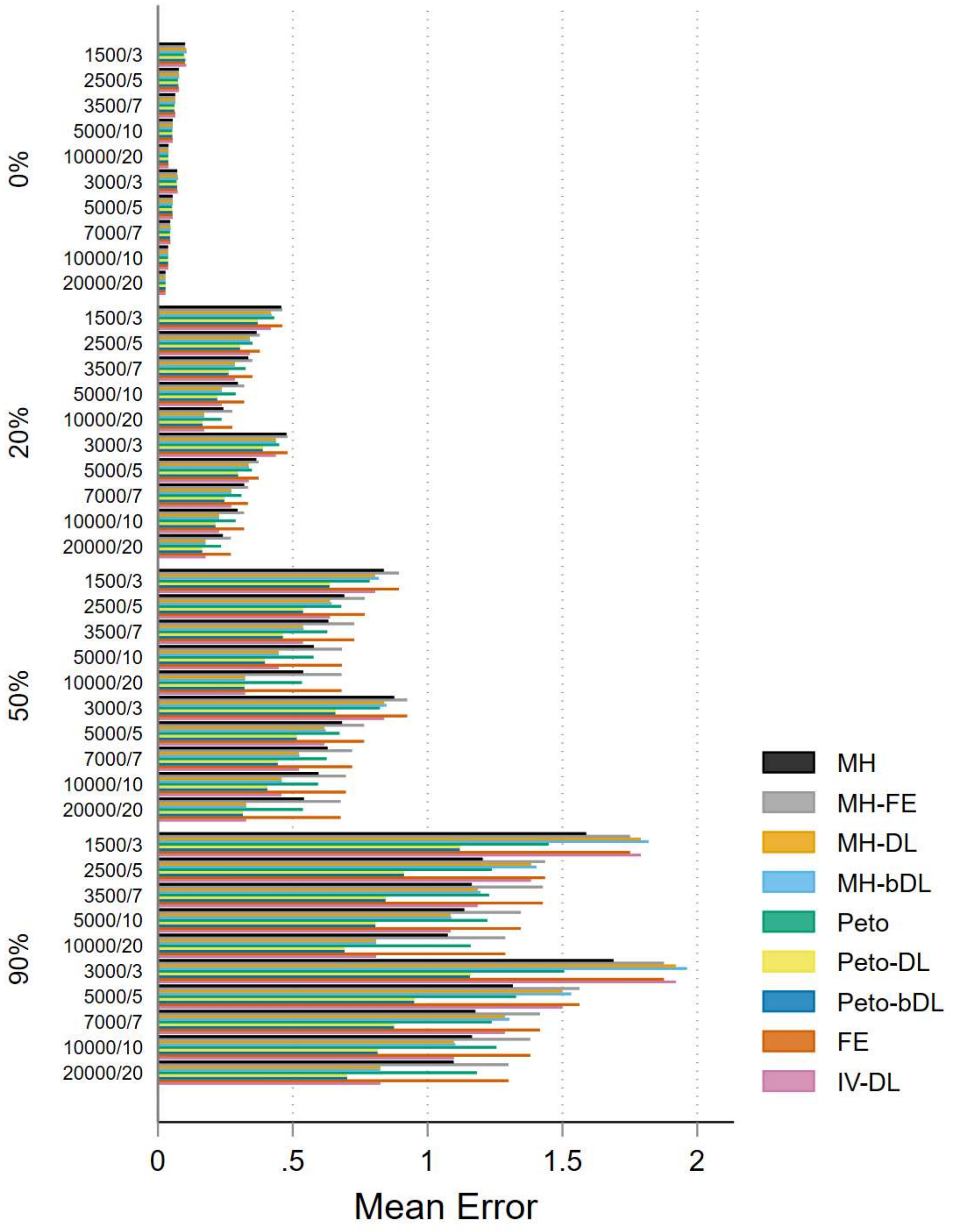

Appendix 5: Coverage

Figure S11: Coverage of rare events for imbalanced allocation (r=0.1)

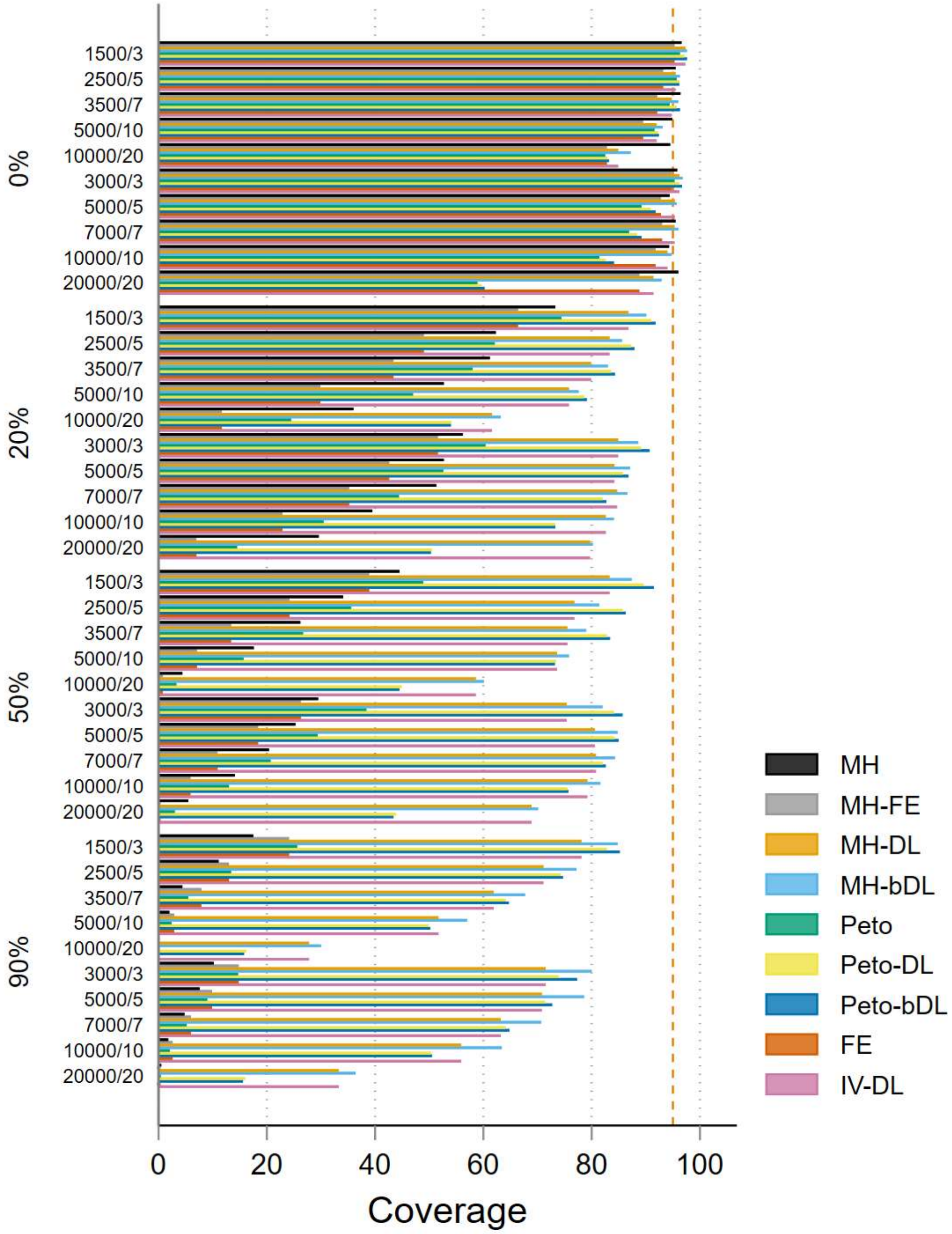

Figure S12: Coverage of very rare events with imbalance allocation (r=0.1)

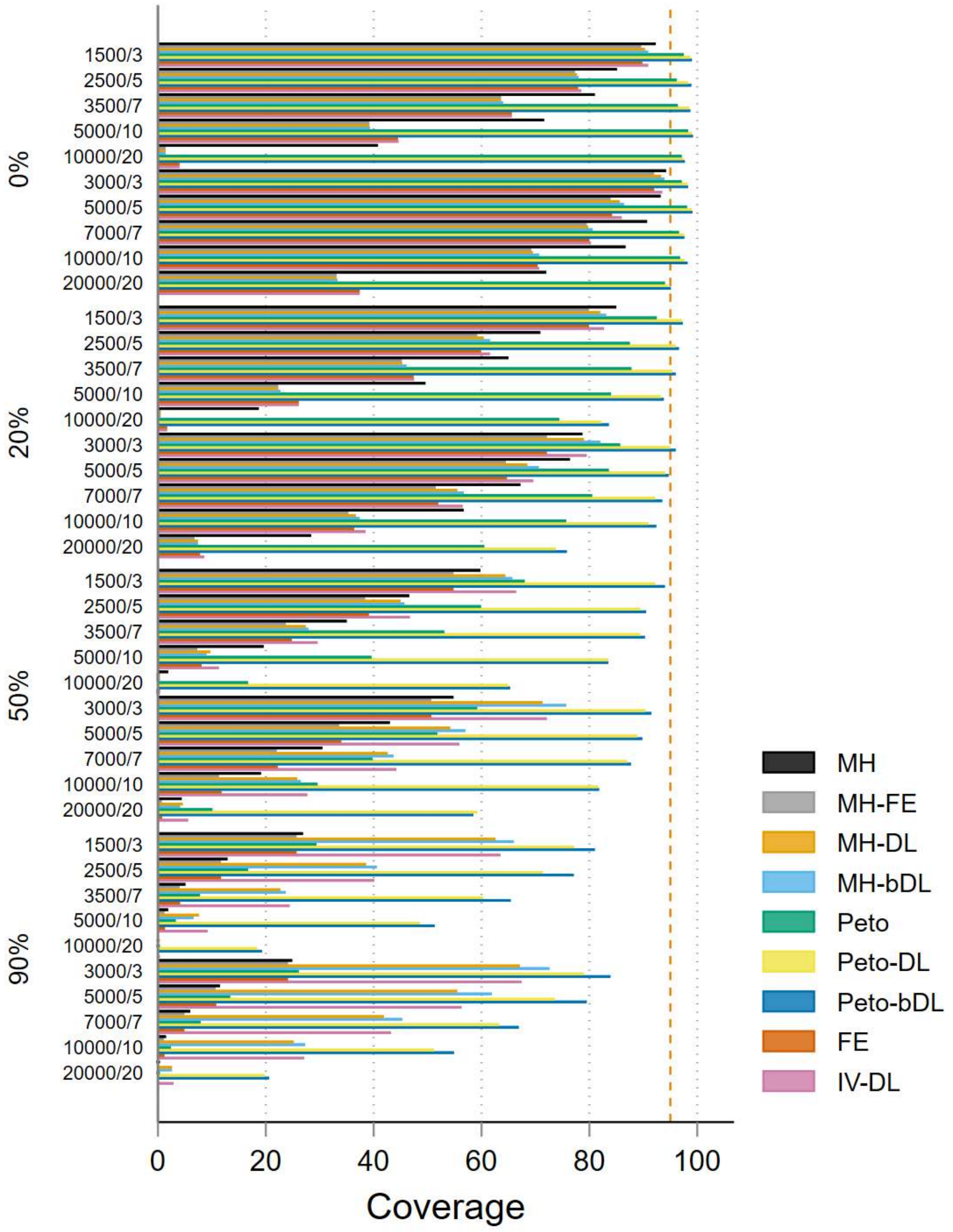

Figure S13: Coverage of very rare events for balanced allocation (r=0.5)

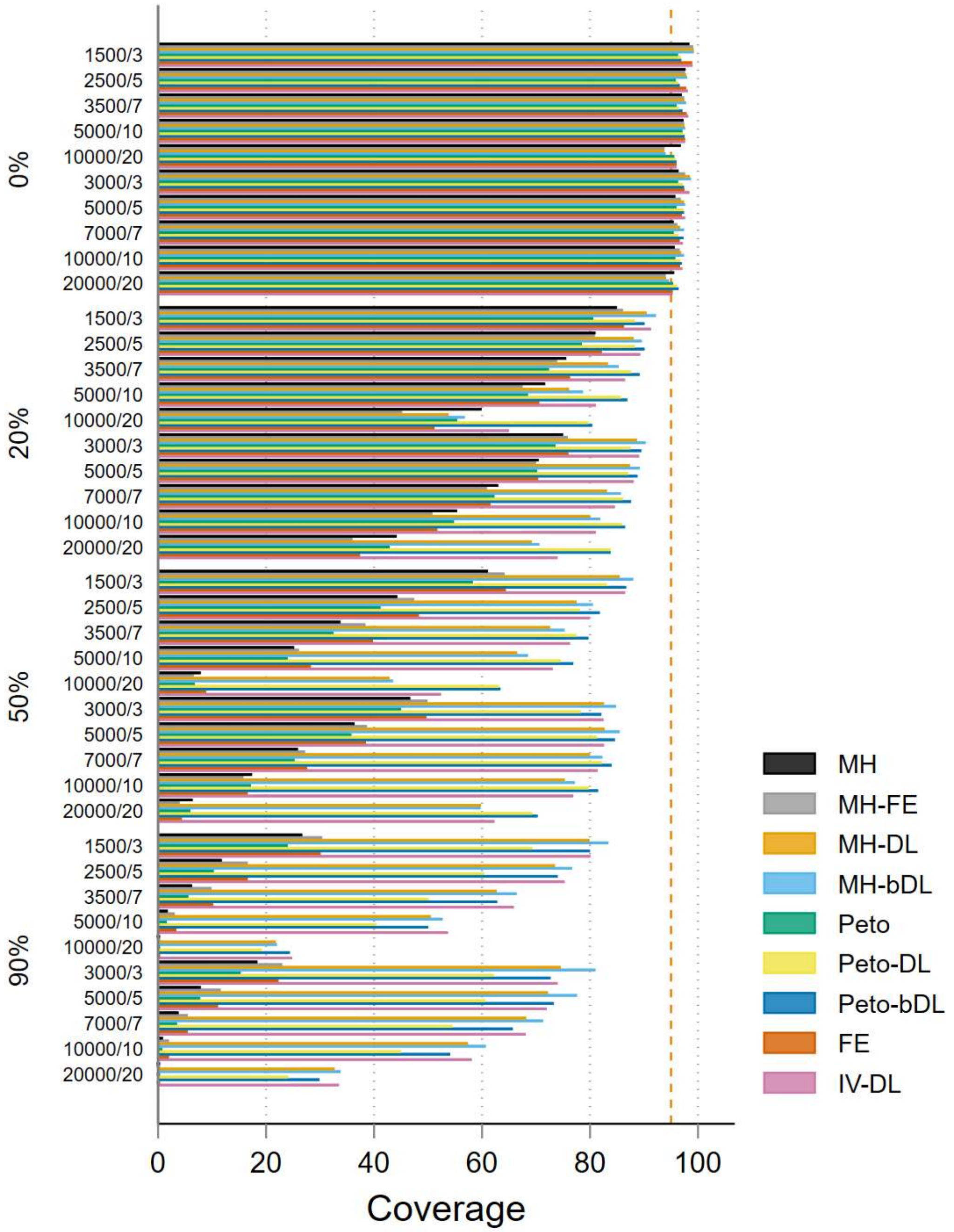

Figure S14: Coverage of non-rare events for imbalanced allocation (r=0.1)

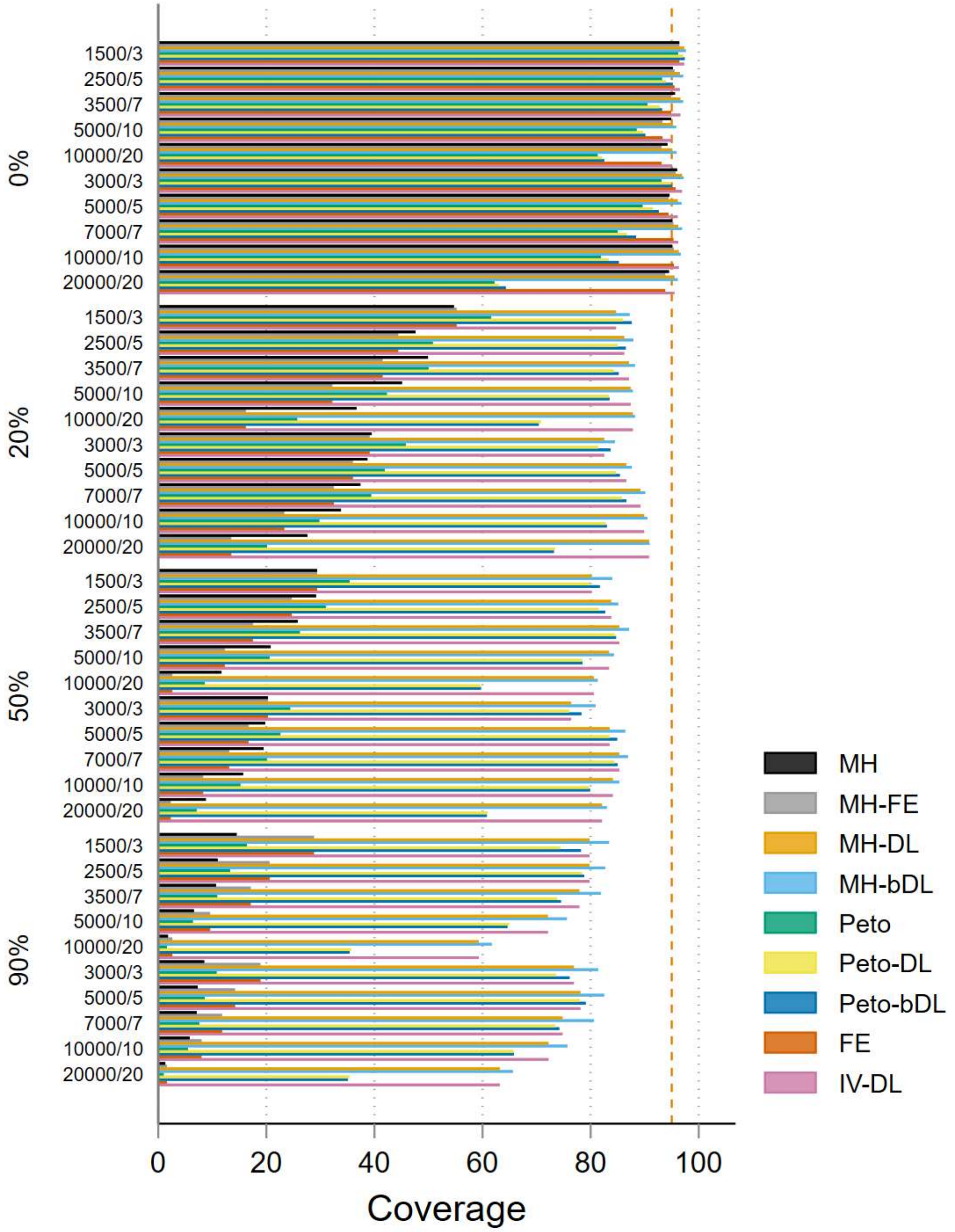

Figure S15: Coverage of non-rare events with balanced allocation (r=0.5)

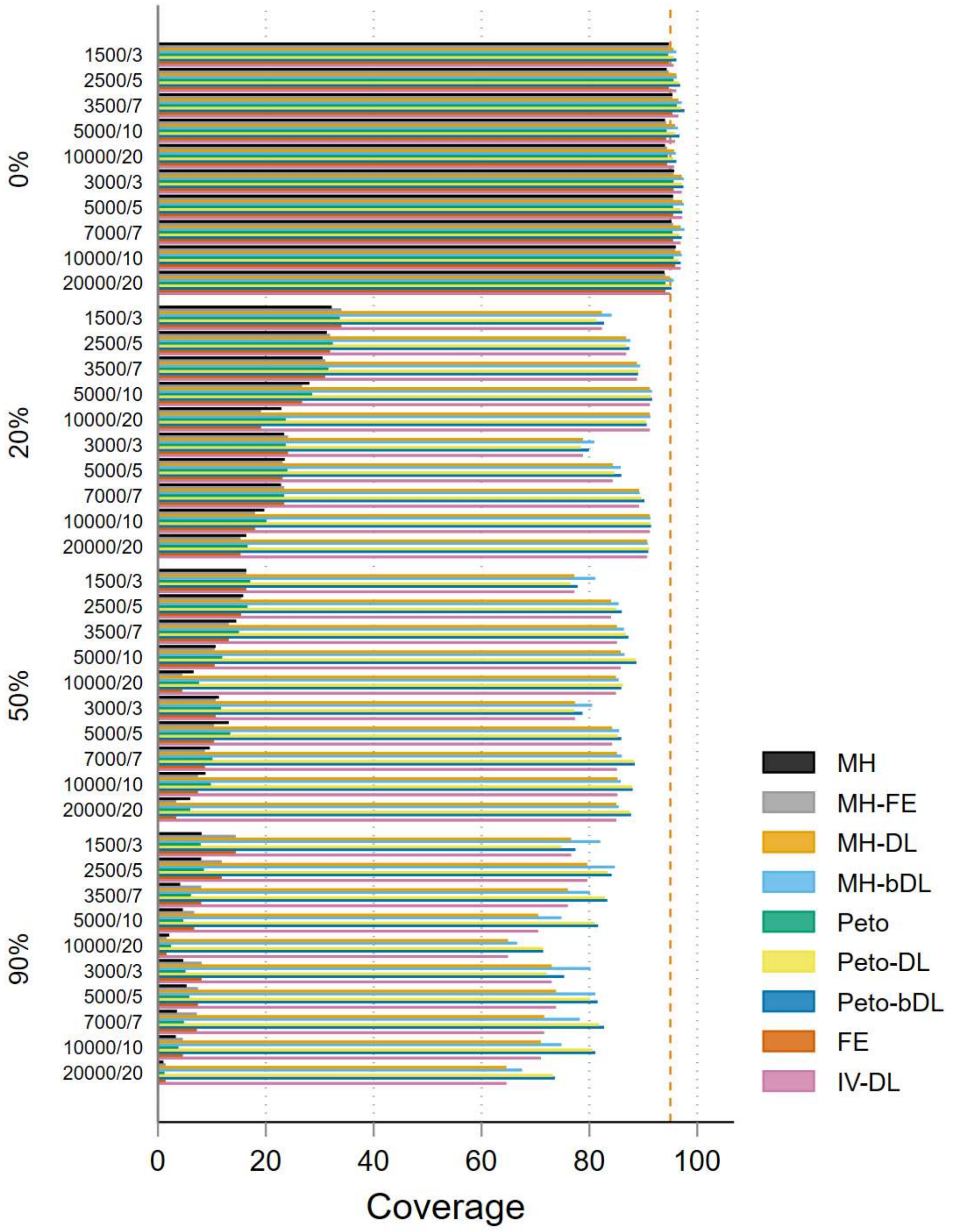

Appendix 6: Power

Figure S16: Power of rare events with imbalanced allocation (r=0.1)

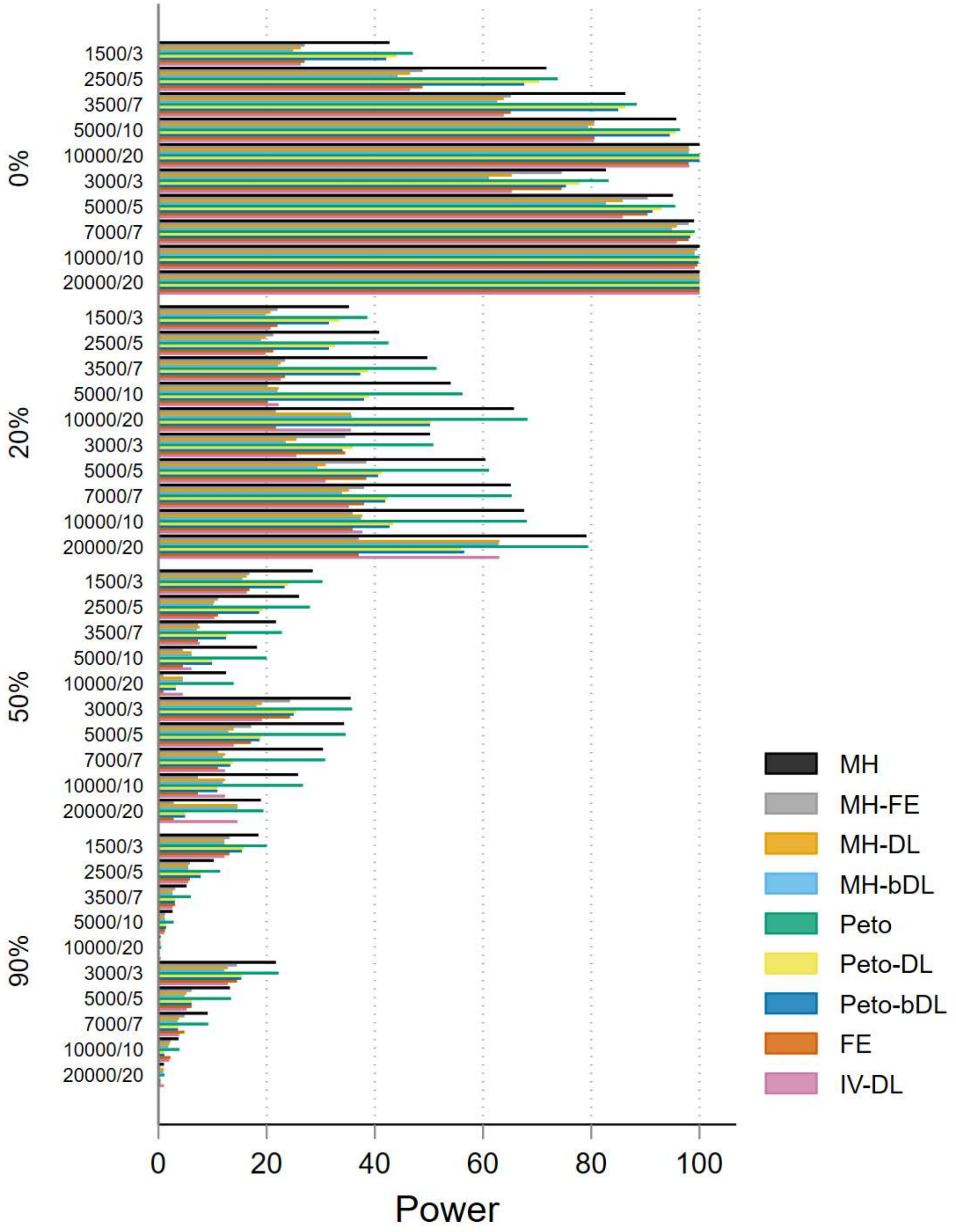

Figure S17: Power for very rare events with balanced allocation (r=0.5)

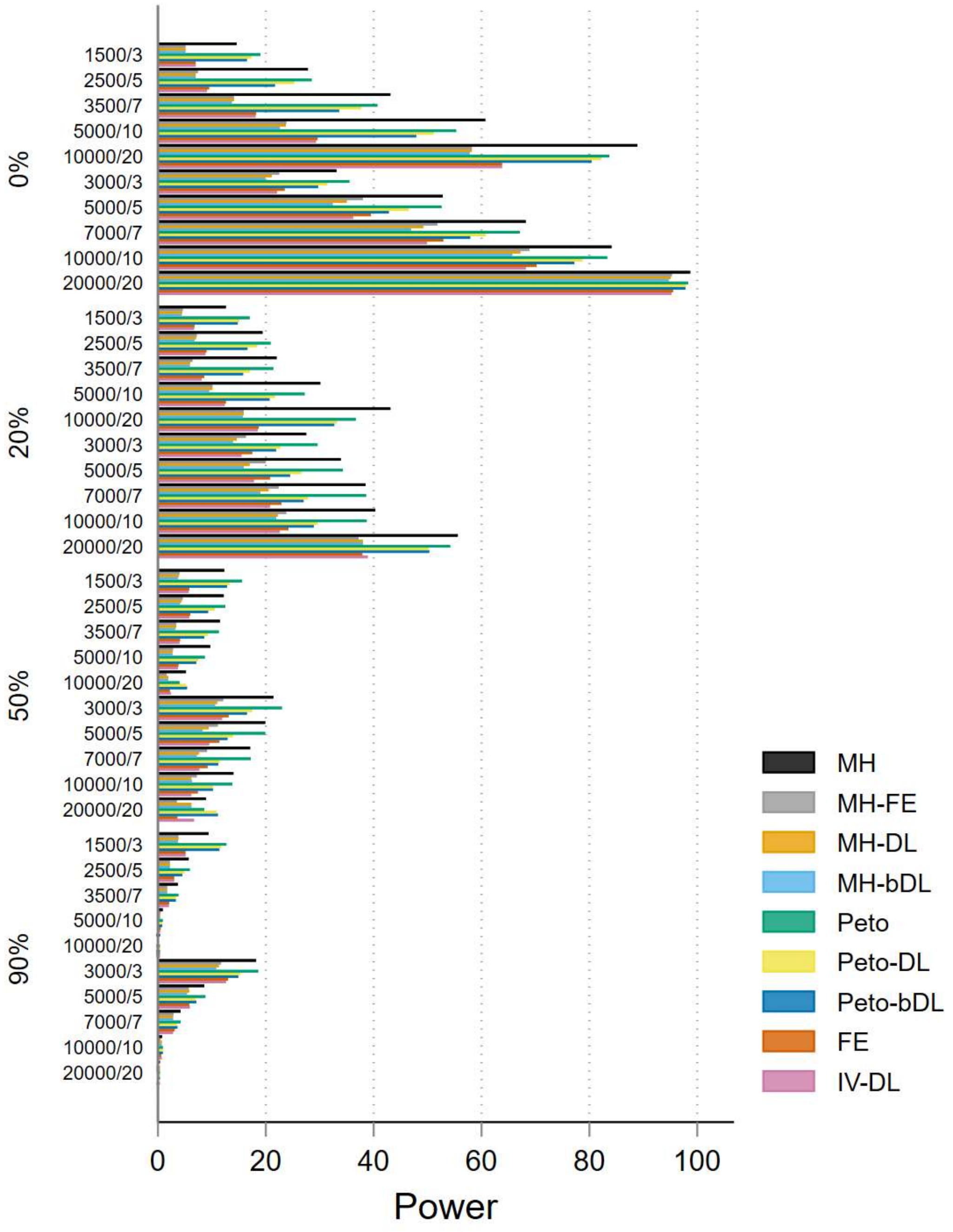

Figure S18: Power for non-rare events with imbalanced allocation (r=0.1)

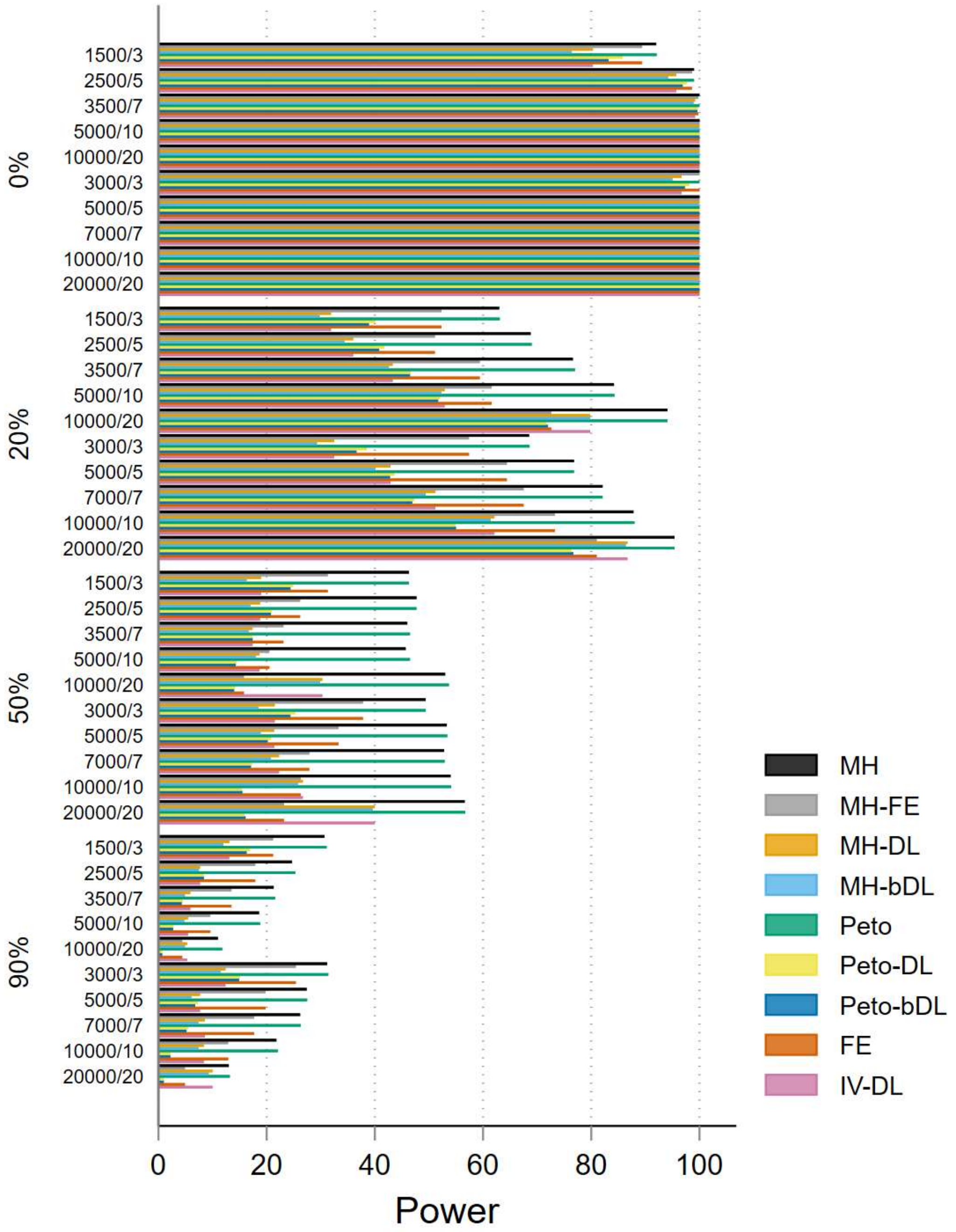

Figure S19: Power of non-rare events for balanced allocation (r=0.5)

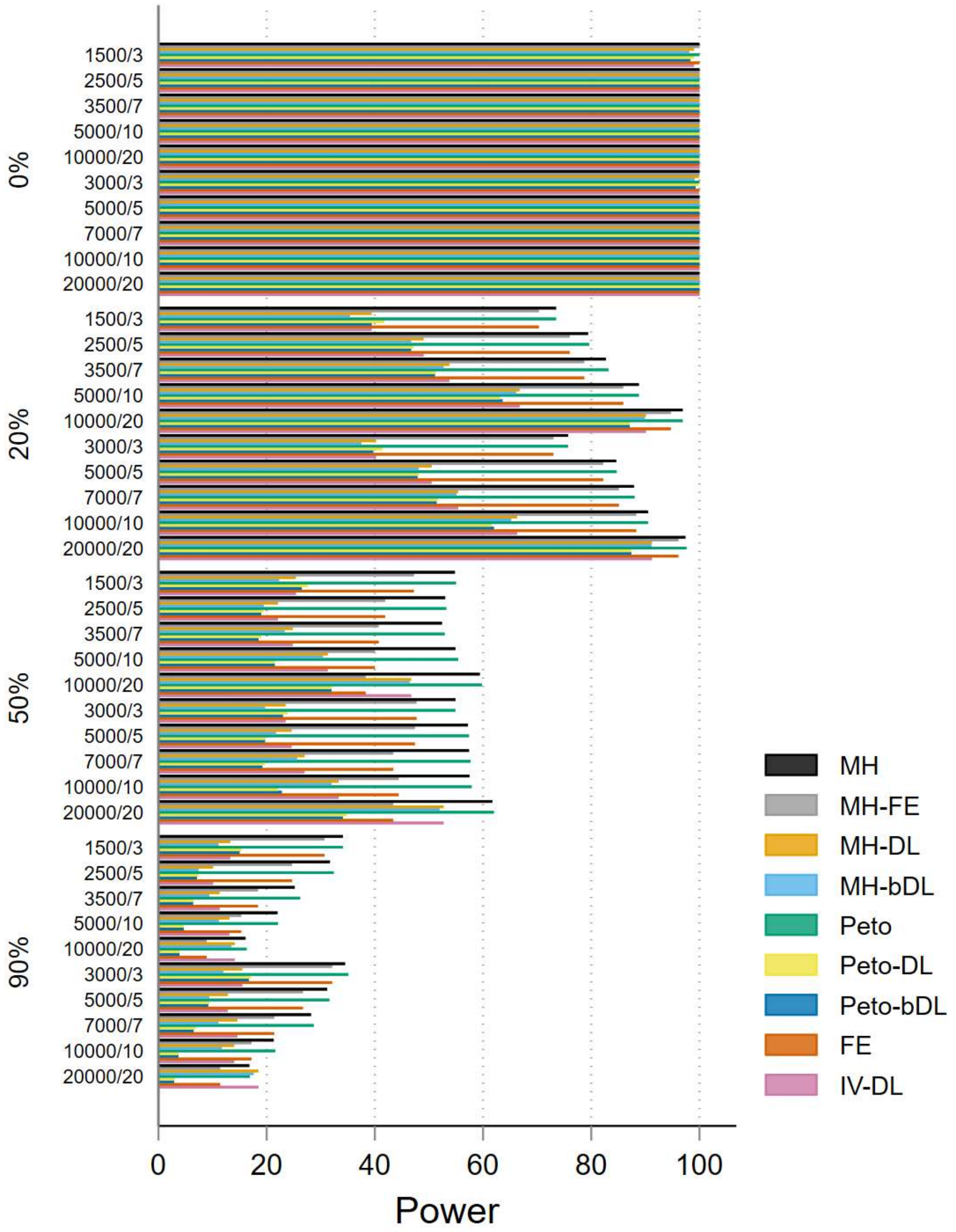

Appendix 7: Convergence

Figure S20: Convergence for rare events (r=0.1)

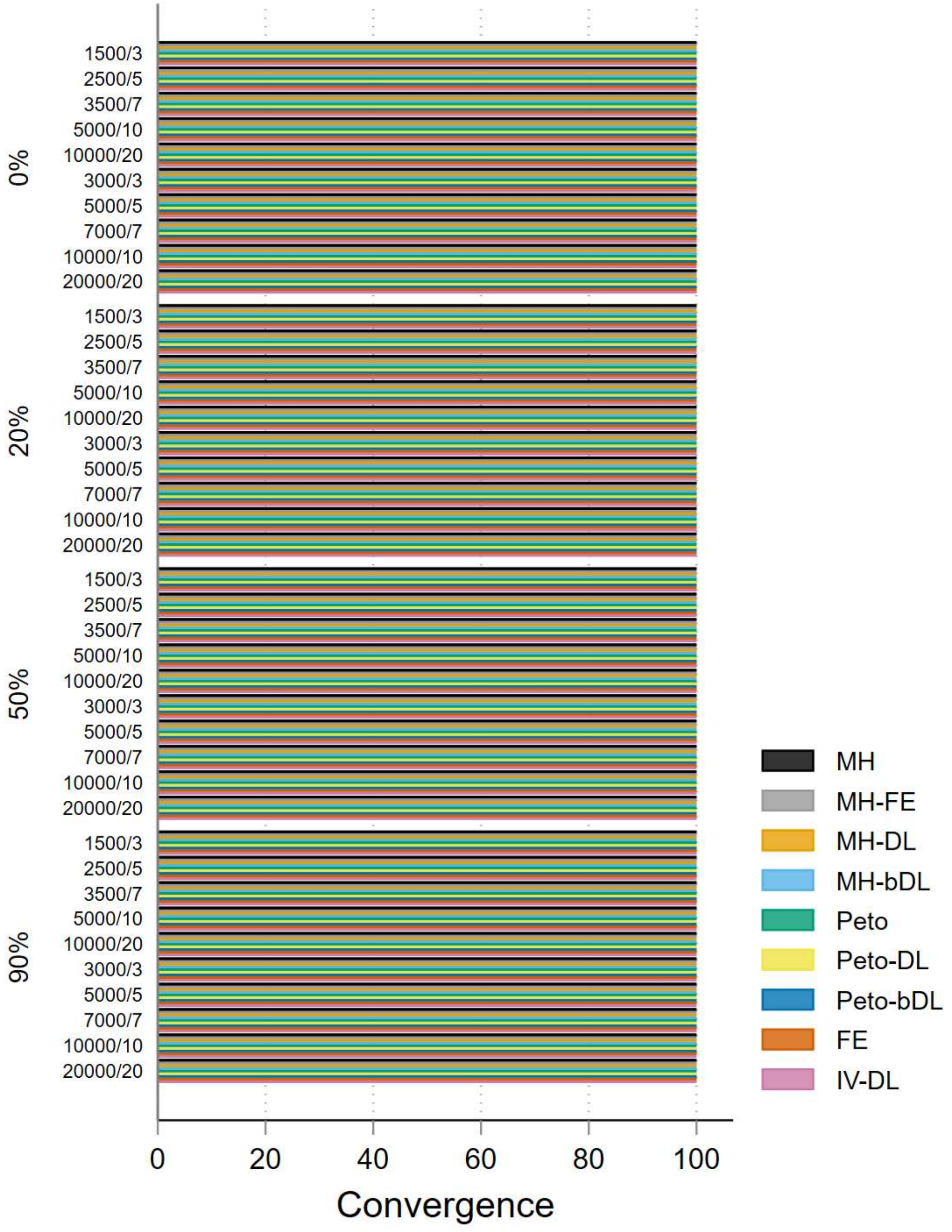

Figure S21: Convergence of rare events (r=0.5)

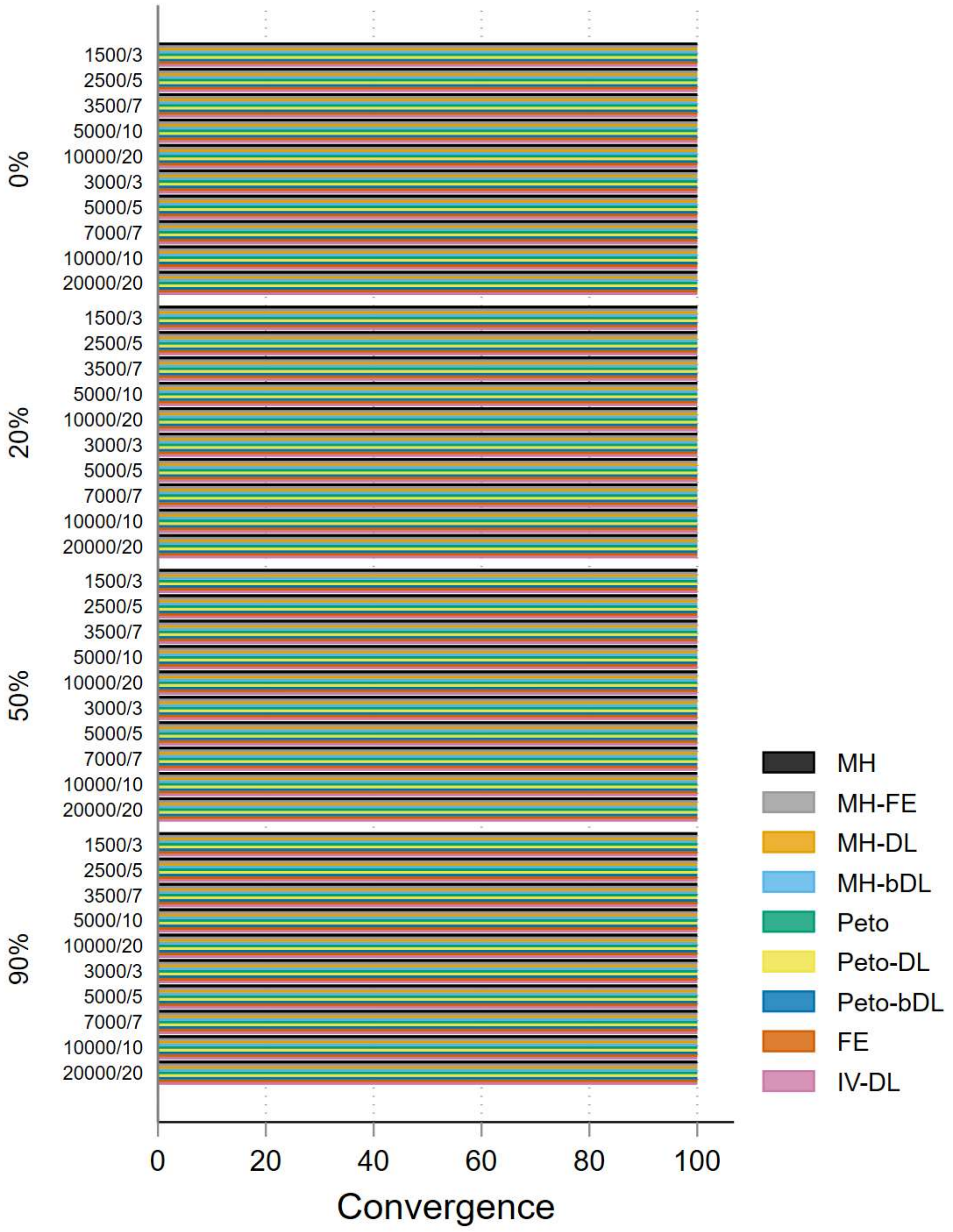

Figure S22: Convergence for very rare events (r=0.1)

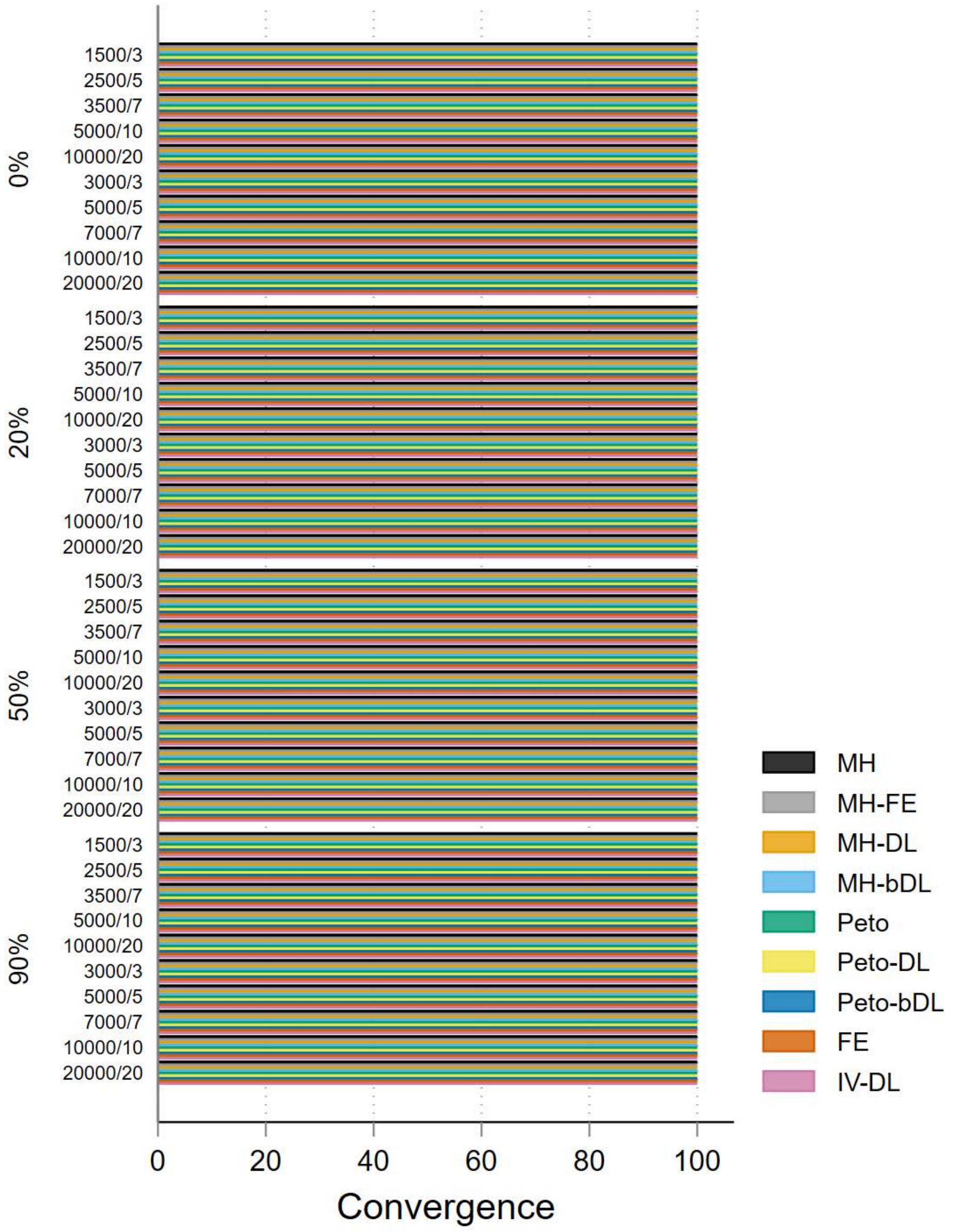

Figure S23: Convergence for very rare events (r=0.5)

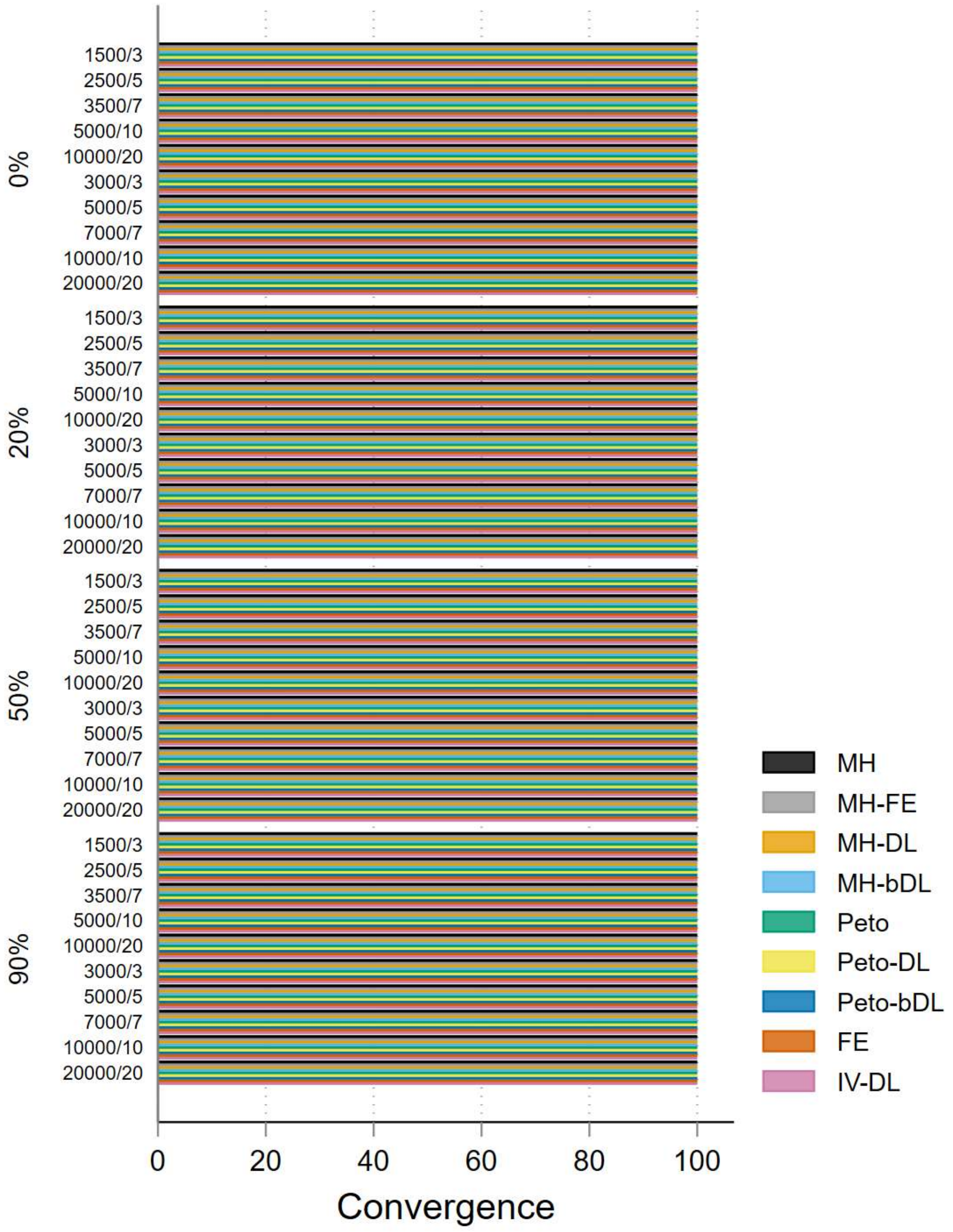

Figure S24: Convergence for non-rare events (r=0.1)

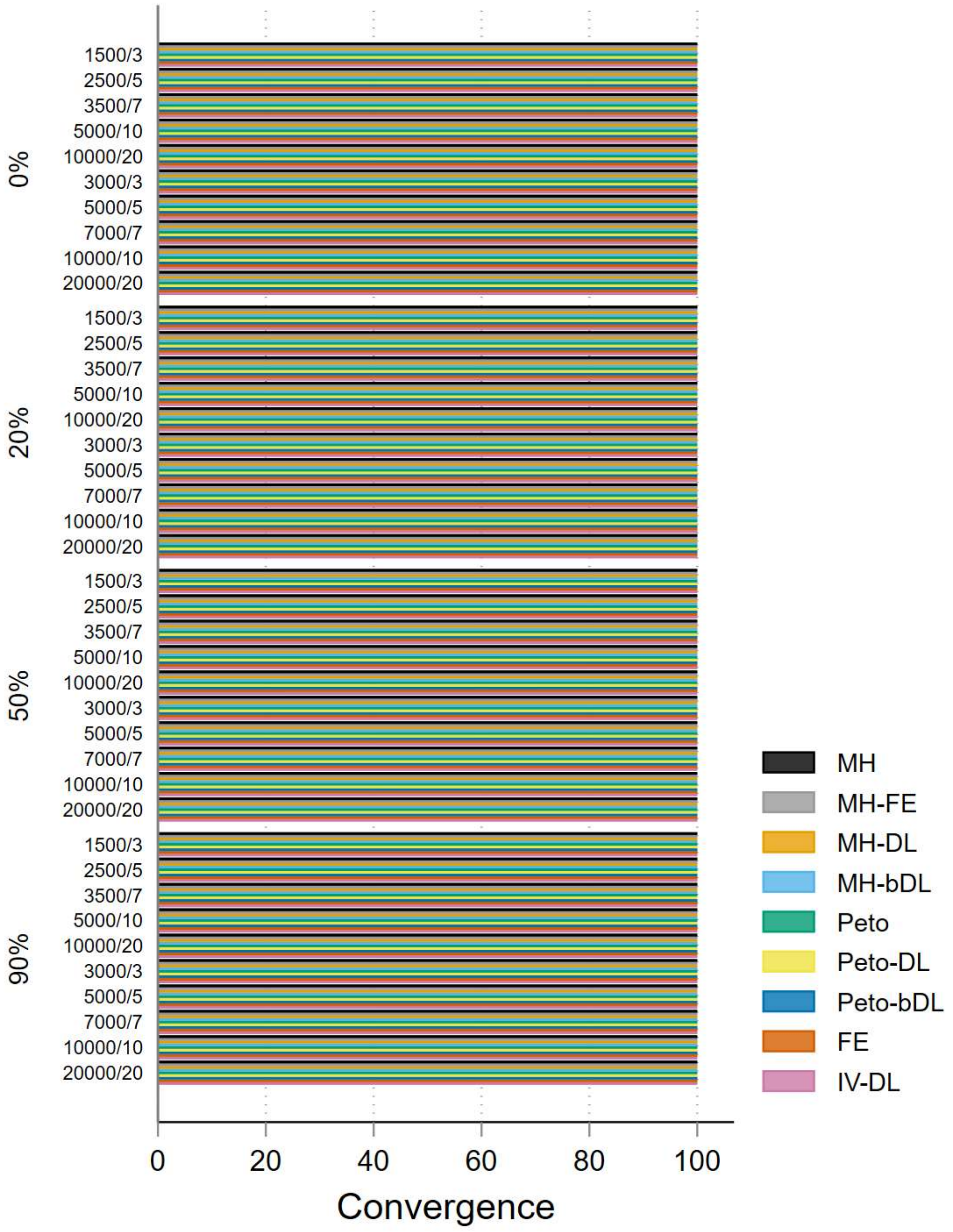

Figure S25: Convergence for non-rare events (r=0.5)

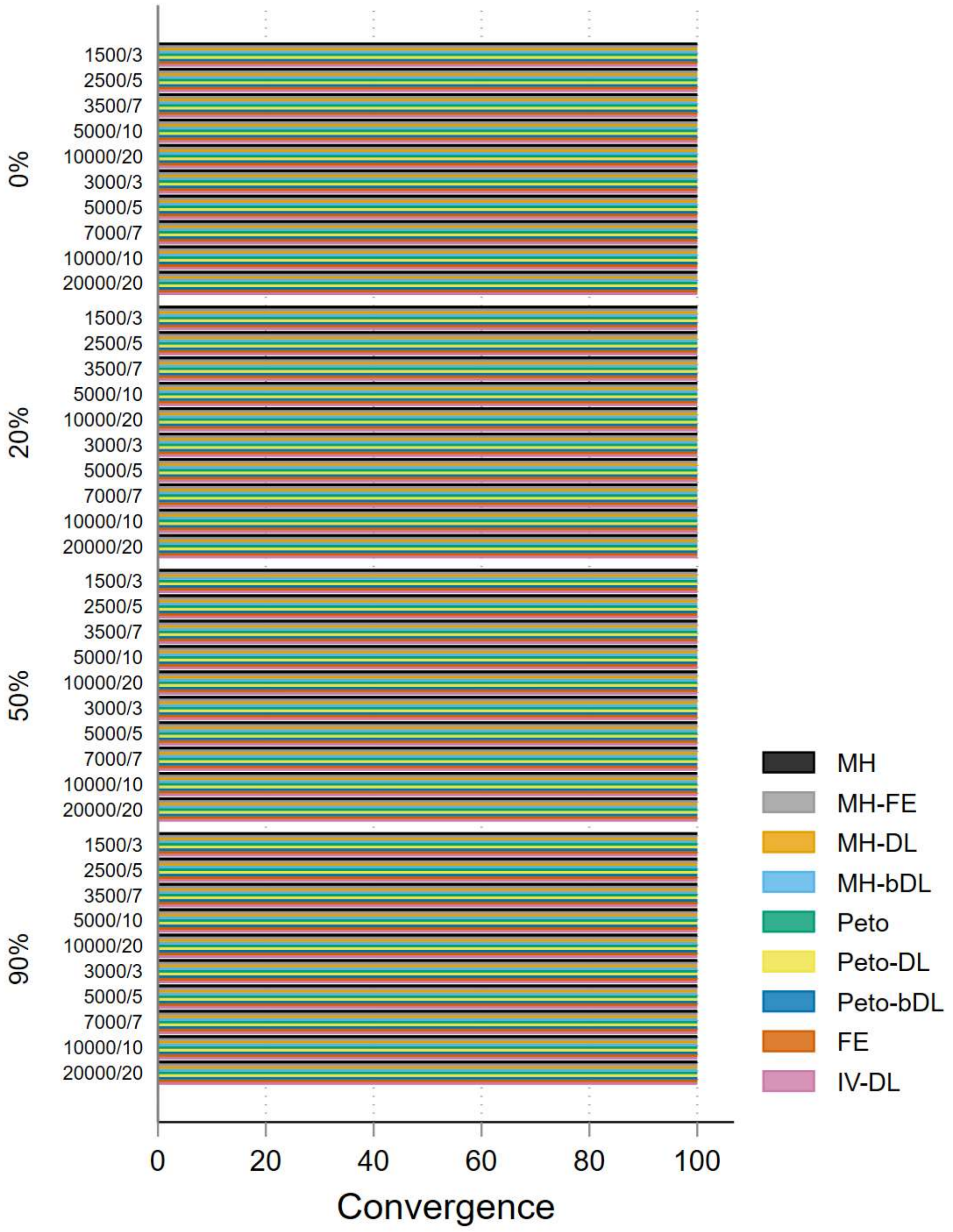

Appendix 8: Application of methods in practice

Figure S26: Coverage + power of rare events (r=0.1)

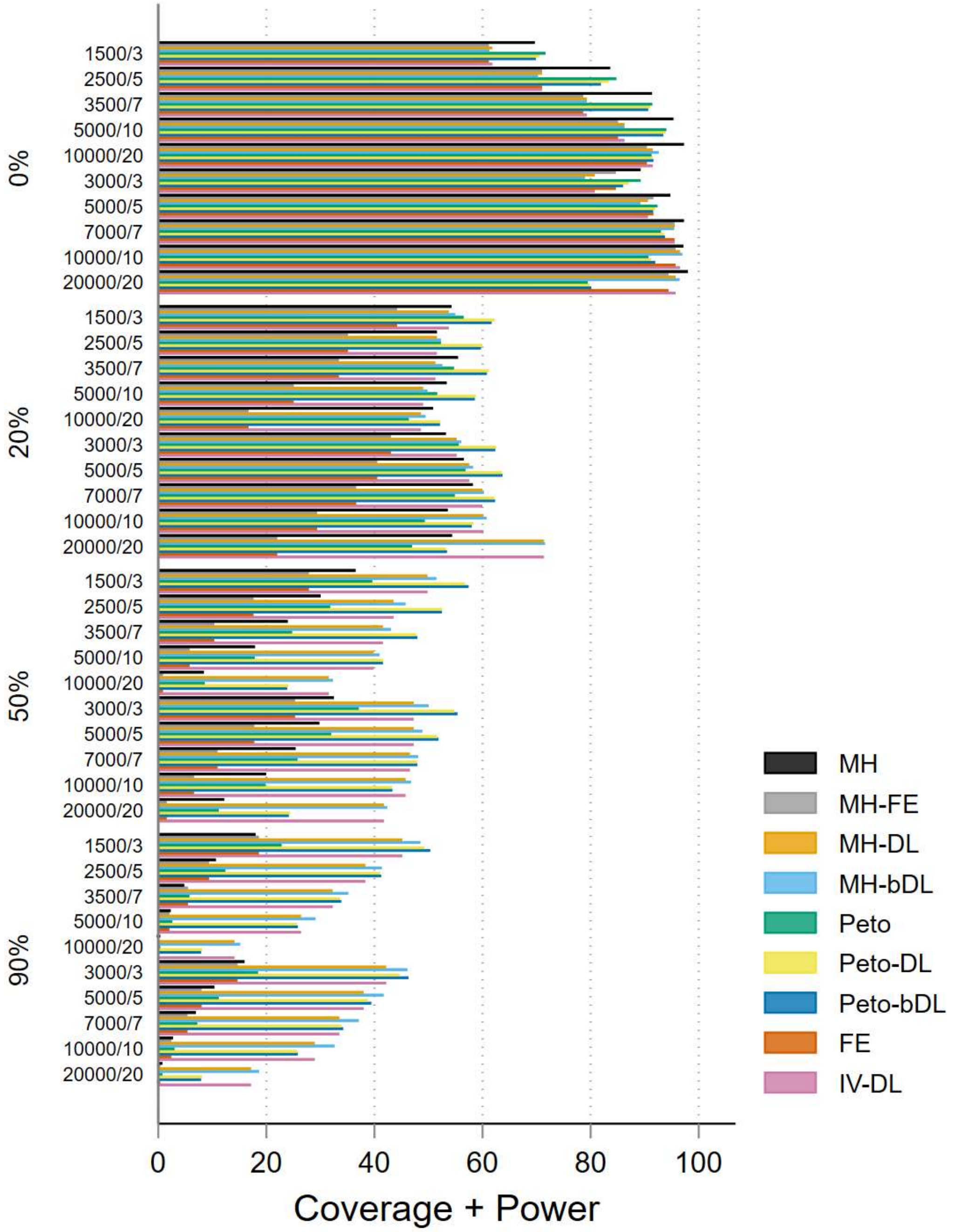

Figure S27: Coverage + power of rare events (r=0.5)

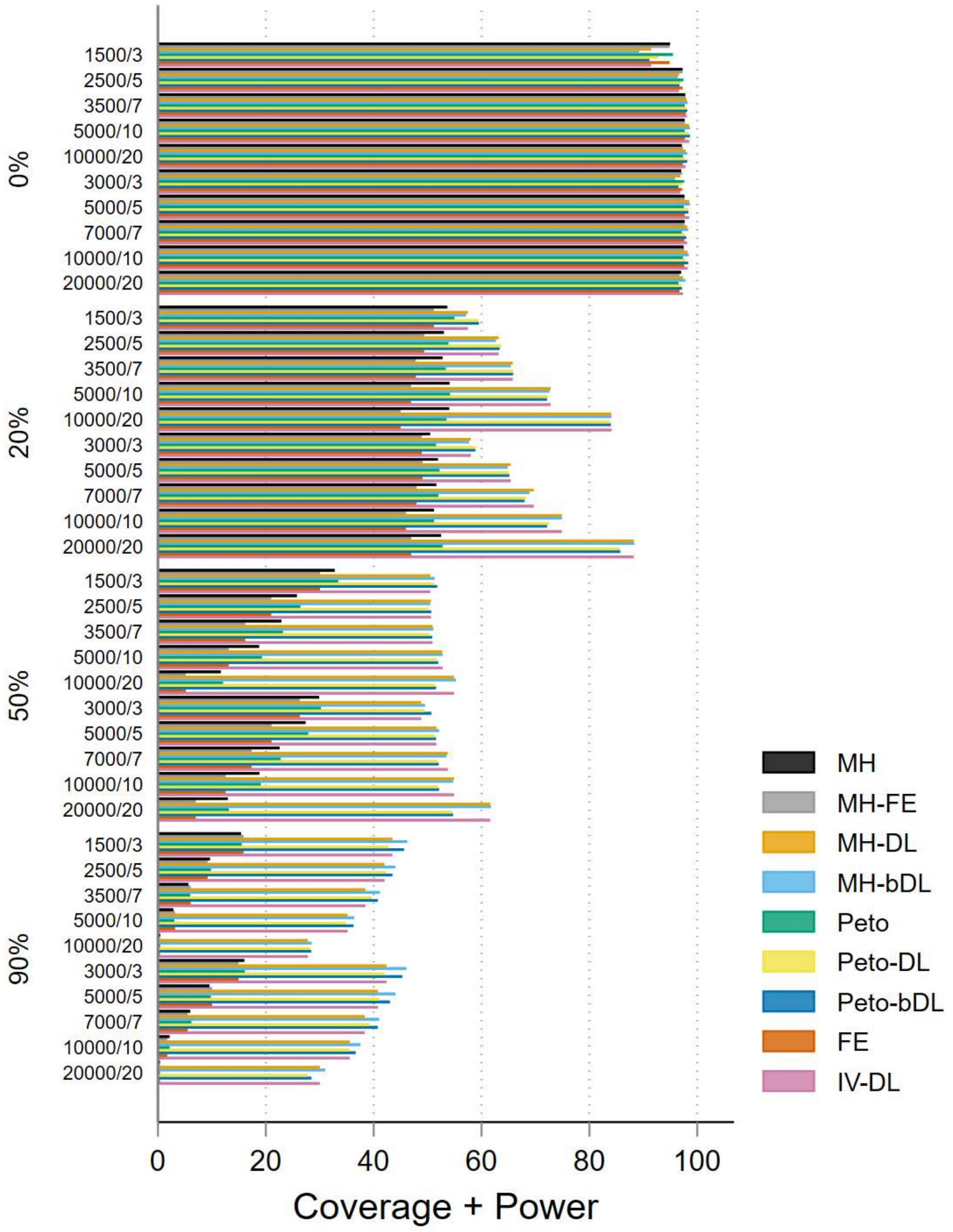

Figure S28: Coverage + power of very rare events (r=0.1)

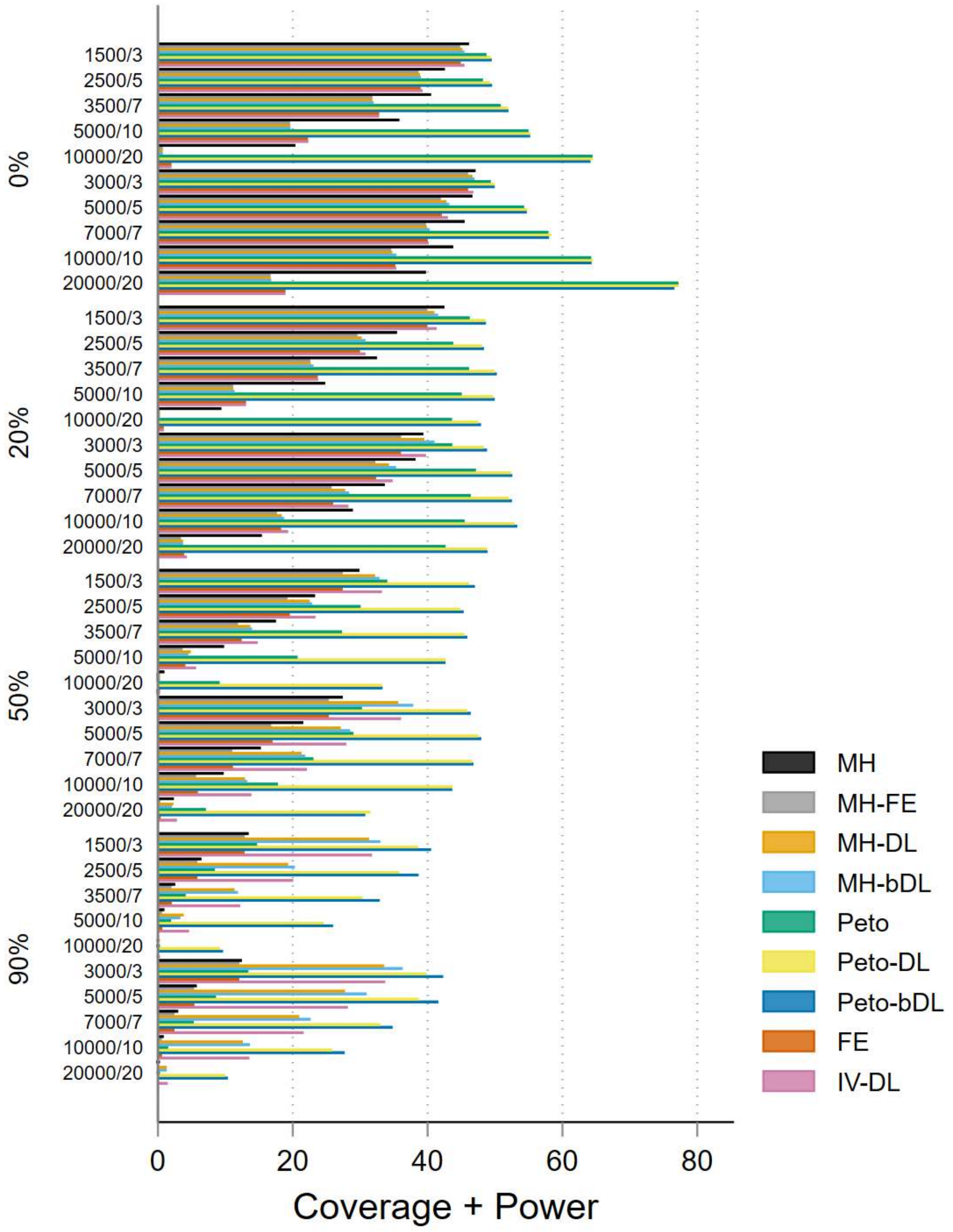

Figure S29: Coverage + power of very rare events (r=0.5)

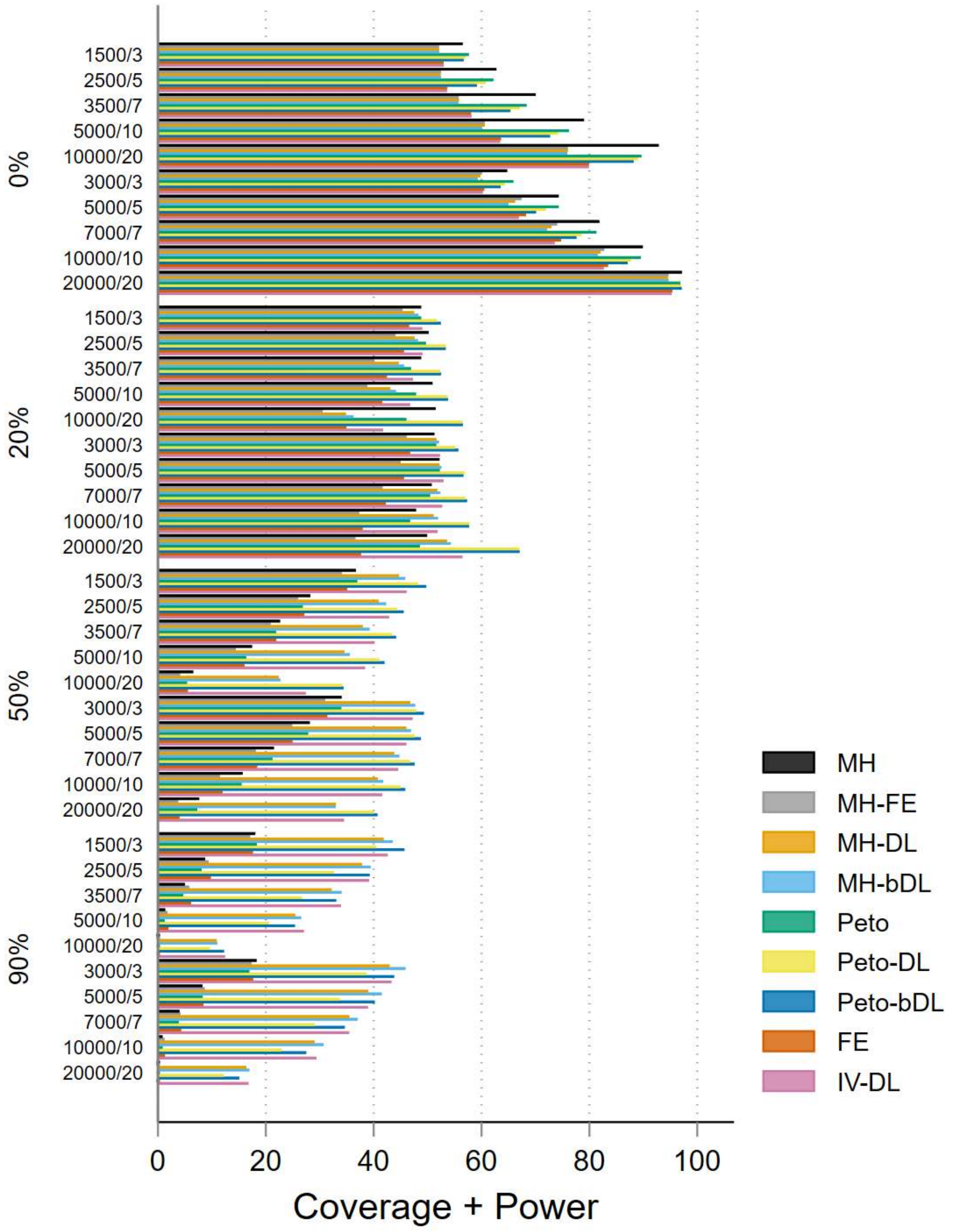

Figure S30: Coverage + power for non-rare events (r=0.1)

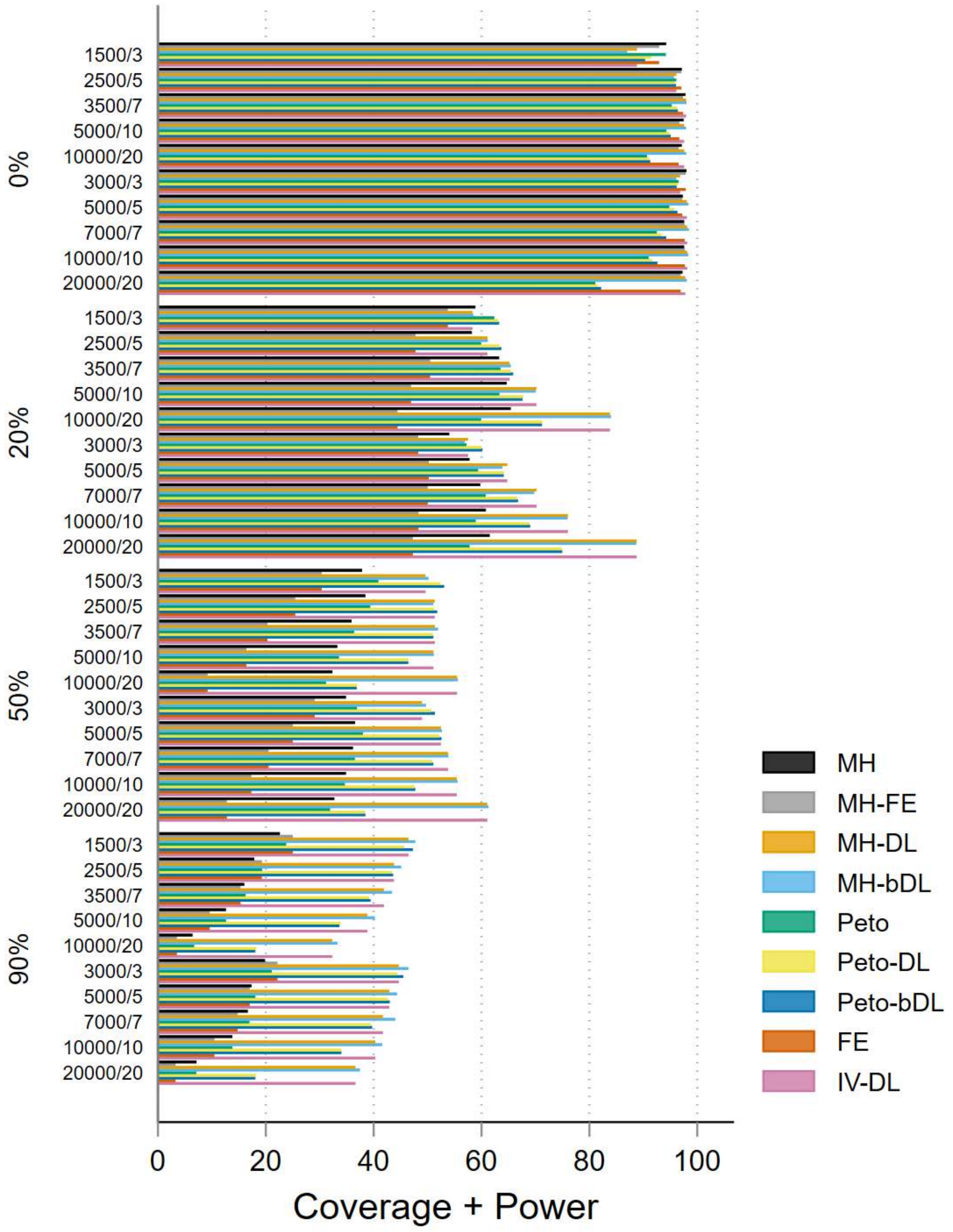

Figure S31: Coverage + power for non-rare events (r=0.5)

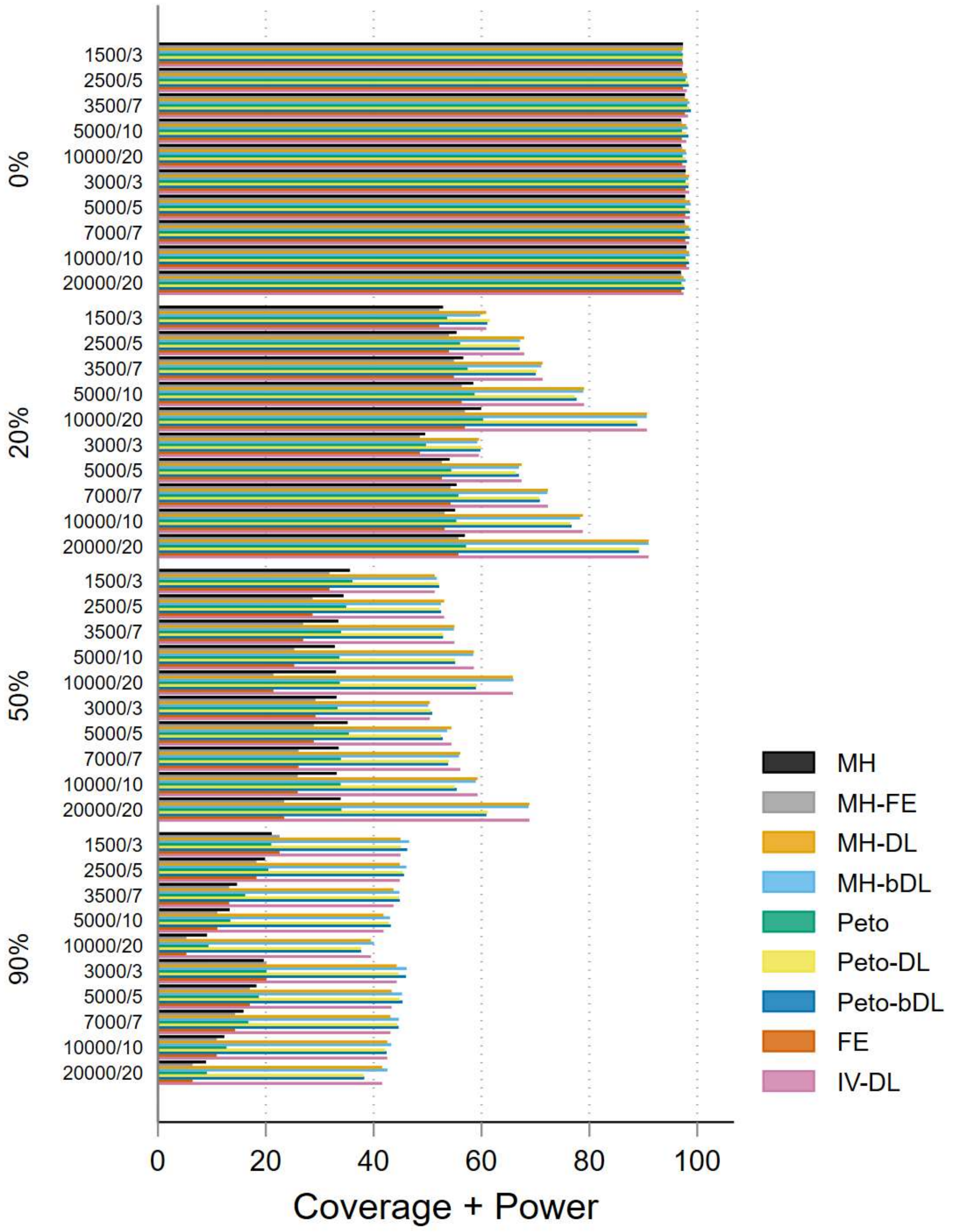

Table S2: Recommended method(s) for meta-analysis with imbalanced allocation ratio (r= 0.1) based on coverage and power

		τ^2			
		0%	20%	50%	90%
Sample size setting	1500/3	VR=Peto-bDL<br>R=Peto<br>NR=Peto/MH	VR=Peto-bDL<br>R=Peto-DL<br>NR=Peto-bDL	VR=Peto-bDL<br>R=Peto-bDL<br>NR=Peto-bDL	VR=Peto-bDL<br>R=Peto-bDL<br>NR=MH-bDL
	2500/5	VR=Peto-bDL<br>R=Peto<br>NR=MH/MH-FE	VR=Peto-bDL<br>R=Peto-DL<br>NR=Peto-bDL	VR=Peto-bDL<br>R=Peto-DL<br>NR=Peto-bDL	VR=Peto-bDL<br>R=MH-bDL<br>NR=MH-bDL
	3000/3	VR=Peto-bDL<br>R=Peto<br>NR=MH	VR=Peto-bDL<br>R=Peto-DL<br>NR=Peto-bDL	VR=Peto-bDL<br>R=Peto-bDL<br>NR=Peto-bDL	VR=Peto-bDL<br>R=Peto-bDL<br>NR=MH-bDL
	3500/7	VR=Peto-bDL<br>R=Peto<br>NR=Not obvious	VR=Peto-bDL<br>R=Peto-DL<br>NR=Peto-bDL	VR=Peto-bDL<br>R=Peto-bDL<br>NR=MH-bDL	VR=Peto-bDL<br>R=MH-bDL<br>NR=MH-bDL
	5000/5	VR=Peto-DL<br>R=MH<br>NR=MH-bDL	VR=Peto-bDL<br>R=Peto-bDL<br>NR=MH-DL	VR=Peto-bDL<br>R=Peto-bDL<br>NR=MH-bDL	VR=Peto-bDL<br>R=MH-bDL<br>NR=MH-bDL
	5000/10	VR=Peto-DL<br>R=MH<br>NR=MH-bDL	VR=Peto-bDL<br>R=Peto-DL<br>NR=MH-DL	VR=Peto-bDL<br>R=Peto-bDL<br>NR=MH-bDL	VR=Peto-bDL<br>R=MH-bDL<br>NR=MH-bDL
	7000/7	VR=Peto-DL<br>R=MH<br>NR=MH-bDL	VR=Peto-bDL<br>R=Peto-bDL<br>NR=MH-DL	VR=Peto-bDL<br>R=MH-bDL<br>NR=MH-bDL	VR=Peto-bDL<br>R=MH-bDL<br>NR=MH-bDL
	10000/10	VR=Peto-DL<br>R=MH<br>NR=MH-bDL	VR=Peto-bDL<br>R=MH-bDL<br>NR=MH-DL	VR=Peto-bDL<br>R=MH-bDL<br>NR=MH-bDL	VR=Peto-bDL<br>R=MH-bDL<br>NR=MH-bDL
	10000/20	VR=Peto-DL<br>R=MH<br>NR=MH-bDL	VR=Peto-bDL<br>R=Peto-DL<br>NR=MH-bDL	VR=Peto-bDL<br>R=MH-bDL<br>NR=MH-bDL	VR=Peto-bDL<br>R=MH-bDL<br>NR=MH-bDL
	20000/20	VR=Peto-DL<br>R=MH<br>NR=MH-bDL	VR=Peto-bDL<br>R=MH-bDL<br>NR=MH-DL	VR=Peto-DL<br>R=MH-bDL<br>NR=MH-bDL	VR=Peto-bDL<br>R=MH-bDL<br>NR=MH-bDL
