# Supplementary material for: Applications of simple and accessible methods for meta-analysis involving rare events: A simulation study
Source: Stat Methods Med Res. 2021 Jun 17;30(7):1589–608. doi: 10.1177/09622802211022385 (PMC8411477; doi:10.1177/09622802211022385)
Supplement: sj-pdf-1-smm-10.1177_09622802211022385 - Supplemental material for Applications of simple and accessible methods for meta-analysis involving rare events: A simulation study [file sj-pdf-1-smm-10.1177_09622802211022385.pdf]

Re: 'A guide for meta-analysis involving rare events: A simulation study.'

Dear Editor,

Meta-analyses (MAs) involving rare events, for example, safety or adverse event outcomes, are methodically more challenging as compared with those with frequently observed efficacy outcomes. The standard methods used when performing MAs of binary outcome data include the standard fixed-effects Mantel-Haenszel (MH) model, the generic inverse variance model, the Peto model or the standard random-effects DerSimonian-Laird (DL) model. However, because these methods are based on approximate likelihood and were developed using large sample theory, there statistical properties for estimating treatment-effects and the between-study variance (τ^2) are often susceptible to bias.

More recently several new methods have been proposed for improved estimation of τ^2. Specifically, the non-parametric bootstrap of the DL estimator was shown to be a better performer in small MAs. This non-parametric bootstrap of the DL has now been extended for both the MH and Peto models, but their performances in MAs involving rare events whilst compounded by heterogeneity, remains unknown. In the enclosed manuscript we present the first comprehensive simulation study involving seven methods that are currently available, recommended in the international guidelines, and can be easily used in MAs of rare events.

In this largest simulation study involving 360 generated MAs scenarios covering difference incidences, sample sizes, between-study variance (heterogeneity) and treatment allocation; we have shown that the Peto-bDL method provides the best coverage and power. But this is only true in MAs of rare events with small sample sizes. For MAs with medium-to-large sample sizes, MH-bDL was preferred. In MAs involving very rare events, Peto-bDL again proved to be the best performing method, but this was sustained across all sample-sizes. However, in cases where heterogeneity is large, performance estimation based on coverage and power were mostly insufficient. We suggest that analysts think more critically about their MAs approach, when applied to highly heterogeneous datasets with very few events, and we encourage them to carry out robust power calculations before considering a meta-analysis.

These findings provide empirical evidence for applied analysts to guide them on the methods that are preferred in MAs involving different rare event scenarios, and to think more about whether random-effects meta-analyses, when applied to highly heterogeneous datasets with very few studies or events, are likely to provide more power than individual studies. We hope that our findings can help communicate efforts toward more informative guidelines and can have major policy implications in relation to the conduct of systematic reviews involving rare events including adverse events.

Taking into consideration the importance and robustness of our findings we believe that Statistical Methods in Medical Research's position on statistical methods in meta-analysis of rare events and adverse events, makes it an ideal forum for dissemination of this research.

We confirm that this manuscript has not been published elsewhere and is not under consideration by another journal.

All authors have approved the manuscript and agree with its submission at Statistical Methods in Medical Research. None of the authors have any financial conflicts of interest.

Please address all correspondence to: Dr Alexander Hodkinson

We look forward to hearing from you at your earliest convenience.

Yours sincerely,

Dr Alexander Hodkinson

NIHR School for Primary Care Research

Manchester Academic Health Science Centre

University of Manchester

alexander.hodkinson@manchester.ac.uk
